# Supplementary material for: Preferences for accessing sexual and reproductive health services among adolescents and young adults living with HIV/AIDs in Western Kenya: A qualitative study
Source: PLoS One. 2022 Nov 16;17(11):e0277467. doi: 10.1371/journal.pone.0277467 (PMC9668131; doi:10.1371/journal.pone.0277467)
Supplement: S1 Appendix — (DOCX) [file pone.0277467.s001.docx]

**APPENDICES**

**APPENDIX 1A: FGD GUIDE**

**Challenges young people face**

1. What are the challenges that young people face today?
2. How do these challenges affect young people’s (i) educational and professional achievements; (ii) socio-economic advancements?
3. How have young people tried to deal with these challenges?
4. To what extent have you people succeeded in dealing with these challenges?
5. What support is available for young people to help deal with these challenges?

**Sexual and reproductive health**

1. What do you understand by the term ‘sexual and reproductive health’?

*(Probe responses as necessary: access to reproductive information, safe sex, contraceptive use, STIs and prevention)*

1. How important is young people’s access to sexual and reproductive health services such as contraception, STI prevention e.g. condoms, PrEP, HPV vaccine?
2. Where do young people currently access information on sexual and reproductive health?
3. Where do young people currently access services on sexual and reproductive health?
4. What is the ideal place/location where sexual and reproductive health services easily and conveniently be access by young people?
5. To what extent are the sexual and reproductive health services offered to young people meet their needs and expectations?
6. What challenges do young people face in trying to access sexual and reproductive health information and services?
7. How does young people being in schools or colleges affect their access to sexual and reproductive health services?
8. How comfortable will young people be discussing their sexual and reproductive health concerns with a lay female healthcare provider of their mothers age?
9. What issues would young people be comfortable discussing a lay female healthcare provider of their mothers age?
10. Where and why would young people want those discussions on sexual and reproductive health to take place?

**Safe sex**

1. When do young people talk about sex with their friends? Do the friends they talk about sex with tend to be of their own gender or different?
2. How do young people talk about sex? (Probe: as joke, show off, serious, giggle, give advice, swap information)
3. When do young people have their first sex? Where/under what circumstances does this first sex happen?
4. To what extent do you think that young people are pressured into sex?
5. Where do you think this pressure for young people to have sex comes from?
6. How do people react if a young woman becomes pregnant/a young man becomes a father?
7. What does safe sex mean to young people?
8. How do we ensure safe sex for young people?
9. What challenges do young people face when trying to promote safe sex?

**Views on potential interventions**

1. How would young people feel if we assigned a lay healthcare provider as a confidant and source of information for their sexual and reproductive health?
2. What would be the attributes/characteristics of such lay healthcare providers to act as sources of information sexual and reproductive health information to young people?
3. What would young people feel about receiving their ARVs together with their sexual and reproductive services?
4. What would be the advantages and disadvantages of receiving ARVs and sexual and reproductive health services together at the same point?

**APPENDIX 2A: IN-DEPTH INTERVIEW GUIDE**

**Challenges experienced**

1. Let’s begin by you telling about yourself? (age, education, marital status, current occupation).
2. What are the challenges that you face as a young person?
3. How do you think these challenges affect your (i) educational and professional achievements; (ii) socio-economic advancements?
4. How have you tried to deal with these challenges?
5. To what extent have you succeeded in dealing with these challenges?
6. What support is available to help you deal with these challenges?

**HIV care services**

1. Describe to me your experience the day you enrolled in HIV care? *(Probe for: when was it; how long was it after knowing that you were HIV positive; where was it; how did you make the decision to enrol in HIV care; what was going through your mind at the time you were making this decision; who accompanied you; what made it easy for you to enrol on that day)*
2. What has enabled you to continue engaging with care at this facility? *(Probe for: close proximity to residence; quality of services at the facility; friendly staff at the facility)*
3. Can you tell me any instance where you ever experienced stigma or been discriminated against because of your HIV status? For example, someone not letting you do something or not wanting to be around you?
4. Tell me anything you know about antiretroviral drugs (ARVs).
5. Tell me about the time you missed taking your ARVs. What happened?
6. Kindly share with me the challenges you facing keeping your clinic visit appointments?
7. Tell me about the last time you missed your clinic appointment.
8. What are your general feelings about HIV care services you currently receive?
9. How do you think your encounter with HIV care services can be made more satisfying?
10. How does being in school/colleges for young people like you affect keeping their clinic appointments and taking their ARVs?

**Sexual and reproductive health**

1. What do you understand by the term ‘sexual and reproductive health’?

*(Probe responses as necessary: access to reproductive information, safe sex, contraceptive use, STIs and prevention)*

1. How important is your access to sexual and reproductive health services?
2. Where do you currently access information on sexual and reproductive health?
3. Where do you currently access services on sexual and reproductive health?
4. What is the ideal place/location where you would like to access sexual and reproductive health services?
5. To what extent are the sexual and reproductive health services offered to young people like you meet their needs and expectations?
6. What challenges do you as a person face in trying to access sexual and reproductive health information and services?
7. How does being in school/colleges for young people like you affect their access to sexual and reproductive health services?
8. How comfortable will you be discussing your sexual and reproductive health concerns with a lay female healthcare provider of your mother’s age?
9. What issues would you be comfortable discussing a lay female healthcare provider of your mother’s age?
10. Where and why would you want those discussions on sexual and reproductive health to take place?

**Views on potential interventions**

1. How would you feel if we assigned you a lay healthcare provider to be your confidant and source of information for your sexual and reproductive health?
2. What would be your ideal attributes/characteristics of such lay healthcare provider to act as your source of information on sexual and reproductive health?
3. What would you feel about receiving your ARVs together with your sexual and reproductive services?
4. What would be the advantages and disadvantages of receiving your ARVs and sexual and reproductive health services together at the same point?
