# Supplementary material for: Preferences for accessing sexual and reproductive health services among adolescents and young adults living with HIV/AIDs in Western Kenya: A qualitative study
Source: PLoS One. 2022 Nov 16;17(11):e0277467. doi: 10.1371/journal.pone.0277467 (PMC9668131; doi:10.1371/journal.pone.0277467)
Supplement: S1 File — (DOCX) [file pone.0277467.s002.docx]

**STUDY: AYA**

**PARTICIPANT FGD**

**Date: 23-AUG-19**

**Venue:** KISUMU COUNTY HOSPITAL

**PARTICIPANT CAREGIVERS**

M What are the challenges that young people face today?

P1 Accessing of the phones and seeing things that they are not supposed to. Previously we could be taught by our grandparents but nowadays we live in towns and it’s hard to get information from them.

P8 Another challenge is that we are not free with the youths. We are normally so arrogant as parents.

P2 Nowadays we pretend to be so busy to the extent that we don’t have time for our children. This makes us not be able to be friends to our children.

P7 There are children whom we cannot know their real character. We only get to know what y they are doing through their friends.

M How do these challenges affect young people’s (i) educational and professional achievements; (ii) socio-economic advancements?

P1 Indeed these challenges affect them especially the girls who may experience sexual abuse at school but doesn’t open up to share with the parents. This may go on until she becomes pregnant.

P2 These challenges makes them not to perform well in schools. I had a sister who stopped going to school. This is another way can be affected.

P12 I would say that there is this issue of our children being influenced in terms of behavior with their friends. I don’t think that it’s the parents who influence the bad behaviors in children but rather we as parents are very strict and would not wish that our children go astray. The only challenge that I see is that we don’t take time with our children.

P8 Let me add on what he has said. There is these thing of peer grouping. I think we should know who our children’s friends are. From this it’s easy to judge our child. It’s us who need to help these children to grow in a better way. For those that are on medication lets us encourage them so that they live with a lot of hope. We should not be harsh.

P1 Just to add on what she has said. Being harsh doesn’t help a child grow up right what we need to do is to stop imagining that whenever they go out with their friends they are going to indulge into sex.so when you are strict on time they can opt to do what they want early and still come to the house early.(Laughter).We should not restrict them on phones but rather tell them the right thing to access on the phones. I remember when my son was joining form one he wanted to have a phone because he was going to a school which was very far. I gave it to him. I accessed this phone when he thought I could not. I realized that he had been accessing pornography. I put him down and talked to him and he changed. I told him that whatever he was seeing their he should just do it practically. (Laughter).I don’t allow them to put passwords on the phone.

P2 Just as she has said I’ve also bought phones to my children, though am very busy because I work in the salon but I ensure that we cook, eat and pray together so that whenever there is a problem I can dictate it early enough.

M How have young people tried to deal with these challenges?

P1 My children have passed through challenges. My son wanted to be recruited to homosexuality with his football coach. He came and shared this with me that he was to be paid six hundred shillings. I talked to the coach and squared this. We even rescued the others who had been recruited.

P8 When we started I talked about openness. There is that one son of mine who comes here and has a lot of exposure. He get a lot of knowledge from William and Mercy and he even challenges me when he comes home. When one who is going through a challenge of peer pressure then when he comes to you then you should help[ accordingly. This will ensure that they come out of the challenge instead of getting deeper into it.

P2 Our children should try and have good friends. Like my son we normally go to Jehovah witness church. We normally have church activities though we don’t have the once that deal with the youth only. Whenever there are activities to be done everyone is always considered to take place and also teachings are given that is helpful to the youth.

P3 I normally see my brother tries to talk to us whenever he has a challenge.

P8 let me add that even us as parents we should have friends that encourage our children. If for example I have friends that are drunkards then we can’t tell the child not to take drugs with alcohol. By this we cannot tell the child to achieve the operation triple zero.so as parents we should look at the friends that we have and what they child can get from them.

M To what extent have you people succeeded in dealing with these challenges?

P8 I can say that my child has succeed in dealing with the challenges but he’s not done it perfectly. This is because the exposure that they get from here.When they come together with others who are also reactive then he feels encouraged how well the others are living positively.

P5 my child has really tried because he used to be very shy. But after meeting others of the same status he is now much open. He also used to have bad company but after being talked to he has really changed.

P7 on those challenges am grateful that the adolescent have a Centre and don’t go to attend the clinic with the adults. This has made them to be together and feel encouraged when they meet people of the same status. There was a time when the teacher asked her about her medication and this really affected her. I told her not to worry because even that teacher was on medication.

P6 Another challenge is my child who doesn’t even know why she’s taking medication.so when am not around she doesn’t take her medication. But from the encouragement from here she has really improved.

P1 What I would like to emphasize on is the issue of having an adolescent Centre. And a I also appreciate the work William and the group are doing.so they really help the adolescents go through their challenges. They get to hear from their peers who are in boarding schools how it’s a challenge for them to take medication there. I would prefer that my child goes to a day school so that I monitor there behaviors and the kind of friends that they have.

P3 The issue of taking medication in boarding school is a challenge because I had a friend who would put her medicine on the handkerchief and run out .so out of curiosity the locker was broken into and everyone now knew that she was taking medication.so when my sister was about to join form one I became really worried of her joining a boarding school.

P6 What I feel is that if the child is to go to school with medication then it’s good that you inform at least one teacher.

P8 The problem is that the teachers don’t have knowledge on HIV and they really discriminate the students based on the status. That’s why I had to transfer my son to a day school.

P11 I think the government needs to write a letter to the boarding schools because I was once in a school and then there was a teacher who came to the assembly and made an announcement that those that are taking medication to move to a certain side and wait there to pick their medication.

M What support is available for young people to help deal with these challenges?

P9 I’m a stepmother and I got this child already on medication. She was nine years by then and the father said that it was hard to sit down a girl to discuss such like things. The problem was that disclosure had not been done.so after several visits and counselling disclosure was done.

M What do you understand by the term ‘sexual and reproductive health’?

*(Probe responses as necessary: access to reproductive information, safe sex, contraceptive use, STIs and prevention)*

P3 Its information given on how someone grows and how to take care of themselves as they go through that stage

P8 we are talking about our adolescents who are our children. I don’t think there is a day that I will tell them that sex is safe. Not unless they are married and are staying in their own houses. Let’s maintain at abstinence in order to help them. Even if we talk of contraception then still it means that we are promoting sex.

P1 What am wondering is that what is the reason for someone who doesn’t have a family to undertake family planning. What family are they really planning?

P8 Another thing is that we are talking about adolescents and family planning. Its like we are setting them free to have sex.

P7 I want to contribute there on the issue of adolescent and family planning. There are more sickness other than HIV that is sexually transmitted. So if the child is sexually active we may only know after they get pregnant.so if they know that is likely not to take place then they will start engaging in sexual activities with even older men.

P12 I think it’s a tricky discussion but I advocate for complete abstinence.

P1 Let us be role model to our children. We should not tell them not to have boyfriends but we keep coming to the house with different men.

P8 I was not good at biology but let me try this. (phone rings) Sexually Transmitted Diseases are very dangerous because it may interfere with our reproductive system and later become an issue if we want to conceive. I normally tell my daughters that I will need to see her boyfriend so that incase of anything I know the person in question.

P11 What I would like to say is that if you suspect that your child is sexually active then the best thing is that you start them on contraceptives. Because I have seen so many school going girls take contraceptives. There is one girl who got pregnant and was given drugs to abort unfortunately she died.

P7 I want to tell my fellow parents that there are other serious illnesses other than HIV. There was a niece of mine who would always seek contraceptives from a private clinic. She later developed signs like epilepsy. This girl who had got very high marks in primary could not concentrate so she failed her form four exams because she was concentrating on prostitution.

P8 I may defend my brother here but not that I want to promote safe sex. But what we should do is to train the children in the ways of the Lord. Am wondering that if a girl is available and the boy doesn’t have money to buy condoms then they may end up having unprotected sex.

P11 I would like to differ with you a little bit. The best thing is to allow them use the contraceptives but under the advice of professionals. This is because the children are being seduced every day. There are others who are even drugged and the sexually assaulted.

P1 I have another opinion. I feel like the youths should be given freedom. We should not think that whenever they go out they are going to engage in sex.

P11 There is a bad stage that the youth have to undergo. It’s the same story that is in the bible about Adam and Eve. Its Eve who put him into temptation. I think we should have trackers in form of spy to our children.

P8 The problem is that when this child gets to know that there is someone spying them then they may end up being rebellious. Even me as wife when I find out that there is a spy my husband has put then I will be very rebellious.

M Where do young people currently access information on sexual and reproductive health?

P8 They see them on the media.

P5 They are taught in school.

P3 in the hospital

P12 They watch them on television.

P2 From parents

M What is the ideal place/location where sexual and reproductive health services easily and conveniently be access by young people?

P1 Hospitals

P3 Clinics like Marie Stops

M To what extent are the sexual and reproductive health services offered to young people meet their needs and expectations?

P8 I believe that hospitals advocate for abstenience.so as much as they access these contraceptives in hospitals they are aware of abstinence.

M What challenges do young people face in trying to access sexual and reproductive health information and services?

P8 I don’t think it’s a challenge because they can get the information from the parents and even in the clinic here.

P3 I don’t think they can access them easily. I do act the set books. i have been to schools. You get that an adolescent who might need PREP but end up not accessing it. There was once one who wanted PREP but was told that instead concentrating on her studies she’s just thinking about sex.

P9 Its hard for them to access these services.

P2 I thinks it’s hard for them to access these services because even the providers who offer the services are also parents so they always enquire what level ibn school you are. From their they conduct counselling and thereafter advise appropriately. They can also tell those that are too young not start engaging in this because in future it can make them hard to conceive. This might make the youth to fear going for the service because of the question that are likely to be asked.

M How does young people being in schools or colleges affect their access to sexual and reproductive health services?

P8 There is a problem with peer grouping of which there are children from rich backgrounds who can mislead others by giving them wrong teachings and information.

P8 I removed my child from boarding school because of issues of stigma and adherence. I brought him to a day school. In this school they have been taught many things. I have been to my son’s school twice when Mercy and the group have gone there to teach them.

P3 I think a part from having a teacher in charge of health issue there should be even a provider picked even from District Hospital here who is there and will give the right information. There should also be someone who is reactive placed there who will understand and give advice to the students,

P1 I know among the teachers there has to be one who is reactive so he should be a representative to the rest.

M How comfortable will young people be discussing their sexual and reproductive health concerns with a lay female healthcare provider of their mothers age?

P8 I’m speaking out of experience because their things that a child cannot discuss with you as a parent but will do it with your friend. Like my boy there are things that he cannot discuss with me but will be very free with my friend.it might be maybe am the one who wronged him and he doesn’t want to argue with me because am the parent so through sharing with a friend it becomes so easy.

P11 There were skits that the government used to organize that was teaching on HIV and Aids. They used to be very educative and informative. But nowadays I don’t see them do that.

P1 I feel that it would be really helpful because I have seen community health volunteers who talk to children and they really open up. I think the issue of age makes them have confidence in you.

P1 What I want to add is that families of single parents like me who have a boy child who is sixteen years. I knew that there are some things that he might be experiencing that I know nothing about. Because of this I took him to a brother that we worship with in church. They really share a lot freely.

P12 This will be a good idea because this means that he/she will have experience.

M What issues would young people be comfortable discussing a lay female healthcare provider of their mothers age?

P5 they can discuss the child s behavior and how they can live well

P4 They can discuss how life is. What to do that is right and what not to do that is wrong.

P9 They can discuss how life is.

P2 There are so many risks currently and our children are at risk.so as they speak on early sex and its consequences.

P10 She can teach her on how to take her drugs and the time to take them. They can also be told on the importance of going to school.

P8 You know that when am sent to the adolescent to discuss something with them (phone rings and p7 walks out) I should have the know how.so having this experienced person is very good.

M Where and why would young people want those discussions on sexual and reproductive health to take place?

P8 I think these children may only talk in an environment where they are free. If we were to bring them here and start a discussion, I don’t think they would open up. This is only possible to those that they are free with.

P2 They can go to a certain park

P8 Even the place that we are sitting in currently is an ideal place.

M Why do you think it should be in those places that you have mentioned?

P8 There is a time way back that we took them to Impala park. Going to such places is very important. This is because they feel happy when they are there. When they also come from there you see that they are relieved.

M When do young people talk about sex with their friends? Do the friends they talk about sex with tend to be of their own gender or different?

P1 When they are at parties.

P6 At school when they sit in groups.

P3 At home.

M Do the friends they talk about sex with tend to be of their own gender or different?

P6 At times when they are of mixed gender.

P3 Girls talk to girls while boys talk to boys.

P8 It depends with an individual like my boy doesn’t talk to girls completely. He feels like the ladies normally talk too much.

M How do young people talk about sex? (Probe: as joke, show off, serious, giggle, give advice, swap information)

P3 At adolescent age they show off. You know when we discuss this as boys or girls there are those that would really want to show he has experience more than the others.

P2 What I want to say is more or less the same with what she has said. I want to say that experience one gets from the first time they will always want to get the opinion of others on their first sexual engagement.so they want to do the comparison.

P1 For boys they are ever pushing each other so that they see who can really seduce ladies.

P12 So when they engage in sex they do it practically but when they talk about it its just like a joke.

M When do young people have their first sex? Where/under what circumstances does this first sex happen?

P7 This one you can’t always know.

P12 This is normally those that are between fourteen to fifteen years and this normally happen when they go to the dancing places.

P3 This is those that are between sixteen to eighteen years. This normally is pushed by peer pressure.

P6 This can happen anywhere. These children normally engage in sex anywhere. This can happen even in the house. You might think that they are playing but they are engaging in sex.

M To what extent do you think that young people are pressured into sex?

P8 You know that when they are in a group and among them there is one person who has not engaged in sex while the others have this will mean that this one who has not engaged in sex is pressured to have sex. They can go an extra mile and give you a partner.

P3 The issue of being searched for a partner is not being currently used but rather it would be an issue of one whom want to fit in the group so he has to do what he others are doing.

P10 There can also be an issue with the younger children joining the group of the older ones and then trying to do whatever the older ones are doing.

M How do people react if a young woman becomes pregnant/a young man becomes a father?

P7 People tend to think of very many different things. They always blame the parents of not guiding the child and hence the pregnancy. Others may say that they were so sure that that particular girl would be pregnant because if how she was behaving.

P2 Others take it as a normal thing.

P12 To me as a parent I will feel so bad because it’s likely to bring disagreement between the families of the children. For the girl if she’s still going to school then it means that she have to take care of the baby instead of going to school.

M What does safe sex mean to young people?

P10 it means that there is no engagement in sex without protection. For those that are reactive it will ensure that there is no reinfection.

P11 this means engaging in sex with protection like condoms to ensure that there is no tramsmition of sexually transmitted disease. This is for those that are knowledgeable.

P8 This is just prevention to them so that nothing happens to them.

M How do young people ensure safe sex?

P3 By asking those that are concern more information about safe sex.

P2 What she has said is right but after getting the information they will need the money to buy the protection so they have to ask for the money from parents. But they will not ask it directly.

P1 So for me it’s different. Am a community volunteer I usually distribute condoms to the youth. So what parents fear is the children indulging in sex but eventually they will haveto.so providing protection is a solution.

P6 Let me say this that I have a son who is in form two right now. I went to where they normally sleep and I found condoms.so I took the condoms and kept them. When he came back I asked him about the condoms and he told me that I should be thankful that he’s using condoms.

P1 My son was also found with the condoms in the bag and fellow students took him to the teacher but he told the teacher that he doesn’t want to have children.

M What challenges do young people face when trying to promote safe sex?

P10 Fear

P4 Shame

P3 Curiosity this is mainly when they want to know how it feels to having unprotected sex.

P11 Lack of information concerning safe sex.

M How would young people feel if they are assigned a lay healthcare provider as a confidant and source of information for their sexual and reproductive health?

P10 This lay healthcare provider will be important because she can talk to the adolescent an make them open up more they would have to the parent. She can also provide a lot of information.

P9 The child may also wonder why such an elderly person has been assigned to her. He/She might feel that this person due to the difference in age between will not know what she is undergoing.

P1 Healthcare provider should be trained so that they have the right information to deliver to these adolescent.

P8 There should be confidentiality between the lay healthcare provider and the adolescents. This will ensure that they maintain the relationship.

P11 this healthcare provider should first come to the level of the adolescents. This will help in creating a rapport.

M What would be the attributes/characteristics of such lay healthcare providers who act as sources of information on sexual and reproductive health?

P8 Loving

P3 tolerant

P8 Able to communicate

P5 Trustworthy

P2 Caring and empathetic

P4 confidentiality

P8 Transparent

M What would young people feel about receiving their ARVs together with their sexual and reproductive services?

P7 I am not agreeing with this because I feel that this should be done in different room because the children that come here are of different ages and the younger once may get into things that are past their ages.

P1 What I know is that issuing of contraceptives will be based on age and the discussion that the child has had with the healthcare provider. A child who is still very young cannot be issued with these services.

M What would be the advantages and disadvantages of receiving ARVs and sexual and reproductive health services together at the same point?

P1 there is an advantage especially for the shy ones who can be given condoms.

P8 The disadvantage is that it’s like these children we are exposing them to sex so they will be sexually active outside there.

P1 I think the two should be put in different rooms so that those that require the other service would just go there.

P8 I think because of shyness someone who needs the service will go because the two services have been put separate.

M Is there any other thing that anyone would like to add?

P8 I would urge the facility to close to the adolescents. There is a time that we complained the providers who were harassing the adolescents and we were called and this was solved. The adolescent center has really encouraged our children and it has made them believe that they are must like any other person. This I have seen in my son.

M Thank you very much for this interactive and informative session

**STUDY: AYA**

**PARTICIPANT FGD**

**Date: 3-AUG-19**

**Venue:** KISUMU COUNTY HOSPITAL

**PARTICIPANT** ADOLESCENTS

**START TIME: 12:53HRS**

**END TIME: 15:00HRS**

M What are the challenges that young people face today?

P8 Discrimination and stigmatization.

M Is this inside the facility or outside?

P8 Outside the facility.

P8 Inadequate love

M How do these challenges affect young people’s (i) educational and professional achievements; (ii) socio-economic advancements?

P8 It will discourage you from drugs.

P9 One cannot concentrate in class.

P8 Discomfort

P8 When somebody doesn’t have adequate love then one is likely to be discouraged in taking drugs because he/she may feel like she’s not loved because she’s taking drugs.

P9 It can lead to stress.

P4 with these challenges someone might be seen as an outcast.

P9 Discomfort when you are around other people

P8 Untrustworthy.you don’t trust anyone because you feel like when you share anything then they will be spread to others.

M How many here have disclosed?

P6 There was a time that I used to carry my medication and then I had to disclose to my teacher.

P9 My teacher came to know of this because when I was being admitted in form one.my mother shared with them so that they could remind me when to take my medication.

P8 I’ve disclosed to the school nurse because that where I keep my drugs.

M How have young people tried to deal with these challenges?

P6 By expressing yourself to the elders and being open to your friends.

P9 When somebody tries to question you on the reason why you take your medication daily then just assume them don’t reply.

M To what extent have you people succeeded in dealing with these challenges?

P9 We have succeeded and I would speak on what she said about speaking to older people. For example, you might have a problem that is really disturbing you but you cannot share with the students cause I believe that our reasoning is the same. The only way will be to look for an older person and share with them.

P9 if somebody insist on asking you why you are taking drugs and what are they for then he best option is just to ignore or lie to them that there are for a certain ailment for example malaria.

P8 If you ignore then they will be suspicious so the best option will be to tell that they are for a different kind of ailment for example I usually tell them I have a heart condition.

P9 At home you can confide on somebody that you trust most.

M What support is available for young people to help deal with these challenges?

P3 I’ve got support from my family. They normally remind me on what time to take my medication.

P4 There is a time in school when members of different schools come as a team to the school and we discussed health related issues. The, knowledge that we got there is always very useful. We discuss things like sexually transmitted diseases and abstinence.

P8 There is support from the government like the DREAMS girls project.

P9 I also get support from my mother because there are thing that I can only share with her solely.

P8 There are the providers who offer to us guidance and counselling.

M What do you understand by the term ‘sexual and reproductive health’?

*(Probe responses as necessary: access to reproductive information, safe sex, contraceptive use, STIs and prevention)*

P9 this means that someone has sex and get pregnant.so the duration when one is pregnant and before delivery then there can be challenges.

P4 I think it deals with private parts and also how to prevent sexually transmitted diseases and the treatment that is available for the same.

M What about in terms of safe sex?

P9 I think safe sex means the use of condom and PREP.I hope you know PREP.

M Who can tell what PREP is?

P5 PREP is a drug that you take when you want to have sex with somebody whom is negative while you are positive to prevent them from being infected.

P3 I have heard of it but I don’t know how it works.

P7 I’ve seen the posters outside the clinical rooms but I don’t know much about it.

P9 I know its prevention from getting HIV.

M Who knows about contraceptives and family planning?

P4 It helps one to plan and avoid unwanted pregnancies.

P5 Contraceptives are those pills that one takes to avoid unwanted pregnancies.

P8 family planning is for those that have a family. it’s usually advise that is given when one has given birth on how to take care and how to plan the family.

m Could you please tell me about STI and prevention?

P3 It is sexually transmitted infections.so if you have sex with someone who you suspect has it then for you to prevent yourself from getting it then you have to use protection.

P4 I agree with what she has said but would add that if a person has STI then he needs to be screened in order for them to get treatment. This will allow them not to spread it to others.

P8 Sexually Transmitted infections.it means that they infect someone’s private parts.

P9 They can be controlled. This means that you can avoid infecting your partner.

M Which ways of prevention do you know?

P5 Abstinence

M How important is young people’s access to sexual and reproductive health services such as contraception, STI prevention e.g. condoms, PrEP, HPV vaccine?

P9 its important because it helps to know one’s health and also inform others for safety. You can tell them that you are reactive but there is a way transmition can be prevented.

P8 its important for prevention of the STIs

P5 Its important because it will help one to abstain and know each other’s status.

M Where do young people currently access information on sexual and reproductive health?

P9 In hospitals for example when you go to the hospital with your partner if you want something for protection then you will be given and the instruction as well

P6 Social support. You will be taught how to take care when having sex.

P5 Some health facilities usually have tents where one can acess these services.

P9 You can also buy them from shops.

P8 You can get them from the pharmacy

M What is the ideal place/location where sexual and reproductive health services easily and conveniently be access by young people?

P3 In hospital because it’s there that you will advised appropriately.

P4 The ones that we buy from shops are not always safe because you won’t get the appropriate information.

P5:P6 Hospital

P8 Pharmacy because there are those people that stay far from health facilities and cannot afford to go there therefore going to the pharmacy would be ideal.

P8 I think hospital is good because you can receive some of the service s for free rather than being charged outside there.

P4 You can get the other services in the hospital but you can buy a condom from the shop. For example, if you have a girl and you don’t have condoms would you leave her to go to the hospital to buy condoms or rather you just buy from the shop. (Laughter)

P5 Buying at the shop or pharmacy should be when there is an emergency but this should be after getting the right information from the hospital.

M To what extent are the sexual and reproductive health services offered to young people meet their needs and expectations?

P9 it depends where you live. You can be given condoms just for safety if the area that you stay in is not safe.

P8 Its good because like the adolescents have their separate clinic where they can access these services.

P8 They have provided us with watches and tins that are marked with days that is helping us take our medication.

M What challenges do young people face in trying to access sexual and reproductive health information and services?

P8 There are those that don’t like to talk about sex and reproductive health issues.so it may be difficult for them to explain to anyone in case they have such a challenge.

P9 You may be afraid to ask for these services because you feel that you may be asked questions that you cannot be able to answer.

P8 There are providers who can be harsh. They don’t give you explanations but rather just give instructions.

M How does young people being in schools or colleges affect their access to sexual and reproductive health services?

P2 in school the other pupils will feel that you are still a kid to access such services.

P6 It’s not a challenge because when you are with your provider what you discuss will remain between the two of you. The challenge only comes in when one has to ask for permission from school to come and access the services. This is because you might need to explain to the teacher.

P8 It’s not a challenge because from our school every Friday we normally have visitors who come to talk to us and they are usually from hospitals. This is usually our Guidance and Counselling day. They normally teach us on how to prevent unwanted pregnancies and HIV.

P8 It’s not a challenge because you can always talk to the school nurse.

M IN your school does the nurse have condoms and can you ask for it?

P8 She doesn’t have them and she will not give you because you are still a child.

M How comfortable will young people be discussing their sexual and reproductive health concerns with a lay female healthcare provider of their mothers age?

P4 It depend on the character of the person. she should not be nosy.

P9 I will be comfortable because she will give you motherly love. She will advise you as a lady on what you need and need not to do.

P8 She can help you in challenges that you have not shared with your mother.

P6 She will guide me like a mother and teach me on abstinence.

P5 She will advise you like your mother and give you the love that you need.

P4 That knowledge that she has will enable her to understand the psychology of the children and even the youth. It will be easy to cope with her.

P3 it will be easy to share with her things that you cannot share with your mother.

P1 I can discuss with her the challenges that I have.

M Where and why would young people want those discussions on sexual and reproductive health to take place?

P1 Around the village in our compound.so that nobody gets to hear what we are discussing.

P2 Around the house in one of the rooms.

P3 I prefer in the hospital because its private.

P4 Anywhere provided we are the two of us.

P5 Private room.

P6 In a closed place either in the hospital or at home.

P7 in the hospital because at home you will be asked what you were discussing once you are through.

P8 Anywhere provided it’s only the two of us.

P9 in a closed room.

M When do young people talk about sex with their friends? Do the friends they talk about sex with tend to be of their own gender or different?

P5 Mmhh we normaly have such discussions after exams and everyone is free.

P9 During games time.

P6 The girls usually discuss alone.

P7 in school both boys and girls had this discussion and they were in groups.

P3 There are those that sit in a group of same gender while others sit in a mixed group

P5 Like us we are in a girls boarding school. You find that when we are having group discussion before or after exams such conversation just emerge. When you hear one of us say that we are going to have discussions about root hair cell that is in biology then most definitely you can always tell where the conversation is heading to.

P6 Every Monday we normally have life skill lessons and in this is where the teacher discusses with us on how to abstain from sex and how to prevent ourselves from diseases.

P8 In our school we are saved(Laughter) we only discuss sex as a topic.

M How do young people talk about sex? (Probe: as joke, show off, serious, giggle, give advice, swap information)

P6 Some are serious while others are not. The once that are usually serious are the once that teach the rest who didn’t know about sex.

P4 This is usually a serious thing that you cannot joke with .By the time we are discussing this in the class we even have to close the door and everyone is attentive.

P5 You know that everyone loves those topics on sex.so when they are started people tend to concentrate. You find that even somebody who has never prayed before a lesson begin will pray before the discussion.

P8 There are those that likes to show off so that they make people believe that they know everything.

P9 You know like our math teacher when people are not understanding he diverts to these topics to bring the attention back. But he normally has good life skill lessons that is important to us.

M When do young people have their first sex? Where/under what circumstances does this first sex happen?

P9 At adolescence

P8 after marriage

P6 Mostly its during adolescence .usually those above thirteen years.

P4 I our school you can know those who have had sex by the way they are talking.its usually from fifteen to seventeen years.

M To what extent do you think that young people are pressured into sex?

P9 sometimes they are pushed by poverty. Someone can make promises of paying school fees if they have sex with them. But in other instance it can be for enjoyment.

P4 Peer pressure.

P6 To express feelings.

P8 idelness

P9 As a stress reliever.

M Where do you think this pressure for young people to have sex comes from?

P4 When you are in a group and they keep discussing how sweet sex is then in most cases you are pushed to have sex so that you can also know how sweet it is.

P6 Nowadays there are parents who have pornographic content on their phones. The children can always access these phones and watch this. From there they will be curious to do what they saw.

P5 When you have friends who talk about se then in most cases you will be tempted to engage into it.

P1 if you see the nude photos then you are likely to engage in sex because they will interfere with you mentally.

M How do people react if a young woman becomes pregnant/a young man becomes a father?

P6 As a girl you can be depressed because your parents have always told you that you should not engage in relationships when still in school. This can lead one to either commit suicide or try abortion.

P8 It will be bad because as a boy will be drop out of school and then use your school fees to take care of the child.

P6 it will not be nice because this will mean that the girl you will be a child who has a child. You will not have any experience on how to take care of the child. As a boy you will not be able to provide the needs of the child.

P4 One of the participant has said that the boy will drop out of school I want to disagree. i think the girl will just carry the burden alone. (Laughter)

P2 It can break the relationship between you and your parents.

P9 The girl can be treated as an outcast.

P3 I think both the girl and the boy should be blamed because they were in agreement when they were engaging in sex.

M What does safe sex mean to young people?

P9 Safe sex means having sex after getting the right guidance from even a clinician.

P4 This is having sex with prevention.

P8 Safe sex is sex that one does but cannot be able to be infected with Sexually transmited diseases. This is by use of condoms.

M How do young people ensure safe sex?

P9P3P5 Abstinence.

P8 Use of protective measures e.g. condoms.

P8 Being faithful to your partner.

P4 So how can an adolescent be faithful? What I know is that only married couples can be faithful. You might have a girlfriend in high school but get another one at the university.

M What challenges do young people face when trying to promote safe sex?

P9 You may not be confident enough to talk about it.

P6 There is a challenge because if you want to talk to your fellow youth and give information on safe sex they may have a filling that you have been having sex.

P4 You may find that you are talking to someone about safe sex but in their mind they feel that they must always have sex without protection.

M How would young people feel if they are assigned a lay healthcare provider as a confidant and source of information for their sexual and reproductive health?

P9 It will be good because there are things that you would be more free to tell your friend other than you mother.

P8 It will be good because there are parents who are very harsh so I think this one will be very understanding.

P7 It will be good because you may find that there are parents whom you talk to but just ignore.so this one will listen to you very well.

P6 There are parents who really don’t care but this lay healthcare provider will listen to you.

P5 it will be good because this lay healthcare provider will have experience and thus you will share a lot.

P4 It will not be good because this is a person who doesn’t know my background so well.it unlike my parent who knows me very well. There is a way I will express myself and he will not understand.

P3 It will be good because you can share with her relationship issues that are bothering you.

M What would be the attributes/characteristics of such lay healthcare providers who act as sources of information on sexual and reproductive health?

P1 Self control

P2 Loving

P3 Be confidential

P4 Loving and caring

P5 secretive

P6 Honest and faithful

P7 Kind

P8 Honest and trustworthy

P1 Dress decently not wear fitting and short clothes

M What would young people feel about receiving their ARVs together with their sexual and reproductive services?

P5 It would be good because the same person who giving you arvs is the same one you will share with if you want reproductive health services such as condoms.

P9 I don’t think that they should be together because you find that your friend might access these services and next time when you go for there she might think that you have gone there to access reproductive health services the way she did previously.

P8 They should be put separate because there are children who go for ARvs and might be spoilt if they see these other services.

P6 It should be separated because there are those children that are not mature so when they get into the rooms and see condoms they will started querying.

P4 I prefer it should be in one place rather than walking from one room to the next if for example you were with a friend then they will start wondering how many diseases you are suffering from.

M We have come to the end of the interview. Thank you for your tine.

**STUDY: AYA**

**ADOLESCENT FOCUS GROUP DISCUSSION**

**DATE: 28-AUG-19**

**VENUE: KISUMU COUNTY HOSPITAL**

**START TIME: 14:20HRS**

**END TIME: 15:18HRS**

M What are the challenges that young people face today?

P3 Peer pressure

P4 Stress.

M Mmmm, tell me more

P4 Sometimes people go through things that they don’t like. So, you find that they end up getting stressed

M Mmmmm. Things like what?

P4 Maybe like of money….

M Okay. P3, tell me more about peer pressure.

P8 Some people would wish to be like other people like what they are doing of be in one particular group so this leads to peer pressure.

M P2?

P2 Laziness

M How is laziness a challenge?

P2 Some people may be assigned some work but they may not do it.

P5 Substance abuse. Most of the youths nowadays like to indulge on modern lifestyle like the western world. They engage in drugs that change how their body functions.

M When you talk of substance abuse, which particular ones?

P5 Mandrax, Cocaine…..

M Mmmmm P6?

P6 To me its sexual addiction because most of the boys and girls start having sex at an early age.

P1 Fear because when you are told to do one thing and you don’t want because you do not know how to do it.

M (Summarizes the points mentioned). Do you think these challenges affect young people?

(All participants) Yes…….

M How do these challenges affect young people’s (i) educational and professional achievements; (ii) socio-economic advancements? P6?

P6 This leads mainly in drop out from school because of the things they start doing at the wrong things like getting into drugs and sex.

This also leads them into stealing because they drop out of school and so they have to look for money to accommodate themselves.

P7 When you are assigned a duty with the parents but instead they spend time with their friends and ends up not performing the task that was assigned.

P4 some people will drop out of school while others can commit fornication and also engage in bad behaviors.

P3 They start failing in their academics due to peer pressure. They tend to think that they don’t fit well in a particular group there are people who do not want to associate with them.

P5 Like what I was saying on substance abuse. In Kenya there is increased incidences of cancer for example, the lung cancer which may be caused by taking tobacco and bhang. It affects the liver.

M So, it affects their health too?

P5 Yes

P1 Bad behaviors. When one engages in sexual and substance abuse he/she will develop bad behaviors. Those who abuse drugs become very abusive and violent

M How have young people tried to deal with these challenges?

P2 Some people have gone to rehabilitation centers.

P3 There are people who are free with their parents so whenever they face a challenge they go to their parents to seek advice

P4 Like fear you can control it. You can be advised on how to talk to people and then with time you overcome it.

P6 Those who are affected with the challenges are trying to take up the challenges and strive to be better.

P5 The youths now are trying to talk to the government through the relevant authorities but the government is not helping. For example, there is a song on the plight of the life in ghetto which was banned. But basically, it was trying to say what kind of lifestyle that the youth live.

M What do you mean by the term Gheto? There are people who may not understand.

P5 Ghetto is a group of people who live a low life in a community and do not have better housing.

P6 I think one of the challenges that we face is that of the youth getting into early pregnancy and they try to get a solution by aborting which is not the right way.

M To what extent have you people succeeded in dealing with these challenges?

P8 As for me I think that if they had bad company they can quit and they can choose the right path to follow.

P1 By avoiding the bad behavior’s which some may have.

M What support is available for young people to help deal with these challenges?

P6 Mostly we can’t say that there is support because on social media those parents that are supposed to guide us are the ones making us go the wrong way. They post wrong songs that don’t encourage but rather discourage.

P3 I think there is support because if someone has a problem and goes to another person he/she can be guided. So its upon them to choose which path to follow

M What do you understand by the term ‘sexual and reproductive health’?

P6 I am not very sure but I will try.

M Ooh yes, sure

P6 I think it’s the way people have sex and the way they are being taught to have that sex.

P4 I think it is all about how to prevent yourself from diseases.

P1 I think it’s about managing oneself especially girls attending cervical cancer screening.

M Good. Any other definition?

P8 As for me I think it what teaches us on the cleanliness of the reproductive system.

M What do you know about PREP?

P4 PREP is used when one partner is positive and the other negative, so the negeative one will take the medicine to prevent them from being infected with HIV when they are having unprotected sex.

M Great. You all have an idea of what sexual and reproductive health is. Generally, it encompasses safe sex, prevention of STDs, use of family planning, PREP, cervical cancer screening.

How important is young people’s access to sexual and reproductive health services such as contraception, STI prevention e.g. condoms, PrEP, HPV vaccine?

P8 I think it can prevent certain diseases like HIV and STIs

P5 Access is very important in that it will make them know their status and thus able to protect themselves and those and those around them.

P2 It leads to increase in the standard of living because when you access the services they will tell you how to prevent the STIs and the STDs.

M Where do young people currently access information on sexual and reproductive health?

P6 Mostly they do get the information from hospitals

P4 Television

P1 Some get from schools

P8 Radios

P5 From parents and guardians

P3 In school during guidance and counselling

P4 In newspapers

P6 Social media

M Where do young people currently access services on sexual and reproductive health?

P1 In hospitals

P6 I think some people buy the condoms from the shops.

P3 Pharmacies

M What is the ideal place/location where sexual and reproductive health services easily and conveniently be access by young people?

P6 In the hospital because the hospital is ever there and available for them. So, they can go there anytime and any day that they want. So, it is accessible.

P5 In the hospital because there are medics and personnel that are well informed of the services.

P1 It’s in the hospital because that’s the place where they get encouragement and other services they need.

M To what extent are the sexual and reproductive health services offered to young people meet their needs and expectations?

P6 Yes at some point because family planning helps in preventing unwanted pregnancies. So, it helps them to focus on the future. Maybe one might not be ready to have a child at some point but rather prefers in the future. Thus, it meets their needs and they are able to complete their education.

P5 I don’t think because someone might come and get the services but when he/she goes outside they change their mind.

M What challenges do young people face in trying to access sexual and reproductive health information and services?

P6 Embarrassment.

M Tell us more about embarrassment.

P6 This because I can’t even ask my mother on how people have sex.

M Mmmm, why not?

P6 One fears and feel embarrassed .

P3 I think some people might feel ashamed for example someone may want a condom but due to shame and the thought of what that person might think of them they end up not asking for it.

P4 I think shyness will make one not to do what she wants to do.

P3 I think I may have a challenge in buying condoms from a shop or chemist because I might be worried of what the shop attendant will think of me.

M How comfortable will young people be discussing their sexual and reproductive health concerns with a lay female healthcare provider of their mothers age?

P8 I will be comfortable because she has gone through this adolescent stage and experienced what am going through.

P5 I will not be comfortable but rather be comfortable with someone much older and of the same gender as me. (Male participant).

P3 I will be comfortable with my sister because I can share with her freely. I will not be comfortable with someone who is a stranger.

P2 I will feel comfortable with someone who is older and is of my gender because they had passed through the stages am passing through.

P1 I will be comfortable with someone who is older and is of my gender.

P6 I would prefer someone who is my mother’s age who is a stranger to me. This is because after sharing I will not see her neither will she see me.

M What issues would young people be comfortable discussing a lay female healthcare provider of their mothers age?

P3 I think I will be comfortable discussing everything with a younger person.

M What is everything? You can give exaxmples

P6 As for me if I have not tried sex and I want to do it then I will ask how should I go about it. I will also ask how I may prevent pregnancies and sexually transmitted diseases. Also, how can I handle myself during sex.

P4 I think I can discuss things like menstruation because once you start experiencing it there is always fear of unknown.

M P5?

P5 Challenges that comes with having relationships with the opposite sex.

M Where and why would young people want those discussions on sexual and reproductive health to take place?

P6 Hospital

P2 In school during guidance and counselling sessions.

P1, p4 Hospital

P5 Disclosed place

M Disclosed place like where?

P5 Anywhere as long as it is private

P3 Private place where we are only the two of us.

M Why does everybody prefer the hospital?

P6 This because if I tell my parent that am feeling unwell, I want to go to hospital then she will not deny me neither will she start doubting. Once I get there then am the one who knows which services that I would like to access. This is because if I go to a private place then she will have so many questions.

P8 I will prefer the hospital because first of all nobody will see me

P3 I would prefer a private place because there will be just the two of us and nobody will be listening to our conversations.

M When do young people talk about sex with their friends? Do the friends they talk about sex with tend to be of their own gender or different?

P6 I think it comes when peer pressure sets in. They may tease someone of being a virgin and from there is where these conversations start. Those who have had sex tells the rest who might be tempted to engage in sex. Mostly for girls they start to have the conversation once they start experiencing their menses.

P2 I think in groups with people of the same gender.

P5 In groups because somebody like me I have different type of friends. Like those who use drugs (addictive drugs) once they are high on it their conversations start building up on sex unlike those who are sober who might only talk about a girl being attractive.

Mostly the discussion always involves both genders.

M How do young people talk about sex? (Probe: as joke, show off, serious, giggle, give advice, swap information)

P8 As for me it’s like a form of a joke.

P4 It’s always like a joke because someone might suspect that you may not like that kind of discussion.

M When do young people have their first sex? Where/under what circumstances does this first sex happen?

P8 I think they engage in sex for financial gain like raising school fees.

P6 The ages at which people have sex is unpredictable. Some at 14 or 16 years old.. I think boys just love sex. Someone may get to high school without engaging in sex but once they get there, they engage in it.

P5 It varies with the background someone comes from. Those from well off background will go to high school that are rated highly. They mingle and discuss about sex and eventually have it.

In the slums you might find a girl who is nine years being raped with a fifteen year old.

P4 I think also boys may caress a girl and they end up having sex(laughs)

M To what extent do you think that young people are pressured into sex?

P6 At some point when you are sent from school to get school fee and you just stay at home and the school fees is not there. So, you start getting into sexual activities to get money.

P4 Maybe if you cannot get pocket money from your parents and you have a boyfriend then most likely you will engage in sex so that you can get what you want.

P5 I don’t think you can be pressurized to have sex especially if you have your way of doing things. If you are swayed by other people’s opinion then you can have sex but if you have your own principles then you cannot.

P3 I think for girls if they come from a poor background and cannot afford even sanitary towels then she might be forced to have sex with the boyfriend so as to buy them.

M How do people react if a young woman becomes pregnant/a young man becomes a father?

P6 It is something that is embarrassing. If it is somebody like me who has not finished school, people will start wondering how I will feed the child.

P8 Some people will bad mouth them saying all sorts of bad things.

P5 In my own opinion I don’t care (Laughter)in our class there is a girl who is pregnant. People are talking about it and others are visiting her and even taking gifts to her. But I don’t think that should be the case because she got pregnant out of her own choice.

P4 I think the girl will be shocked and also shy.

P3 I think as for me I will lose my dignity but for the boy he will continue going to school. People will discuss more about the girl because the girl child is more important.

P2 The girl will be embarrassed and if she stays in a homestead or even estate they will start backbiting her.

P1 The boy will be shy and his friends will start talking about him.

M What does safe sex mean to young people?

P6 Safe sex to young people means preventing ninety-five percent of diseases and unplanned pregnancies.

M P5?

P5 No

M How do young people ensure safe sex?

P2 By using condoms

P3 Family planning

M What challenges do young people face when trying to promote safe sex?

P6 Mostly those who have experienced sex say that at some point the condom might burst and the lady might get pregnant.

P5 People will start seeing you in another way. for the girl if she wants to use a condom like condom then the boys tend to think that they will not have pleasure during sex.

M How would young people feel if they are assigned a lay healthcare provider as a confidant and source of information for their sexual and reproductive health?

P8 They will feel good because they will get a chance to get more information.

P6 They would feel free and it would be affordable because if they are assigned they won’t need to pay.

P4 It will be easy and good.

P1 It will make the person to feel free. Share out all his/her problems.

M What would be the attributes/characteristics of such lay healthcare providers who act as sources of information on sexual and reproductive health?

P8 He/She should be kind, understanding

P6 To me I think the person should be honest.

P4 The person should be confident and courageous.

P3 The person should be encouraging.

P2 Patient.

M What would young people feel about receiving their ARVs together with their sexual and reproductive services?

P6 I think they will feel comfortable.

P8 For me I will feel bad because it’s ashaming

M What do you mean by ashaming

P8 They will feel shy

P5 I think it will be better because it will be saving time since you will not have to see different providers for the different services.

P5 I think it will be better because when you receive the services at the same time.

M What would be the advantages and disadvantages of receiving ARVs and sexual and reproductive health services together at the same point?

P6 The advantage is that it will save time. The disadvantage is that some people may shy off.

P4 as for me I feel that it’s easy to give both services.

P3 I think it’s an advantage because when you are receiving these services you will also be advised on the use.

P1 The advantage is that it will help one with the knowledge he/she doesn’t have.

M Thank you very much for your time. Any questions?

P3,P5 None

M Okay. Thank you very much for your time.

**STUDY: AYA**

**FOCUS GROUP DISCUSSION WITH AYA CAREGIVERS**

**DATE: 23-MAY-19**

**VENUE: LUMUMBA HEALTH CENTRE**

**START TIME: 14:41HRS**

**END TIME: 16:04HRS**

M Good afternoon. My name is XXXX

Welcome to today’s focus group discussion with the caregivers/guardians to the adolescents and young adults. Our note taker is XXXX.

M Generally, what are the challenges that young people face today?

P2 One of the challenges is of lack of understanding. You find a very young girl is getting married to an old man of above 70 years old.

P5 Okay they lack motivation from the parents or whoever they are staying with. Most of the time you find that most of them go to work and they don’t create time for their children and so most of the time the adolescents are alone and think for themselves so they do what they think is right. They lack advise from the adults who are supposed to be responsible for them.

P6 I don’t know how I can explain but some of them are just ignorant

M In terms of what?

P6 In terms of social life.

P7 Some of them don’t get counselling. They grow up to the adolescent level without knowing what they should be doing. Their parents do not take time to talk to them on what they should expect when they get to adolescent. Like body changes among others…

P4 What I can say ….. I don’t know how I can pass my point.

M Just say it in a language you are comfortable. We will understand.

P4 (Speaks in dholuo…)We should keep these adolescents busy and in addition to that we should offer them counselling to let them know of their healthcare. We should be free with them and guide them on how to take their medication.

M (Translates what P4 has said)

P3 I can say that nowadays it’s really hard to get free time to talk to our children. These children get friends from their schools who misadvise them. This can make a child develop weird characters hence interacting with them in the house becomes very difficult. A child comes back from school and starts questioning why he/she is not being treated well like a fellow child who probably comes from a well off family.

P1 What I can say is that there is social media influence. The young adults who can access social media are being misled by the information that they get there.

M (Summarizes what the participants have said.) P 5-Anything you want to share?

P5 Whatever I wanted to share has been mentioned by my fellow participants. Peer pressure…

M Great. How do these challenges affect young people’s education, professional achievements and socio-economic advancement?

P3 By the time a child is growing up the parents try their level best to bring them up well. But the problem is the friends that they get who influences them hence they don’t perform well in terms of education. There are others who are always sent back of for school fees. They can decide not to perform well due to this. And you see in Kenya today there is massive unemployment. This always discourages the children from working hard because they see so many people who are educated but lack jobs.

P6 These challenges make them not to perform well. The issue of peer pressure. You will find that the child will always be with the friends. Even you as a parent when you tell them to spend more time on education than with the friends they tend to disagree.

P5 As a result of peer pressure, you will find that they engage in sexual activities for money because the peers will tell them that this is an easy way of getting money without asking from the parents. This will be because of the influence from the others.

P1 There is lack of good communication between the caregivers and the young adults. This makes them rebellious and tough headed. Some even start black mailing their parents and their lives does not go on well.

P2 You get that they might start engaging in drugs and substance abuse. This affects their school performance and some may end up in prison.

P7 We have various social lives. A child who is from low class can meet one from high class and then admire that lifestyle. She might want their parents to put them in that class. This can affect a child so sometimes it’s good that you put down a child and talk to them about the issues of class.

M Great. To what extent have young people succeeded in dealing with these challenges?

P5 To some extent some have. A child tells you that he/she would like to have a lifestyle similar to that of the friend then it means that he/she has seen that he cannot solve the problem alone but engage the parents. They have probably tried to address it with then peers but have not found a solution. Hence the parent is there to advice accordingly.

M Mmmmm P2, do you think the AYA have tried to deal with their challenges?

P2 It seems that they have not been able to solve these problems because you might get that there are others who might even commit suicide. Majority may have tried to deal with them but there is also a number who are not able to cope and deal with these challenges.

M What support is available for young people to help deal with these challenges?

P4 There is support where the parents are able to discuss with the child on what they are able to offer them and what they cannot. They try and make the child understand their situation.

P6 I think the other support will be in the clinic where these young adults normally attend. They are categorized based on their ages and they discuss their experiences and encourage one another.

P5 In schools

P2 In churches

P1 I just wanted to add that in the health centers we can find counselors who can provide very good advice even more than the parents.

M Any other support available for the AYA to deal with their challenges? P7?

P7 These peers can be involved in some trainings as peers in a group so that they can be able to know what is expected of them.

P3 Those that are in care then there should be meetings in the year and have a discussion on what is affecting them. They should also be given target on their activities for the year and motivation. This will really be helpful. In addition, as healthcare providers, you can organize sessions where you go to various schools to talk to these youths. As parents we need the support of the clinic and also the schools to be able to support our adolescents and young adults.

P6 Parents can also come together for a meeting and be taught on ways in which they can handle these young adults. Apart from that teachers and also matrons can also be trained.

P5 And to add on that there could be a group of adolescents who have a support group you can bring in a motivational speaker at least twice or once a month. This will encourage the adolescents and young adults.

M P2 You seem like you want to share something…

P2 Yes. To add onto that I can say that when the adolescents are corrected in a group it’s easier than when it’s done individually.

M Okay. We are moving to the next section on sexual and reproductive health. What do you understand by the term sexual and reproductive health?

P1 I understand it that it’s when somebody starts to feel hormonal change in the body and wants to get into intimate relationship with the opposite sex.

M P6

P6 Pass it on to P5

P5 I think this starts from the puberty level from nine years or even earlier This is when they realize the body changes like the breasts

M Mmmmm. P7?

P7 These are changes that occur in an adolescent and this starts at the puberty stage.

M Great. When we talk of sexual and reproductive health, we think in terms of access to sexual and reproductive health information, access to STD/STI information and services, access to contraceptives, cervical cancer screening, HPV vaccine, safe sex, condom use and STD prevention among others….

How important is young people’s access to sexual and reproductive health services such as contraception, STI prevention, Prep HPV vaccine? P5?

P5 This will help them to know the effects of these STIs at an earlier age. The information they get they can also pass it to others to also change their behaviors.

P1 The information also can help them to take care of their bodies.

P2 It helps in prevention of transmission of diseases like gonorrhea.

M Right.. P3?

P3 Access to sexual and reproductive health services and information is important to adolescents as it helps prevent early pregnancies among the girls and sexually transmitted diseases.

P4 It will help those that are taking drugs so as to be able to adhere well.

P6 I think it’s very good for them to access these services because it will create awareness. Most of the adolescence don’t know a lot of things. When they access these services they are taught the right things.

P7 It makes them share out with other peers. They act as advisors to their peers.

M Where do think young people currently access information on sexual and reproductive health?

P5 (Laughing…) Social media

P2 Churches

P3 Their friends

P4 Their phones

P1 Health facilities

P7 When they are attending adherence sessions.

P5 Some even get the information from their parents/care givers from home from their parents or even care givers.

P1 Some get the information from their fellow peers.

M Where do young people currently access services on sexual and reproductive health?

P5 They do buy from the pharmacy/chemists

P2 Health facilities.

P6 Some fo their friends give them

M Where else do they get the services?

Silence……..

Okay. Where should be the ideal place where they access sexual and reproductive health and services?

P2 Health facilities.

M Why?

P2 This is because the health facilities have people who have the right training and information.

P3 The hospital is ideal because in case they have any questions then it can be answered.in the social media one can also get information especially those that don’t have the courage to go to hospitals or even ask their parents.so on social media they can easily google. They can also go ahead and teach others who are not on social media.

P1 I think with the adolescents they are not free to receive the services at the hospital because they feel ashamed so they should be provided with the services in school because even when they come to the facilities and see people who are mature than them they feel ashamed.

P1 In school.

P5 I think it should be in school with one person who is qualified to give these services. And this person should go there to also give advice because they should also be able to give psychosocial support.

M To what extent are the sexual and reproductive health services offered to young people meet their needs and expectations?

P7 Some but not all some people are taught but ideally, they don’t make use of the services offered. You find that a person may be taught on the importance of condom use but they do not.

P1 Some services are expensive like the HPV vaccine so they cannot afford hence they do not meet their expectations.

P2 It has really helped because there are those that did not know how to use the services but they have been taught and now they know.

P5 You know at this stage the children are at the stage of trying so many things to find if there are benefits or not so, this education should be started earlier before they reach teenage. This will help them have the knowledge early enough.

M What challenges do young people face in trying to access sexual and reproductive health information and services?

P7 The most challenge is the issue of fear for example when the adolescent wants to take condoms then they will start to think of others perception about them.

P6 Apart from the challenge of fear there is also an issue of providers’ attitude where they are ignored based on their age whereas they really need these services.

P5 Just to add to what she has said I think they just lack people who understand them.

P3 The challenge that especially girls get is that when they go for family planning services is that they are usually asked why they are seeking these services but they are still school going so this makes them not to access the services.

P2 Another challenge would be that when the adolescent access these services then most likely they will not concentrate on their education but will always concentrate on these services.

P1 There is also lack of cooperation from back home. The teenage will want to use the family planning methods but the parents might not be wiling. this will make it hard to take these products home. This will hinder their freedom of accessing these services.

M How does young people being in schools or colleges affect their access to sexual and reproductive health services?

P1 Yes it does because there is a time that they should come back for services but they are in school. This may make them miss these services because in school there may be no such services.

P3 It can hinder them from getting the services because some may fear to face their teachers to seek permission to go and access these services.

P4 It’s a challenge especially for those that are in boarding schools. This not only for sexual and reproductive health only part also on ART. They may have their drugs to school but not take them mainly because of stigma.

P6 It affects especially those in boarding.

P7 It would affect them because the teachers nowadays are very strict and it hard for them to give permission to the students to come back home.

M How comfortable will young people be discussing their sexual and reproductive health concerns with a lay female healthcare provider of their mothers age?

P1 I think the girls will be more comfortable with a female healthcare provider because they know that that provider has passed through whatever they are going through and has experience.

P5 That will depend on how the provider will treat them and the training the person has concerning what they can deliver. Only if you can get into their shoes then they will understand you.

P2 Let me say that age really matter because if a fifty-year-old talks to a fifteen-year-old then most likely they will not understand each other because there will be a lot of fear.

P3 To add on what he has said if they can be of the same age then most likely they will be open unlike an older person which can be a challenge.

P7 It will only be possible if they are almost of the same age because a girl might have so many questions to ask but will not be free to ask if it’s an elderly person. Then you need to create a rapport first so that even if they ask sensitive questions then they can respond.

P5 To add on that they normally look at the dress code. If you come dressed in a suit then they will not feel easy to communicate. They will in most cases prefer somebody who is of their age.

M What attributes would young people want in a lay female healthcare provider of their mothers age?

P5 someone “dot com” (somebody modern who dresses like them and speaks sheng)

P3 They would prefer someone who is casual you know that nowadays the young people cannot be free with somebody in a suit.

P2 They will prefer somebody who is well trained not somebody who has just been picked from the village.

P1 I think they would also prefer somebody who is very social with them and also someone who gives them time to air their views.

P6 They will also want someone who listens to them.

P7 You have to put yourself to be of their age.

P5 Somebody who is non-judgmental

M Where and why would young people want those discussions on sexual and reproductive health to take place?

P6 Anywhere where they are alone and not where there are adults.

P1 Somewhere away from home where the parents are not there.

P5 Most likely from schools.

P2 somewhere private where they are with their peers only.

M What issues would young people be comfortable discussing a lay female healthcare provider of their mothers age?

P1 They would want to know how it feels to be pregnant and how labour is.

P6 Menstrual cycle.

P7 They would want to know the consequences of early pregnancy.

P2 They may want to know the effects of drugs.

M When do young people talk about sex with their friends?

P1 Maybe when they are in a group.

P7 When they are in the support group for the adolescents.

P5 When they are with their peers

P3 When they are with their friends.

P2 When they are together in seminars.

M Do the friends they talk about sex with tend to be of their own gender or different?

P7 I think of different sex because they want to compare.

P1 I think they usually discuss with those of the same sex because they want to know if the feeling is the same.

M How do young people talk about sex? (Probe: as joke, show off, serious, giggle, give advice, swap information)

P6 I think they talk in form of a joke so that they get the opinion of the other one.

P3 In school they may ask their fellows if they have boyfriends and what they get from them.

M When do young people have their first sex? Where/under what circumstances does this first sex happen?

P6 It depends some of them start it at a very early age while others get to it because of the circumstances like the death of a parent which really made them engage in sex early as six or seven years.

P7 A child can be raped.

P5 Some of them start it as a play like acting to be the mother and father. Some also imitate from what they normally watch on the television.

P1 I think they engage in sex as soon as they start a relationship with one of the opposite sex.

M To what extent do you think that young people are pressured into sex?

P3 Through friends because you have a friend who has a boyfriend who can advise you to also get one. Through this you engage in sex because of that pressure.

P5 Okay you can find that an adolescent may be from a poor background but there are things that she may lack but really need. So, in that case they engage in sex in exchange for something that she really needs.

P3 Pressure can come from their friends who are sexually active.

P6 Pressure can also come from some parents who are careless with their social lives especially the single mothers who bring their boyfriends to their houses. So the child feels that it’s okay for them to do whatever their mother is doing.

P7 It comes from the little knowledge they get on sex.

M How do people react if a young woman becomes pregnant/a young man becomes a father?

P3 As a guardian I will have a lot of thoughts on this. For the first time when am told about this I will really be in shock.

P5 First of all the blame will be on the parent or guardian for not advising and bringing up the child well. The blame always lies with the mother.

P1 It will be seen as something that is shameful.

P2 You can be so mad with the child but later start that you are the one who did not give the right advice.

M What does safe sex mean to young people?

P7 Safe sex means that one engages in sex without getting STI, Pregnancy and other infections.

P1 I think to young people safe sex having sex without getting pregnant.

P2 Safe sex is engaging in sex without getting any diseases.

M How do young people ensure safe sex?

P7 The use of protection.

M What challenges do young people face when trying to promote safe sex?

P1 Yes there can be challenge with people of the opposite sex because one person might be willing to use condom but the other may not.

M Why would they want not want to use the condom?

P1 From what they hear that it’s not sweet so they want natural sex.

P2 So many challenges always arise from the debate on whether to use condoms or not. Another challenge is on how to use it.

M How would young people feel if they are assigned a lay healthcare provider as a confidant and source of information for their sexual and reproductive health?

P6 I think it will be okay for them because they will find someone who they can open up to.

P7 There are issues which will be addressed with them.

P5 They will feel secure because they will have a feeling that there will be someone for them.

P4 If there can be someone who is for them then they can be free because they have someone who can talk to them at a personal level rather than being in a group.

P3 It will help them to share their problems especially those that have personal problems.

P1 They will have confidence in sharing their issues.

M What would be the attributes/characteristics of such lay healthcare providers who act as sources of information on sexual and reproductive health?

P7 It should be somebody who they can confide in.

P6 They look at the age

P5 Somebody who can understand them and suits to their feelings.

P1 I think just as p5 has said it should be someone who is confidential.

M What would young people feel about receiving their ARVs together with their sexual and reproductive services?

P6 It will be okay but it depends on the on the age of the adolescent. This will be fine because it will save on the time.

P1 it will be good for them because they will get both services at the same time.

P2 It will be good because it will help them access those services.

P3 It will be helpful because as they go to pick the drugs they can ask questions on issues of sexual and reproductive health.

P4 It will be helpful because the adolescent can be able to share everything that they wish to know.

M What would be the advantages and disadvantages of receiving ARVs and sexual and reproductive health services together at the same point?

P2 When these services are easily available then they will engage in sex more often.

P1 I think getting this services at the same time will make them more free.

P5 Just as she has said. something that has a positive state must have negative one. But on this case the negative is minimal. They will feel like they are free to indulge in sex because they are being provided with the services. Those who don’t know can also be tempted to try.

P7 (same point as p5)

M Thank you so much we’ve come to the end of our discussion unless there is any question. The time is four minutes past four.

STUDY: AYA

FOCUS GROUP DISCUSSION WITH ADOLESCENT CAREGIVERS AT KCH

DATE: 29-AUG-19

VENUE: KISUMU COUNTY HOSPITAL

START TIME: 15:13HRS

END TIME: 16:37HRS

M What are the challenges that young people face today?

P5 I have a sister in a boarding High school. We told her not to share her HIV status with fellow students as they will stigmatize her. She can only share her status with their teacher whom can give her permission whenever she wants to go to hospital. Unlike the students, teachers will not discuss her HIV status with other people.

P1 To me just like my friend has said. If you don’t tell the children why they are taking medication then in most cases they will not take the drugs. They will keep the drugs in the box and not take them as required. I’m grateful for my son because when he went to high school the guardian that he got was also taking drugs so I confided in him. He assured me that my son will take the drugs as prescribed and on time. He has not had any challenge with the medication because whenever he has run out of drugs, he seeks permission to come for them.

P4 There is a challenge especially for children in boarding schools. I have a daughter who was studying in Lwak girls, when it was time for taking medication, she used to become worried because the other students would ask why she has to take medication everyday. This made her to default hence her deteriorating health. She was admitted in hospital and that is then time I found out that she was not taking her drugs. We were forced then to transfer her to a day school.

M Any other general challenges adolescents and young adults go through?

P6 Challenge that can be is that if you meet someone you know when you are in the company of your child around this adolescent area then if their child goes to the same school as your child then he/she will go and share with their child. This happened to my son and I forced to relocate because everyone knew that my son was on care.

This will make the other kids in the school discriminate and even mock the child.

P3 Some of the adolescent face the challenge on disclosure. You find that when they get into relationships then it becomes hard for them to disclose to their partners.

P1 I just wanted to add something on disclosure. You find that nowadays children are told about HIV when they are still young. You find that children even play discussing this. Disclosure should be done appropriately and at the right age. There is my nephew whose mother died and has been living with my mother. He was on medication for a long time. He went to hospital and was disclosed to. He was told that he had HIV and that was what his mother had succumbed to. From that day he stopped taking drugs and even ran away from home. So, when we are doing disclosure, we should do it very well so that adherence is good.

M Great. Other than HIV, could we discuss other challenges the young adults and adolescents face?

P4 The challenges that I have seen with these youths is that they engage in relationship but they don’t tell their partners their status. I usually advise my child to be cautious so that he doesn’t mess somebody’s life. He normally says that he is aware of these things because they are usually taught at the clinic. He denies that he is not sexually active but I can’t trust that because am not always with him. As we speak now there is a party going on in my house so I can’t tell what will happen. He feels that he is now grown up and can take care of himself.

P6 On that issue of disclosure I think I really need help because my child is eleven years old and I have tried disclosing to him and he doesn’t really understand. When I come to the clinic, they insist that I should be then one to disclose to him.

P2 My sister you should understand that children are different. So, just take your time and he will understand though slowly. Do not be in a hurry trying to disclose to him till when he will have that understanding. As the child grows, he will come to understand.

P4 Nowadays is when they insist that you should disclose as soon as possible. Long ago, it could take time. We started taking ARVs in 2010 and at that time my child was 6 years old. I disclosed later when he had joined High School. You know that there are stages that these children go through. You may find that I can explain something to her but she doesn’t understand. But if someone else does then she will definitely understand. We should be able to tell them about HIV but in bits. Let them understand. We should ensure that we tell these children the truth rather than they find out from other sources.

P1 At the same time we are requesting you that are our providers you should not get angry with parents who have not disclosed to their children who are already in standard five. It is like you now blame the parents for not disclosing. This should be looked at because it can make someone stop coming for clinic at this facility.

M Great. A part from the challenges you have mentioned, do you think we have other challenges the young adults and adolescents go through?

P4 Language. There is language that the service providers normally use that may not be good to the youth. Like a provider telling a pregnant youth “the reason she started eating adult food”. This is being judgmental without even wanting to know how this youth got pregnant. You don’t even know if this youth was raped and by saying that you will open the wounds again.

P3 Another thing is that when they get into a relationship then if the partner insists on going for testing it becomes a challenge. There are relationships that usually come to an end after the testing. This is because one of them turned reactive. So, the youth becomes demoralized to the extent that that they would not wish to get to another relationship.

M How do these challenges affect young people’s (i) educational and professional achievements; (ii) socio-economic advancements?

P2 Just like we had said in a situation where the child doesn’t take the medication well because of fear. There is also discrimination where other parents would even go ahead and tell their children not to interact with others who are HIV positive. So, this will mean that these children cannot share anything in terms of education. This can lead even to psychological torture.

M Mmmmm P5?

P5 As for me am the first born and my sister who is reactive is the third born. She got positive through rape. There is a point she got tired of taking medication and started asking why others were not taking medication except her. I encouraged her to take her medication by pretending that I was also taking septrin. This helped her a lot and she finally accepted her status. She wanted to take a course in hospitality but changed her mind to take a medical course.

P4 You get that when they stop taking their medication they are likely to be weak and hence this can lead one repeating the classes. When this happens, they tend to have no one to socialize with because these are all new.

P1 Mmmmh another thing could be the drugs that they are taking which may make them sleepy in class. Especially the medication that is taken in the evening it usually really affects them during night preps. This affects their grade. I had once received a complain that my son could sleep a lot but I told them it’s because of medication.

M How else have these challenges affected their advancements?

P4 I think we have said it all.

M Okay. How have young people tried to deal with these challenges?

P2 For those that are in day school and in primary school that come to the clinic and interact with the others then they are usually encouraged with each other’s stories. The providers here are equally supportive. When they interact in camps, where they normally share experiences.

P1 They usually go through their problems because when they go to the hospital they find people of their age set so they are not afraid to come to the clinic. So, they feel that they are equally important. The retreats and meetings that are held here are very important.

M To what extent have young people succeeded in dealing with these challenges?

P4 There are those ones who have succeeded in dealing with the challenges. Let me talk about Tina and the issue of wrist watches. Tina told her story from the time she was ten years when she was under the care of her grandmother until now, she is an inspiration to many.

P3 Most of the adolescent have seen those that have lived and are doing great so they are an inspiration.

M What support is available for young people to help deal with these challenges?

P4 There is counselling support, motivational speakers, games, support groups.

All As parents we are also a support.

M What do you understand by the term ‘sexual and reproductive health’?

*(Probe responses as necessary: access to reproductive information, safe sex, contraceptive use, STIs and prevention)*

P1 I think it’s the age where one is sexually active and can get pregnant.

P4 It’s a transition period that’s between ten to twenty-four years where the government allows one to access information on sexual and reproductive health.

P3 Its those people who have become sexually active. It’s the time when the youth identify themselves.

M Great. Sexual and reproductive health encompases all that you have said. In addition, it entails access to sexual and reproductive health information, family planning, safe sex, condom use, HPV vaccine, STI/STD prevention.

How important is young people’s access to sexual and reproductive health services such as contraception, STI prevention e.g. condoms, Prep, HPV vaccine?

P3 Like family planning it will help to reduce the rate of abortion. The use of condoms also prevents sexually transmitted diseases.

P1 As of me at that age I won’t encourage the use of family planning. If for example I put a girl on family planning to prevent pregnancy then she won’t know that there are also other diseases that she can get that are sexually transmitted. I support the use of condoms but not family planning. There are children who have blamed their parents for starting them on family planning when they are young because when they get married, they have a problem with child bearing.

P6 Even me I don’t think family planning is good because it should only be encouraged to those that have already sired children.

P3 These are adolescents who you might not know whether they have started using family planning, the best thing is to give them information and leave them to make decisions. You can’t trust when they tell you that they are not on any family planning.

P4 I want to make some clarification. There is one of us who has said that family planning can make someone not to sire children in future. You should know that our bodies are different. These family planning majorly deals with hormones. Being an ambassador of family planning I will tell you that the best we can do to the youths is to give information and let the youth make the decision on family planning. But don’t tell them not to use family planning. If you tell them so then they are likely to engage in methods that will affect them. On this family planning methods, they have both advantages and disadvantages. So, for this method known as DEPO we will encourage that its used mainly on those that have given birth because it has a high percentage of hormones. For the underage they can use coil because it doesn’t have any side effects. You should also know that condom is part of family planning. When you say that you don’t want family planning then you are also knocking out condoms.

P1 I support condom use and I stand firm on that.

P4 For the boys they can only acquire Sexually transmitted diseases but for the girl she will get both the STI and pregnanacy.so the important thing here is that you should give information. Because with time they will open up and ask you which family to use if they are sexually active

P1 On one side what you have said can be helpful but am convinced that these children can be talked to. But for me am not part of family planning. Once a child is on family planning then it becomes very hard to deal with them. This because they can get into nasty things without informing you. So, maybe if I take them to family planning then am only preventing one thing but what about the other diseases? I can’t advise that the youths be put on family planning.

P4 I have a question. Suppose your child tells you that she has a partner then enquires from you to advise on the best family planning method. Can you deny them the information?

P2 You cannot deny such a kid the information the best one can do is to give information then t he guidance.

M Where do young people currently access information on sexual and reproductive health?

P4 Social media

P6 Schools

P4 Friends, google

M Where do young people currently access services on sexual and reproductive health?

P4 At the facilities.

P3 Chemists

P4 From their buddies

M What is the ideal place/location where sexual and reproductive health services easily and conveniently be access by young people?

P4 From the facility because it is where they will receive all the information and the right commodities.

M What challenges do young people face in trying to access sexual and reproductive health information and services?

P4 There is the issue of consent. This is in where an adolescent who is not yet eighteen years goes to seek for these services and the provider seeks to get the consent from the parents first but at the same time the adolescent does not wish the parents be told.

P3 Stigma because one might feel that others might wrongfully judge them for accessing such services.

P4 Confidentiality. For example if I have to access these services where my mother’s friend is the one issuing these services then there are higher chances that she will share with my mother.

There is also a challenge on language because the youth might want a certain family planning method but the healthcare provider might not be able to understand.

There also another challenge which is disability because there are others who might be deaf and damn hence communication becomes a problem.

M How does being in schools or colleges affect adolescents and young adults’ access to sexual and reproductive health services?

P4 I’ve never heard of a school that give students a break so that they can go and access sexual and reproductive health services.

M How comfortable will young people be discussing their sexual and reproductive health concerns with a lay female healthcare provider of their mothers age?

P4 It’s not easy but that person must have a youth friendly mind.

P1 It’s not easy unless you bring your mind to their level.

P2 I never used to like Facebook but I told my daughter to help me join so that we are together with them on this platform. From this then we can come up with a discussion.

P1 As parents it is always good that we reduce to the level of the youth so that we can have a good discussion and they are able to open up.

M What issues would young people be comfortable discussing a lay female healthcare provider of their mothers age?

P1 They would be comfortable discussing about relationship.

P4 Changes that they have identified in their bodies.

P3 Methods of family planning.

P4 There are those who need information on safe abortion.

P4 Knowing their safe days.

M Where and why would young people want those discussions on sexual and reproductive health to take place?

P1 Health facility because they will be free and can ask questions.

P3 They believe that the health workers have the right information.

M When do young people talk about sex with their friends? Do the friends they talk about sex with tend to be of their own gender or different?

P4 It depends with a child. There are those like my son who at class seven had five girlfriends.

M How do young people talk about sex? (Probe: as joke, show off, serious, giggle, give advice, swap information)

P5 It’s like a joke but ends up being serious. This is because there those that are already mature while others are not. Those that are mature always take advantage.

M When do young people have their first sex? Where/under what circumstances does this first sex happen?

P2 It depends with maturity as previously said. Another problem that I would like to talk about is on how we live with our children. Some people stay in houses that are a single room. Like recently my house help found very young children kissing. This might be from they had seen from the parents. You find children mentioning their mates that they did “bad manners”. I always ask them why they term it as bad manners.

P4 The first sex usually happens at parties under the influence of alcohol.

P4 Experiment and exploration.

M When do young people have their first sex? Where/under what circumstances does this first sex happen?

P4 There those that pressure pushes them especially child led families. This is because the parents are not there and they have to provide the basic needs.

M Where do you think this pressure for young people to have sex comes from?

P3 From peers

M How do people react if a young woman becomes pregnant/a young man becomes a father?

P3 For the boy there is no evidence. But mentally the boy has to be disturbed.

P1 They will say that a child has given birth to a child.

M What does safe sex mean to young people?

P5 Safe sex to them means using protection while having sex.

P3 To them safe sex means that they cannot be pregnant.

M P2?

P2 They fear being pregnant. So, when they have sex and go through their menses then it means that they are safe.

M P1 you wanted to say something

P1 Just what they have said……..Adolescents are more worried about becoming pregnant as opposed to contacting the sexual transmitted diseases. So, safe sex to them simply means not becoming pregnant.

M Okay. How do young people ensure safe sex?

P2 It depends on the circumstance that has led them to have sex for example if they are drunk and didn’t plan for it then they don’t use protection. So, the only thing they will do is to ensure that they take pills to prevent pregnancy.

M Mmmmm P1?

P1 (Shaking her head) nothing…..

(Laughter form the whole team)

M What challenges do young people face when trying to promote safe sex?

P4 Abuse from the boys who tells them that they don’t know the sweetness of unprotected sex.

P3 Rejection

M Mmmmmm

P3 There those whose partners insist on unprotected sex claiming that it is sweeter yet the adolescent knows the importance of using protection. It becomes difficult for her to negotiate for safe sex.

P6 Peer pressure from friends who sometimes share their experience with each other on how they felt while having unprotected sex. So, the adolescent would also like to experience the same and in the process become pregnant or contact sexually transmitted diseases.

M How would young people feel if they are assigned a lay healthcare provider as a confidant and source of information for their sexual and reproductive health concerns?

P1 Is it you to assign or they choose?

M They will be assigned at the facility.

P1 They will feel good because they will be sharing one on one and so they will be comfortable.

P4 I can say that I have referrals from my niece who are youths of the same age group. We normally talk and they share very sensitive information with me. We continue with the conversations even on WhatsApp and they get helped.

M Great.

P2 I think it will depend on the person undertaking the task. So, once they are in the facility then they will believe that this person is informed and can help them.

M What would be the attributes/characteristics of such lay healthcare providers who act as sources of information on sexual and reproductive health?

P4 Should be all round and ever available.

P5 Jovial and very free.

P1 Should be easy to contact even when the youth is away. He/she should give out their phone number so that AYA can get in touch freely.

P3 Flexible

P5 There is a youth at this center known as William he’s really loved with the adolescents because of his positive and caring attitude.

M Good to know. What would young people feel about receiving their ARVs together with their sexual and reproductive services?

P4 They will be happy

P3 (Interrupts P4) They will be very happy because once someone comes from the doctors room to room 4, people will definitely know that you are going for planning services. When all the services are provided in one room there is privacy.

M What would be the advantages and disadvantages of receiving ARVs and sexual and reproductive health services together at the same point?

P4 Time

P3 Confidentiality.

P6 Privacy

P4 convenience.

M Any disadvantages?

P4 It will increase work load on the service provider

P4 some providers may not be knowledgeable in both the services. It will therefore be a challenge for this provider situated in the specific room for adolescents

M Mmmmm. What do you think can be done to these adolescents to increase their access to sexual and reproductive health services?

P4 Have one stop shop

P1 Counseling

P4 The one stop shop will also offer counseling services

M Great. Any other interventions to improve adolescents’ access to sexual and reproductive health?

Silence…..

M Thank you very much for your participation. Any questions?

P6 What is the best time for an adolescent who is HIV positive to disclose to a partner. Is it after they have gone for testing?

P4 The best is not to get to the VCT cause the relationship might end after the testing. The best way is to pose leading questions.

(Members discussion on disclosure amongst themselves)

P2 We are grateful for adolescent centers like this one where they can get information on what to do. They can also consult the parents.

M Thank you so much for your time.

**STUDY: AYA**

**PARTICIPANT FGD2**

**Date: 17-MAY-19**

**Venue: LUMUMBA HEALTH CENTRE**

**PARTICIPANT CARE GIVERS**

M My name is Irene Okumu.I work for KEMRI.Today we are meeting so as to have a discussion the way we had agreed earlier. I’m grateful you have already helped me with signing the consent form for the recording and taking part in the study. With me is Sharon Okaka who is going to help me with taking notes on your responses. So I will be helping you in doing moderation and giving you questions while you will will be giving responses and in any case you have not understood anything you are free to ask. If you have not got it, I will repeat. In case you want me to ask in a language you understand you can say. Ahh whatever we will discuss will remain here there is no person who will get to hear them, see them apart from my colleagues who we are together with in research, after some time we will destroy all that we would have recorded in the recorder. In that case all the names whether somebody mention you or you mention someone will not be used by anyone. Have we agreed? You be free and anything you want to say you can say it here so that it will help us to help our children that we wanted to help in sexual and reproductive health. ok

Amm. So we will start. These our children they normally have challenges here and there. On the challenges that the children normally have. The first that I would like to ask is that challenges do they have?

P In which occasions?

M in general children usually have challenges

P6 Of which ages?

M Ammm starting from age…. all children especially those between 3-14yrs.Soo the children that you normally have you get that a child has challenges. Which challenges do they normally have?

P2 Disclosure

M They don’t have disclosure? Mhhhh

P6 You see these children are getting to those ages that they are now big people .They have eyes that can now admire girls. They want to talk to girls and seduce them. They don’t have means to do that and at the same time these girls are refusing. Like my son I have seen him, whenever he sees a woman he starts salivating.{Laughter}And he is still young. And even my girls are telling him that if you are found with someone’s daughter you will be arrested. You will be taken to the chief. But of late this has reduced. Whenever he sees a girl he likes laughing with them. So I see that he has started being a grown-up. At times I tell him not to do somethings. I tell him to complete school and get a wife of his own. I tell him that if you touch somebody’s daughter you will be taken to jail for seven years. He normally replies that he doesn’t want anything like this to happen to him.

M mmmh Another person?

P1 Nowadays you can get a five-year-old who knows everything and they are even trying this on themselves. So this is usually when a mother does not take care of herself well. When the children go to school there are kids whose mind has been corrupted. You find that there are mothers who engage on that act when the lights are on and the children are there and when they see this the next day when they go to school they start teaching others. Now you find that you can imagine that your child is young and so you say that you will start talking to her once she reaches 10yrs but at five years she already knows everything and is also engaging in it.I normally see my neighbor who does the work of this body part going down[LAUGHTER] and has a child of five years who know everything. So when this child goes to school she puts the other children in a group and start teaching them. wawawawa! So you are sited knowing that your child does not know anything but actually she knows more than you. If you want to know that your child knows these things you should be close to her and talk to her. Whatever she tells you will shock you because you always know that she doesn’t know anything. And then again another challenge is the television where there are programs.

P6 some behaviors they get from television.

P1 You get that a child knows everything. When these people are doing this thing they might think that this child is asleep but.

P6 They can switch on the television when you are not around.

P1 and they watch it.

M Any other challenge?

P4 My colleagues have mentioned quite a number. Another challenge is through social media especially when we leave our children to be exposed to social media. They know and do a lot of things on the phone of which parents do not know. And again children at adolescent are selective on some issues. They are selective on food, dressing and even at times selective on the groups of people they walk with. EEhhh these kind of challenges are also with the adolescent.

M Yes, ok do we have another?

P5 Me what I can say that these children are not open with their parents. They normally do their things in secret.

M And these children these challenges that they have through education and even work place how does this affect them? You might get a young adult who is between twenty to twenty-two years who is working. If maybe you don’t have a child in this category maybe you can talk about the once that are going to school.

P2 You normally get those in upper classes where it forces that teacher to share with you their character that they have in school and you have to accept because the teacher is your eye.

M Is there anything that you would wish to add in terms of education?

P4 In terms of education when a child reaches adolescent and has been affected with social media with the things they see especially when they have started knowing about relationships maybe she has got a boyfriend who writes her messages he writes him a letter like our time when we used to be written to letters. But nowadays there are phones that even a child can ask a neighbor to let them use their phone. So when this child goes to class she cannot concentrate because she still thinking of the movie that she watched. Or the message from the boyfriend indicating that they are going to meet on some particular day. This is stuck on their heads and she is anxious for that day to reach so that they can meet. She wishes that day to really come to pass. This makes them not to concentrate on their studies but rather concentrates on other things.

M: And these challenges that they have is there a way that they deal with them?

P4 With me the ways in which they deal with these challenges social media it’s still with us parents. If you have a television which programs do you watch? You have to talk to your child and tell them that these programs will affect their brains because that is what will still affect them. So instead of watching these movies will affect your education maybe they should read storybooks that have lessons of somebody who struggled but finally suceded. You encourage them. On the issue of boyfriend girlfriend, it’s not bad but a time for this is coming. It’s not a bad thing but tell them that once they are impregnated they are the ones to loose. You will be at home for nine months after which you stop going to school because you are a mother. Maybe now you will be taken to school but once the children know that you had given birth. So your advice and then see how they react to see whether they angry or happy. If it fails, you can call someone to talk to them.

M Mmmm any other way?

P1 Me to add on that you see a child who is an adolescent is really hipper so what we parents can do is to first you put her in church so that she knows the teaching while praying to God to help you in a way to talk to this child because you know that they are hipper and if it’s a boy you see them walking like this[demonstrates with hands stretched on the side ]like this

P6 Even me am seeing mine.

P1 So what you will do is that you become close to the child so when he is high you calm down and look for something that they really like a lot and when you get this you use it to lure them. Then you talk to them so that you agree. If you can’t agree then you cancel this. Then you start again and you will slowly do this until you reach a point that they will listen to you. I used to see with my brothers child who started that thing at nine years this is because of the surrounding we stay in. So she started and after this the mother could not handle her. The father really beat her but there was no change so I decided to take that child and I looked at a way to talk to her and bring her close and she told me everything. Then I told her the goodness and badness of that thing. You know you can beat up a child without giving her a reason. You even tell them not to walk with specific people. So I told her the problems you will get are two you will be infected and also get pregnant. If you become pregnant you will not continue with your education. So you will choose what you want because I have told you both sides. She told me that the parents were beating her telling her that a certain person was bad.so as women we should always observe our children. You should know how they were the previous year and this year.so you put them close and show them love

M Ahhh thank you so much and I can see that whatever you’ve talked of has covered my number two and three questions and how you help these adolescent deal with challenges they have and the support you give them. You had touched on the way you support them on religion. And this issue of sexual and reproductive health how do you understand? Like access to information. Where do you get this information? Issues of sex where do you get them? Where do you get them as parents?

P1 Please elaborate I have not understood.

P4 she’s speaking of something I understand

M What do you understand by the term sexual and reproductive health?

P4 Ok me what I understand is that when I child stars growing there are changes that occur in the body especially when the girls start their menses there are others who will be shocked. When a girl starts experiencing menses there is a risk of pregnancy. She will develop breasts .so you as a parent you must explain this to her because mainly you will buy for her sanitary towels. If you don’t buy her sanitary towels another man will do this. He will buy for her and can even impregnate her. You tell her that she’s no longer a child but transitioning to adulthood. If you see these thing when you meet a boy or any man sexually then you will be pregnant. These are changes that are in your body so you will be pregnant. And you cannot only be pregnant but can also get diseases and then you explain to her the kind of diseases that he can get. You tell them you went through the same changes and what you did. Just tell her that you were patient until you gave birth to them. There are others who are sexually active and you can talk to them. For those that are sexually active you can talk to them to use protection. There are men who can lie to be using protection but tamper with it and spread infection. You tell them the changes. I usually tell my child who is sixteen years and she is the last born.

P1 To add onto that point when a child reaches adolescent we as parents should be very friendly with them.at times you just take your time and explain to them everything. Don’t hide anything mmmhh. This is helpful and it has helped me. This helped me with my daughter. When I child has dressed kindly tell her that she is smart. If you don’t she will hear it from someone else. If you tell her that she is smart she will get used to it.

P4 she knows that she is smart.

P1 so when we sit with our children who are of this age then we are supposed to be their friends and close to them. Maybe if they go outside and sleep when they come back you don’t talk to them but beat them. This way you are not helping them. So you become friends with them and they won’t hide anything from you.

M In relation to contraceptive and family planning do you talk to them.

P1 I talked to my child and she asked me whether it’s used even if someone doesn’t have a boyfriend. [Laughter]I told her that this can prevent pregnancy but not other infections. She told that she’s going to take care of herself until she gets a husband.

M MMmmh ok

P6 You see starting family planning with girls who have not given birth interferes with the uterus as a result they might not sire children.it only good with us who have two, three or four children. But not these girls. The only advice we give them is to protect themselves. When that time comes they can go to the husband instead of having sex with many men.

M And what about PREP? In adolescents. Can you talk to them about Prep too? Does it have benefit on them?

P4 Mine I had already told her about the family planning and the effects. I told that she knows every side effect and if doesn’t want it then she should take care of herself. So the decision lies with her. When she wants to do anything she should think of the benefits. You have to explain to a child the benefits of something then they can make a choice. You cannot take a child by force to family planning.

M what about HPV vaccine this one that is used for cervical cancer on our ladies? Have you heard about it? There is that vaccination known as HPV.And that one can it help our children?

P2 What I see is that if we start with them early then it can really be helpful.

M And what about if they receive this HPV vaccine?

P4 I see that its ok because children can decide to do what you tell them or not.so you just tell them so that they know so in case they get into an act they should not be infected. These condoms have heard they are not very good. Someone can put them on not in a proper way hence leads to infection. That friction can also bring infection to the vagina.

M What about information and access to services on reproductive health where do they get them apart from you parents the way you have said?

P2 Hospital

P4 Friends

P5 schools

P4 social media

P4 some practice by telling themselves to just start.

P2 Mine was like that she stopped taking medicine the way she had heard that it will make her die. This made her to be put on second line treatment.

P6 How did she come to be?

P2 she is okay

P2 She was doing it secretly. I didn’t know. I give her the medication and she does not take it.

M And these services a good location where we can get them as you had earlier said school,

P2: Hospital

M Hospital is a good location. Any other suppose the hospital is far

P6 Churches

M Which other can we add cause we have hospital, churches and in the house what else can you add

P4 We also have cancelors.

M Where do we find these cancelors? [LAUGHTER]

P3 Some children go to DREAM GIRL where they are taught.

M What is DREAM GIRL?

P3 Organization

M: Ahhhh to what extend do these children get sexual reproductive health services and does it help them?

P4 Ok a child will start knowing and understanding themselves when they start being taught since some will be taught and start menstruating and when this time comes. They are even taught the signs of pregnancy, through this when they don’t see their menses then they will know that they have messed up. They will know the changes they expect from their body.

M These our children usually experience challenges in access of sexual and reproductive health services. What are these challenges?

P2 Being ashamed.

M Any other person?

P4 Kindly repeat that question

M children can have access to sexual and reproductive health but you find that they don’t go to get them. What can hinder them? Someone had mentioned on shame any other?

P4 At times even parents.

M Parents in which way?

P4 Maybe a child wants to go somewhere to get these services but you give them aa lot of work.

P6 so they escape to go. Even my daughter ran away and slept outside. She came back took some water and went to bath. I really got mad and even didn’t want to speak to her but she was still laughing with me .Then my youngest daughter told me that I should relax. I warned her that should she be infected or get pregnant she would have to go to that husband. I told her that I don’t like her and despised her like rubbish. I told her that I was really struggling with my leg which was broken now for the second time. I told her to keep on walking around outside there but not to bring a child to me but rather take the child to her husband. “That body size you have is really deceiving you”. When I talk to her like that she really calmed down.

M These our children some are in colleges, secondary or even primary if they want services on sexual and reproductive health is there a way they can get them? Those that are in school.

P2 Through teachers.

M Through teachers mmmmhhh

P2 church

P4 There are others who have chapels. But this is still school

M IF they are in school they normally get it from school and if they are not in school what challenges can they get when they are trying to access it.?

P2 So this they can get during half term and when school has closed?

M And these children can they accept to be with a person same age as their mother to talk to them on issues of sexual and reproductive health?

P5 They can really be free with them more than even the mother.

M So they can openly and freely talk to them? If somebody like you talk to them. These adolescents.

P4 Maybe those that maybe are of the same age as you and maybe you are in the same church group.

P6 EEhhh the people at the church can talk to them

P2 They will be open with them.

P6 They can even talk to our friends. This I think they really love,

M What do you think they can discuss with this woman?

P6 The woman will tell them that they are children who have reached a certain age so don’t engage in bad behavior. I want you to be good children who behave well.

M And these children what can they talk about?

P2 They talk about the problems they are undergoing. They can say what the parents are doing that they don’t like.

P1 To add on this we as parent at times we mislead our children. I can misbehave and when I try to correct my child when she does the same thing it’s not possible since they had seen what we did. So we should examine ourselves. Like me I lost my husband in 2008.I cannot be introducing to my children to different uncles. When we behave well our children will always listen when we talk to them.

M why and where would the adolescent be willing to discuss issues of sexual and reproductive health?

P2: With themselves or?

P4: When they are with their peers.

P2 Even at home when they are with their peers.

P4 when one of their age mates starts talking they contribute

M ahh when they discuss issues of sex do they normally talk with those of the same gender or different.?

P1 You see when children are playing they discuss this usually when they pretend to be their parents, they therefore portray the real character of their parents.

M When they are discussing issues of sex do they tell it as stories, as a joke or show off?

P1:P2P5P6 like a joke

P1 At times with the children the one teaching them who knows says it like a joke but the rest being taught normally take it serious.

P4 some talk about it from experience by exchanging information,

M And these our children when do they have their first sex and what drives them to it?

P : There is no specific age because you might assume that your child is young but that’s not the case. In the past years it was easy to know,

P6 Like my child after finishing form four I began to hear that she had a boyfriend. I really chased the boy away. I am a mad woman.

P4 If I contribute. Children know about sex at about five years. It’s not only by doing the act but knowing that male and female have different body parts. Thats why you will never find a girl saying that she is a father, you even find a child playing with a doll and even pretending to be breastfeeding it.

P1 And even nowadays you normally find that even a two-year-old you cannot lie to them that newborn babies are bought. In the past our parents could lie to us that they bought babies.

P4 Nowadays you cannot lie to them when you are pregnant that you are full from eating.

P6 So we should tell children freely that the newborn is their child and if they engage in sexual act they might get children before the right time.

M So these children are not always willing to have sex but what drives then in it?

P4 Peer influence.

P6 From their friends who can always keep their secrets.

M Apart from the two that you have mentioned which other?

P1 From the family

P2 Environment

P4 Lifestyle

M Any other?

M How do people react if a young woman becomes pregnant or a young man becomes a father?

P6 There are actions that are taken. The villagers can really talk about it, if the family of the girls reports then they can be compensated or the boy can be arrested. When they are still young giving birth might be a problem.

P4 Let me add something you know that when a child gets pregnant at an early age they can be stigmatized.and can be discriminated. Those of the same age when they see them they can laugh or talk behind their back.

M Any other?

P6 We as parents we should teach our children. We should guide them in terms of marriage and education.

M What does safe sex mean to young people?

P2 Use of condoms

P4 Abstaining

P6 Use of condom

M Any other apart from the two?

P4 For example, when they reach twenty-five years and now want to marry each other. They are still our children, We advise them to go for testing so that they know their status first before engaging in sex. This should be done after every recommended interval. This will ensure that the pregnancy may be safe.

P6 But nowadays they are very fast in engaging in sex before even testing. The boys just do what they want to do and run away. Those who are calm will live long unlike those that are not.

What challenges do young people face when trying to promote safe sex?

P2 You get that they think deeply before starting this conversation They first look for people who are close to them.

P4 Ok there are others who can be cheated by being told that one cannot eat sweet with the wrappings.so they are told that the wrappings have to be removed for the sweetness to be felt.

M So is this a challenge to the one being taught or the one teaching?

P2 The one being taught. So they are therefore being given wrong advice. There are other children who are clever but usually mislead others.

P6 Mine has been reported.

P4 there others who will despise the right advice simply because they are of the same age because they see them like they don’t have experience.so they want someone who is older than them for advice.

P1 To add on that you will get that the one who is advising may not have the real experience to the extend where they will be asked if they have ever done it.

P4 it’s normally said that practice makes perfect.

P1 So when they are told this they will fear to another place to give advice.

M How would young people feel if they are assigned a lay healthcare provider as a confidant and source of information on their sexual and reproductive health?

P2: this will be based on the teachings.

M [repeats question]

P1 This is usually still pegged on the parent. The way you have raised up your child will make them to be able to sit down talk and listen to another woman. And also you as a parent if you have never sat down with your child to talk to them then it will be a challenge.

P4 As a parent you should also talk to the lay healthcare provider about your child. The provider should also know the character of the parent to ensure that the provider gives the right advise. Then the provider should be someone who is able to keep information private and confidential so as to earn the trust of the youth.

P1 You see as a parent there things that we normally do and the children just watch us and when it reaches a time we want to talk to them they refuse. We should really focus on how we live with our children, we should set a good example and direct them to church. So as they attend Sunday school and even youth groups they are taught.so when such a child loses focus its very easy to bring them back on track.so such a child even when you will find a person to talk to them it will be very easy.so as a parent you should the children to the right direction.

M {elaborates further on the question}

ALL That is ok

P6 The parents should be the first to talk to the child since you are the one who sired the child. Since you are the one who breastfed the child advise them to do right things.

What would be the attributes of such lay healthcare provider who act as sources of information on sexual and reproductive health?

P1 This woman who will talk to the child?

P2 Christian

P4 secretive

P5 The way she presents herself

P1 should be someone with wisdom. And should also know how to approach and get information from a child.

P4 And then again the provider should be able to learn the moods of the child so as to see whether they will b able to get information. If the child is not willing to talk the they should postpone the talk.

P1 [Laughing]Let me tell you a story of a friend of mine who was staying with a brother in law who was sexually abusing their child who was in nursery school. Whenever I would go their place I noticed that whenever the child saw the uncle fear always engulfed her. I asked the mother who said that everything was okay. I took the child bought her chips and soda and asked her to draw the things she likes and the once she doesn’t like. After several attempts the child opened up and shared with me what the uncle normally does. I asked her whether she had shared with the mother and she said that she hadn’t because the uncle had threatened to kill her if she does so. So as parents we should examine our children’s behavior.

P4 Even my child I told her that if somebody touches their private parts she should tell me.

M What would young people feel about receiving their ARV together with their sexual and reproductive services?

P1 That will be good. This because it will teach them how to take care of their lives.

P4 I see it to be good because adolescent usually needs privacy. They usually don’t like things that are done openly.so when it’s done in a closed room it makes them comfortable. This will make them open up unlike in a group. This will help them express themselves better.

P2 I also see that a closed room is good. if one is open enough they can ask or be asked questions.

M {Elaborates on the question further}

P6 Those that are below fifteen years should be taught what to do and what not to.

P1 In my opinion children should be taught in a group. This is because different views are shared and this one is likely to help one who was really down.

P4 another thing that I see in these adolescents is that their senses are very active in terms of smell, touch, they can always organize for a projector and show them a video on challenges that other people pass through and how they did overcome and the ask questions after that. Through that they would have learnt a lot.

P1 it’s also good to organize girl to girl talk and boy to boy talk. This is because when they are put together some may fear talking because they can be shy.so with time they can be put in one group.

M ((Summarizes what has been said)

M what would be the advantages and disadvantages of receiving ARVS and sexual and reproductive health services together at the same point?

P2 this will help them save on time especially if they are going back to school.

P5 she will have courage because there no person there and the services are done collectively

P4 if the children go inside one by one they will fear because they are alone unlike when they are in a group. The children will not know the challenges the others in the same group are facing.

P1 Again you can get that when its somebody of the opposite sex and they are alone then the doctor will be talking to them yes but the mind will be off.

P4 there should be an arrangement where the provider sees the children of the same gender as the providers. Or also there should be both male and female providers in the room at the same time.

P6 If there is only one provider then the girl may shy off from talking to the male provider. She will not open up.

M Thank you so much for your time that has enabled us to have a discussion on ways that we can help our children. We have come to the end of the discussion. The time now is 1325hrs.

**STUDY: AYA**

**PARTICIPANT FGD2**

**Date: 15-MAY-19**

**Venue: LUMUMBA HEALTH CENTRE**

**PARTICIPANT HEALTHCARE PROVIDERS**

M Good afternoon, everyone? As I said earlier my name is XXXX and our note taker is XXXX. She also works for kemri. Today we shall be having a focus group discussion on adolescent.

What are the challenges that young people face today?

P4 stigma

M Any other?

P1 Age barrier

P2 Peer pressure

P1 Fear of the unknown

M These challenges how do they affect the young people in terms of educational and professional achievements and socio-economic advancements?

P1 When the young adults come to the clinic they always have that when they go there they always have that mentality that they will find someone who is the age of my mother and she will be judgmental. they might be suffering and need cervical cancer screening but when they find an older person they will not open up.so if they go there and find someone who is older then they go back to their closure.so this is a challenge to achieve sexual and reproductive issues on young adults.

P5 so the part of discrimination it affects their self-esteem. You find that when one’s self esteem goes down it becomes a challenge.

P1 The young ones they rarely think of socio-economic activities. They are geared towards social media and so they focus on buying an android phone, dressing well. They don’t think in line of empowering themselves economically.

M How have young people tried to deal with these challenges?

P1 Some have been dealt with partners like having friendly spaces for the adolescence where they can talk about sexual and reproductive health peer to peer .And again we have integrated services there is comprehensive package like care and treatment aspect, screening and treatment itself and the commodities themselves.

P3 Okay so once we have identified the challenges we take them through health education and we tell them how stigma and discrimination affects them socially .so ideally we teach them how these challenges affect them and how they should deal with i.e.we want them to make their way out. We don’t make decisions for them.so we do a one on one discussion and by the end of it we must ensure that they themselves come out with solutions to address the challenge

M To what extend have people succeeded in dealing with these challenges?

P6 as a healthcare provider (talks while looking down) normally for example when they come here for care and treatment they normally tell you what they are undergoing.so as a healthcare provider you do counselling based on the problem.so after the counselling you look and see if they are satisfied or not and then you find what next step to take.

P2 The adolescent normally comes in with different challenges but the healthcare workers try to investigate and find out if they can share the problems. It’s a challenge but we try as much as possible to make them tell us their challenges.

P3 For us healthcare providers we have created support groups for them so that they can share their ideas and then because when another person who has the same problem share it’s very easy for them to adapt than the healthcare provider as per what the healthcare to do.

M What support is available for young people to deal with these challenges?

P3 Yeah from the support groups those discussions there are challenges that crop up. Those that cannot be dealt with here are referred to places they can be helped.

P5 In addition to that we have also created WhatsApp group for those that have smartphone for us to share, it’s a bit favorable because they discuss the problem on their own and come up with solutions. so your part is just to add a little bit.

M What do you understand by the term sexual and reproductive health? (p6 walks out) (p7 walks in)

P1 What I can say on sexual and reproductive health. the sexual part is the feminine or the male .it involves the genitals from the reproductive is vagina, fallopian tube, uterus. All those .

P3 Majorly it talks about the male and female reproductive system. Again for the sexual part how do we deal with sexual issues like how do we come up with facts like safe sex in the context of HIV how do we come up with policies that will reduce transmission of HIV.Actually when you have talked of the reproductive system then you tell them what is normal in a woman and what is abnormal and what is normal and abnormal to a female so when there is an abnormality they can know.

M How important is young people’s access to sexual and reproductive health services such as contraception, STI prevention?

P3 All these are important because when we are preventing STI which in future can cause infertility. So when we offer these condoms because they are vulnerable so we want to prevent these young ones who are not infected from being infected.

P1 Maybe through that they get enlightened and educated that if they have unprotected sex they are prone to get STI and unplanned pregnancies.

P3 that is important to prevent these young adults from infection that are caused by HPV.

M Where do young people currently access information on sexual and reproductive health?

P1 We have google

P3 in school, peer and from parents. There are parents who are open enough talk about sexual and reproductive health. And some on the magazines.

P1 some just walk in the hospitals to get information from the clinicians. In the youth friendly centers.

P3 Now that we have the youth friendly centers even if they go to the hospital they are referred here.

M What is the ideal place/location where sexual and reproductive health services easily and conveniently be access by young people?

P1 Youth friendly center

M To what extent are the sexual and reproductive health services offered to the young people meet their needs and expectations?

P6 it meets their need to satisfaction.

P1 I wish they were here they would have told you that the satisfaction is beyond optimization.

M what challenges do young people face in trying to acess sexual and reproductive health services?

P5 You can find the youth and the mother receiving sexual and reproductive health services in the same facility.so the youth will fear being seen by the mother and what the mother will think of her when she sees her there.so this brings a lot of fear to the young one.

P1 The youths have really enclosed themselves and it takes the time before they realize that they have a problem with the reproductive system so that they can come to the hospital.by the time you see them coming it means that something has really pushed them. Maybe it something beyond them. When they come they start the blame game, they don’t want to own the treatment. They start that it could be their boyfriends, To girls candidiasis is very normal but for the youths it takes the time to seek treatment. They always think everything is sexually transmitted.

P4 There is also the bit of Christian background. The religion that they come from. People have different faith and believes.so some in one way or another affect these services so you find this affect the reproductive health. This Christianity and religion set up they always think that this is the best way to handle this and this is not the way. For such people to convince a young adult to come for this services it’s a little bit tricky.

M How does young people being in schools or colleges affect their access to sexual and reproductive health services?

P7 its true and its possible it can affect let’s take for instance like in the college schedule let’s say a youth who is learning away from the clinic where she normally attends.it becomes impossible for them to honor their scheduled appointments. They may as well fail due to lack of resources like fare.

P1 they may come yes but when we talk of contraceptives. We understand that jadelle is put here (points at the arm) so there are some schools that associate contraceptives with being promiscuous and a bad example.so when the schools are open school the teachers usually check the upper arm.so it’s hard for the girls to have these contraceptive because of the judgmental aspect. Sometimes back we had those that wanted it to be put somewhere else and not the upper arm.

M How comfortable will young people be discussing their sexual and reproductive health concerns with a lay female healthcare provider of their mothers age?

P3 No

P1 they cannot open up.

P7 Mostly the youth at their age. they will always prefer someone of their age. They usually shy away from people who are senior in age. Even if it was not in age but also superiority. They want the provider to go the grassroot so that the can talk the same language.

P3 And also the fear of being judged. This is someone who is like their mother. They may be fourteen and need family planning services. She fears being looked at and imagines that she will be told that at that age what she needs the family planning is for. But if there are in the same age group they will be able to share.

P1 Then there is also the issue of power over. When you meet someone who is the age of your mother then definitely there will be power over you cannot meet at some point to agree.

M what issues would young people be comfortable discussing with alay female healthcare provider?

P1 They would be comfortable discussing almost everything but the aspect of sexual and reproductive health in the relationship then at this point they will have closure.

M where and why would young people want those discussion on sexual and reproductive health take place?

P1 Adolescents what works for them is mainly might be through a text or WhatsApp or just one on one it depends with what is convenient at that point. Like if I have got providers number and probably I got infection we can chat via WhatsApp and even make a call.so this depend on the convenience of the client.

M when do young people talk about safe sex with their friends? Do the friends they talk about sex tend to be of their own gender or different?(p3 walks out)

P1 The adolescents when we are having a discussion tend to say they are not sexually active and view it as bad manners which they cannot engage in until a point where a peer who is a friend reveals this. They will always go round and round to a point where you tell them to give you the contact to that friend so that we can book an appointment.at this point is when they open up and say that its them and not the friend as they were alleging they will tell that they were afraid to that its them since they have already been enlightened in the support group.

P7 it’s also not easy as she is saying they are only comfortable where they are alone like the support group and they can discuss their issues and maybe be able to advise each other. because by the time the youth is coming to a provider with an issue it means that they are really overwhelmed. They normally like to solve their problems at their levels.

M how do young people talk about sex?

P1 it’s always a secret talk. like they don’t want someone to hear the conversation for fear of being judged. they giggle between themselves.

P4 actually it depends on an individual. personally have experience with youth who feel it’s a fun .one tells the other how he has met a lady who is their girlfriend and then ask a fellow how to handle the girlfriend. Basically they open much when they are by themselves. Normally it’s like they are adventuring the sex life. some even show off by telling the others that he has never seen them walk with a lady.

P5 like she has said it’s all about an individual. For you to get information from the adolescent one needs to behave as if they are story telling.

M When do young people have their first sex?

P1 In most cases their sexual involment is not voluntary based. they are ever cohersed. they are not always ready to have sex but circumstances push them to have sex. some just do it for show off because they were previously intimidated by the peers that they have never been seen with a girl.so when the boy and girl meet they always think they have to do something. They were however not ready for this so they cannot access condoms so they just do it.

P4 Social media. They visit each other to watch movies. During that moment they see what happens and begin to practice especially these pornographic movies.

M To what extent do you think that young people are pressured into sex?

P1 It depends on the transition. During transition we have physical, sexual aspect. when these young boys/girls are transitioning from child to adulthood there is a lot of pressure from peer influence or individual based. Sometimes the body might be demanding and when you get in and only realize you did a wrong later. The peer pressure is real especially when the peers keep on discussing their experiences with sex.one feels left out so one will like for sense of belonging.

P6 with me I can say that its mostly through peer pressure. They want to practice what their partners have experienced.

P2 Drugs. Alcohol makes a lot of youth engage in sex. Bang also does this.

M How do people react if a young woman becomes pregnant /a young man becomes a father?

P4 They feel like it is a disgrace. Both feel uncomfortable but the female is more affected.

P5 The female feel more disappointed and to her it’s like the end of the world. And with that they can even start with a way of looking for a way of abortion.

P2 The male mostly they feel shocked and disappointed because they have done something which their parents never did at their time.so they may feel they have wasted their lives.

P1 To add on that today I had an experience with a youth who came for testing. He told me that he had two girlfriends one was away and one was near. When I asked him what he would do if he impregnated one of them he said that he would run away(Laughter) because currently he doesn’t have money. He said that he would look for them latter when he has made it in life.

P7 They don’t want to be involved in shameful things.

P5 At the same time running away from responsibility doesn’t apply to all men.its mainly due to instability in finance.

M What does safe sex mean to young people?

P7 I want to assume that now that we have very few incidences of unplanned pregnancies. I think they really value it even if we say they shy away from approaching a health worker now they are finding their way out to have protected sex.

P1 At some point the youths believe that safe sex is having contraceptives and therefore they won’t have a child. The youths now fear pregnancies and not HIV.As long as they are on contraceptives and cannot be a disgrace to their families they tend to think that nobody can know if they are sexually active. And for HIV they tend to think that they will just take drugs and nobody will know. they shun away from pregnancy like a real plague.

M How do you know people ensure safe sex?

P1 Mostly here at the Youth Friendly Center the youth come for contraceptives but they are never accompanied by their sexual patners.but is hard for them to pass through the HIV and counselling booth.so that one itself speaks for itself. And again being that DEPO (contraceptive)can be bought in pharmacies and be injected there they use is without the knowledge of contraceptives. they go there with a fixed mind of which contraceptive that they want. Postinor-2(contraceptive)is mostly used by the youths because they don’t always carry condoms.so its used mainly to prevent pregnancy.

M What challenges do young people face when trying to promote safe sex?

P7 First challenge can be like we said they fear away because of judgment. they would prefer anything that will show evidence that they are doing it.it has to be a secret as possible.

P5 Another one is that when it comes to patners. there are others who complain that their partners don’t want them to use condoms.

M How would young people feel if they are assigned a lay healthcare provider as a confidant and source of information fortheir sexual and reproductive health?

P1 if it will be a lay health worker or the same peer to peer influence then it will be a noble thing. If there is that power over it will not ogre well with the young ones. They will shun it because they will keep a lot to themselves.

M What would be the attributes/characteristics of such lay healthcare provider who act as source of information on sexual and reproductive health?

P5 one who is friendly

P4 Must be very outgoing

P7 Very understanding. I mean you don’t judge them, you don’t view them like they are from another group and you don’t coherse your will on them. You support their decisions as much as possible.

P1 You give them a win win option. You tell the them that you are there to help each other like that issue affects us. You bring yourself to their understanding to help them think outside the box. Like you are rebranding the problem.

M What would young people feel about receiving their Arvs together with their sexual and reproductive services?

P7 I guess this could be the best for them because this where openness is.as there are receiving their HIV care you are able to explore more and get any other problem they have regarding sexual and reproductive health.at the same time it’s so easy to win their confidence as you speak to them about their health. However, there are others who are specific on the individuals they want to talk to. A youth may come to you to access HIV care but on top of this he/she has another problem on reproductive health and may be choosy on the person to see them. They may consider someone who has been there for a long time and is used to this person and has been sharing for some time with them. If you bring a new person it becomes very hard for a youth to open up.

P4 For me giving the services concurrently is still much better. If the appointment for collection or ARVs is different from that of reproductive health, then you know that not all youths are sexually active so when the dates are different then some are likely to come for the ARVs and not the reproductive health issues.

P2 I think it will be wrong because giving a youth who has HIV tools for sex.so if you give then it will not be well for some and not all.

P1 At times it might act as an eye opener to those that are not sexually active. It may be basic and some of them might say that they want to practice sex. They might look at it as a fun thing.so there will be a provider in the room and the issue of adolescent. if the provider is transferred or is not there then the new provider will have a problem from ice breaking to the point where adolescent opens up.

M What would be the advantages and disadvantages of receiving ARV and sexual and reproductive health services at the same point?

P5 The first advantage would be they will be able to access bot services at the same time.

P1 it’s convenient because it’s a one stop shop where you can get everything

P4 Empowerment for those who know nothing on sexual and reproductive health.

P7 Because of the quality of care you can split the services. If there is something that is related to both then you handle it at the same time.

P7 it takes long for the provider to finish with the client all those services and it may delay the queue .

M Thank you very much for participating in this FGD.

**STUDY: AYA**

**FOCUS GROUP DISCUSSION- HEALTHCARE PROVIDERS**

**DATE: 23-MAY-19**

**VENUE: LUMUMBA HEALTH CENTRE**

**START TIME: 10:59AM**

**END TIME: 12: 47PM**

M Okay. Welcome to today’s Focus group discussion my name is XXXX and our note taker today is XXXX. The time now is 1059hrs and I would like to begin by just asking what are the challenges that young people face today?

P7 In relation to HIV or just general

M General challenges

P7 Young people face a lot of challenges in terms of lack of jobs mmmmhh (silence).

P6 They face challenges for example like mmmhh lack of communication and advice from adults. For example, there are some kids that come from families that there is no adult who can take care of their education and their needs. They lack parents who can give better advice.

M P4

P4 Young people face a lot of challenges one is peer pressure, alcohol abuse and substance use. We also have different sexual orientation and so to crown it all they are having body changes as they grow.

There is also peer pressure that come from the groups that they are involved we can say being idle so they are exposed to lots of vices. So I would say that basically they are trying to accept their body changes and there is a lot of confusion involved in all this.

M (Summarizes points mentioned by p4) P2 kindly tell us how changes in the body is a challenge to the young people

P2 It can be challenging because when there is changes in the body, they are tempted to test what they have like the breast they will like touching. And so, they might even try to experience what others experience like the adults they have sex so when they start having such kind of things then they will love trying how having sex feels. It may be challenging if they do it at the wrong time. And for the ladies they would easily get pregnant or any challenge that are involved in it.

M P4 anything that you can add?

P1 Maybe another challenge that they face is when they are developing changes, they lose their self-esteem especially young girls when they develop breasts. They feel as if that is something abnormal happening to them. The girls when they get their periods, they feel ashamed. Another challenge that young people face is gender-based violence. This is sexual and physical harassment from older people.

M When we talk of gender-based violence and sexual and physical harassment from older people is it a challenge for both adolescent and young adults?

P1 It cuts across both

M P8 anything you need to add?

P8 Maybe I can say it’s the challenge of the know-how and maybe education.

M Please explain

P8 When I talk about the know how generally this is majorly through peer influence when you take for example adolescent in high school they tend to engage in some behaviors or activities which they don’t know the harm which later on or in future or at the moment, so without being informed the adolescents or young people they won’t know what they are engaging in.

M P5

P5 I want to add on what P2 said about physical changes in the body. If I can go back to those olden days where they could say that if you develop those adolescent symptoms it means that you are either sexually active or you have a stunted growth .Nowadays people are like if I don’t see you with “ndevu” (beards ) then you are a young man. Then youths don’t have opportunities in the society cause if you look at our baraza those people in the olden days are still the ones that are there. So, we still work with those from the past than now.

M: P3

P3 Young adolescent who are coming from poor background have a challenge of lack of education. So, you find that most of them who have not attended school engage themselves in different activities like that on of peer pressure. So you find a young adolescent engaging herself in sexual activities

M Any other challenge we may have left behind.

P4 Maybe just to add access to internet and social media is playing a key role in the young generations. Betting and cyber could both be positive and negative.

M Maybe the positive

P7 I’m just nodding

P4 Education

P1 Betting is part of income (Laughter)

P4 The other negative part could be pornography; Facebook those could be just the vices.

M Any other thing?

P5 Language being used by the youth now is a modernized language which most people don’t know. They are trying to mix French and Kiswahili in it so that you don’t understand but for them they really understand. They are also making it hard for them to be helped.

M Do you think these challenges affect their social, academic and professional life?

P1 Yes, definitely like alcohol and substance abuse and so this affects their judgment and makes them engage in sexual behaviors and changes their mentality and thus affect their performance.

M P3

P3 It affects their education in that those that are still in school when they engage in sex accidentally get pregnant the final decision will be to drop out of school.

M P7

P7 It does affect their performance like when we were discussing on the topic about changes in the body especially the ladies when they start experiencing the menses at times they may not know exactly what they are experiencing. Maybe this lady may not be in a position to afford sanitary towels so more often than not the lady may be embarrassed so she will not be able to concentrate when it comes to lessons. At times she may hide somewhere or go home for that period. And for the boys when they engage in social media for example the one that has been said here it really reduces the concentration especially the betting part of it. It reduces the concentration because you want to follow up what is going on in the social media .So when you get into it so much you start finding that the concentration is reducing.

M We have discussed more on those that are in school. There is also this other group that is working.

So how do these challenges affect their socio-economic activities?

P8 Maybe for example if we look at the social media like they said it’s a source of income so somebody is engaging himself into betting, gambling the moment he starts earning on social media then he will be able to grow educationally and economically. You will be living like fast lane. Now when they get the cash and knowing that next time they will also get the cash then his life will be stagnant position

M When you talk about their life is on fast lane what do you mean?

P8 Get rich fast.

M P2

P2 Still I will dwell on social media. I will go on pornography. When you are working and you are always watching pornography it brings addiction ,You get addicted to this thing and you always try even doing it so if that gets to that level than you have to get to masturbation .Masturbation is very addictive so someone who is supposed to wake up to go to something will not be able to do so because you masturbate the whole night ,so you will not be able to have time to go and do that constructive thing that you love to do for you to survive.

M P4 anything?

P4 Just narrowing down to what other participants have said so for betting you have to have an input there. You have to deposit some money. We see some of them who are in colleges using their fee money and you may not end up getting the money. We now get to hear of depression and suicide. And now when we come now to those who are taking alcohol and substance abuse we still narrow down to mental health which still goes back to depression. So, at the end of the day those who are also working may end up not performing as required as compared to others who are not engaging in it. I would generally say that the challenge we are facing is that if they don’t come out of it if they don’t get the support to walk them out of that particular aspect then if its employment you get even terminated because of the vices.

M P6 anything?

P6 I just wanted to talk about alcohol. Those who engage themselves too much in alcohol, it may hinder them to do their activities as expected for example when they are in school, it may make them not to perform well.

M Do you think adolescents and young adults have tried to deal with these challenges?

P5 I’m thinking yes at the same time no because some of them who have role models have tried and they are in the right track. Those who don’t are doing things not the way they are supposed to be done. There are those who will do right but inside they don’t really want to do right. There those who want to do the right thing to please you.

M P1

P1 I think they try. When you check on so on social media especially Facebook you find that there are youth groups or even individuals and they raise these issues. You know when you raise issues through social media people tend to give you advice. Those who know that we work in hospital come to us for counselling. I think they show some effort.

M P3

P3 I think they are trying to deal with these challenges. Some of them are trying while others are not. You find some adolescent are so introvert to the extent that they can’t talk out what they are undergoing. But for those who are eeeeehh. those who can share out their experiences they do come out and share their challenges like my colleague has said they come to us for assistance.

M Any other ?

P4 I have seen places where people are going for rehabilitation for alcohol addiction, drug and substance use. There is that part where the government has put up centers to rehabilitate drug abusers and again counselors both spiritual and professionals, they are trying to talk people out of addiction, masturbation, prostitution. There are also facilities that have come out with centers to specifically support this. They are called MAPS and mostly deal with at risk population mostly prostitutes and male having sex with male and mostly these are young people.so they are seeking out to support.

M Any other? Yes, p8

P8 Maybe I don’t know but maybe yes or no. I’m trying to assume or look at it at this angle. Somebody who lacks fee for education maybe we take for example a lady engages in sexual behaviors so that she can pay fees. She gets into these behaviors with intentions to getting the cash but later on she gets addicted to it and still continues with the behavior irrespective of whether she has finished her schooling or not. At some point there are people who to some extent get into bad activities or immoral behaviors mainly to support themselves.

M Any other way? P5

P5 Use of talents. There are others who are using talent. there are days where parents would say that I want you to be an engineer, doctor but this has changed because they are leaving them to do what they can do. There are others who are using their talent to make money and also to enjoy themselves.

M To what extend have young people succeeded in dealing with these challenges? p3

P3 (silence…….) pass

P7 Mmmh as we are just exhausting the points that has been said by all participants, they are trying, they are really trying. It’s only that what is lacking is empowerment. I think they need to be empowered so that they know what to do anytime they experience these small challenges. This is because they are still learning and young. They are trying but at times they still feel the challenges. I believe that if they are consistently and persistently empowered, they might do a lot from what we are seeing currently.

M Any other? P2

P2 To my opinion I think it’s not really exhausted whatever the youths are really doing to get help because still we can see them getting more challenges and the worst part that its going up to suicide. When someone cannot deal with something the end point is just to take their lives feel it’s not yet exhausted however much there is help but they don’t have the opportunity to get help.

M Any other?

P4 I would say its fifty-fifty. Fifty percent of the problem is sorted fifty percent have gained access to help while fifty percent is still not because we still seeing young people killing each other in schools, colleges, as couples all we hear is SHOKA (being killed by an axe)

M What do you mean by shoka?

P4 You see young people in colleges are using other partner or gender as a source of income. You find that by this indulgence you find that you are trying to get source of income at the same time the other person has his own expectations. You find the other person murdered so we are trying to question this aspect of drug use and acceptance. That means that they are not accepting that they are poor and cannot support all their needs. This leads to the aspect that jobs are lacking and so most of the young people are not working.

M P8

P8 Yes, just maybe to add something I think it’s not yet truly exhausted. We need to do something. We need to really change our education system and find ways of nurturing talents from way down when somebody is still very young and we try to come up with a way we nature talent from way back and change the education system. We find that you can be in school for eight years you add another four years but you find that after that you come out with nothing cause life itself is a school. When you learn something from way back you can find that when you are out of school you can do something else which is different because most of the things, we learn in school we don’t apply them in our day to day life. We read them in the books and leave them there. At the end of your college your eight years in primary it another learning session altogether

M Any other thing?

P3 I think nowadays Kenya has run into the digital world because in school the health talks that used to be there because they think that all students or pupils who are in school has knowledge on internet so there is no need for health talk.

M And do you think that support is available for these young people?

P5 Yes especially social media. This is a support system. Maybe if I have information that I would like people to know then I use social media.

P1 I think there is support like my colleague said we need an education system where the young people are being trained at a young age to identify their talent like the government has come up with a new curriculum which is talent based so I think the government has put more effort to help these young ones when they finish school. You know, previously when you finished form four you could do nothing. You even finish college and you don’t have anything to do. Talent you can finish class eight or form four and play football. This can curb issues of unemployment, alcoholism and peer pressure. I think there is an effort.

P2 I’m still thinking

P7 I think there could be enough support system but now but most of these adolescents could not be in position to know what to do. I think what need to be done is sensitization so that they be informed. So, you may be faced with challenges but you don’t know the protocol to follow. I think when you sensitize these people with information so that they know what to do at the right time it will become very easy for them when they have a problem to be able to solve the challenge. I think as much as those systems are there these guys may not be having any information, to me sensitization is very vital.

P1 Just to reinforce what p7 has said there is …. Because nowadays we see people going to school like the public speakers and talk to them. And even Sundays I see “kubamba” (gospel show in television) they visit schools and sensitize them. But still more needs to be done.

M Yes, P8

P8 I want to agree with what P1 and P7 has said but also disagree at some point. There is support system at the same time lack of sensitization. You take the urban and rural sensitization. Sensitization in the rural is different. Somebody who is the rural cannnot get the same sensitization as the same person in the urban area.

M P2

P2 I think there is the support system but you may not see the intensity because the challenges that the adolescent face they are not identified at an early stage that they can easily be solved. In our society people are too busy looking for money including parents such that they do not have time with their children plus the adolescent to even check if someone is having a problem. It could be very easy to identify a problem if you are close to your family You talk to that person think the support system the problems are not identified early.

M Any other thing? yes P 6.

P6 What about sex education that was introduced in schools. can it be helpful in to the youths?

P5 Sex education was introduced in schools from class four to eight. they are now being taught about sex.

P8 I wanted just to add on what P2 said. When you are growing up your parents tell you that you go to school and after that you look for a job. If there is no other thing you can do and there is no other support or any other sensitization or empowerment that it what you will tell your kids. ” your work is to read and go to school” so, as the generation goes the parent looks for money schools that child and the child looks for money.

M Thank you very much before we procced we will take a short break.

M Now we will continue to sexual and reproductive health. What do you understand by the term sexual and reproductive health? P7 you look like you are ready. (Laughter….)

P7 Sexual is an act of getting intimate between opposite gender. Aaaahh and again there are different types of sexual intercourse that involve even men with men and female and female so it is so it is intimacy between two or more people. Then reproductive health is generally health that touches on the sexual organ of a human being.

P5 According to P5 (Laughter….) It’s the well-being of sex and this can be both male and female. It can also mean the act, it can also mean the reproductive organs.

P5 I think it has to do with sexuality and then reproductive organs in both male and female. Health is a state of well-being. When we talk of reproductive health, we talk about wellness of the reproductive organs.

P8 It’s just the well-being of sexual organs (Laughter)

M Summarizes the feedback and probes further.

M How important is young people access to sexual reproductive health services such as contraception, STI prevention e.g. condoms, PREP, HPv vaccine?

P4 It is important for them to access sexual and reproductive health because on there are in the age where they are developing fast and also, they are informed. They are seeing changes in their body and so with peer pressure and they may be engaged in one way or any other way.so it’s important for them to learn and also access the services because it will be able to prevent diseases like STI and any other. Under sexual and reproductive health, we also have cervical cancer screening because they are sexually active.

M Any other?

P8 Maybe I will just say that without them getting these services we can say that it can cause harm and it can also cause disability.

M How can it cause harm and disability? Just expound on that.

P2 If you don’t access these services you may develop some diseases for example cervical cancer which can cause death. If it’s not diagnosed early and treated, then it can cause harm to you and others.

M Where do young people currently access information on sexual and reproductive health?

P4 Internet

P3 In the health centers and some of them access it in schools.

P1 Fellow peers.

P5 Social media. There is something I want to echo. There was a talk in the previous session that they teach sex education in school and also sexual and reproductive health in school. I want to confirm that this not happening because they don’t allow us to teach sex in school. If you are a healthcare provider and you go to school and then you get a question on sexual and reproductive health and issues of contraceptives then you don’t have to answer it there but at the youth friendly centers’ remember we have been chased thrice because we talked about it in some schools. The first one was because we talked about it. I don’t know if it’s the school or because they think that you are teaching the children bad manners. They are forgetting that this is something that is very open and the kids are doing it so it is happening. In the universities you can talk about it freely.

M Does this mean that there is no set curriculum for this?

P5 It’s there in written like biology and science so whatever you are to learn i.e. there is the vagina, penis and reproductive health. They don’t talk about condoms.so if you go there to ask to be given a chance, they will ask you to tell them what you want to talk about.so when they go through and find things like condoms and contraceptives they tell you not to talk about them. Remember that some of them are catholic schools and catholic schools don’t allow this. Actually, Muslim schools you don’t touch anything to do with sex and anything to do with reproductive health. That is the biggest challenge happening in Kenya I don’t know of other countries.

M Where do young people currently access information on sexual and reproductive health?

P1 Hospitals

P2 Google

P3 Youth friendly centers

P6 Health centers.

M What is the ideal place/location where sexual and reproductive health services easily and conveniently be accessed by young people?

P5 Public hospitals

M Why?

P5 Because its easily accessible and affordable. Some of the services there are free and there are also youth friendly centers. There are private youth centers and public youth centers. Like Family Health Options Kenya (FHOK) is a private youth center.

M To what extend are the sexual and reproductive health services offered to young people meet their needs and expectations?

P1 Yeah. I think they meet.

P4 I say to some extend but not one hundred percent. As we were saying that they are supposed to get these services in public facilities, we have shortages and stock outs for commodities even family planning commodities. They could be accessing but not to satisfaction.

P5 I’ve just remembered something. There is staff attitude I go there and maybe I want to access condoms and when I get there the staff is looking at me like “why are you starting sex so early what is your problem?” Then I will have to do it without anyone knowing or not just do it. Another point is that am thinking adolescent who are HIV positive get access to these services than those that are negative. This because when they come to clinic there being told that if your sexually active are told to take condoms, cervical cancer screening. if you get these adolescents who are HIV negative when they come to look for these services, they are referred back to the youth friendly center.

M Others prefer buying the contraceptives themselves

P5 Yes, especially the emergency contraceptives which are only to be taken twice but it’s being abused. This is because after sex then people run for it. The Pharmacists know well that it’s supposed to be taken twice will still sell it to me even if I’m there for the tenth time he will still sell it to me because he needs the money.

P4 Okay she has raised a good concern and she’s the one who proposed that it should be given at public facility. You Know why? Public is accessible and cost effective as compared to private. And when you say private when you go to purchase in the pharmacy nobody will ask you who you are purchasing for. Because they are in for your money and they are in for your drugs. Actually, the public facilities are the best to access reproductive health services. Especially for the young people because outside there it is abused.

M And do these young people have challenges in trying to access to sexual and reproductive health services.

P4 One of the participants already mentioned about attitude. Since they are young, the healthcare provider will be looking at them and wondering why these are young people are engaging in sex. There is that aspect of attitude and even norms and standards that we put as a society, that’s why we have those that are opting to look for other means to solve their circumstances and situations. Probably, we can say that there is implication of cost because they will not go to their parents and say that they want to buy contraceptives. There is the aspect of cost effectiveness and the attitude of healthcare providers. We can say that it our African culture.

P6 I can talk of information. Right now, we issuing Arvs, some of them come here but others don’t. Some of them have attitude

M Any other thing? p3

P3 I have an input I don’t know if it’s true but my colleagues will help me. Parents denial. A parent denies a child from getting these services. You find that the child may want to take a condom but the mother refuses by saying that the child is still young. She says that she won’t be able to give birth in future if she starts using the contraceptives early.

M When these parents refuse do, they still go for these services?

P3 That’s when you find that this child goes for these services from quacks without the parent knowing.

P7 To add on what has been said most of these adolescent are people who are so secretive. Their privacy and confidentiality should be assured. Most of the time you find that they come in but if you can’t handle the issue of privacy and confidentiality plus the perception that is there that you are just a young lady and why are you coming for this services at this time. It’s not only about preventing diseases.

You see these young ladies when they come with that mentality that they want to seek sexual and contraceptive services to prevent pregnancy not knowing that there is more to this than that pregnancy. They can get other infections. So, when they come, they should be informed, but most of them fear because of the privacy.

P8 Most of what I wanted to say has been said so maybe to add I will talk about the access to facilities. Maybe the location where the facility is distance can also be a challenge. This can make one opt to quacks or even herbal medication.

P2 Maybe I could add something. AYA have the fear of being judged. Have you ever gone to a chemist to buy something that will make someone look at you surprised? Most of the adolescent are actually growing and exploiting all those things that are coming. I think they may fear going there because they fear how the healthcare provider will react. So I think they fear being judged by the society which has put that somebody has to reach a certain age to be able to access contraceptives. You can only access it only after giving birth.

P5 Most of them have been said but I have one. Religions like “ROHO, LEGIO MARIA” ,catholic and muslins .These are topics you can’t share when they are there. You can’t talk about contraceptives. surprising enough they are the once who have the largest population of early pregnancy, abortions and UTI. Then there is adolescent self-esteem. You see an adolescent who is grown and something tells you that they are sexually active but you ask that adolescent “Are you using condoms?”

And then they ask you “Who told you am sexually active? This is because you had met this adolescent somewhere and you really want to help but you can’t because he/she has said no. Our parents know so much that they are supposed to be teaching us on sex because they know it exist but the same way p3 said they don’t want one to take contraceptives. But at the same time when they realize that your sexually active, they are the same people who will force you to take them or even do it without your consent. I have forgotten another important point.

M Do you think being in schools or colleges affect their access to sexual and reproductive health services?

P4 I think they have access. If we talk of colleges. Most colleges have access to sexual and reproductive health, they have condom dispensers. They even have dispensaries that have pills. Maybe now with focus in primary and secondary schools them they may not have access as per what we have discussed they have information that is not comprehensive to the aspect of sexual and reproductive health when they want to engage or disengage. Somewhat they have access somewhat they don’t.

P5 I’ve remembered. The side effects of contraceptives. Even if we look at the location for example if am seventeen-year-old and then you put an implant on me when I go to school, I will always be stigmatized so I will forever be on sweaters. There those that will make you fat or thin or even bleed for long.

P1 I think I will reinforce what had been said because if you check on our school set up there no facilities to stock those commodities. The dispensaries are very minimal and they only have medicine for ailments.

M How comfortable will young people be discussing their sexual and reproductive health concerns with a lay health provider of their mothers age?

P1 Not comfortable.

M Why?

P1 Just because of the society perceptions. If you are an adolescent and your still young you are not to mention anything on reproductive health.

P5 I’m thinking like the government has tried to bring the youths on board so that it becomes peer to peer but at the same time we are looking at it like if you get an older provider and you are sharing one on one then you get more sense to it. She’s like a representative of your mother. If I get a younger person we can just talk and maybe, I will lie to that person. It’s both positive and negative. The government should bring both the old and the young. You can choose who to see you. If you get a female or a male one.

P8 Just maybe to add on what p5 had said. I think both peer to peer and peer to adult it depends on the rapport that you create with the person or how you welcome the person. The rapport that you create is what will lead for you to get what you want to get from the peer or for him/her to deny you that information.

M What issues would young people be comfortable discussing a lay female healthcare provider of their mothers age?

P3 I think they can discuss issues to do with health like changes in their body.

P6 I think they can also discuss challenges that one can face in the services that they are receiving.

P4 They can also discuss on having friends of the opposite sex.

P7 I think we cannot generalize on individual basis because it will also depend on the preparation of the lay healthcare provider and the friendliness will put you on the level to disclose more or keep some.

P8 I will still bring it back to the relationship that you will build will lead to everything just flowing.

M When do young people talk about sex with their friends? Do the friends they talk about sex with tend to be of their own gender or different?

P1 I think at the age of fifteen mostly but at that time is mostly same sex. You find boys talking about it and they don’t know even what they are talking about. (Laughter) Maybe they heard it somewhere. You find them asking “Do you have a boyfriend?”

M How do they talk about it?

P4 Just to add on what he has said the age between ten to fifteen i.e between class eight and form one where there is biology where you have started reading and discussing about reproductive health based on that you know that is when information starts trickling in in your mind and you start internalizing. That is when they can start talking about it based on what they were discussing in class .That is also when the teacher introduces the difference between a boy and a girl on what happens when a boy and a girl have intercourse.

M They prefer to talk about it with same gender as p1 had said do you agree?

P4 This is because most schools most schools if not mixed school then they are either for girls or boys. Also, in class you tend to separate because in school if you are seen with the different gender then it will raise some questions. You will be called to the staff room to explain so you socialize based on your gender.

P8 I don’t want to disagree with what they have said but we are in 2019. We have internet, television and there are several soap operas going on and just like p1 had said people tend to talk about these things without knowing what they are really talking about. It starts even from babies from less than ten years because these are things they will start seeing on television like people kissing each other. They will start doing the same without really knowing what they are doing. This is something they have started building inside them in their heads so sex starts from when you are born. (Laughter)

P7 What I wanted to share (Laughter) when do they start to talk about the sex. They start talking about this immediately they start feeling or seeing those physical changes That is the moment they start having enquiries when they start having things like wet dreams. They start to share on what they had experienced and learn more on sexuality.

P5 Unlike boys, girls when they start experiencing things like menstrual periods especially when they are in school then the others will laugh at them. From this then the girl will be told that she should not be near boys. I can confirm that my mum used to tell me that when I start my menses then I should not be near boys but I could not understand why I should not walk near boys. My mother had never talked anything to do with sex but right now that am old enough then she tells me that you know we have contraceptives and family planning. I don’t know what she means.

P7 In our setting we are brought up in a culture that does not embrace sex. It is a culture where you can’t talk about sex with your child. It’s a challenge and I wonder how parents get over all these issues for the parents to open up and even the child to open up so that you share. That why it’s very difficult for the parent to probe the children.

M When do young people have their first sex?

P7 During that time that they are playing. When they are playing there are others who are old enough and understand what they are doing. They are the once to introduce the once that are learning (Laughter)

P8 I was just saying that it happens that when they are playing, they tend to imitate what their parents do. Like things that they see on television and the things they see going on in the house. They play pretending to be the mother or father. I remember when I was young everyone wanted to play the role of acting like a father so that he can sleep with the mother. (Laughter). The children they have their bedroom far away.

P5 There is also rape through people that we know.

M Are they really pressurized to sex?

P5 Like what is trending now everyone wants to be a baby mama (to have a child with someone without marriage) some parents even agree for their daughters to get pregnant for those rich people to get money. There are also parents who trade their children in exchange for something like fish. There is also pressure from others who tried it and then tells someone else to try it because it was sweet.

P1 There is the poverty aspect of it. You see young girls having sex for the sake of money. This is because they either don’t have parents or their parents might be poor.

M How do people react when a young woman becomes pregnant /a young man becomes a father?

P2 I think it’s always shock and it reduces the self –esteem. You find the pregnant adolescent putting on sweaters all the time think it’s always traumatizing especially to the young girls.

P7 You see the young ladies who get pregnant it’s very hard for you to face your parent when your stomach is swollen. You can’t really dare so most of the time the moment a lady realizes that she is pregnant then she will be advised by the peer to abort. They tend to do abortions Even the boys who have fathered the children do not take responsibility but run away. It will be on the lady to take full responsibility of the child and you see at this point the lady is still going to school and cannot guarantee the upkeep of the child so she will seek the options of aborting and this one leads to many deaths.

P1 They are defended by their fathers. (Laughter)

M How?

P1 They deny the pregnancy. They say” My son has never done it”

P5 Even us if we just go back do these girls and boys know that there is a pregnancy? No, they don’t. Most of the time when a girl is pregnant, she doesn’t know because she might be doing it for fun. The boy on the other hand doesn’t know what is going on.

P4 I think also thirteen or fourteen-year-old don’t know that they are pregnant but its only their parents who will suspect because of body change or by taking them for testing. And also they are informed because recently we had a case of a form one who came for prophylaxis the boy had information that he is positive but the girl was smaller and the boy knew that he was to bring the girl to take post exposure prophylaxis and even went ahead and bought contraceptives for the girl. Though they were in the same school. different levels have different levels of communication and information. The boy may take responsibility or even refuse.

M Do these young people know what safe sex is?

P5:P7 They know

P5 They know because there is this advert of “Kuwa true” (For condoms) I think it was removed because the way they used to demonstrate how you put on socks (condoms). And my cousin will ask the mother that he wants the chocolate one and he didn’t know to respond to that. The only thing he knew was that there was the chocolate and the strawberry (condom flavors). Maybe the media is trying to help the kids but the parent is barring them.

P5 Maybe the aspect of sex is a matter even to adults. Sometimes people are carried away by the moments and they end up messing and they find out latter that they should have done. That is when they come back to their senses. The moment there is too much pressure and people cannot think they may have the information but the pressure and hormonal imbalance cannot allow them to do the right thing.

P3 I think they have information to safe sex on social media as p5 has said.

M Do they really ensure safe sex?

P7 I don’t think majority of them really ensure safe sex or practice it. That is why we are seeing so many teenage pregnancies. It really tells you that there is something going on. They don’t practice safe sex but just do it for the sake.

M What challenges do young people face when trying to promote safe sex?

P8 Maybe on that I could say that like it was said being carried by the moment, peer influence from colleagues like when one tells you that I did it and that thing is sweet without a condom. I will also try it without a condom.

P1 Also, there is the economic part of it.you see these young boys they don’t have money to buy condoms and when the opportunity presents itself they just do it. Also, the venue because they do it in rice plantations (Laughter)where there are no condom dispensers.

P2 It is not always something that they normally plan like today we are going to have sex. It’s something that just comes out. (Laughter) it comes out in those moments that you were not expecting. The young ones they feel that why should I carry a condom whenever I go. They don’t have time to go and buy while the girl is there ready because the moment you leave her there and go, she might change her mind. The moment they normally get they cannot bargain for safe sex.

P8 Maybe on that generally looking what is the size of the condom. Imagine a person fifteen-year-old (Laughter)how will he fit in the condom?

P1 They are not condom friendly.

P8 Even for the female condom. For the women don’t know if it will fit. Maybe the information on how they put it on that is the information they lack. But the condom is not friendly to the young ones

P8 Then maybe one last thing this sponsor society (having a sexual affair with an elderly person) we currently live in. You know that men are very cunning so, when they get these young ladies you know they ask them do you want to have sex with or without a condom. He then tells them that if they use condom is five hundred shillings and without condom is one thousand shillings. (Phone rings)

M How would young people feel if they are assigned a lay healthcare provider as a confidant and source of information for their sexual and reproductive health?

P7 To me I think they will tend to learn more from the healthcare they will tend to believe all that they are being told. It will be a good moment for them to ask question and explore everything.so it be really helpful to them.

P5 I’m thinking about what she said earlier that is attitude and rapport. She can be brought there but if nobody is talking about it then its …how you establish and advertise it also matters.

M What attribute would they like to see on this lay healthcare provider?

P7 This person must be jovial. She must behave the same way the young people are behaving.it should not be someone who is tough talking and has a serious face.

P3 She has to be someone who maintains confidentiality.

P8 When you are talking about adolescent, these are tricky age set. it’s upon the adolescent to choose. There should be both male and female lay healthcare providers so that the adolescent is able to choose which one they prefer.

M How would young people feel about receiving their ARVs together with their sexual and reproductive services?

P5 It would be good because we are checking on time. Most youths don’t like ques and they don’t like being put on wait. They want that when they get there, they say their problems, get their stuff and go away. So, if I finish here and am told to go somewhere else then am likely to go away saying that it’s not important.

P4 I think it will be awesome because we all want to go to a supermarket where we get everything, the one stop shop. I think it will save on time and stigma issues will be addressed. Health providers should be trained on all manner of screening and offering other services.

P8 I’m just thinking that its best receiving it in one room because that is the moment, he/she can open up. (P1walks out of the room)

P8 I just wanted to ask do you think the social media, television or radio like the advertisement for condoms promote sex?

P4 I’d ask in the first place this is a five-year-old watching the advert. I get the sense that they are protecting e a five-year-old would watch it but will tend to ask. Having that luxury to explain and explore to this five-year-old would be a challenge. It will depend on the age group (P1walks back to the room) The society give us pressure to relay this information to these young ones.

P7 Maybe I would want to add that before any advert with adult content is run on the television there has to be a disclaimer. There things that cannot be aired when the children are still awake.

P8 This is the culture that we live in where we treat them as children yet they are so informed by the internet and television. The moment you deny a sixteen-year-old that he or she is still very young

M Thank you very much, it was a wonderful discussion and thank for your time.
