# Supplementary material for: Preferences for accessing sexual and reproductive health services among adolescents and young adults living with HIV/AIDs in Western Kenya: A qualitative study
Source: PLoS One. 2022 Nov 16;17(11):e0277467. doi: 10.1371/journal.pone.0277467 (PMC9668131; doi:10.1371/journal.pone.0277467)
Supplement: S2 File — (DOCX) [file pone.0277467.s003.docx]

I Feel most welcomed. I would like us to begin our discussion. Are you able to tell me about yourself..

R Okay…. I am called….……(Name withheld) I studied at Arina Primary School next to Manyatta estate. I finished my Form IV at Chulaimbo High school and I am currently waiting to join University.

I Okay that is good. What is your age?

R I am 18 years

I 18 years….. What is your level of education?

R Form IV

I Form IV, so you’ve done your secondary… Are you married?

R No

I Are you engaged in any activity or are you employed?

R Currently I am not employed

I Okay, that is good.

I Generally, what challenges do you face as a young person?

R Mmmm as a young person there are many challenges

I Mmmm

R Actually sometimes you need to put on clothes or shoes and yet you do not have that cash. So that is is a very big challenge

I Mmmmm

R Also lack of employment

I So, you’ve told me that as a young person you need money. You are not able to buy things like shoes, (mmmm) clothes. You have also talked about employment.

I Okay. What other challenges?

R Actually those are the major challenges.

I Okay. As a person those are some of the challenges you are facing?

R Yes

I Ahaa.. So do you think these challenges affect your education and professional achievement or social economic achievement?

R No, I don’t think so. You know these are challenges you can overcome. You know these clothes and shoes are just luxury. You want to put up with someone’s dressing

R It is not a must. You can for go. (Laughs..)

I Mmmm (Laughs….)

I So you think clothes and shoes are luxury?

R Yes, you can for go them..

I Is there a way the challenges you have experienced affected your academics or school life. Even maybe when you were in High school before you finished

R When you are in High school you are really affected. You know sometimes you have closed schools; you want to join your friends during the holiday yet you don’t have something to put on. You can’t show up there with very bad clothes (Laughs…)

I (Laughs….)

R You feel embarrassed.

I You feel embarrassed…

R Yes..

I Okay. What do you mean by bad clothes?

R There are some clothes that are out of fashion..

I Oooh out of fashion

R Yes

I So you feel embarrassed

R Yea

I Okay. So how did that affect your education?

R I can’t say it affected my education. You know those things you can avoid. If you see someone dressed better than you it is not a must you join him or her. You can just put up with people of your level and move on.

I mmm.. .Okay. As a person, is there a way you have tried to deal with these challenge?

R Sometimes I deal with them. You buy some few clothes and you dress well maybe once not everyday. You know you can’t afford that everyday

I Ooh, that’s a nice way. What else have you done to deal with the challenges?

R Actually now I am just moving on very well

I You are moving on very well..

R Yes

I Okay. Initially you told me that apart from having money to buy a few items you also talked of unemployment as a challenge. So, is there a way you have dealt with this challenge?

R I have not dealt with it. (Pauses…) because you know it’s not easy to stay at home, wake up, be at the same place like you are just in the house. You can feel bored, you feel like someone should give you some sort of employment so that you can make a living there. Go somewhere and do something then come back later in the evening.

I Mmmmm….For the other challenges you talked about of not being able to get money to buy a few things here and there. You said once in a while you can buy but some things you cannot control. I mean you try to live your life..

R I try to evade them….

I Yea.. you try to evade them. So with employment you have not ben able to find a way of dealing with it?

R I have not found a way. You know you cannot force someone to employ you.

I Mmmm

R Yea

I That’s true. To what extent have you succeeded in dealing with these challenges as a person?

R I can’t say I have succeeded because I have been at the same place that I have been. There is no change

I mmm.. There is no change

R Yes, there is no change.

I Okay. Do you think there is support available for you to deal with these challenges?

R Support….

I Mmmm

R There can be support.

I Like?

R Maybe if I can get some sort of employment..

I Mmmm

R Yea

I What I mean is do you have people who can assist you through these challenges? Like earlier when you mentioned about you not having enough money to purchase maybe clothes or to go and meet your friends….Are there people help you in such scenarios?

R Yea

I Mmmmm

R Like I told you that you can buy those clothes maybe once and put them on. You can’t afford to do that daily. I can borrow my sister and she will give me a few cash and I manage and buy a few stuff.

I So your sister is like your support system

R Yes

I Okay. Apart from your sister, any other support available?

R None

I Okay, that is good. We were looking at the general challenges young people experience. I want us now to move on to HIV care services. So I want you to describe your experience the day you got enrolled to HIV care. Like, when was it? How long after you knew you are HIV positive? How did you made a decision to enroll to care. I just need to understand your experience.

R Actually, I cannot explain hoe I got enrolled to HIV care. I was enrolled while I was still a young boy. Class three I think…..

I Mmmm

R I got know of it when now I was in Form 1 that I am HIV positive. (Silence…). I can’t explain that..

I You have told me that you got enrolled when you were in standard 3. At that time you did not know your status

R I was just taking drugs and did not know what they were used for

I Mmmm

R I came to learn about the drugs when I was in class 8 waiting to join Form 1

I When you were waiting to join Form 1?

R Yes

I How was the experience when you got to learn of it. How was it?

R When I learnt that I am HIV positive, I took it normal because I saw we were a bunch of kids. We were many, I just felt happy…

I Okay.You felt happy…. What do you mean by feeling happy?

R Because I felt were not left out, someone still cared.

I Mmm….there is someone who still cares. That is good. So at that moment who disclosed to you, is it your mother or the care provider. How did it go?

R No, my mother never disclosed to me. The disclosure was at the hospital when I came for support group

I Mmmm

R It is when I came to learn of it

I So the disclosure was at the facility when you were attending your support group. So at that moment you were already enrolled in care and you were taking your medication only that nobody had explained to you that you that these are drugs for HIV. You came to learn of it later…

R Yes, later

I What went through your mind at that time

R Actually I did not have many thoughts. I was just someone who was quiet. I was not talking a lot. So I just thought this thing is for my own good. I decided to just take my drugs.

I Did you feel at any point that it could be challenging or did you feel like why me? Have you ever felt like that?

R Sometimes you are at school and no one knows your status and they talk about those with HIV. You get bad ideas, you sit somewhere and think.. I mean you have weird thoughts like why me? Why am I taking these drugs? If you are not…..You can even decide and leave these drugs

I Mmmm Okay. Up to this level what has enabled you to continue engaging in care at this facility?

R I can say that I want to be good so that I may not fall sick and just to keep on taking those drugs. You know if I do not take those drugs my immunity can go down and I get attacked by several diseases.

I Okay. I meant what has enabled you to continue coming here. Like is the staff? The quality of service

R Actually at the facility there are so many caring doctors. Even when they talk to you they get encouraged and even want to come here very often.

I Mmmm

R So that you can take the drugs

I : Okay. Apart form these friendly staff; and those who work here and encourage you, what else has enabled you to continue engaging in care here

R When we come together as adolescents we meet and encourage each other. So you come to the hospitals to pick your drugs as you meet your friends, have some fun and you feel encouraged.

I How far do you live form this facility?

R Actually I live in Manyataa.

I Mmmm, Okay

R Yes

I That is good. So you have told me that you have friendly staff, even you get encouraged when you come to the clinic because you have friends your other friends whom you meet with here whom have enabled you to continue engaging in care. Is there any other thing you would like to add on that?

R (Laughs..)No, I have said everything.

I (Laughs..) Okay, that’s fine. Can you tell me instances you’ve experienced stigma or you have been discriminated because of your HIV status.

R I have never experienced stigma because in my family everyone is caring and in school people know me as that boy who does not care. I do what I want to do. You cannot let someone decide for you your life. You do what is best for you

I Mmmm. So you have not had an instance where someone is discriminating you because of your status or even maybe when you were in school someone saying we don’t want him to touch this or that. You have never had that experience?

R No

I And have experienced someone go through that?

R Yea

I Ahaa……Tell me more about it

R The boy was from the opposite class. His classmates did not like him because he had some…..his skin was not good. He was kind of sick but it was not HIV. Actually I think it was cancer. They were abusing him and he finally got transferred to another school.

I Okay. So people were abusing him..? How did you feel about that?

R Yes, they were abusing him. I felt sorry for him but I couldn’t do anything.

I Mmmm

R Yea….

I Okay, good. Tell me anything you know about ARVs (Antiretroviral drugs)

R ARVs are drugs that you take when you are HIV positive. You take them in order to boost your CD4 and weaken the number of viruses in your body.

I Mmmm okay. Any other thing you know about ARVs?

R They help you to be healthy.

I Any other thing?

R None…

I Tell me about the time you missed taking your ARVs. What happened?

R Actually there was a time that I was missing, not willingly. You know sometimes you are with friends, those friends that cannot leave your side. You just have to miss those drugs. When you go to the toilet you are with them even when they are not pressed they just follow you, you got eat they are with you. Now you cannot go with them to take your drugs because they do not know your status. So you just have to miss those drugs.

I So you have missed on several occasions?

R Yea, before

I Before? Because of the friends that you are with almost everywhere with them so it was challenging for you to take your drugs so you opt to miss..

R Yes

I Okay. So could you please share with me the challenges you face keeping your appointment?

R Sometimes you have an appointment, you have to go and meet someone. If you go to the clinic you spend a lot of time there because you cannot fasten the program. You cannot tell them to do faster because you know they have their program. So you have to miss some appointments.

I Mmm, okay. What makes you miss your appointment?

R Sometimes you can have an appointment at the facility and yet you are not in Kisumu. That, then you can miss though not willingly missing.

I When you are not in Kisumu that means you have traveled or?

R Yea

I Okay. So that is the only challenge you could be experiencing?

R Yes

I Any other?

R There is no other ….

I Tell me about the last time you missed your clinic appointment

R Actually that is what I was talking about. The last time I missed I had travelled to Nairobi for the holidays with my younger sister. I was to come back the day after the clinic, so I missed the clinic.

I So, did you miss drugs?

R No, I had drugs

I You had enough drugs

R Yes

I Okay. So, what did you do when you missed?

R Actually when I cam e back I went to the clinic the next day

I Okay. What do you feel about the HIV care services you are currently receivg

R We are receiving the best services. There is no discrimination, everyone at the facility is loving, they encourage us.

I Aahaa that is good. Any other thing about the services you are receiving?

R I don’t have any other thing

I Okay. How do you think your encounter with HIV services can be made more satisfying?

R Actually that one is very tricky

I How?

R Maybe when someone is coming to pick drugs on a particular day, you can ensure that those coming are not many the way they usually are. Maybe they can come in lots , some from 8am-9am, you finish with them then others later to avoid crowding at the facility.

I mmmm to avoid crowding. What you are trying to say is that people are given appointments in lots for example, I am just thinking and I am not sure if what I am saying is right. 10 people to come between 8am and 9am then another 10 people? Like that?

R Yes and then you have them in a class and educate them about HIV and complete their files at once.

I Mmmm

R Yeah

I Okay. For your encounter with HIV services to be more satisfying, you have talked of reducing ques, seeing people in lots, educatiing them as a group..

R Yeah..

I What else?

R Have some exciting stuff to encourage the adolescents to come to the clinic because you know some people miss knowingly. They don’t want to come because of those long ques and it is tiresome.

I Ahaaa, so you are proposing some exciting stuff. When you talk of exciting stuff which ones are these?

R Maybe when we come many people are at the clinic you have some mind engaging games so that we can play to avoid feeling that w are wasting time.

I mmmm

R Yea

I Mind engaging games like which ones?

R Like playing cards. You know while playing you do not feel like time is moving but when seated you keep on looking at the time and feel you are wasting time and feel like going

I Mmmm, okay. That is great. I like that.. So, how does being in school for young people like you affect their clinic appointments and taking their ARVs?

R At school sometimes you may be having your appointment yet during the same week you have exams. If you are in boarding school, you cannot be allowed to go out of school because they will see as if you are a joker as you are missing exams willingly. So you end up missing your appointment.

I Mmm, okay. What else affects their clinic attendance?

R (Silence….)

I Just exams?

R Yes, I think that is the major challenge

I So they end up missing their appointments because of exams?

R Yes.

I How about just taking ARVs while in school

R If you have disclosed your status to your teacher you will be taking the drugs well but if you keep them for yourself, you will end up missing the drugs.

I Mmmm

R Yeah

I Okay. Any other thing you would like to add about being school for young people affect their clinic appointment attendance and taking their ARVs?

R Actually, I think when you are in school you just have to let out your problems to the teachers. Without the teachers you cannot do anything. You cannot write your own out leave out sheet and leave the school without teacher’s help. So by that, if you keep your own drugs you cannot do anything with them. You can miss them, you know you can be in class and the dorm is closed. You have to call a teacher to escort you to the dorm because you cannot go by yourself.

I That is good. We are going on well. I want us now to discuss on sexual and reproductive health. In your own words what do you understand the term sexual and reproductive health?

R I think that is the ability of someone to be sexually active and also to reproduce.

I Mmmm, okay

R Yes..

I When we really want to understand more on sexual and reproductive health, we talk of things to do with contraceptives, and when I talk of contraceptives, I mean (family planning), this STI prevention such like…

R As in?

I I really wanted to have an understanding of what you are saying on sexual and reproductive health is. So, in your own words what do you understand by that.

R Sexual and reproductive health is the ability of one to be sexually active and also the ability that he/she can reproduce

I Mmmmm

R Yes

I Okay. All you have said is actually right and that is encompassed within sexual and reproductive health.. We can look at sexual and reproductive health in a way that how easily do we access information in relation to sexual and reproductive health, we talk of safe sex, things to do with family planning, sexually transmitted infections/ diseases and prevention. Okay?

R Yes

I How important is your access to sexual and reproductive health services?

R It helps you to.. Maybe when you are engaged in sex, you know what to do and what not to do. Actually you will know that you have to use a condom to avoid unwanted pregnancy

I mmmm

R Yeah

I So, where do you currently access information on sexual and reproductive health

R When we come for support group, we are taught everything on reproductive health.

I Okay. What are you taught on specifically? Maybe you can highlight a few?

R We are taught that if you are about to have sex, you should ensure that you have a protective………you have a condom, or you take some drugs (pauses) I have forgotten the name

I You mean Prep?

R Yes, Prep to avoid contacting HIV if you do not have

I Mmmmm

R Yes, only that.

I Okay. So you have said the only way for you to access information is just through the support groups. Is there any other way you can access this information?

R Yes. At the estates, there are people who call out people for discussions at some halls, they entertain and also educate.

I Okay. Are they groups or organizations or what are they?

R They are organizations

I Mmmm. Okay. So they call people at the estates and teach them?

R Yea, they give gifts so that you can attend.

I Ahaa, that is good. So, how else do you access apart from the groups and support groups at the clinic? How else do you get access to this information?

R There is also the Internet. You can download whatever you want to see

I Mmmm…

R Yes.

I Okay, that is good. Any other place you can think of?

R None

I What is the ideal place/Location where you would like to access sexual and reproductive health information and services?

R At the facility

I Mmmm, so you think the facility is the ideal place to receive this information and also the sexual and reproductive health services?

R Yes

I Why do you think it is the best place?

R You know if you access those other things maybe there are not good places you can get from them. But here you will be told the good ones and the bad ones

I When you talk of you will not get good information from them. Like which one?

R You know some people teach you that you should use a condom but they will not teach you how to open it or how to put it on

I Mmmmm

R But here you are taught many things.

I Mmmm, so you feel the facility will be an ideal place because you get all the information.

R Yea

I Okay. To what extent are the sexual and reproductive health services offered to young people like you meet their needs and expectations?

R You know young people are always sexually excited. You want to explore many things…. I don’t know how to explain…(Laughs….)

I You can explain, I will understand. Don’t be afraid

R I don’t know how to put it

I Just put it in your own words I will understand

R You know young people are usually sexually. They are… You understand adolescents…

I Mmmmm

R They like practicing sexual behaviors

I Mmmm

R Yea

I So, do you feel the sexual and reproductive health services offered to young people meet their needs?

R Yeah

I Okay. so it meets their needs and you’ve talked of them wanting to.. they get excited about this sexual thing. Maybe trying to explore if I get you right, to what extent do you think the sexual and reproductive health services offered meet their needs. Do they meet their needs and expectations?

R I think they do meet their needs because they are taught what they want to hear and also practice what they are taught.

I Mmmm.. Good.

R Yea

I What challenges do you face as a person when trying to access sexual and reproductive health services?

R Actually for me at this time I don’t have any challenges because I always come here for anything. When I want to meet the doctors I just come to the facility and look for one of them and tell him/her my problem and they are able to assist.

I Mmmm so, you don’t have any challenge you have experience not necessarily now even before?

R There is no challenge

I Okay. How does being in school or college for young people like you affect their access to sexual and reproductive health services?

R You know when you are in school or college; you are fixed to a certain program even the school program. You have to be in school at that particular time. Sometimes you are in school and you wan to go for the sexual and reproductive health education, you cannot leave school and go. You are just there…..

I Mmmm..and even the services..

R Even services you cannot go

I Like maybe STD prevention and contraceptives services like family planning for females. Does that hinder them from accessing sexual and reproductive health information and services?

R Yeah, I think so

I Okay. What else while you are in school affect someone’s access to sexual and reproductive health.

R You know in school there is peer pressure. You want to do something so that someone sees you. Maybe you want to know more about sexual health yet there is your friend who tells you that those things are rumors they don’t help and you end up not going for the education.

I Mmmm

R Yeah…

I Apart from peer pressure-people influencing you not to attend while in school and also exams as you had earlier said, what else could make young person not access these services.

R Sometimes young people are always scared. One may be scared to express him/herself thereby keeping the things in his/her heart.

I Mmmmm. Any other thing you would like to add?

R Mmmm, no

I How comfortable would you be discussing your sexual and reproductive health concerns ith a lay female healthcare provider. Somebody maybe of your mothers age?

R I can just feel free to explain myself to her because you know the good thing and bad thing about it

I Mmmmm

R Yeah…

I So, will you be comfortable talking about your sexual and reproductive health needs to her.. it may not be necessarily your mum but at the clinic like a lay female healthcare provider. Some one mature, you know….someone of maybe your mother’s age. Would you be comfortable with such a person?

R I can just let it out.

I mmm, why?

R Because she may understand you better.

I Okay. When you talk of understanding you better what do you mean?

R You have said a clinician, someone at the hospital

I Okay, it may not necessarily be a clinician. When I talk of a lay person I do not mean he/she is a clinician. A lay person is one who is trained on a particular area and not a professional.

R She has been trained so she understands you. You tell her everything so that she may help you in any way.

I Would you be comfortable discussing your issues with them?

R Yes

I Okay. So, what issues would you be comfortable discussing with a lay female healthcare provider of your mother’s age in relation to sexual and reproductive health?

R You discuss with her the challenges you have been facing and she may help you out.

I Mmmm….. Which challenges?

R I cannot think of any challenges right now

I Mmmm maybe you can think in relation to sexual and reproductive health. What issues would you be discussing with this person?

R You could be taught on how to use condom (Pauses)

I What you are trying to say is maybe you want to know how to use a condom

R Yes, you know such things. How to put it on, how to remove…

I Mmm okay. What else?

R Only that…

I Mmmm, where would you want to have such discussions on sexual and reproductive health to take place?

R At the facility

I At the facility?

R Yes, at the county hospital

I Why at the facility?

R This is a favorable place because many people here understand you. You know this is the place I came to pick my drugs and it is the place where I come to pick my drugs and attend support groups

I Mmm okay. So you would prefer the facility?

R Yes

I If I may ask, and why not a t home?

R (Laughs….) I will not be comfortable

I Mmmmm, (Laughs..) Why wouldn’t you be comfortable?

R I can’t say….

I Just feel free and share..

R You know at home many things might happen

I Many things? Mmmm Like what?

R (Laughs)…..You might be sexually excited.

I Mmmmmm, so you would prefer the facility

R Yes

I Okay, I understand. We are on the final sections of the interview. I want us to look at your views on potential interventions. Things that can be done to improve access to sexual and reproductive health services among adolescents and young adults.

I How would you feel if we assigned you a lay female healthcare provider to be your confidant and source of information for your sexual and reproductive health.

R It’s a good idea…

I Mmmm what do you feel about it?

R Its good because she may be talking to me about sexual and reproductive health.

I Okay. What would be your ideal attributes or characteristics you would wan to see in such a person. That is the lay female healthcare provider

R She should be encouraging, jovial. You know there are some people who are angry and they do not know how to approach someone

I Mmmm

R Someone who is happy, understanding and also lets say my age.

I Okay. So you would prefer someone your age

R Yeah..

I Earlier on when I asked of someone who is of your mothers age, you were also comfortable with such a person

R Yeah…

I Okay. You have talked of someone who is jovial, encouraging, understanding……What other attributes would you want in such a person?

R That’s all I can remember

I Okay. What do you feel about receiving your DRVs together with your sexual and reproductive health services?

R It is good.

I Why do you say its good?

R You don’t have to create time for going for ARVs and also reproductive health. You do them at the same time

I Great. What else?

R Only that..

I Okay. What would be the advantages and disadvantages of receiving ARDVs and sexual and reproductive health services at the same point?Maybe you start with advantages?

R Okay, when you come for your ARVs you will be taught on what to do and what not to do. Now you go home thinking about it and also you will receive reproductive health information and you have to digest it……

I Any other advantage of receiving these services together?

R They go together. You know when you do not take your drugs the virus in your body increases and if you do not have sexual and reproductive health knowledge you may spread the disease to other people.

I Mmmm

R Yea..

I Okay. Do you think there are disadvantages of receiving your ARVs together with the sexual and reproductive health services together at the same point?

R Receiving ARVs and sexual and reproductive health services together? At the same facility?

I Yes

R I don think there are disadvantages

I Okay, so you don’t think there are disadvantages/

R Yeah.. You know if you do not get them at the same point. You know you have to move from one place to another and it is tiresome. The advantage of getting them at the same place is that you benefit twice

I Mmmm.. that is good. What other advantage?

R I don’t have another advantage

I Okay. Thank you very much for your participation we have come to the end of our discussion. Do you have any questions, concerns or clarification in relation to sexual and reproductive health?

R I just wanted to emphasize… Like when we come to the clinic during your appointment. Sometimes you come the first person and you sit waiting for almost 2 hours and someone who cam much later is being attended to. You feel irritated.

I Mmmm so you would wan the facility to be aware of this?

R Yes

I Okay. What about in relation to sexual and reproductive health?

R No, only that.

I Okay, I will inform the facility about it. Thank you very much

R You are welcomed.

**AYA IDI: KCH 004**

**Site: KCH**

**Date: 20 May 2019**

Age: 24 years

Sex: Female

Level of education: College

Occupation: Employed

Orphan hood status: Partial

Sexually active: Yes

Marital status: Single

Have you ever become pregnant: No

I: I would like us to start. We are going to have three sections that we will go through during this interview. We will look at the general challenges experienced, HIV care services and wind up with sexual and reproductive health services and your views on potential interventions.

What are the challenges you face as a young person

R: As per adherence or just general

I: Generally, what are the challenges you face as a young person?

R: Mmmm challenges may be having friends… Like this person is my friend but you cannot communicate.

I: Mmmhh so communication is a challenge

R: Yes,

I: How? May be I need to understand how this communication is a challenge

R: Maybe someone is telling you like.. “Can you come to such a place?” I am an introvert, I don’t like going out..

I: Mmm okay. So you find it challenging

R: Mmmm, again saying no and this person is your friend.. Its very difficult

I: Okay, so that is the challenge you are facing?

R: Yes, So its like I will just go but I really don’t feel like. I will go so that I don’t disappoint him/her

I: Mmmm okay. Any other challenge you think young people face?

R: Peer pressure

I: Ahaa, tell me more about it

R: Doing what they want to do

I: Maybe an example?

R: Aaaa, like drug abuse.

I: Mmmmm, drug abuse. As a person have you experienced issues to do with drug abuse ?

R: No

I: Do you have friends who abused drugs?

R: Yes, it it really drug abuse? Mmmm, they just use it for fun but they are my friends and there is nothing I can do

I: Which drug are they abusing specifically?

R: How do we call it in English? Oooh, they like smoking weed.

I: Okay, how is that a challenge?

R: It was challenging when I was in High School. I used to stay in a single room

I: Single room or dormitory or a cube?

R: A cube. So they were like, J…..(name withheld) we are coming to your room

I: So they used to come and smoke weed in your room?

R: Yes, since they were my friends I could not prevent them from coming. I could tell them to just come then I leave

I: Mmmm, was it a boarding school or?

R: Yes, it was in College, KMTC

I: Sorry, it was in college and not High school

R: Yes, KMTC

I: Oooh, okay. Do you think these challenges like you have listed-peer pressure, dealing with drug abuse..

Do you think these challenges affect your educational and professional achievement?

R:Mmmm, they didn’t really affect me because I was not into them.

I: Okay. And any other challenge you could face, not necessarily when you were in college but even now when you are working

R: I don’t know how I will call that.. Like most of my friends are married and they have children. They keep on asking…… (Name withheld) you are left behind..(Laughs)

I: (Laughs) mmmm

R: They pressurize me and I am not ready.

I: Okay. Does that affect you in a way?

R: Mmmm…I cant even call them because they will tell me that my baby this…..my baby that….

I: Okay. It affects you emotionally if I get it right from what you are saying.

R: Yees

I: Does it affect your socioeconomic advancements? Does it affect your work?

R: No

I: Okay. So, how have you tried to deal with these challenges?

You have talked of not being able to communicate, peer pressure, your friends are getting married and now they are pressurizing you like (Name withheld)…… what are you waiting for?

How have you tried to deal with these challenges as a person?

R: For communication, I have just been saying I am busy and if I look at what has made me busy, it nothing actually.

I: Apart from being busy, how have you dealt with other challenges?

R: I just let it go

I: Mmmmm, it doesn’t affect you in any way?

R: No

I: Okay. To what extent have you succeeded in dealing with these challenges

R: Nothing much….

I: Okay. Do you feel like there is support available to help you deal with the challenges?

R: Mmmmm There are support groups like the ones being held at KCH its only that I never attend because the time they are scheduled I am supposed to be at work.

I: Mmmm, apart from these support group. Do you think there is any available support that helps you deal with your challenges? Not necessarily the support groups here

R: I deal with them myself..

I: You deal with them yourself?

R: Yes

I: How?

R: I let it go and I tell myself that my time will come

I: Mmmm. Okay. That’s a way of encouraging yourself that your time will come….

R: Yeahh

I: You don’t have any other support system like friends who could support you, relatives….

R: In terms of friends, when I feel like I want to go out I call them. Since they like going out, they will gladly agree. So I go to have fun so that I can clear my mind and be stress free

I: Okay. I will use an example of the challenges you earlier mentioned. When your friend calls you and asks what you are waiting for yet they have children and they are married….. and what you are waiting for…….

To get through that, do you have people you talk to as your support system, who can encourage you?

R; Currently no

I: Mmmmm okay. We will now proceed to HIV care services

I: I would like you to describe to me the day you were enrolled n to HIV care and services

R: I was enrolled in 2010 while I was in Form 1

I: Mmmmm. What was your experience or rather how was it?

R: It was not really a nice experience for the first time mmm I think for the first few months. I guess I was just a strong girl.

I: Mmmmm

R: And then my High school life was good. I talked to my class teacher about it and she used to encourage me a lot. So I didn’t have the experience of things like stigma

I:Mmmm

R: If someone asks me how I was taking my drugs in High school.. I cannot really explain because idid not have that feeling like someone is going to see me neither did I experience stigma

I: Mmmm. Okay

R: My class teacher was just too good.

I: Okay. That is good. If I may ask, who accompanied you for the enrollment to HIV Care services?

R: My dad

I: Your dad?

R: Yes.. my dad.

I: Okay. Maybe you can tell me, how did you come to learn of your HIV status?

R: Mmmm I became sick in Second term Form 1. So, I went back home. My dad had a friend here in hospital called Dr. (Name withtheld). Since he is experienced, he knew that it could this.. or that. He brought to the PSC the other side and they have experience they know how it manifests…

I: Okay.. So, how did you feel on that specific day?

R: It wasn’t good. I really cried but again at the end of the day even if I cry it is there already. I am in Form 1 and life has to continue..

I: mmmmm okay. What was going on in your mind at that time?

R: What would people think? I have never taken these drugs, I just hear about them..

I: Mmm what made you decide that I need now to enroll for HIV care services?

R: I think I just decided immediately. After the confirmation that I was positive, I was taken for adherence session for 3 days then the last day I was given medication.

I:Mmmm, okey.

R: That is how it started

I:Okay. What has enabled you to continue engaging in care at this facility?

R: I have a little things to achieve.

I: Mmmmm, Maybe I can ask again. What has enabled you to continue engaging in care, Is it the distance, the type of service you receive, is it the staff that are here?

R: It’s the type of service and the staff. Again changing from one place to another is like starting a new life.

I: Mmmmm

R: It will be such a struggle to get used to those other people

R: Mmmm okay. Then now you get to know where you are supposed to go. Like maybe Russia, no let me not say Russia I have been there before. Lets say a place like Lumumba. If I transfer to Lumumba I will start asking where Lumumba PSC is…

I: Mmmm okay. So, you are saying what has made you to continue engaging in care here is because you are used to this place

R: I am used to the staff

I: Mmm, how is the service provided here?

R: The service is good

I: Staff?

R: The staff are good as well

I: When you say good, what do you mean? You can elaborate

R: How they receive you mmmm

I: How they handle you?

R: Mmmm yes

I: Could you please tell me instances you have experienced stigma or been discriminated against because of your HIV status?

R: I have never experienced stigma

I: You’ve never experienced that?

R: No, that’s why I told you that my life has just been a normal one

I: Maybe just to jog your mind a little bit. Do you think there are people who have experienced stigma?

R: Yeah

I: Do you know people who have experienced stigma?

R: No

I: Okay. Tell me what you know about ARVs

R: ARVS? (Laughs…)

I: (Laughs) Anything you know about them

R:In laymans or …(Laughs)

I: Anyway you would love to put it

R: ARVs are these drugs that fight the virus

I: Mmmmm, they fight the virus

R: So that you have a suppressed viral load

I: Mmmmm

R: With a good CD4 count

I: Mmmm, any other thing

R: They are big… Very big (Laughs)

I: Mmmmm

R: lets say like when you have a sore throat..

I: Throat infection

R: Swallowing it is very hard

I: Ooooh

R: Yeah, it doesn’t move down. It gets stuck here (touches the throat)

I: Mmmmm

R: And then it starts to corrode

I: So you do not swallow?

R: You just have to take a lot of water to enable you to swallow

I: Mmmm okay. So, they are all big

R: Yes, all those I have seen are big and the number of times you have to take…… Its everyday

I: Mmm every day.. Okay.

R: And forgetting a day is a mess. So you cant forget, you just have to take them daily

I: What do you mean by it’s a mess?

R: Its like you have woken up the viruses

I: Aaah okay. Any other thing about ARVs?

R: Mmmmm no.

I: Okay. Telll me about the time you missed taking your ARVs, what happened?

R: The time I missed, I didn’t take it. I took it the next day.

I: What happened till you missed?

R: I think I overslept.

I: Was it morning or evening?

R: Morning

I: Ahaaa..At what time do you take your medication?

R: At seven

I: Then what happened?

R: I just felt like sleeping a little more when it was time (Laughs..)

I: Mmmm

R: Then in the process of catching some sleep, I woke up at 9.00am

I: Mmmmm

R: This thing should be within +1 or -1 hour. Past 2 hours? Let me just wait for evening and tomorrow morning

I:Mmmm okay. What did you do after you had missed your drug?

R: I did not do anything. I was just waiting for evening

I: Okay. And have you ever missed again?

R: No

I: Okay. It was just a one time thing

R: Yes, it was just once

I: How did you feel?

R: It was long ago and I was like, what is going to happen to me

I: Mmmm

R: Yea

I: Okay, great. Could you please share with me the challenges you face keeping your clinic appointment?

R: Like now I have not arrived at work. I am here…

I: Ahaa, so at times your work interferes with your clinic appointment?

R: Yes

I: Okay. What other challenge?

R: There is no other challenge. Only that

I: Only that..

R: Yes

I: Tell me about the last time you missed your clinic appointment.

R: I have never missed my clinic appointment

I: Really?

R: Yes, my dad was a very strict person. You could never miss your appointment.

I: When you say your dad was strict, is he still alive or?

R: No, he died

I: Sorry about that. So, he used to ensure you attend your clinic.

R: M,mmm, in High school when fellow students could just see him, they be like “J…… your dad has arrived”. Everyone knew my dad has come to school but they did not know why

I: Mmmm, so he could take you to the clinic? Was the clinic near school?

R: No, I had to come here. He had to bring me. In Kisumu Girls.

I: Mmmm

R: But when I was at KMTC, he could come to the clinic pick the drugs and send to me as a parcel.

I: KMTC which place?

R: Nairobi

I: Nairobi aaah

R: Yea

I: So, you’ve never missed your clinic

R: I have never missed my clinic

I: Okay. Even now when your dad is not around you still maintain your clinic visits?

R: I still maintain. That is what he instilled n me. You cannot miss

I: Okay. What are your general feelings about the HIV care services you are currently receiving

R: They are good, they are nice… The services are friendly and now the partitioning of the youth friendly services and the other one (points at the CCC)

I: The other one you mean the man clinic?

R: Yes. So, you get like youths were not coming. If you mix them with the other people they don’t come. They fear

I: Mmmm. So having this youth friendly centre is a good thing.

R: Yeah, it’s a good thing

I: Okay. Any other thing you feel about the HIV care services that you are currently receiving?

R: (Pauses) Just that.

I: Just that. So, how do you think your encounter with HIV Care services can be made more satisfying?

R: Repeat the question

I: How do you think your encounter with HIV care services can be made more satisfying?

R: Already we have the youth friendly center, it is satisfying already

I:Mmmm, good. What else?

R: The activities they carry out at the youth friendly center

I: Mmmm Like which ones?

R: The support groups they have makes the youths come together and feel like I am not alone, we can do this together. When everybody is here they share their experiences

I: Mmmm, that is good. Do you participate in any of those activities?

R: I used to..

I: But now you don’t?

R: Time. I used to participate but when I have time I come here…..

I: Okay, that is great. Any other way you think the services can be made more satisfying? Apart from support groups and sessions where people share their experiences

R: There is a program where they go to schools and enlighten people

I: So, that is something you are proposing or its something that is happening?

R: Its something that is there but I don’t know which group. A friend of mine is engaged in that.

I: So, you feel its something that should be on to make the experience more satisfying

R: Yess.

I: Okay.

R: Lets just say like by bad luck one who had been enlightened then later discovers that he/she is positive, you wont feel like it is over

I: Mmmm

R: Because she/he has received a lot of information

I: That is good. So, how does being n school or college for young people like you affect their keeping clinic appointments and taking ARVs?

R: On my part, I don’t know what to say because mine were being brought. When my time comes, I am told they are on the vehicle and they will arrive at such a time and I go and pick.

I: Okay. What about others, what do you think?

R: The challenge is , like at KNH when people have gone for ward rounds or a friend of yours is at the pharmacy and you are expected to go, You will not because they will feel like somebody is going to see you.

I: Mmmmm

R: You wont go, and that time is class time…You will have to wait for him/her to leave and you will have wasted time.

I: Mmmm, any other thing you think can affect young people’s clinic attendance and taking ARVs while in school or college?

R: That one now I don’t know how it can be corrected because attachments, internships have to be there

I: Mmmm What else can affect young people’s clinic attendance and taking ARVs while in school or college?

R: While in college, Fridays people like going to clubs and then lets say like me I take my drugs at 7am/7Pm just in case I found myself somewhere at 7 and I didn’t carry drugs, I have to start telling my friends that I am leaving….

I: I will be asked where I am leaving to and we haven’t finished? You will be forced to stay even though you feel like you should leave to go and take medication

I: What else?

R: I told you like somebody will see him or her

I: You fear being seeing, you have gone out with friends and your time for medication has arrived. You really feel like you should go….

Ahaa.. what else?

R: Lets say like you went out with your friends and you carried the drugs, your time for medication has reached and you are enjoying yourselves, how are you going to excuse yourself to take them? That another challenge

I: Great. We are doing well. We are moving on to the next section on sexual and reproductive health.

R: Mmmm

I: What do you understand by the term sexual and reproductive health?

R: Being sexually active

I: Mmmm, what else

R: Intimacy

I: Mmmm, what else? May be things to do with access to reproductive health information, safe sex, contraceptive/family planning, things to do with STI and STD prevention and such like…

R: Mmmmm

I: How important is your access to sexual and reproductive health services?

R: It is very important because I have to be told more about contraceptives, these STDs and STIs

I: Mmmm, okay. So where do you currently access information on sexual and reproductive health?

R: I like the internet

I: Ahaaa… you like the internet. Apart form the internet where else do you access that information?

R: When we are free with friend we really like talking about it

I: Ahhaa with your peers?

R: Yes

I: Okay. Where else do you access information on sexual and reproductive health?

R: At the hospital

I: So, what are you taught at the hospital?

R: I have never really attended sessions to do with contraceptives but when I come for my clinic the clinician always asks.

I: Ahaa, so you get nformation from the clinician

R: Mmmmm

I: Okay. Where is the ideal location you would like to access information on sexual and reproductive health?

R: Location?

I: Yes location

R: Here at the facility

I: Here,here… Why?

R: I just love KCH

I: Why do you love it, I am sure there is a reason as to why you love it

R: (Laughs…..) The people I meet here at KCH are quiet friendly.

I: Mmmmm, apart from being friendly what makes you love this place?

R: How it is structured. When I want to come for family planning, I know where to go. Like where we are now

I: Mmmm, not the adults side

R: No

I: Okay. To what extent does the sexual and reproductive health services offered to young people like you meet their needs and expectations?

R: (Pause….) Yeah…

I: To what extent?

R: Its 100% to get your responses on what you want like contraceptives. They are very committed

I: Is it only contraceptives or other services too?

R: Other sexual and reproductive health services too

I: Okay. What challenges do you as a person face when trying to access sexual and reproductive health information and services?

R: I haven’t attended any session so I cannot say if I have a challenge or not

I: Mmmmmm. But, do you think there are challenges that you can anticipate if you decide to come for sexual and reproductive health services?

R: Yes, because you maybe you might get deeper in to conversation and you are asked if you have a boyfriend and if your boyfriend know their status…. Those ones I don’t know

I: Mmmm

R: What will I answer? Where is he?

I: Mmmmm. So you feel that will be challenging to you?

R: Yeah…

I: Okay. How does being in school or college for young people of your age affect their access to sexual and reproductive health information and services?

R: I think it’s time

I: Time. Mmmm

R: You are in class and you have to explain where you are going….

I: So, you think time really affects their access? What else?

R: Yes. Maybe if they go to a clinic within the school and the person to offer the service is not there…

I: So, within the school?

R: Yes, within the school

I: Mmmm, what else?

R: Also if you go with a friend and they ask you why you are going for those services….mmm

I: So, you are trying to say that your friend is a barrier?

R: Yes, your friend might be a barrier

I: Mmm

R: You know there are somethings you can do alone but others you cannot when you have a friend

I: Mmmm okay. How comfortable will you be discussing your sexual and reproductive health concerns with a lay female healthcare provider, someone of your mothers age?

R: I will just tell her what she needs to know… (Laughs….)

I: (Laughs…) Like what? What does she need to know?

R: (Pauses…)

I: Maybe you have a challenge… How confortable will you be to discuss with her?

R: Not quiet comfortable

I: Not quiet comfortable.. why?

R: Like when you have a STI, how are you going to begin explaining that you have a sexually transmitted disease to an elderly person

I:Mmmmm

R: Maybe she won’t understand

I: So, you would prefer your peer?

R: I would prefer my peer

I: Not someone…. A lay female healthcare provider probably of your mothers age or there about?

R: Maybe she wont understand what I am trying to say and then she will be like…. My daughter you said like this.. and that (Laughs…..)

I: Okay…… What issues will you be comfortable discussing with this lay female healthcare provider?

R:About sexual and reproductive health?

I: Yes

R: Like I am having a STI? What do you think….. HIV is there so she know. What she knows is what I will make her hear

I: Mmmm it not just about STI, maybe you would want something like a condom demonstration. Would you be comfortable discussing with her? Or which person would you prefer

R: Maybe lets say a nurse, a clinician or my peer

I: Mmmmm maybe that clinician is your mothers age….

R: It depends on how we relate….

I: You don’t prefer a lay person?

R: Mmmm no

I: Okay. Where and why would you prefer those discussions to take place?

R: Between me and the person?

I: Yes..

R: At a confidential place

I: A confidential place like where?

R: A room where it is just me and her

I: Okay, a room in a hospital, school, home or where?

R: Whichever place…. If its at home it is okay or evenhospital because we will just be the two of us.

I: Mmmmm

R: But if in school….. I t will be like why her? What are they discussing?

I: Mmmmm. So, you prefer either home or hospital?....

R: Mmmmm yes.

I: Okay. We are winding up. I want us to discuss your views on potential interventions. How would you feel if we assigned you a lay female healthcare provider to be your confidant and source of information for your sexual and reproductive health?

R: A layperson?

I: Yes

R: I think it will just work because I will make her understand

I: Mmmmmm, so, you will feel comfortable or you will be uncomfortable. How would you feel?

R: It is a mixed reaction… (Laughs….)

I: Mmmmm,

R: Maybe you can explain to her then she understands and oyu will be comfortable but also she may not understand.

I: So you have mixed feelings about it

R: Yea. A layperson may not understand everything so you really have to explain yourself

I: Mmmm okay. So what would be your ideal attributes or characteristics of such a lay healthcare provider to act as your source of information on sexual and reproductive health

R: Can I confide?

I: Someone confidential?

R: Yes. Will she understand what I tell her?

I: Mmmmm

R: Because if she doesn’t, we will not communicate

I: What other attributes would you like in this person apart from being confidential, understanding,

R: Friendly

I: Mmmm friendly… What else?

R: Just that

I: How would you feel when you receive your ARVs together with your sexual and reproductive health services?

R: It will be good because you just come once

I: Mmmmm

R: Like when I come for my clininc, I also attend the session then just leave at once

I: Mmmmm any other thing?

R: HIV and reproductive health go hand in hand

I: Mmmmmm

R: If you are sexually active then you go to the clinic you will also be issued with condoms and you will practice safe sex and there will be no reinfections

I: Mmmmm what you are trying to say is that if you receive them in one room you will just come once so you don’t have to come another time.

R: Yes…

I: What do you think are the advantages and disadvantages of receiving your ART and sexual and reproductive health services together at the same point?

R: Time

I: What about time

R: I don’t think you will take a lot of time.You don’t have to come for your clinic today and plan to come another time for another service

I: Mmmm. Any other advantage?

R: I think of you are getting them at the same point maybe you finish your clininc session then you will get other people waiting for the service then you will be there togehter

I: Mmmm, okay. Do you think there are any disadvantages of receiving your ART together with the sexual and reproductive health services at the same point?

R: There might be….. You may not have wanted to have a session on sexual and reproductive health but if they are now offered together, you will be forced to.

I: Mmmm, okay. Any other disadvantage?

R:No

I; Do you have any questions in relation to what we have discussed?

R: Question?

I: Yeah, any in relation to what we have discussed?

R: Taking the drugs everyday is a challenge and they are very big. Also coming to the clinin frequently is also a problem.

I: What would you suggest?

R: If someone is taking TDF, 3TC and Lopinavir, you know they are a lot. So maybe if there could be combinations of those ARVs so that you take them at once…

I: Okay. I will pass the information to the clinic staff. Is that okay?

R: Okay.

I: Thank you very much for your time

R: Mmmm okay.

**AYA IDI: KCH 006**

**Site: KCH**

**Date: 20 May 2019**

Age: 23 years

I: I would like us to begin our session. What are the challenges you face as a young person

R: Challenges are so many. One is lack of money, these break ups…

I: Mmmm, break ups?

R: Break ups as in when you are in a relationship with someone then you part ways.

I: Mmmm

R: Okay. That is the worst challenge I have ever faced and I learnt a lot

I: Mmmm, could you please tell me more about it?

R: It happened 3-4 days ago. I had a girlfriend, she was called P…..(Name withheld). We used to do family planning and I was the one who was paying. Suddenly, we quarreled over some issues. She wanted me to send her money and since I did not have, she started insulting me. I tried to talk to her but she decided to leave. I begged her but she refused. That is how life is….

I: Okay, I ‘m sorry about that. How did it make you feel?

R: I felt desperate and knew that no one in this life should be trusted. No one is there to stay, they come and go

I: Mmmm okay. That’s one challenge you have had-break up and you say it a major challenge you have had as a young person.

R: Yeah, I don’t see any other challenge because I have been living with this thing. It is not a challenge because I am the one who made my family know that this thing was in the family.

I: When you talk of this thing what do you mean?

R: HIV

I: Mmm, okay.

R: I was the first person to become sick in my family. I was taken to hospital admitted at the ICU for like month. After being discharged, I used to be brought back every week to the hospital for treatment but they never knew I was positive. My grandmother decided that my two sisters and I, we are three and I am the first-born should go to the hospital. So we went to Tuungane, I was tested and found positive. And my two sisters too were found to be positive and we were counseled at that time. I knew this was something that was in my blood and I knew it was the one that was killing me. Recently, I buried my aunt and after that I got stressed and that is what made me even become sicker. I loved my aunt very much such that when she died I started becoming sick.

I: Mmmm…

R: I was sick for quiet sometime and was retested after 3 years. My sisters and I were then initiated on ART. My father and my mother also went and tested positive. So that means it is something that had always been there. Let me say like I am the one who mad the family aware. It has not been a challenge to me

I: Mmmmm okay. We will come back to that. So, generally you have talked about break up and how it made you feel. Another challenge you told me you face as young person is lack of money. Would you please tell me more about it?

R: When you need something you find it hard. When you try to ask someone, no one is giving you so you have to come out to go and look for money. So its like that. I am waiting to join college in September so for now money is a problem.

I: Mmmm, okay. So, apart from money, break ups what other challenges do you face as a young person?

R: I am not facing any other challenge.

I: Do you think these challenges you have mentioned like break ups, money issues have affected your education, professional life or your socioeconomic advancements in a way?

R: They have made my financial (Pauses) Like, I needed money to start a business but I have not been able to. I wanted to start a smokies business but I never got money to buy the grill. Also a stationery business but I never go money . So that’s how it has….

I: Mmmm, so it has affected you in terms of your economic advancements and achievements. How about your education, has it been affected?

R: No. My education has been successful because my dad has always supported me all the way.

I: Okay. As a person, have you tried to deal with these challenges?

R: Yes

I: How?

R: I am trying to cope because my dad has also experienced this. I was not doing anything and my dad saw the passion in me. I needed a job, so I went for another job but it was not fitting me. I left and went back to stay at home. My father saw that I was not happy, he took me to his business to help with the selling and he gives me 200/= per day or 300/= per day. So it depends…

I: Okay. To what extent have you succeeded in dealing with these challenges?

R: The extent is like….. Mmmm my mum is always there for me and when I need anything shen can give me.

I: Mmmm. So you feel you have succeeded in dealing with these challenges?

R: Yees

I: Mmmm, what about the break up? How did you deal with it?

R: I dealt with it. You know this girl left without me playing her we just had a quarrel. So, she had a friend, a very close friend.

I: Female or male friend?

R: Female. Her friend liked me but I never knew. So, the moment we broke up, she came and told me that my ex-girlfriend told her that if she dates me then their friendship is over. So they are no longer friends because of me yet the girl has insisted we continue with the relationship.

I:Mmmmm, okay

R: So, the other feels betrayed and the friend insists we continue with the relationship. That is how I overcame it

I:Okay. What support is available to help you deal with these challenges you are facing?

R: Support?

I: Mmmm

R: My mum, she is the support I can think of.

I: Tell me more …

R: She loves and cares for me, watches my moves, talks to me about my status and God… Story to do with church

I: Mmmm how does the church comoe in your support?

R: She has introduced me to groups, the choir, church youth programs. Those are the things that keep me busy. When I did not have a job and I was home, I could go to church sing, discuss with people then go back home.

I:Where do you go to church?

R: St Paul’s Parish

I: So you have these groups within the church?

R: Yes

I: Okay. That is good. We are moving to the next section and will be talking about HIV care services. You had earlier talked about how you came to know about your HIV status, how you got enrolled to HIV care and I appreciate that. If I may ask, who accompanied you to when you were going to be tested for HIV?

R: My grandmother

I: Okay. Did this happen after your admission in the Intensive Care Unit or before?

R: Yes, it was after admission but my whole family was healthy. I was the one who was always sick. After the admission to ICU, I become well and was discharged. They used to pray for me to get well. Two weeks later, my body turned yellow and was rushed back to the hospital, got transfused and later discharged after my condition had improved. I used to get admitted after every week because the blood levels in my body was very low and had to be transfused. I then developed TB at some point malaria and another disease I have forgotten the name where my urine used to be bloody.

I: Mmmmm

R: So, my grandmother belongs to a certain group in one of the hospitals and they do advocate for HIV testing. I think she wondered what was happening to the grandson and thought I should be tested. She discussed with my parents and they agreed, however my mother did not want us to be tested. She was very hesitant. My grandmother insisted and my two sisters and I were taken to Tuungane Youth Center for HIV testing. We all tested positive and we were counseled. I wasn’t afraid though. I was supposed to be initiated on ART but since I was on TB treatment, I was advised to finish them first before I could start the ARVs. I continued taking the Anti-TBs for a month then went back to the hospital where I was initiated on ART. Taking the ARVs was challenging, day 1 I was having very bad dreams

I: Mmmm

R: I was dreaming of my aunt who had died. This went on for close to one week then stopped. My body was now adjusting to the drugs and up to now I am used to taking the drugs such that if I don’t take I don’t feel normal.

I:Mmmmm Just to take you back a little. At what age did you discover you are HIV positive?

R: (Pauses) I was in class 4

I: That’s about 9 years old or there about

R: Yeah

I: Okay. At that time when you were being initiated on ARVs, what was going on through your mind?

R: I was young then but I could understand. There was nothing on my mind but I was told that I will be taking drugs every day. I was used to taking drugs because I had been sick for 3 years. I wasn’t scared, and was even told that if I leave taking the drugs I will die. I told them I couldn’t stop taking the drugs because I was used to taking drugs.

Its like I was addicted to taking drugs.

I: Mmmm okay. Thank you. What has enabled you to continue engaging in care here at this facility?

R: I have been in Tuungane and it was a good place as it was somehow a private clinic. We were then moved here but it was an open place. We were still there (Points at the main support center for adults) and had not moved to the adolescent and youth friendly center here .It was an open place and it was challenging to me because I was afraid. I used to miss my appointments because I still had some drugs and could only come when they are completely over.

I: Mmmmm

R: Moving here was a good thing because we were now not mixed with the adults, its somehow private and when you come for your drugs nobody knows what you have come to do.

I: Mmmm, so what has enabled you to continue engaging in care here is the privacy?

R: Yes, this building. I wanted to move to KDH but since they built this adolescent center I felt it was now okay to stay.

I: You wanted to move to?

R: Lumumba

I: Okay. What else keeps you to engage in care here?

R: Like here, what has really encouraged me in my journey with HIV is the love this people have for us. They create for us support groups, camps etc. Like we went for camping for a whole week and I really liked it. It made me meet and get to know many people. Very beautiful girls are taking ARVs and many people whom you cannot imagine are taking them. This encouraged me because I am no the only one taking this thing.

In the support groups we are also taught on OTZ-adherence to drugs, and zero CD4 count. It has been good because we are taught and also encouraged at the same time.

I: That is good. Could you please share with me any instances you have experienced stigma or has been discriminated against because of your HIV status?

R: Oooh no, I haven’t faced stigma. Actually, I was almost facing it but I can say I was smart while in High school.

I: Mmmm tell me more

R: I joined a boarding school while in Form 2. I was taking my drugs but did not disclose to any of the teachers. I used to keep my drugs in one of my bags in the box and nobody could tell I was on drugs for more than a year. During my second year in that school, I disclosed to our Guidance and Counseling teacher. I used to go the clinic to get my drugs but could lie that I am not feeling well. When I disclosed to her, she did encourage me and told me not to despair and continue taking the drugs secretly as I used to. I had a good friend called R….. whom I disclosed to about my status. He was very supportive and could always remind me when it was time to take my medication. While doing my final exams in High school-KCSE, I had kept my drugs inside my bag in the box and one of the students stole it. I knew the person who had stolen because he had been expelled from school because of theft and was just coming on a daily basis to do the exams.

I: Mmmmm

R: We informed his father since he was also working in our school. He was told to return that bag. I was worried because he probably had seen those drugs and would tell everyone. The good thing was that that was the last day we were doing the exams and he returned it with the drugs inside but the bag was interfered with. He came and told people about my status but luckily I had just left the school after my final paper. I didn’t experience the stigma though and I have lived all a long with them in school and they had never known my status. I used to laugh with them, ask for relationships with girls while with them but nobody ever knew of my status. You know when there is stigma you do not have courage so that how I left High school without them knowing and experiencing stigma. After we finished school, there was one of my schoolmates who sent me whatsapp message that they knew about the secret-that I was taking drugs just before I left school. I told him that I didn’t care and he is stupid and the world will teach him. I told him to mind his business

I: Okay. Tell me what you know about ARVs

R:ARVs?

I: Yes, Antiretroviral drugs.

R: ARVs are drugs that if you leave, you will die.

I: Mmmmm

R: I have seen so many people who have stopped taking drugs and are not doing well. They are in and out of hospitals. If you stop you can get diseases you know not of their origin. I cannot stop taking those drugs and they are my life.

I: Okay. Any other thing you know about these ARV’s?

R: Side effects?

I: Mmmm,

R: I have not experienced the side effects.

I: Okay. You have told me ARVs are your life. What do you mean?

R: If I leave it I will die. That is what I can say.

I: Mmmm. Okay, tell me about the time you missed your ARVs

R: That day when my bag was stolen with the drugs inside I slept without taking them.

I: Okay. So, what did you do? The bag was gone, no drugs?

R: Its like I missed almost thrice but I didn’t experience any side effects. I was stressed but encouraged myself that once I get my bag, I will take them.

I: Kindly share with me the challenges you face keeping your clinic appointments

R: I have no challenges

I: Mmmmm okay. You keep your clinic appointments as expected?

R: Yes

I: Okay. What are your general feelings about HIV care services you are currently receiving?

R: I am okay with the services. I have no challenge. Our facility is good, we usually have fun together as youths, we win trophies when we compete with other facilities. Everything is just fine

I: Okay, so you feel everything is good.

R: Yes, everything is good.

I: Mmmm, what do you mean by everything is good.

R: Everything. You see when you come here you cannot be idle, you come you are given your drugs in one of the rooms and you go home happy. We are treated well

I: Mmmmm, how do you think your encounter with HIV services can be made more satisfying?

R: Here?

I: Yes…

R: We need a TV at the waiting bay. That is what is missing here. In Tuungane Center they used to have one

I: Mmmmm, for your services to be more satisfying you need a television?

R: Yes.

I: What else

R: Camp

R: We need a camp where we go and stay. There is one we had in Kisii organized by the Kisumu team even though at that time I was still taking care at Tuungane. People from Ahero came and some of them I knew because were studying together at Ahero and some of my friends too. So we need more camps like those where we spend at least 1 week. It was fun…

I: Mmmm, you got to learn and interact with people?

R: Yes, we learnt a lot and meet with people from different places.

I: How does being in school or college for young people like you affect keeping their clinic appointments and taking their ARVs?

R: Pardon

I: How does being in school or college for young people like you affect keeping their clinic appointments and taking their ARVs?

R: I may not be able to tell that because I have not joined college. I haven’t experienced any challenge even though I was in a boarding high school. As I earlier told you, I could just take my medicine without anyone knowing.

I: Okay. But, do you think other people could face challenges with keeping their clinic appointments and taking ARVs while in school?

R: Yes, some are afraid they will be seen. That is the major challenge

I: Mmmmm, who would see them?

R: Someone you know even your neighbor will see you taking the drugs or something like that.

I: What other challenges would they face apart from knowing that so and so could be seeing me

R: You know, if you carry the drugs in the bottles they shake. If you have them in your bag you will feel uncomfortable if there is movement with the bag.

I: Any other challenge?

R: Only that.

I: How about clinic attendance?

R: I don’t think it is a challenge because one can ask for permission to come and take the drugs and they will not be denied. I used to attend my clinic as scheduled.

I: Great. We are dong well. We can move on to discuss matters sexual and reproductive health.

In your own words could you please tell me what you understand by the term sexual and reproductive health?

R: That deals with testing for STD and STI and ensuring hygiene of the sexual organs

I: Any other thing in terms of contraceptives/family planning, condom use… Do they also form part of sexual and reproductive health?

R: Yes

I: Mmmm, even STI prevention among others.

R: How can family planning keep health?

I: When we talk of family planning we do not define it as keeping health but we talk of use of medication or other devices to prevent pregnancy. Family planning only prevents pregnancy but not infections like STI among others.

R: Okay.

I: How important is your access to sexual and reproductive health information and services?

R: I have no idea

I: Mmmm, you have no idea

I: Maybe I can ask you in a different way. Do you currently access sexual and reproductive health information and services?

R: Yes,

I: Where?

R: Here…

I: At the facility

R: Yes.

I: Okay. Any other place you can access this information?

R: In school but now I am not in school

I: So, in school who could teach you?

R: Those people who use to come to schools to mobilize for circumcision could teach us and even our teachers.

I: Where do you currently access information and services on sexual and reproductive health?

R: Support groups, at the facility.

I: Mmmmm. So which services do you receive here in regards to sexual and reproductive health?

R: We receive male condoms. That is what I actually receive.

I: Maybe to just take you behind, you talked about a break up in your relationship with your girlfriend who was pregnant and you said you used to pay for the family planning. Where were you getting that service?

R: We used to buy from a certain pharmacy next to our home.

I: Which specific method if you can remember. Was it a a tablet or an injection, or what?

R: It was an injection

I: Okay. So you could take your girlfriend for the injection

R: Yes

I: Okay. What is the ideal place/location where you would like to access sexual and reproductive health services?

R: Jusr here..

I: Just here at the facility?

R: Yes, at the facility.

I: Why here?

R: There is privacy and confidentiality here. Someone may not know what you have come to do

I: Great. To what extent are the sexual and reproductive health services offered to young people like you meet their needs and expectations

R: Pardon

I: Do the sexual and reproductive health services offered to young people meet their expectations?

R: Yes, they do.

I: Mmmm, how?

R: Like trust condoms that hey receive prevent infection and pregnancy and also the family planning they receive prevent pregnancy.

I: Okay. What challenges do you as a person face in trying to access information and services on sexual and reproductive health?

R: No, I don’t face any challenge.

I: Okay. So you feel you do not have any challenge or you have not experienced any so far

R: Yeah. It is just normal to have things like condom burst which can be a challenge, but again that is just carelessness because we have been taught how to put it on here at the facility.

I: Has it happened to you?

R: Yes

I: How may times?

R: Once with that girl who became pregnant

I: Okay.

R: It was an accident

I: It was an accident.. Okay. How did you address that challenge?

R: The challenge is still there. The girl is insisting I support her but my mother told me to leave her because we come from the same village. My mother never wanted us to have that relationship but she still calls me. I don’t have a job but I am hustling

I: Okay. How does being in school, college or university for young people like you affect their access to sexual and reproductive health services and information?

R: What I can say is that it is challenging to boys like keeping themselves clean.

I: When you think of it in terms of the services of you get at the clinic, what challenges would a school going youth or adolescent face? For instance when you want to come for condoms, family planning among other sexual and reproductive health services how does being in school or college affect your access?

R: I have no idea

I: May be I can ask it differently. For example, when you are in school and you want condoms. It is among the sexual and reproductive health services offered and but are in school and you need them. How does being in school affect your access?

R: You know nowadays condoms are not hard to get. They are placed everywhere, like me I have one box here. If you want I can give you for free. They are placed on the windows in hospitals and at the condom dispensers

I: What about other services? For example a girl in school who needs family planning method. How does being in school affect her access to the family planning?

R: The question is hard

I: Mmmmm, the question is hard. Okay. I really want to understand how being in school affects one from accessing sexual and reproductive health services?

R:I was in a boarding school so I did not used to have sex

I: Mmmm, so it didn’t affect you?

R: Yes

I: What about someone who is in need of the sexual and reproductive health services and is in school? How does being n school affect their access?

R: Being in school requires one to ask for permission to go to the clinic. In my school we used to ask for permission and go to the nearby village facilities. It was called Nyang’ande Health center.

I: Nyang’ande?

R: Yes Nyang’ande. They used to test people for HIV but I did not allow them because I knew my status. So, that’s how they can get access to the services.

I: Okay. How comfortable will you be discussing your sexual and reproductive health concerns with a lay female provider, somebody of your mothers age?

R: That is hard….

I: Mmmmm, why?

R: Its hard but when I am here and somebody like that is available, then I will be free to discuss with her. I can’t find anyone outside the facility to discuss with my issues even if it is a woman, I will refuse. It’s better here at the facility.

I: So, you will be comfortable discussing your sexual and reproductive health concerns with a lay healthcare female provider

R: Yes

I: Someone of your mother’s age but at the facility

R: Yes

I: Okay. What issues will you be comfortable discussing with a lay female health provider in relation to sexual and reproductive health?

R: Issues… (Pauses) Circumcision or anything she wants to discuss with me

I: Or even when you have issues concerning your sexual and reproductive health

R: Yes like STIs, if I have any pain, discharge I can tell her

I: Mmmm, will you be comfortable discussing with her that?

R: Yes

I: What about issues to do with family planning, STD prevention ?

R: Yes

I: Okay. You said that you would be comfortable to have those discussions at the facility and not at home

R: Not at home

I: Mmmmm, why not at home?

R: (Laughs…) I am not free at home

I: You are not free at home…. So the facility works best for you

R: Yes, the moment I get here I am normally free to talk about anything to do with HIV, STI.

I: Mmmmm, okay.

R: This is the place

I: Okay. We are soon winding up. I want to get your views on potential interventions that we could have for the young adults and adolescents in relation to sexual and reproductive health services.

How would you feel if we assigned you a lay female healthcare provider to be your confidant and source of information on sexual and reproductive health

R: You wan to provide?

I: No, if we provide how would you feel about it

R: Its okay

I: Mmmm its okay. So you feel comfortable with that?

R: Yea because its from the facility

I: Okay. What would be your ideal attributes of the lay healthcare provider who will act as a source of information for your sexual and reproductive health?

R: That’s easy. At least I can find a way of talking to my girlfriend about things. If I call her and talk to my girlfriend I will be free and comfortable to discuss issues on our health

I: Mmmm. What characteristics would you want to see in this person since you will be sharing your sexual and reproductive health needs with her?

R: Confidentiality

I: What else

R: Honesty

I: Mmmm

R: Trustworthy

I: Mmmmm, any other attributes?

R: No, only those.

I: Okay. So you have mentioned someone confidential, honest, and trustworthy

R: Yes

I: Okay. How would you feel if you receive your sexual and reproductive health services and ARVs at the same point?

R: That is still okay.

I: What advantages come with that?

R: No time wasting, you just come for the service and the drugs then you leave.

I: Mmmm

R: Yea

I: Do you think there are disadvantages of receiving your ARVs and sexual and reproductive health services together at the same time?

R: No, there are no disadvantages

I: Mmmmm

R: Just advantages

I: Mmmmm, please tell me more

R: Like it saves time. When you come for your ARVs you can also consult on the sexual reproductive health or get the services like condoms.

I: Mmmm

R: Yeah, you don’t just come to the clinic; you only come when you have an appointment. If it is not your clinic day then you come for a condom that is awkward.

I: Mmmmm, but at least you will get what you want.

R: No, its awkward, I do take my condoms when I come for my clinic appointment.

I: So, during you clinic day you get all the services you want at once

R: Yes

I: That’s good. It has been a good discussion with you. Do you have any questions or anything you need to add to what we have been discussing?

R: No, I am okay.

I: Thank you very much for your time.

R: Thank you too.

**AYA IDI: KCH 008**

**Site: KCH**

**Date: 21 May 2019**

**Interviewer: Harriet Fridah Adhiambo**

**Transcriber: Harriet Fridah Adhiambo**

**Version: English**

**Interviewee Details**

Age: 17 years

Sex: Male

Level of education: Some secondary

Occupation: In school

Orphan hood status: Partial Orphan

Sexually active: Yes

Marital status: Single

Have you ever impregnated someone: No

I: I would like you tell me the challenges you face as a young person

R: Challenges?

I: Yes

R: Peer pressure, education…

I: Mmmm, how is education a challenge?

R: Balancing it with the day today life

I: Mmmmm. When you talk of balancing it with the day today life, what do you mean?

R: As in you have to learn at the same time you have problems concerning relationships.

I: Mmmm so relationship is a challenge. You’ve talked of peer pressure, would you please tell me more

R: I used to study at Onjiko High School but was expelled because of peer pressure.

I: Mmmm, peer pressures made you expelled. Maybe you tell more, like what happened?

R: People cheat you to do something wrong and you also just give in then later on you are left alone.

I: Mmmm, I hope I am not pushing you too much. What led to that expulsion from school?

R: There was a group of students in my former school that were bhang smokers and being my friends, I used to walk with them. They were reported and I was also called and informed that I too smoke bhang. I defended myself that I was not part of them but no one believed. Later on our class prefect reported me to the teachers because of another mistake. The mistake and the bhang issue led to my expulsion.

I: Okay. As a young person you have experienced challenges to do with peer pressure, education trying to balance your education with relationships. Would you tell me more about these relationships?

R: I like enjoying life, socializing. I also take things seriously like relationships to an extent that I end up not focusing. As compared to the past, things have been different since I joined my current school.

I: What has made it different?

R: Just focusing on what I need to do. I stopped bothering about other things…

I: Mmmm, any other challenge you have faced as a young person?

R: Those are the only challenges I have been facing

I: Do you think these challenges have affected your academic life

R: Yes

I: How?

R: I was not concentrating on my studies, my performance dropped but now I am improving day by day.

I: A part from the challenges affecting your education-dropping in your performance. Have these challenges affected your socio economic advancements, professional achievement?

R: Yeah

I: Tell me more

R: I am talented in dancing and drawing. While in school, there was an organization that came to identify students with talents to audition in Nairobi and I was lucky to be among them. This was an achievement to me.

I: Okay. That is good. Have you tried to deal with these challenges?

R: Yea,

I: Mmmm, how?

R: Just by forgetting the past, moving on and doing the right thing

I: Mmmm. So you have succeeded in dealing with these challenges

R: Yes, I think I have. I can say that I have changed, here at the clinic my viral load was very high but now it has dropped.

I: Any other way you have tried to deal with these challenges?

R: No other way, just forgetting the past and moving on.

I: Okay. What support is available to help you deal with these challenges?

R: My dad

I: Mmmm

R: Mostly my dad

I: Okay. How is he a support system?

R: He gives me advise on what is right and wrong

I: Any other support available to help you cope with these challenges?

R: My aunt and grandmother do also help me with the challenges I have.

I: Great. Moving on. I would like you to describe to me your experience the day you were enrolled to HIV care.

R: I remember I had been sick for a long time. I was taken to Mombasa for treatment but there was no change. I was brought here at KCH and they used to withdraw blood from my vein every time I attended clinic. I asked why I was attending clinic regularly and why they were taking blood samples form me every time I came to the clinic but nobody answered. Instead, I was taking behind the clinic where they do counseling and was asked to name viral diseases. At that time, I was in class five. I named those I knew and they told me that I had forgotten one, HIV. They told me that I was HIV positive. I felt very low at first and even questioned myself “ why me?” but I consoled myself that I should not blame myself because I was probably born with it based on what I had learnt. I took it simply and gave in. They counseled me and I was told that having HIV does not mean I will be wasted but as long as I take my medication I will be healthy. Its been 7 years since I was initiated on ARVs and I have not had any side effects.

I: Mmm, okay. So, How did you feel at that time?

R: I felt very low, felt like crying

I: Mmmm, did you cry?

R: No, I started but something held me and I didn’t cry, I was just normal. I was told that both my dad and brother were also HIV positive. I took it positively though….

I: Okay. At that time, who accompanied you to the clinic?

R: My grandmother and my younger brother

I: Mmmmm, how many are you in your family?

R: Four, no five. We have a stepsister.

I: Okay. You told me that your mum died, at that time how old were you?

R: I was in class 2

I: Okay. Are you a first born?

R: I am the 3^rd^ born but the first-born boy.

I: If you can recall at the time of enrollment in care, what was going on through your mind?

R: All I wanted was to get well. Whatever was happening to me….. (Pauses) My body was swollen, body aches all over as in I was sick all the time. I just wanted to get well

I: Okay. What has enabled you to continue engaging n care at this facility?

R: Support from the doctors like W…. and M… (Names withheld). They have been encouraging me.

I: Mmmm. Any other thing that has enabled you to continue engaging in care here? How about the distance to this facility? Quality of services offered…..

R: Usually what really motivates me to come here is the company I have. When I am here at the clinic I usually feel I have a family unlike when I am with other people elsewhere I usually feel like I am the only one who is HIV positive. I just love this place.

I: Okay. Have you ever ben discriminated against because of your HIV status?

R: No

I: Could you have friends who could have experienced that?

R: I don’t know. Usually when I am with my friends who are of the same status we do not talk about HIV. All I tell them is to take drugs as expected

I: Mmm, okay. Please tell me anything you know about ARVs

R: I know they are supposed to be taken on a daily basis to reduce the no. of virus in the body

I: Good, you have told me that ARVs are taken to suppress the virus and are taken on a daily basis. What else?

R: We are also taught that they do prolong life

I: Mmmm, any other thing?

R: Only those I have mentioned

I: Okay. Tell me about the time you missed taking your ARVs, what happened?

R: I usually forget

I: Mmmm, on this specific day that you missed did you miss or is there something that happened till you did not take your drugs?

R: Distraction especially while in school. Mostly I’m with my friends and we end up story telling till I forget to take my medication.

I: You couldn’t excuse yourself to go and take the drugs?

R: I just forgot

I: You just forgot.

R: Yes

I: Could you please share with me the challenges you face with keeping your clinic appointments?

R: I don’t have a challenge with attending the clinic

I: And now that you are in school, isn’t it a challenge?

R: No

I: How have you managed?

R: I just go to the School principal and ask for permission. The school knows that I am supposed to go to the clinic for my ARVs.

I: Okay. You are in boarding or day school?

R: Boarding school

I: Okay, so you are allowed to come and have so far not experienced any challenge.

R: Yes

I: Have you ever missed your clinic appointment not necessarily when you are in school/

R: Yes, once. I looked at the calendar wrongly, I knew that I was to go to the clinic at a later date during the month yet the scheduled visit had already passed.

I: So you confused the dates and ended up not coming as scheduled?

R: Yes

I: So, what happened when you realized this?

R: My aunt came the next day for the drugs on my behalf.

I: What are your general feelings about the HIV care services you are currently receiving?

R: I am just okay

I: When you say okay, what do you mean?

R: I don’t have any problems with the services, I am just okay

I: Is it that the services are good, bad or both?

R: The services are good.

I: Mmmm, tell me more

R: The services are good because I am known here. I attend my clinic regularly. When I come here they greet me, find out how I am doing and I share a lot with them.

I: Okay. How do you think your encounter with HIV care services can be made more satisfying?

R: As in?

I: How can the services you receive here be made more satisfying to you?

R: I think everything is okay. I don’t see anything…

I: You don’t see anything that can be improved in relation to the services you receive?

R: Maybe to others. Everything is okay for me

I: Mmmm everything even the care providers?

R: Yes

I: Have you identified anything that would want to make you continue coming to the clinic or get the satisfaction with the services you receive

R: May be they buy a TV and have it at the reception.

I: Ahaaa

R: Only that

I: Mmmm, what else apart for the TV?

R: Video games

I: Mmmmm

R: I don’t have ideas but either way I will still come to the clinic

I: How does being in school affect keeping your clinic appointments?

R: It doesn’t affect, once I look at my clinic card and identify the date, I will plan in advance and ask for permission to attend.

I: Okay. Do you think being in school affects other from attending clinic?

R: If it affects them then that’s their own problem because I usually don’t care about other people. If I am asked where I am going I tell them that I am going to pick drugs and if they ask me questions I just ignore.

I: How about taking your ARVs? Does being in school affect taking your ARVs?

R: It doesn’t affect, when the time comes for me take my drugs, I just take them. In this world you were born alone and not with other people

I: Mmmm, do you think other young people not necessarily you, face challenges of taking their medication because they are in school?

R: Yes, they are people who face challenges mostly my friends.

I: Mmmmm

R: My friends whom I studied with them at Ojinko. I kept encouraging them that no one will beat you up like my father always says even if people know your HIV status they will not kill you, they will just talk and later keep quiet when they see you are healthy.

I: So your friends were afraid of taking their drugs?

R: Sort of

I: Mmmmm, so they were not able to attend their clinic as expected?

R: They used to go. I don’t know how they used to attend their clinic but I know that they used to go.

I: And taking ARVs?

R: Sometimes I would ask them and they would say they have taken but I am not certain if they did.

I: Do you feel it was challenging to them?

R: Yeah

I: Mmmm, okay. We want to discuss issues to do with sexual and reproductive health.

From your own perspective, what do you understand by the term sexual and reproductive health?

R: It is just….. (Pauses) It is to with teenagers practicing safe sex and maintaining their health

I: Mmmmm, any other thing you would like to add

R: Only that.

I: Okay. Are you able to relate sexual and reproductive health to STD (Sexually transmitted disease) , safe sex, condom use and family planning.

R: Okay

I: How important is your access to sexual and reproductive health services?

R: People who do it… (Pauses) I have never practiced it. Therefore, it is good to ensure you use protection i.e condom use

I: Mmmm, so what you are saying is that the access is important because you need protection fro other infections?

R: Yes

I: Currently, where do you access information in relation to sexual and reproductive health?

R: Mostly the forums I attend like the fun day organized by KCH and Sunburst. Then other day on Saturday we had a camp and we were taught on sexual and reproductive health.

I: So you get information during camp out and fun day. Where else do you access this information?

R: Here at the facility

I: Okay. Where would you like to access sexual and reproductive health services and information?

R: Place to access?

I: Yes

R: At the hospital

I: You would prefer to receive the services at the hospital?

R: Yes

I: Okay. To what extent are the sexual and reproductive health services offered to young people like you meet their expectations/needs

R: Pardon

I: To what extent are the sexual and reproductive health services offered to young people like you meet their expectations and needs?

R: Yeah, to some

I: Mmmm, tell me more please

R: You know teenagers are usually stubborn. They are told things and someone else also tells them another thing. He/she will be confused and does not know what to listen to. But if they are willing to listen then they will understand what they are being told.

I: So, it meets their need

R: Yes

I: Okay. As a person, what challenges do you face when trying to access sexual and reproductive health services?

R: Is there a challenge in accessing the sexual and reproductive health services?

I: No, I am asking if your as a person has challenges when accessing the sexual and reproductive health services?

R: I don’t have a challenge I always access them through the facility

I: Okay. So you do not experience any challenge

R: No

I: Okay. Does being in school for young people like you not necessarily you but even young people of your age affect their access to sexual and reproductive health services?

R: Yeah

I: How does it affect?

R: As in they should be taught in school? Mostly in school there are instances where there are outings and interaction occurs where boys and girls meet. So, they should be taught about these things so that just incase they end up doing anything they know how to abstain..

I: Mmmm, do you think being in school will hinder a young person from accessing those services?

R: No, in most schools some organizations usually visit and teach them. Like our school we have had several organizations coming to teach us on sexual and reproductive health.

I: As they talk to you or as they give you the information, do they also offer the services to you? For example, do they give you condoms?

R: No, just the talks

I: Okay. What if you want to access condoms and you are in school? Or for females you are in school and you want to access family planning

R: If they are in school why do they need it and where are they going to use? Unless you are planning to sneek out. I don’t think they should be given while in school, they should just access them when outside the school.

I: Okay

R: It may also hinder their academic performance

I: Mmmmm

R: They will just be thinking of those things.

I: Are there instances where someone is in school let use a female. The female student wants family planning. What that means is that they have to go to a facility where they will be offered the service. So, can being in school affect your access to that family planning?

R: No, you can just ask for permission and go

I: Will you be granted?

R: Some schools you will be granted whereas others you will not

I: Mmmmm okay. How comfortable will you be discussing your sexual and reproductive health concerns with a lay female healthcare provider, someone of your mothers age?

R: I have no problem.

I: That is interesting, why?

R: I am always free

I: Okay. What issues will you be comfortable discussing with the lay healthcare provider in relation to sexual and reproductive health?

R: Anything

I: Like what issues? Maybe you could give a few examples

R: You are the one to tell me what to ask

I: Okay. For example, you have a girlfriend and you need condoms. Are you free to discuss it with the lay female healthcare provider? Or even you feel you have signs and symptoms of an STI, would you be free to discuss with her? Or even you impregnate someone; will you be willing to share with her?

R: Yes, I will be free to discuss with her

I: Okay. What attributes or characteristics would you want in this person you are going to confide in?

R: Characteristics?

I: Yes, the characteristics of the lay female healthcare provider you will be discussing your sexual and reproductive health needs with

R: As long as I am not being pressurized

I: Mmmm, someone who is not pressurizing you. What else?

R: Some one who is free and approachable

I: Mmmmm. You have said you want someone who is free, approachable and does not pressurize you. What else would you like to see in this person? Remember this is someone you are going to confide in on personal issues and sexual and reproductive health concerns

R: Lets say this person is ready to help

I: Mmmmm

R: Willing to give you advise

I: Mmmmm, okay. Where would you want these discussions on sexual and reproductive health to take place?

R:In a room?

I: Could it be a facility? Home?

R: Anywhere in school, home or even facility

I: Mmmm, why?

R: Just anywhere as long as it is private.

I: Okay. We are almost winding up. I want us to discuss on the potential interventions in relation to access to sexual and reproductive health information and services among adolescents and young adults.

I: How would you feel if we assigned you a lay female health care provider to be your confidant and source of information for your sexual and reproductive health?

R: I would be okay with it

I: You would be okay with it. And how comfortable will you be discussing your sexual and reproductive health issues with the lay female healthcare provider?

R: How?

I: How comfortable will you be discussing your sexual and reproductive health issues with the lay female healthcare provider?

R: I will be confortable.

I: Okay. What do you feel about receiving your ARVs together with sexual and reproductive health services together at the same point?

R: At least you will be taught that when engaging in sex you should use protection. Also you will be taught on family planning, condoms

I: Okay. When you come for your ARVs and sexual reproductive health services and you receive them at one point, how would you feel?

R: Just normal

I: What advantages are there in receiving your ARVs together with the sexual and reproductive health services at the same point? For example like today when you come to the clinic for your ARVs and the same place we are offering sexual and reproductive health services. What are the advantages?

R: Receiving information, being taught

I: Any other thing that would be advantageous if you receive these services at the same point?

R: None

I: Are there and disadvantages of receiving ARVs together with sexual and reproductive health services at then same point?

R: No disadvantage

I: Mmmmm, thank you very much for you time. We have come to the end of our interview. Do you have any questions in relation to sexual and reproductive health services among adolescents and young adults?

R: Yes, at my age like now I am teenager is it a must I engage in sex?

I: Mmmm, what do you think?

R: I don’t know that’s why I have asked

I: You have a very good and valid question. I am going to refer you to one of the staff here so that you can have a one on one discussion. What do you think?

R: No, It’s not something affecting me

I: I know its not something affecting you. It’s just a question

R: I want you to answer

I: Mmmm, okay. Since you have been coming to the clinic and this is your center. We have adolescents here and young adults whom you interact with during support groups and health talks. What are you told about sexual intercourse?

R: They have always told us to just interact but if we engage, we use protection. They have never told us not to have sex

I: At times it is not easy to tell someone not to have sex especially when they have already started engaging in sex but it is good to be faithful to your partner, use condoms because of the risk of transmission of diseases to another person and abstain if you haven’t engaged till when you get a partner.

R: Okay.

I: Thank you

**AYA IDI: KCH 010**

**Site: KCH**

**Date: 21 May 2019**

**Interviewer: Harriet Fridah Adhiambo**

**Transcriber: Harriet Fridah Adhiambo**

**Version: English**

**Interviewee Details**

Age: 19 years

Sex: Female

Level of education: College

Occupation: In school

Orphan hood status: Not orphaned

Sexually Active: Yes

Marital Status: Single

Ever pregnant: No

I: What are the challenges you face as a young person?

R: I have a lot of challenges.

I: Mmmm

R: Sometimes my mother does not have money to send me for up keep in school. I am forced to prepare porridge and take as that is the only thing I have. I stay outside the school compound. I don’t share with anyone my problems; I just keep them to myself because at one point I shared with a friend of mine my financial challenges and he ended up telling my classmates that he is the one who supports me financially. I didn’t like it and I decided to keep to myself the challenges my household and I had.

I: Okay. So you have said you are financially constrained and not receiving supports from your parents

R: Yes

I: Just so that I may understand. Do you have both parents?

R: Yes

I: What is their occupation?

R: My dad lost his job when I had just completed my high school. He had gone to attend his mother’s burial and had not asked for official permission from work. When he reported back to work he was sacked. He is currently just in the house with no source of income. My mother on the other hand does small-scale business. She sells groceries and fries next to our home. Sometimes she calls to inform me that sales were not good and can only afford to send me fifty shillings.

I: Okay. Apart from financial constrains, what other challenges do you experience as a young person?

R: None

I: Okay. How do you think these challenges have affected you?

R: Education wise they have affected me. I become stressed while in class, I am not able to read and I am forced to go outside and sit at the green bench and chat.

I: What do you mean by green bench?

R: It’s a place where you can access Internet services while in college. So I go and sit there then later go to my room.

I: So you are not able to study as expected

R: Yes, I find myself crying while in my room and just wonder why I have to go through this. Sometimes I hear my friends talk about their house, how they have been sent money like even 2,000/= and this makes me even more stressed.

I: Mmmm, have you tried dealing with these challeges?

R: I have tried. I share with Lillian and she often encourages me a lot. She encourages me to be patient and sometimes that’s how life is.

I: Mmmm, how else have you tried to deal with these challenges?

R: No other way. I just share my problems with her

I: Okay. To what extent do you think you have succeeded in dealing with these challenges?

R: Yes, I think I have succeeded. Keeping quiet about my problems has in a way helped me to deal with the challenges. I cannot share with anybody because they will discuss my problems with other people.

I: What support is available to help you deal with these challenges?

R: I can’t see any

I: Mmmm, do you have people who support you when you experience these challenges?

R: I can say that it is only Lillian who sometimes can help me by sending me money like 200/=. Even the transport I have used today she is the one who sent me. She finds out how I am doing and even reminds me to take my medication. I had once told her that I would stop taking the drugs so that I can die then my mother will remain peaceful but she really encouraged me and always assists me.

I: Any other support available to help with these challenges? Like you parents, friends or even relatives?

R: I cannot share my problems with my mum because she will get stressed and start crying not knowing what to do. I usually don’t want to bother her.

I: Okay. I want us to talk about HIV care services. Can you please tell me your experience the day you were enrolled to HIV care?

R: I came to the hospital with my mum and I was tested. When we went back home, my mum was crying. By then I was very young, about 6 years old and had not started attending school but was very keen. I asked her why she was crying and she didn’t tell me the reason. When I joined primary school I started asking her why I was taking drugs on a daily basis and she opened up and told me that I was HIV positive and that is why I had to take those drugs on a daily basis.

I: Mmmmm

R: One day while in school I asked my teacher what HIV was. Instead of responding to my question, she called me outside when the lesson was over and found out why I had asked that question. I told her that I have been taking drugs and my mother had told me that I was HIV positive, so I wanted to understand what it meant being HIV positive. She told me that I should not tell anyone about my status and keep it to myself.

I used to take my drugs as expected till when I reached Form II. I could go pick my drugs from the clinic but not take them instead I used to throw them away. I became sick and was admitted here at the hospital. The clinic staff counseled me and I was told that if I don’t take the drugs then I will die. After discharge, I embarked on taking my medication as expected.

I: Has everyone in your home tested for HIV?

R: My father is not HIV positive. It is only my mother and I that are HIV positive.

I: How about your siblings?

R: I am the only child in that in that family

I: Since that day you were enrolled to HIV care what has been going through your mind?

R: When I reached class 6, I started wondering where I got the disease because my father was not taking the drugs and I used not to see my mum taking them too. I thought that probably I got the disease while playing childhood games of being a parent or even had cut myself with a razor blade I had picked. My aunt was HIV positive and unfortunately she died. I was scared and thought that I was also going to die. I used to cry over it but my mum encouraged me that since I was already taking ARVs, I was not going to die.

I: What has enabled you to continue engaging in care at this facility?

R: The advice and encouragement I receive from the clinic staff. This has enabled me to continue taking my drugs as expected and even my viral load has dropped drastically and they are very happy. They do appreciate my efforts. My viral load had thousands of copies and I promised them that it would go down.

I: Apart form the support you receive from the care providers what else has enabled you continue engaging in care here?

R: My neighbor in college. When it reaches 6.00pm, she finds out if I have taken my medication and at times even she goes to my room and brings the drugs plus water form me to take. Even when I don’t have food, she insists that I have to take medication even with porridge.

I: Is she your classmate?

R: Yes, she is.

I: Do you reside at the hostels or outside?

R: No, I have rented a house outside college. The hostels were full when I reported in January.

I: Can you tell me any instance where you ever experienced stigma or been discriminated against because of your HIV status? For example, someone not letting you do something or not wanting to be around you?

R: Yes, that once happened when I was in high school. I used to tell my friends that I had a chest problem that why I was taking drugs on a daily basis. One day, one of my friends opened my bag in my absence and read the names of the drugs. When I came back to the dormitory, she telling my friend that I pretend that I am having a chest problem yet I am taking ARVs. I immediately looked at my bag and found it had been tampered with. I then knew she had accessed it. When I asked her if she opened my bag she rudely said that my bag had ARVs.

She told me that I have been walking around with men that is why I am HIV positive and that I should take my ARVs as expected. I was angry and told her that I know I my HIV positive and that the drugs are helping me.

I: Mmmmm

R: One day the doctors came to visit our school and they were testing people for HIV among other services that were being provided. I prayed to God that my girlfriends who mocked to be also tested for HIV. Fortunately, they were tested as I had wished but it ended up that they were found to be HIV positive. I did not delight in their positive results but told them that they should also take the drugs and take as they had earlier told me. I encouraged them to take their medication as expected and they will not die and we became very good friends to date.

I: When your friends told you that it is because of your behaviors of going out with men that made you take ARV, how did you feel?

R: I felt very very bad. I even made up my mind not to be talking to any boy/man while at home, instead just be indoors. During the holidays, my mother wondered why I was not going outside to meet my friends since I was spending most of my time watching television. I was afraid my friends would meet me and start telling people that I am HIV positive. This continued till I finished my secondary education.

I: Were you able to overcome that?

R: I did. I told my mother what was happening and she really encouraged me. She even told me that even when I get a boyfriend I should not fear but disclose to them my HIV status.

I: Okay

R: When I met a certain boy who later became my boyfriend, I disclosed my status to him and he was very supportive. He later got a job and started ignoring me, not sending me money for shopping and worse of all not even responding to my calls. I decided to move on with my life and focus on my education. I already had support from the clinic staff like Lillian.

I: When you disclosed to him your HIV status, how did he take it?

R: He took it in well and was very supportive. Currently if I call his number, he is either unavailable and doesn’t even respond to the missed calls. I feel like I am the only one interested in this relationship and I have decided to move on with my life and forget him.

I: Has this affected you in any way?

R: No, initially it used to stress me. I used to wonder why J… (name withheld) is not concerned about me. My friends boyfriends could come and visit them in our classes while me I am just there alone. I wondered how I was created, was is it because of my status and everyone knew and that is why they were avoiding me? I sometimes wished for death. One day I even bought poison so that I could take away my life. Luckily, one of my friends came to find out what I had prepared for dinner and found the poison on my table. She poured it and talked to me discouraging me from committing suicide. She encouraged me that all was not lost and all will be well. I was tired; my mother was struggling to provide for my family and I and it was best at that moment if I died so that she could rest from all the troubles. My friend was so emotional and cried, she promised to always support me.

I: Mmmm sorry about that. Did your boyfriend know his HIV status?

R: Yes, I brought him to this clinic and he was tested. He was HIV negative.

I: Tell me anything you know about ARVs (Anti retroviral drugs)

R: Mmmm, the drugs help us. They kill the virus and make them inactive. Also they prevent the virus from multiplying in the body. That’s all I know.

I: Any other thing about ARVs?

R: Only that

I: Have ever missed taking your ARVs?

R: I have missed taking my drugs several times. I used to lie to my mother that I have picked the drugs yet I never even attended the clinic. The clinic staff (names withheld) M and W used to look for me at home and tell my other that I no longer come to the clinic. I told my mother that I did not want to continue taking those ARVs. The clinic staff counseled me and I embarked on medication. Nowadays I come to the clinic as expected and take my drugs as prescribed.

I: So, what happened when you missed your medication?

R: I became very weak and could not do any chores

I: Mmmm, why did you miss

R: I just used to miss. I used to tell myself that it is not a sin to miss even with one day and I wanted to see what would happen to my body if I missed. The next day I would encourage myself that nothing happened to me when I missed the previous day and this became a habit.

I: Kindly share with me the challenges you face keeping your clinic appointments?

R: No, I don’t have any challenges

I: You don’t have any challenges? You are able to come to your clinic as expected?

R: Yes

I: The last time you missed taking your ARVs, did you also miss your clinic appointment?

R: Yes, I missed several appointments

I: Tell me about this specific day that you missed. What happened?

R: I belong to a dancing group known as Sakata. On this day, I was supposed to be coming to the clinic were having a dance at Kibos. I decided to attend the dancing meeting instead.

I: Okay. What are your general feelings about HIV care services you currently receive?

R: Very good services. We get advice

I: Any other thing apart from advice?

R: Everybody at the clinic loves you and no one discriminates against you. The staffs are free and friendly

I: How do you think your encounter with HIV care services can be made more satisfying?

R: The services are okay.

I: You don think there is anything that can be done to make you encounter more satisfying?

R: They are okay.

I: Okay. How does being in school/college for young people like you affect keeping their clinic appointments and taking their ARVs?

R: It depends on an individual. I am currently in college and I know the benefits of taking ARVs. I will come to the clinic on time since I don’t want to bargain with my life. My CD4 has at one time been very low.

I: Okay. Does being in school interfere with your clinic attendance?

R: No

I: How about taking drugs?

R: No, I take my drugs as expected. My drugs are taken once a day. So I take them at 6.00pm till the next day again at 6.00pm that finds when I am out of class.

I: What if it finds you in class?

R: I usually carry in my bag and I will just take even if people are looking at me. That is their own problem and I am continuing with my life. When they ask me I tell them that it doesn’t concern them.

I: What do you understand by the term sexual and reproductive health?

R: (Pauses and Laughs…) I don’t know

I: May be you can think of it in terms of safe sex, contraceptives/family planning use, access to reproductive health information like condom use, prevention of STD/STI’s.

R: Okay

I: Currently, where do you access information on sexual and reproductive health?

R: No, I don’t receive any information

I: You don’t receive any information in regards to sexual and reproductive health? How about here at the clinic?

R: Yes, at the clinic we are taught but it’s been long since I attended the support group meetings

I: Okay. So you received the information at the clinic?

R: Yes. M and W taught us that when we have sexual partners we should not hide but disclose our status to them to avoid spreading the disease.

I: So you were being taught here at the clinic?

R: Yes, it was during support groups that use to take place every Saturday at the clinic.

I: Apart form the clinic, where else do you get information on sexual and reproductive health?

R: In college when we attend the love skill sessions

I: Okay. What do you mean by the term love skill?

R: Love skills are educative sessions we have in college about our relationships with then opposite sex. We are taught on how to use condoms and other love related issues like being faithful among others.

I: Okay. What is the ideal place/location where you would like to access sexual and reproductive health services?

R: When you are just two people

I: Okay. Is it a t the hospital, home or where?

R: Anywhere, even home and hospital.

I: Okay. Where would you like to access those services? May be at one point you may need condoms or family planning or just need someone to give you information on how to use condoms or protect yourself from infections. Where would you want to access these services?

R: At the hospital

I: Why at the hospital?

R: Because the staff are very knowledgeable

I: To what extent are the sexual and reproductive health services offered to young people like you meet their needs and expectations?

R: It doesn’t meet their expectations because they do not do as they are advised. For instance, you tell them to use condoms but they do not use yet they do not know even then HIV status of their partners

I: Okay. What challenges do you as a person face in trying to access sexual and reproductive health information and services?

R: I haven’t experienced any challenge

I: You haven’t experienced any challenge?

R: No

I: How does being in school/colleges for young people like you affect their access to sexual and reproductive health services?

R: It doesn’t affect because young people will always find ways to access these services, so I don’t think if being in school is a challenge to them.

I: How comfortable will you be discussing your sexual and reproductive health concerns with a lay female healthcare provider of your mother’s age?

R: That is hard, it better when you get a younger person to discuss with you issues and can advise you. When I have a problem I come to the clinic and Dr. O advices me on what is right and wrong.

I: Not necessarily a doctor but someone within the clinic whom you can share with your concerns. Will you be comfortable?

R: Yes

I: What issues will you be comfortable discussing with this person?

R: About your boyfriend and the challenges you could be facing in relationships

I: Any other thing you would be willing to discuss

R: Only those.

I: Where and why would you want those discussions on sexual and reproductive health to take place?

R: At the hospital so that we meet together. You know at the hospital you will receive adequate information that which we know and do not know.

I: Okay. We are soon winding up. How would you feel if we assigned you a lay healthcare provider to be your confidant and source of information for your sexual and reproductive health?

R: That will be good. Because if for example I am planning to have sex, I will discuss with her and she will advice accordingly. Sometimes even you want to be sure when you are safe to have sex during your cycle and she will help you count.

I: Okay. What would be your ideal attributes/characteristics of such lay healthcare provider to act as your source of information on sexual and reproductive health?

R: I would want someone who is calm and confidential.

I: What else?

R: Polite. Not someone who will discuss your issues with other people and pretends to be good.

I: Mmmmm, any other attributes.

R: None.

I: What would you feel about receiving your ARVs together with your sexual and reproductive services?

R: That would be great.

I: Why do you feel that would be good?

R: I feel so because I will be helped.

I: How?

R: Just like that.

I: Okay. What are the advantages receiving ARVs and sexual and reproductive health services together at the same point?

R: The question is so hard. I cannot explain

I: Okay. What I am asking is that when you come for your ARVs and also at the same point you receive family planning for example. I am not saying that you use family planning, its just an example. What is the advantage of receiving these two services together at the same point or would you prefer if today you come for your ARVs then after 3 days you come back for family planning?

R: Receiving them together would be good.

I: Mmmm

R: Pauses (Yes)

I: Okay, do you think there are any disadvantages of receiving the services together at he same point?

R: No

I: Okay. Thank you very much for you time. We have come to the end of our interview. Do you have any questions?

R: No, I don’t have any question

I: Thank you

R: Welcome

**AYA IDI: KCH 012**

**Site: KCH**

**Date: 17 May 2019**

**Interviewer: Harriet Fridah Adhiambo**

**Transcriber: Harriet Fridah Adhiambo**

**Version: English**

**Interviewee Details**

Age: 21 years

Sex: Female

Level of education: Completed secondary

Occupation: Unemployed

Orphan hood status: Total orphan

Sexually Active: No

Marital Status: Single

Impregnated woman: No

I Fell most welcomed. I would like us to begin our interview.

R Thank you.

I Generally, what challenges do you experience as a young person?

R Challenges in terms of?

I Just general challenges you could be experiencing

R Like right now I am idle. There is a lot of sitting that is kind of boring and also being new in Kisumu is also hard.

I Oooh, where were you before?

R I was in Nairobi.

I Living with your parents?

R No, my parents died. I live with my aunt, the sister to my mother.

I Okay. When did they die? When you were still young or recently?

R My mother died when I was 2 years old and my father died just a few months before I w as born.

I: Sorry about that. So you stay with your aunt?

R: Yes, I stay with my aunt.

I: Your aunt moved to Kisumu?

R: No, here in Kisumu I stay with my elder sister.

I: Okay. How many siblings are you?

R: We are five

I: Okay. You have said one of the challenges you are experiencing is being idle

R: Yes, I am idle. I was supposed to join campus but I haven’t because I became sick and was admitted. I had to defer till September.

I: Which university were you to join?

R: Maseno University. I was going to do Social work.

I: What other challenge do you experience apart from being idle?

R: No that is so far what I am experiencing as a challenge.

I: Do you think this challenge has affected your educational, professional or socioeconomic advancements?

R: To some extent yes because now I am idle and broke. I am broke and it has reached a point where I am not comfortable asking for minor things I just want to stand-alone. Those are the kind of challenges I am facing.

I: Being idle since you don’t have anything to do, you were supposed to join school but you had to postpone till September and again you do not have a job so you are forced to depend on someone else for money. And a you feel like…

R: Mmmm, it sounds like you are being too much

I: Ahaaa, any other challenge?

R: If it is general challenge, I don’t have any other unless you tell me some (Laughs..)

I: Just what you as a person could be going through. My challenges could be different from yours (Laughs..)

R: And considering with my status there are also challenges.

I: Mmmm

R: Maybe they are not here right now but sometimes back. Like 2 or 3 years ago I was undergoing stigmatization while I was in school and this led me default from my drugs and my performance in school was not that good since I was in and out of school. Those are the kind of challenges I face, it is not easy to open up to everyone and disclose to them your status and even if you open up to anyone you are not sure of their reception.

I: Okay. You have talked of stigma while you were in high school. Were you in a boarding or day school?

R: I was n a boarding school

I: Could you please tell me what happened?

R: I accidentally went with the yellow card we are issued with at the clinic to school. I went with it unknowingly then one of my classmates found it in my box and went and told everyone in my class but I wasn’t aware. When I came to class I found my locker where it was not supposed to be. My locker and books were outside and when I returned them back, my desk mate did not want to sit with me. So it was like I am in school but I did not have any friends, the whole school knew my HIV status and they feared that I might infect them. I could not share books, cups or anything with them and even if my plate was lost, I could hungry because no one was willing to share with me.

I: Mmmm

R: Yeah, those are the kind of challenges I went through

I: I’m sorry about what happened. How did you feel when your locker was taken outside?

R: That was then lowest point in my life and I attempted suicide because I thought that the pressure was just too much. I thought it was best to end my life and end all this suffering. Unfortunately, my suicide did not got through

I: Mmmm, what wee you planning or what did you do?

R: I tried drinking jik but it did not work. I tried overdosing with the ARVs, it didn’t work then I tried moving to a running motorbike but my brother unknowingly saved me.

I: Sorry about that. Just to go back to how you were being stigmatized, did report to the teachers in school or shared with someone?

R: The only people who knew about my status were the headmaster and the school nurse. I felt like I am alone. I am more of an introvert. Even when I went home, I did not share with my parents/guardians I just kept it to myself and it depressed me even more. I used to go to the clinic monthly but I was not taking the drugs. I just kept them in my bag. By not taking the drugs I would die slowly so I thought of something that would kill me faster but they did not all work.

I: I’m so sorry about that. Did it affect your performance in school?

R: Yes, very badly. I performed poorly

I: How was the trend?

R: I started failing even in subjects that I was good at. The teachers got worried but they knew I was in and out of school. They knew I was not okay as I was always sick but none came to ask me what was happening until a time when they were searching for some lost items in the dormitories. One of the teachers opened my box and got the drugs. That is when then other teachers knew that I was HIV positive because there was drugs like for 2 years in my box. You can imagine how my box was, like a pharmacy.

I: Mmmmm

R: I was taken to the director’s office and to where I always took my drugs for counseling but I did not take them seriously. It was just for a formality even after being talked to I still did not take my drugs until 2017 when I started taking my medication well.

I: What happened in 2017 that made you to start taking your medication well?

R: I moved from Nairobi to Kisumu and Joined Bishop Ojolla School. I transferred my clinic to Lumumba and that’s when they gave me hope. I was taken through counseling and I even saw my peers who were very healthy and that when I knew the world needs me.

I: Wow! I like that statement. It’s a motivation

I: If we go back to the challenges you earlier mentioned like you are idle, you don’t have money. Have you tried dealing with those two challenges; leave alone the HIV related challenges

R: No, I am the last-born. I don’t think even my sister will allow me to go and hustle somewhere. She is really close to me and if I get to a point that I am struggling, she will really support me.

I: Okay. To what extent have you succeeded in dealing with these challenges?

R: Idleness none, I cant because it is beyond my reach unless something happens. I was trying to see if I can volunteer somewhere but I haven’t had that opportunity. I am still looking into it.

Stigmatization, I am still trying to deal with it. It is still a problem but not as much. I know I can do it.

I: What support is available to help with the challenges?

R: If I can get somewhere to engage myself it would be good. I like doing a lot of charity work and this will help deal with the idleness. If I can get somewhere where people are not stigmatized, people are friendly I can really do a lot of the charity work and maybe one of these days I can be able to open up to the whole world about my status and I wont be ashamed of it.

I: Okay. DO you have people who can support you in dealing with these challenges?

R: Yeah, I have. My sisters, brothers and family do support me

I: Apart from your family do you have any other people who support you?

R: Yes, I have friends who do support me

I: Great. I want us now to discuss more on the HIV care services. Just to take you back a little bit I know we have discussed about stigma but could you please tell me your experience the day you were enrolled to HIV Care?

R: I was so much relaxed that I think I surprised the doctors. I knew my status when I was in standard 6. I was very sick while in school and my dad or uncle summoned. I call him dad because he is the one who raised me. I was taken to Nairobi women’s hospital and that was the first time I heard this test known as CD4. The doctor ordered for that test and the results were 364 and he said he was impressed with the number and there was no need for me to start the drugs then so I just go on with septrin. I used to take septrin but I did not know why.

I: So nobody told you that you had been tested for HIV and explained why you were taking the drugs daily?

R: No, no one

I: Okay. So, your dad accompanied you to the hospital the day you were being tested?

R: No it was my mum who accompanied me. She was then one talking to the doctor and I was never involved in the discussion. I knew when I ask my mum what CD4 count is she wouldn’t have told me because if she wanted to she would have told me along time ago.

I: Mmmm

R: I stayed home for like 3 months and even after I had recovered, my mum did not take me back to school. When I asked why I was not going back to school she said that once I am okay I would go back. Eventually I went back to school then I wen to my science teacher and I asked him what CD4 count was. He was surprised like how did I know of CD4 and I was just in class 6. He explained that it is a test that is done to patients who are HIV positive. He asked me why I was interested in it and I told him that I heard the some doctor tell another patient but I did not mention that it was I. The discussion ended and that is when I knew I was HIV positive. I had my doubts before because I was ever sick and always had sores in my mouth and you know HIV is a topic in science lessons so I read the signs and symptoms. This time round when I learnt of that CD4 was done to HIV positive patients then I knew that I was also HIV positive. I did not tell my mum till 2013 when I became sick again and when my CD4 count was taken it had dropped to 11 copies. I can remember one of the doctor’s say that I may die and may not survive.

I: Sorry

R: Yeah, I lost my temper and called the doctor telling him that I was ashamed of him. He was supposed to be giving me hope that I will be living instead he was saying that I would die. At that time I was in form 1. The day of disclosure came and my mum could not even bear to go with me, so she sent her daughter who is my cousin to accompany me. At first they all refused because they did not know how I was going to react. I am this girl who is full of life and they probably thought that I was going to be depressed. When we reached the hospital, I even made the doctors work easier, they came and started talking like .. “ You know…..” You know how the doctors talk and behave during disclosure (Laughs). They take us through all those bushes before they tell us what is happening. I told the doctor that there is no need of stressing yourself because I know that I am HIV positive and today I am here to pick my drugs because my CD4 or whatever tests you call it is very low. The doctors were surprised and even asked me if I knew what I was talking about and if I new what being HIV positive is. I told them that I knew I was HIV positive and I should be taking ARVs. They thought that I was mad and even called my sister. I told them I was okay and they should give me the drugs and tell me how to take them.

I: Mmmm. So that means you mum or aunt use to give you medication but did not explain to you why you were taking them

R: Yes, it was just septrin because I had not been initiated on ARVs

I: Okay. So when your CD4 was 11 is when you were initiated on ARVs

R: Yes. When I was from the hospital my sister was crying and I even wondered if she was the one who was given those ARVs or it was I. I was actually consoling my sister that it was okay and I can go through it, as it was not planned and that is how life is. I was consoling her instead of her consoling me. At first I really embraced it, even when my mum came back from work and found me playing outside with other kids then first question she asked me was if I had gone to the hospital. I told her yes, and she was like “ then?” I am supposed to take my ARVs as I was told. She wasn’t convinced and had to call my elder sister who accompanied me to the hospital just to find out if we truly went to the hospital. My sister told her that I have been jovial since we went to the hospital but my mum thought that I was not okay and I had to be taken back to Nairobi hospital for check up. I told her she was wasting money because I was okay and I knew that I am supposed to be taking ARVs. The doctors checked me and she was told that I am fine. My first reception was very good until 2015.

I: Mmmm, so what happened in 2015?

R: That is when stigmatization came in

I: Okay. That is when your locker was removed outside and nobody wanted to share his or her items with you

R: Yes. I remained without a desk mate till I finished my form 4.

I: So, how were you sitting in class?

R: I was sitting alone

I: Like where?

R: In class. They cannot chase me out of class. My desk mate shifted to the person seated behind me so there was space. We used to sit two people in class but now they were three. It was to my advantage because my plate’s cups and buckets used not to get lost as no one could touch them (Laughs)

I: (Laughs) Mmmm

R: Even textbooks nobody could borrow me, so I used to do my homework and keep them. I used not to lock my locker and I could still find my books intact because they were afraid of getting infected. It was like a blessing in disguise

I: I’m sorry about that. I am just wondering at that time when your desk mate moved and there was space, didn’t the teachers ask?

R: The teachers asked but no one used to talk. I was in a school that students had an upper hand than the teachers. It was a private school and the Director told the students to always ensure they were comfortable so the teacher could not do much. The teachers were afraid.

I: Oooh, so the teachers were afraid and even if the students did not respond the teachers could not force them

R: Yeah

I: So it was not a big deal

R: It wasn’t a big deal. The teachers could ask me why I didn’t have a desk mate and I told them I was also wondering why I did not have one. They also knew I am a funny girl so they did not take it that serious

I: How did you feel about all this

R: During the day when the teachers are around, I would keep up a smile but at night I used to cry. My pillow was ever soaked with tears every other night. Remembering I am waking up to no desk mate, no one to talk to, I am just there alone and I think that is where my urge to write came in. Every time I wanted to talk to someone I would go to the library and reads. I used to write my future and a lot of stuff

I: Okay. How was it then after you finished your form 4?

R: I got poor grades and I decided to go back to form 3 but in another school and another city

I: Mmmmm

R: That is when I came to Kisumu

I: Okay.

R: I joined another school and finished my form 4 in 2017. I decided that here I will not tell anyone about my status but fortunately I melted the heart of the Deputy teacher on my first day in that school. When my sister admitted me she told her that I am sick and I am on ARVs and she should take care of me. The teacher asked me how I felt now that I was on drugs. I told her I was living with it and I am lucky because I don’t have cancer. I think that is what she liked about me and we became best friends so my life was comfortable

I: If you compare the experiences in your former school and this other school

R: It was good. The only challenge was that I was from a private school and this was now a public school.

I: Ohh not private to private?

R: Yeah. The food, the environment, the climate….. Everything just changed. Then you know it’s a school in Nyanza so people talk a lot in vernacular and I came from a school where people used to talk English from then day you open to the day you close school. People did not want to associate with me because of my English and they thought I was behaving the high class way which honestly was not true. That is just me and that is how I speak English. Most often they could speak Swahili when the teacher is not around and at times vernacular. That was the challenge I went through but I had to fit in their shoes and speak Swahili even if I don’t want to

I Wow! Okay. What has enabled you to continue engaging in care here at this facility?

R I just sat down and thought that this is my life and I cannot change my past. It is not my fault that I was born HIV positive and even if I was to choose I could have not chosen to be born HIV positive. I have dreams to fulfill; I have empires to build so I have to take my drugs in order to fulfill these dreams.

I Okay. What makes you continue engaging in care specifically at this facility and not any other? Is it the distance? The staff?

R Staff, mostly staff. How you are handled when you come to pick your drugs

I Mmmm, how are you handled?

R Very well. There is a warm reception and you don’t feel like you are alone in this, we are going to fight it together

I Okay. How about the services offered?

R Services offered are good. You are not frustrated and you are directed politely

I Great. Could you please tell me anything you know about antiretroviral drugs?

R I don’t know if this is a rumor but I have also experienced it. One has the side effects like nightmares that have happened to me and is normally on and off. If you stop taking the ARVs for a while you will be resistant causing treatment failure which will make you move to another lane and adapting to it may be difficult.

I When you talk of lanes what do you mean?

R There is lane 1, 2 and 3

I Do you mean first line and second line ARVs?

R Yes

I Okay

R Moving to another lane also means you will have side effects like headache, drowsiness

I Good. Any other thing you know about the ARVs?

R No

I Could you please tell me about the time you missed taking your ARVs. What happened?

R It was during the stigmatization when I defaulted and I did not want to take them.

I Oooh, it was at that time when you really felt like

R I felt like the world is coming to an end and that is when I decided I will not be taking then drugs

I You stopped taking the ARVs for a period of how long?

R 3 Years

I So the whole time you were in the other school you were not taking your medication but you could attend clinic?

R I would attend clinic just because I used to go home (Laughs)

I Motivation was going home (Laughs)

R Yes. So I used to go to the headmistress office and the moment she sees me she would know that I am going for my drugs. I used to go home in the evening and come back the next day after visiting then clinic

I Okay.

R At least I had that time to go home, meet friends and have people to hang around with a few hours then I go back to school. That is what motivated me to attend the clinic

I Okay. Could you please share with me any challenges you face keeping your clinic appointments?

R I did not face any challenges because I had understanding teachers and even if I could not attend my sister was there for me.

I So, do you currently experience any challenges?

R No, I don’t experience any challenges

I Could you please tell me the last time you missed your clinic appointment. What happened?

R I have never missed any clinic appointment.

I since you started taking ARVs?

R I have never missed any appointment and even if I were to miss, my sister would always go and pick the drugs for me

I How do you think your encounter with HV care services can be made more satisfying?

R I don’t think there is anything that should be done. The services are okay.

I May be you have ideas that can make the experience more satisfying

R I think for my case I am just comfortable

I What makes you comfortable?

R There is always adolescent clinic day and also a day for adults so it’s always comfortable because you have your peers

I How does being in school or college or young people like you affect keeping their clinic appointments and taking ARVs

R In High school it cannot be a challenge because you will get permission to come for your drugs but in college it may not be possible. At times you are out with your friends and you will find it difficult to excuse yourself to attend the clinic. So it will be like a challenge because you are afraid of your friends finding out you are coming for drugs. It will be hard

I Okay. Any other challenge?

R None

I We can now move to sexual and reproductive health. What do you understand by the term sexual and reproductive health?

R I think its how to prevent infections sexually and how to treat sexually transmitted diseases.

I Mmmm, it also deals with safe sex, access to reproductive health information, contraceptive use/family planning, STI prevention and treatment and the like. How important is your access to sexual reproductive health services and information?

R It is important because it makes you aware of the diseases, how it is transmitted and how you can avoid getting it or even if you have how to prevent spreading the infection.

I Where do you currently access information on sexual and reproductive health?

R At the facility

I Any other place you access information on sexual and reproductive health?

R Just at the facility

I Where do you think is the ideal place to access information on sexual and reproductive health services?

R At the facility

I The facility is your ideal place. Why?

R The people at the facility are knowledgeable. They can take you through it and you will understand

I To what extent do you think adolescents and young people like you meet their sexual and reproductive health needs.

R Pardon

I To what extent do you think adolescents and young people like you meet their sexual and reproductive health needs.

R Please explain

I Adolescents and young people often access sexual and reproductive health services regardless of the type of service that they want. Some would want condoms, some family planning and others would just want to get information on how to prevent disease transmission among others. To what extent do these services meet their needs or expectations?

R At some point it does meet their expectations but youths are just youths. They may take part of the advise and ignore the other or others will take the who package. So I think the youths are given direction and I don’t think someone will come here to be misled so it is upon the youth to decide what to take and what not to take

I When you say youths are just youths, what do you mean?

R They do want to take everything that they are told because they already have other ideas from elsewhere. So when they came here and you advise them they still have advise from elsewhere

I Okay. So they decide on which one to use and which one not to use

R Exactly

I Great. What challenges do you as person face when trying to access information and services on sexual and reproductive health?

R The only challenge I have is may be shyness. I am afraid and it’s like how will they judge me (Laughs)

I Do you feel youths are being judged?

R I don’t think they are being judged. If they come to the clinic and follow the right procedure I don’t think they will be judged but if they go for the wrong ones then they will be judged

I Which wrong ones?

R If they seek wrong advise from friends.

I Okay. You have said that you are shy and that is why you are afraid of accessing sexual and reproductive health services. How about when you just want to get information on sexual and reproductive health?

R Still shy

I An example is when someone comes for a demonstration on condom use it necessarily does not mean that they are going to use it, they could just be wanting to know how it is used

R Yeah

I how have you dealt with your shyness?

R I am yet to deal with it

I Okay. Do you think being in school/college for young people affect their access to sexual and reproductive health services?

R Yes it does affect may be because in school they do not have time or they do not have a teacher to teach them on sexual and reproductive health whereas those in college just want to have fun but they can access the services if they want.

I Mmmmmm. So its challenging because while in school you cannot get information on sexual and reproductive health because of lack of teachers to teach you. How about when the service is available like at the clinic here and you are in school, does that affect you from coming for the service?

R If you are in school it will because you want a service or information on a particular matter but you are afraid because you will be judged. In college it shouldn’t be a problem but it is a problem the youths want to just have fun and cannot spare a few minutes to access information or services on sexual and reproductive health.

I Okay. Just to get you right, you are saying that it is challenging while in high school because of fear whereas in college you do not have the time. It is more of an individual’s decision.

R Yes.

I Okay. How comfortable will you be discussing your sexual and reproductive health concerns with a lay female healthcare provider, some one of your mothers age?

R I will be comfortable (Takes long to respond then laughs)

I Mmmm, why would you be comfortable with her?

R (Laughs…) I will be comfortable

I 100% sure

R Yes

I What issues will you be comfortable discussing with this lay female healthcare provider?

R General or?

I Issues in relation to sexual and reproductive health

R I think anything concerning that I will be okay

I Specifically like what?

R The right age to have sex, why I should use condoms and maybe why I should not use contraceptives, family planning and all that, side effects of condoms. I also heard that condom has side effects so we can discuss all that.

I Okay. Where would you prefer such discussions to take place?

R At the facility

I Why at the facility?

R Because it is much safer here

I How safe is it? How about home?

R The facility is safer.

I When you talk of the facility being safer, what do you mean?

R There is privacy compared to home

I Okay. Any other place you would think of?

R No, facility would do for me

I Okay. We are almost winding up. I want us to discuss your views on potential interventions. How would you feel if we assigned you a lay female healthcare provider to be your source of information and confidant for your sexual and reproductive health needs?

R Pardon?

I How would you feel if we assigned you a lay female healthcare provider to be your confidant and source of information on sexual and reproductive health?

R It would be a good idea because maybe I will stop being shy.

I If we assign you this person what attributes would you like to see in this person?

R Kind, humble, someone who listens and is there for you anytime. You approach them and they set aside their work to listen to you

I Mmmmm

R Whatever you discuss is between you and her

I That is confidential?

R Yes, confidential

I Mmmmm, what else would you like to see in this person?

R Should not be very serious about life (Laughs)

I Mmmm, what do you mean by being serious with life?

R There are these people who are usually very serious such that you fear even talking or approaching them. You should have a friendly face and they should be approachable

I How would you feel about receiving your ARVs together with your sexual and reproductive health services at the same point?

R It would be good

I Mmmmm, why would it be good?

R You are killing two birds with a stone (Laughs)

I Mmmmm

R Yes, when I am coming for my ARVs then I have to organize another day for the sexual and reproductive health services. Atleast I will save time when I get them all at once. And it also saves my energy

I Mmmmm. Do you think there are disadvantages of receiving your ARVs together with the sexual and reproductive health services together at the same point?

R No, I don’t think so because you will be saving time.

I Mmmm, any disadvantage

R None

I Great. We have come to the end of our discussion. Thank you vey much for your time

R Welcome

I Any questons in relation to sexual and reproductive health?

R No questions.

I Okay. Thanks

**AYA IDI: KCH 0014**

**Site: KCH**

**Date: 22 May 2019**

**Interviewer: Harriet Fridah Adhiambo**

**Transcriber: Harriet Fridah Adhiambo**

**Version: English**

**Interviewee Details**

Age: 19 years

Sex: Female

Level of education: Completed secondary

Occupation: Unemployed

Orphan hood status: Not orphaned

Sexually Active: Yes

Marital Status: Single

Ever pregnant: No

I Welcome to this interview session. My name is Harriet. Let us begin by you telling me about yourself.

R I am L (name withheld) I am a sister to 3, a daughter and a friend to many. I think that’s all about myself.

I Okay. May be you can tell me about you age, education, marital status and occupation

R I am 19 years old. Just finished my high school and will be joining University in September

I Are you employed or engaged in any activity?

R I am nurturing my talent in music

I Good. Where?

R A place known as my life in Sije. It a place where people nurture their talent particularly in music.

I That is great. You are a musician mmmm. Have you produced any songs?

R Yes, one but it is not yet out. It will very soon

I Okay. Generally, what challenges do you face as a young person

R I have challenges but I don’t know how to tell them because in a way I don’t see them as a challenge and I have learnt to live with them as part of my life. Basically I don’t know if I have stepped on them so hard till I feel I have overcome them. I don’t know like personal challenges mmmm mayabe no.

I Okay. You have said that there are those challenges that you have stepped on hard till they are no longer challenges. Which ones are these?

R Like about HIV, how people view it outside here. The reception of people living with HIV outside here is not welcoming at all

I Mmmm

R Yeah

I Okay. May be we can begin with general challenges that are not HIV related. We will tackle that in the next section. Lets talk about general life challenges

R Life is hard, it is hard to trust. I think trust is one big challenge I have

I Mmmm, it is hard to trust, please tell me more like who? And in what context you find it hard to trust

R Friends, relatives and may be your parents are the only people you can trust. What I have realized in my 19 years is that someone can’t just help you for free you have to pay. Someone can’t just give you something and expect a thank you there must be something in return

I Okay. An example

R Like when someone may be helps you with homework you pay them cash or someone pays your school fees so you pay by doing for them laundry. That was back then in high school and now you are in the outside world and life is totally different from what you expected. People are different, people are hostile, you don’t know how to act but you have to accept because you don’t have anyone to tell you that this is what to expect. Parents are always busy so it’s hard

I Do you think these challenges have affected you in one-way or another?

R Some how they have and at some point lowered my self-esteem to an extent I don’t accept good things that come my way because I have this mentality that you are doing this for something.

I Could you please share an experience with one of the challenges you talked about , like trust?

R I want to be a musician and you know there are several opportunities. You find someone who is being nice to you and when you ask them to link you up so that you can at least get to where they have reached they want to sleep with you. And its not like you are going to sing to earn their money but you are going to earn your own

I When you talk of sleep you mean?

R Have sex with you

I Okay.

R You never see it coming. They start by being nice to you then when you think you are now comfortable then they tell you this is it. Nowadays they don’t even hide, “ You wan this, then lets have sex”. It’s hard because I am not ready for this

I Mmmm, so nobody hides their intention

R No, they don’t. Its not like before, they just go straight to the point even people you look up to, those who can be your parents. That is why I find it hard

I Have you tried to deal with these challenges?

R The only was to deal with these challenges is to just walk out no matter how painful it is. I have realized I can live without music. You have to step down but in reality it pains but you have no option. You are afraid even of talking to your parents about it because you don’t know their reception. You just walk away

I Walking away is your way of dealing with it but again here you love music. How do you handle this?

R I say to myself, yes I love music and thank God it is something that is in me and it is not going anyway. I resolve to work hard and just postpone it as I will do it later. Right now when I join campus I just want to focus on my studies then after that I will do music

I To what extent have you succeeded in dealing with these challenges?

R You know when you tell these people point blank they do not have an option. Where I felt I have dealt with it when they tell me that I will be one who gets hurt instead I don’t show interest in it.

I Okay. How about other challenges that you mentioned where someone expects you to do for them laundry so that they can help you. How have you dealt with that?

R Those are some of the things that have made me to be somehow stronger and learn to do without other things. I first look at it like is it a must? Do I really have to do this? That how I look at things. Is it going to add value to me? Is it so basic? Do I really need it? If it is something I can postpone then I will.

I Okay. What support is available to help you deal with these challenges?

R There is no support. Outside here….no, thee is no support. I have learnt to live with this slogan “ Everyone for themselves, God for us all”. I don’t really see where you can go and air out your problems then you are helped because there its no trust. People can’t just be trusted, there is no where maybe to God. Human being s no, I don’t see

I You don’t have support system anywhere like your parents, friends or relatives?

R I have friends who are doctors but you cannot tell them everything. There is something’s that if you tell someone they will feel like you are a bother. Everyone has a share of his or her own problems to deal with. The ones I can share I always do. As a teenager there are things I do that I do not want my parents to know because they will be disappointed. I therefore share with my friends who are doctors here at this facility.

I Great. We will now move on to HIV care services. Kindly describe your experience the day you were enrolled to HIV care.

R I was 9 years old at that time. I really did not expect it. I used to take some drugs and was wondering why I was taking them yet I wasn’t sick. Whenever I asked my mother used to come up with some excuses like I have flu, and stuff. Finally she told me that I was HIV positive. I was very annoyed at that time and you know, I am one person who get hit by something later and not immediately. When it hit me that I am HIV positive, I started asking questioning myself. I am the third born in my family, how about the first and second born? How about the last-born? Are they too HIV positive? Why me? I asked so many questions but again I realized that I did not have an option, as it was already spilt milk. The only option as to take the drugs fro the sake of the goals I had. It wasn’t easy.

I What was going through your mind at that time?

R I didn’t know much about HIV

I You said you were 9 years old right?

R Yes, I was 9 at that time. I heard people say that If you were HIV positive then it was a curse, you were not loved, a disgrace to the family. By then people did not know much about HIV at least now people are informed. I asked a lot of questions both at the clinic and home but at the end of the day I did not have an option but to take the drugs.

I At the time of enrollment to HIV care, who accompanied you?

R My mother. We are both HIV positive, us only. When I learnt that she was also HIV positive at least I had someone to talk to.

I Okay. So, you mother accompanied you to the clinic the day you were being enrolled to HIV care

R Yes

I At that time of enrollment to HIV care were you told that you are HIV positive and you will be taking your drugs or you were just given drugs?

R I was told that I am HIV positive but I did not know what it meant being HIV positive. I was told I would be taking the drugs for life and this was a challenge to me. I didn’t have an idea and by then there was no HIV education in school.

I So, you just took the drugs because your mum used to tell you that you have flu.

R Yes. I stopped asking a lot of questions when I realized that we were going through the same thing with my mum. I could remain silent and wait to be told that the drugs are over so that we go back to the clinic. When we went back to the clinic, I was told that I have to take the drugs for a lifetime. I was like… really? What am I going to do? At that time I had dreams of becoming a fashion designer and was wandering how I was going to do it. How was my life going to be? Remember I told you that HIV education was not much then. I was worried that my skin, eyes, and body are going to change. At that time when you talked about HIV I could see like it’s someone dying in the hospital, I didn’t know much.

I How do you feel now?

R I am good. I have learnt to live with it.

I Are there instances you have felt otherwise?

R Yeah, I can’t lie, it is hard. Anyway, besides HIV I have things I want to accomplish. I have dreams. Those things that I want to accomplish give me strength and I don’t have an option so I just have to do it.

I What has enabled you to continue engaging in care here?

R What has made it easy for me is somehow the fact that I don’t have an option and I have to do it. I always say that this is how it was planned and it was meant to be. So, I just have to do it. Another thing that encourages me is that I did not get HIV thorough misconduct but was born with it. I always tell myself that may be there is a reason because back at the center I have so many people who look up to me. That also encourages me and I often I don’t want to disappoint them.

I Just to take you back, you said you and your mum are the only ones in your family that are on care. How about your father and other siblings?

R No, they aren’t.

I How many siblings are you?

R We are four

I Okay. Did your mum tell when she started care?

R I saw her in pain and never wanted to ask many questions. She had roles to play. She was not only a mother to me but also to my other siblings, a wife and other things. I started finding out things on my own as the doctors were very friendly to me.

I What else has enabled you to continue engaging in care at this facility?

R The doctors are friendly

I Mmmmm, how about the distance to the facility?

R Given the circumstances, I have to do it. I don’t have an option but it is also not far

I Okay. How about the quality of services offered here?

R Good. I also don’t pay for the services

I Can you tell me an instance where you have experienced stigma?

R When I joined high school nobody told me what to expect. I did my own research and asked my friends who joined earlier their experiences. They told me that there is a lot of gossip and competition among students. When I joined Form 1, I made sure a vied for the position of a dorm prefect not because I was interested but for the sake of my drugs. I became a prefect for 3 years and at least I had control of the dormitory and could ensure students are out of the dorm when I am supposed to take my drugs.

I Okay. Have you ever-experienced stigma?

R Yes

I Could you please share your experience?

R I was also a drama chairperson while in school. I remember this day when we were going for drama competition. I told students in the drama club to carry the bags we were using then one of the girls told me to go back to the dormitory and take my drugs instead of telling people to carry those bags. I was so shocked because I had not disclosed to any of them my status. Remember I told you I have issues with trusting people….

I Mmmm.

R If there was one thing I protected was my drugs. I was strong enough to tell her that I will take my drugs its only that the time had not come. She was equally shocked and I can’t really tell where the strength I had came from…

I How did she come to know that you were taking ARVs?

R I really don’t know but when I was about to finish my high school, I realized some of the teachers knew.

I How?

R Through the school nurse. She used to keep for me my drugs and I could get them in bits. The only challenge was that at times she was not around and my supply was depleted and also going to her every week could raise eyebrows. I decided to keep them for my self. She was the only one who knew my status and therefore exposed me to the teachers and other students. You find that a teacher could come to class and teach about HIV and could look at me through out the lessons so that he/she could see my reaction but I had to be strong. Those are some of the things that I don’t share with people. Maybe I will share with my children in future. There is stigma in schools and it is not a joke.

I Mmmm. was it a planned lesson on HIV or it was just a talk during any lesson?

R Imagine it was intentional so that the teacher could see your reaction and confirm like if it is true. The nurse also shared with the grounds men and the cooks because they used to look at me differently. They wondered why I was always strong and courageous

I Did you experience a typical case of stigma where someone does not want you to do something because of your HIV status? Not necessarily in school

R Yeah. That happened when I disclosed my HIV status to my boyfriend. He started being over protective and our relationship was not even normal. It was hard. He wasn’t treating me well like he used to before I disclosed my status to him. He started questioning why I did not tell him earlier, but you know disclosure is not easy. That is the most painful thing that happened to me

I How did that make you feel

R Trust me, I felt so so bad. That feeling is awful. Come to think of it, this is someone you have been so free with then later the behavior turns out not to be welcoming at all.

I How was he behaving?

R He was in denial. We was pushing me away. His actions could tell because he could not talk about it

I Like what actions?

R He was in denial. So if we could kiss, he thought that he was going to be infected. So he stopped. I would explain to him that he cannot be infected but he never trusted me.

I Okay. So, the moment you disclosed is when your relationship started having issues

R Yes

I You broke up or the relationship is still on?

R I am done with that relationship. It is only that I do not know how to tell him. Every time we argue he has to mention my HIV status. This is someone who takes advantage of me; he cheats on me with other girls and when I bring up the issue, he mentions my HIV status. Trust me it is not easy

I How does he bring up your status in discussions?

R He doesn’t want to accept. He asked me if I could get a child now that I am HIV positive but still did not believe what I told him. HIV education is still not out there as it should be. I think for him he lacks the education

I Have you educated him on HIV?

R I even tried through my friend who is a counselor but he doesn’t want to be exposed. He thinks that if you go to a counselor other people will conclude that you are HIV positive. I have even tried to convince him that we could do it over the phone but he doesn’t want to be associated with anything to do with HIV.

I Mmmmm. Okay. Moving on… would you please tell me anything you know about Antiretroviral drugs?

R All I know is that the drugs are there to prevent your immunity from being lowered, they suppress the virus

I Any other thing you know about ARVs?

R They are they to maintain your condition. They won’t change your status to negative but are there to suppress the virus.

I Tell me about the time you missed taking your ARVs, what happened?

R This is a life long commitment. Once in awhile you wake up and you don’t fell like taking them. When I used to take my drugs at 6 sometimes that time could find me out with my friends. So I could not take when I am with them till I return to the house. I could forget. You know I could not excuse myself from them because they will wonder what I am going to do and I also could not take it with them because I don’t trust people.

I Okay. So you missed taking the drugs because you were out with your friends and could not excuse yourself because you have not disclosed and also later forgot to take when you got home?

R Yes. That was before, nowadays I don’t do things to please people. I will do anything to take my drugs because I know how important they are to me.

I Could you please share with me the challenges you face in keeping your clinic appointments

R Challenges I face?

I Yes

R In the past when I was in primary school, my mum could come to school and ask for permission so that she could take me to the clinic. People started raising eyebrows till I could hide when my mum could come to pick me.

I You couldn’t attend clinic because of

R Fear of people asking why I always shave to leave school to go to hospital

I Okay. Currently do you face any challenges with keeping your clinic appointments?

R I ma doing music and there is a lot of competition in the industry. People look for that one thing that can bring you down. When you come to the clinic you fear that someone will see you.

I So you don’t come?

R I come but I have mixed feelings about it because of fear. I have to come for the sake of myself.

I In the recent past when you missed your visit, what happened?

R When I missed?

I Yes

R It didn’t even go past a day then doctors called. They were curious to know what had happened. But I told them I was not around but could come to the clinic the next day

I Okay. What are your general feelings about the HIV care you receive here?

R I feel contented. Remember I told you that I don’t pay for them, I’m just contented, I don’t have a complain.

1 Okay. Does being in school/college for young people and adolescents affect their keeping of clinic appointments?

R Yeah, it does

I How?

R Peer pressure. It is not a joke. You know the youths are confused, they do not know what they want in life. It is not easy to find a youth who is straightforward. I think that affects because you are afraid of what people will think about you. I still feel HIV education is not enough. As much as those who are HIV positive are taught, those who are negative should also be taught

I Okay. How does being in school/college also affect them taking their ARVs?

R For example we are in a hostel and my time for taking medication has reached and my friends are here conversing and enjoying the conversation. I will not be able to excuse myself because they will start asking questions. So you it affects taking my ARVs

I Okay. Any other thing?

R No.

I Great. Lets move on to sexual and reproductive health. What do you understand by the term sexual and reproductive health?

R I think it deals with sex itself, the diseases, and contraceptives.

I Mmmm and even access to access to Sexual and reproductive health information. How important is your access to sexual and reproductive health services and information?

R It is important to me because I know what to do and what not to do.

I Currently, where do you access information on sexual and reproductive health?

R When there are events like at Agakhan Hall, road shows. Remember I told you that I don’t trust easily so I have people whom I can get the information from. Like the doctors whoa re my friends.

I So you get information from the doctors at the facility?

R Yes

I How about the events you have talked about. What type of information do you get?

R Family planning

I Okay. Where is the ideal place/location where you would like to receive information on sexual and reproductive health and services?

R If I were told to choose, my ideal place would be events.

I Why events?

R For events people would come first of all to look nice but most importantly no one will know whether you are HIV positive or negative. Again where there are events, trust me youths will turn up in numbers.

I Okay. When you talk of events, which specific ones?

R I often go to see the performances. The last one I attended was chaotic though but most of them are organized by several organizations.

I Okay, so you think when you want to give information on sexual and reproductive health services, the best would be events?

R It depends on which people

I Mmmmm

R For the youths evens would be the best because when they learn that there is one, they will dress up nicely to meet people and that would be the best time to give them information on sexual and reproductive health.

I To what extent are the sexual and reproductive health services offered to young people like you meet their needs and expectations

R To me, it is not enough

I Mmmmm, so you feel it doesn’t meet their expectations?

R I feel like there is some information that we need to know. Nowadays parents don’t give us information, they fear so we end up getting everything we need from technology and stuff. Maybe they think we know much than they do.

Trust me, parents don’t talk about sex education with their kids any more

I Okay. What challenges do you as a person face in trying to access sexual reproductive health information and services?

R There are places if you go you can access the information free of charge, but I what I fear is what they are going to think of me. As I enter the health center what is at the back of my mind is how are these people going to view me…

I Apart from fear, what other challenge do you experience while accessing sexual and reproductive health information and services?

R Some of this sexual and reproductive health information like for example on contraceptives/family planning the more you learn of them the more you would want to mess any how because they are available.

I What do you mean by the term mess?

R Mess means having sex. My friend’s say that you can have sex anyhow you want since the contraceptives are available. The contraceptives are good but they also influence negatively.

I So they would prevent pregnancy by using the contraceptives….

R Yes and forget about protecting themselves from HIV. The worst is that the parents are the ones who take them for the family planning methods and that means that they are aware of their sexual lives.

I You earlier said that when young people are trying to access information on sexual and reproductive health, the challenge they experience is that their parents are no longer giving them the information on sexual and reproductive health

R Yes, they don’t at all

I So, that means they get the information from the facilities or at the events?

R Mostly at the events.

I Okay. How does being in school or college or young people like you affect their access to sexual reproductive health information and services?

R Most people think of the reception when they go for these services. What will people think of them? Or you talk to a counselor or someone at the clinic about it then that person exposes you. Like for me, I trusted the nurse in school but she disclosed my status to people. Those are some of the things young people fear

I So, how does being in school/college affect their access to sexual reproductive health and information and services?

R For example if I go to the clinic and meet my relatives or my friends get to know that I went for those services, what will they think of me? Will they think that I am sexually active? They will start judging me.

I How confortable will you be discussing your sexual and reproductive health concerns with a lay female healthcare provider, someone of your mothers age?

R Someone my mother’s age?

I Yes

R Yes, I can be comfortable but again, I don’t trust. You see, when someone looses trust in people it means that they have gone through a lot with that. I don’t know even why I am talking and explaining this to you it could be just a miracle that I trust you. (Laughs…) Its not easy to really trust people but I know someone wants to use this information.

I (Laughs..) Let me give you an example. Like today it’s your clinic day and there is a female/lay person not necessarily a doctor or nurse but someone trained and has experience on sexual and reproductive health. How comfortable will you be discussing your sexual and reproductive health concerns with her?

R No, maybe an open place like at the events. At the events people are free and if they want to give youths information and sexual and reproductive health then I would advise they use events and create a good rapport with them.

I Okay. I know you have said that you can’t trust because of the experiences you have had with people. Suppose you have this female or you are assigned one, what issues will you be comfortable discussing with them?

R I can ask about these contraceptives and just confirm some myths.

I What myths have you heard about contraceptives?

R They make you delicate when you want to give birth, you are fragile. I hear them from my friends who have used before and those that are using. You cant be hit by someone, you are just too weak

I When you talk of being hit by someone, what do you mean?

R Like you are weak/ not healthy. You have to be treated with care

I Mmmmm

R I am already taking ARVs then again I add contraceptive? No, to me that is just stressing. Those are the things I would like to discuss with that person.

I Okay. Where would you like those discussions to take place?

R In a health center

I Why health center?

R There is no other place. Obviously, this is where you will get all the information. This is where we are provided with health related information and services.

I Okay. We are winding up. If I may ask you again, how would you feel if we assigned you a female lay healthcare provider who will be your confidant and source of information on sexual and reproductive health?

R Its okay. Just personal?

I Yes

R First I have to be friendly to that person then she can earn my trust. That’s a very good idea though I have to earn the trust first before I start asking them everything

I Okay. What attributes would you want to see in this person?

R Someone who tells you the truth and does not hide anything from you and also does not tell you things to make you happy or feel good. Someone who calls a spade, a spade and not a big spoon.

I So, you want someone who is truthful and tells you when you are both right and wrong

R Yes. You rather tell me the painful truth than lie. I need someone who is down to earth.

I When you talk of down to earth, what do you mean?

R Someone who is humble and not a slay queen. (Laughs..)

I What do you mean by slay queen?

R You don’t know?

I Yes (Laughs)

R I need someone like you who does not know who a slay queen is (Laughing)

I Okay. I just needed to understand you definition of that

R I know you are lying and you definitely know who a slay queen is. Okay, I don’t need someone who concentrates on these earthly things. I need someone who has gone through that and has the experience and is honest.

I Apart from being honest, want else would you want to see in this person?

R I need someone who has the experience, understands and has messed and made mistakes. For example, myself I know the pain and I have gone thorough a lot.

I Mmmmm, great. How would you feel about receiving your ARVs together with sexual and reproductive health information and services at the same point?

R At least, that would be a relief. That means I will take them where I receive my ARVs and you know what, that the place I have built my trust in over time.

I What advantages come with receiving your ARVs together with sexual and reproductive health information and services together?

R I don’t have to go and look for those services somewhere else. That means meeting different people and I have to build the trust again.

I Any other advantages?

R I can access them any time I want and they are available.

I Any disadvantages you would think of when you receive your ARVs together with sexual and reproductive health information and services at the same point.

R I think that can come later when I start receiving them together.

I You can’t for see any challenges?

R Okay. Maybe I ask too many questions concerning my sexual life and then one time my viral load goes high, they will say that I am concentrating so much on sexual matters and not my health. Maybe that is what can come up

I Okay. It was a nice discussion we had. Thank you very much for being open and your time. Any questions?

R None

I Okay. Thank you.

Name of study: AYA

Type of Interview: IDI

Date of interview: 5-Jul-19

Location: Kisumu County Hospital

Name of interviewer: Harriet Fridah

Name of transcriber: Harriet Fridah

I Okay, so welcome to this interview, my name is Harriet and we are going to discuss on access to sexual and reproductive health services among the adolescents, so we begin by you telling me about yourself, in terms of age, education, marital status and current occupation

R Mmh, I am XXX and I’m 21 years of age, I am single and my occupation, as at now I am acting as an adolescent peer leader at this center, KCH and my educational level is college

I Do you have both parents?

R Yeah

I Are you sexually active?

R Yeah

I Then, have you ever impregnated someone

R No

I Okay, that is fine, so what challenges do you face as a young person?

R As a?

I Young person

R Challenges when it comes to?

I Just general life challenges

R You know when it comes to challenges you need to be specific to some extent

I Like which one?

R Like there are some emotional challenges,

I *(interrupts)* yeah, any, any challenge that you face

R *(interrupts)* psychologically

I All challenges be it emotionally, psychologically

R They are quite many

I You can tell me a few

R *(laughing)* so like specifically the challenge I face as per now as much as my age is concerned, is lack of support from my dad and also psychosocial problems. My health is not all that good because I am HIV positive and epileptic. The challenges I face normally affect me psychologically. You know like if you are a father, you are expected to take care of the family and be with them closely. My dad, for now he is just here in town and my mum is at home and he doesn’t even send upkeep money and you know with this little salary that I get to some extent is hard to cater for family needs and personal needs so it is quite a challenge for me and whenever I share with him he says he will see on what to do about that.

I So do you stay with him here in town?

R I can’t say that I stay with him because he doesn’t even come to the house

I So he has another house somewhere?

R Yeah

I or another family

R Yeah

I Okay, so your mum is at home?

R Yeah

I and your other siblings?

R I stay with my other siblings

I So you are the one who takes care of them?

R Probably

I He doesn’t support or even come or?

R He doesn’t support unless I tell him that I’M tired

I How many siblings do you have?

R Like for me there is another sister who is in Mombasa who we help each other, and another young sister

I She is in school?

R Yes she studies here in Kisumu, Xaverian Primary School. My elder sister and I have to cater for all her school needs.

I So you are the one who provides food and transport money? How about school fees?

R It is a public school

I Oh yeah, no paying school fees.

R So when it comes to school fees we don’t have much burden being that it is a public school. My mum is not financially stable, and she is at home so you don’t expect someone who is not financially stable to come to help you yet you are the one in town but sometimes when she harvest her maize she brings it to support us

I Okay. You earlier told me that another challenge you face is related to your sickness, being epileptic

R Yeah

I Okay

R Like in most cases it affects me if I have stress

I So the epileptic episodes come in when you are stressed

R Mostly

I Mostly?

R Yeah

I and how have you handled these challenges like your dad not supporting you?

R I decided to leave him so that he could do whatever he felt is good. As a son, I tried talking to him as much as I could but still there was no improvement.

I You said he has another family; does he have children?

R Yeah

I How many?

R He has like four

I Maybe he feels that you are working and you should also take responsibilities

R I have even told him that as much as I am working, he cannot expect me to provide for the whole family, because I also have my personal needs that I have to take care of, like my college, you know I have done like 2 courses, first I did social work and the second was electrical engineering so you see these things, there is even one which some person helped me…..

I Helped you pay the fee?

R Yes, but for the electrical engineering I pay with the small money that I get

I So you go to class in the evenings or weekends?

R Evenings

I Oh evenings, you just pay for yourself?

R Yeah

I and now when you tell him, and he doesn’t do it, what other way do you use to solve this issue?

R Mmh

I How do you deal with it like say when you call him for example you need money for upkeep, and he tells you he doesn’t have

R Yes, he says he doesn’t have and there is nothing you can do about it because even if you go personally to him where he works at the stage, he will say he doesn’t have money. You know he is a manager, there is a group

I (Interjecting) SACCO?

R Yes SACCO and he is the manager, and for me a person like that cannot lack money even if it’s not enough to buy food, he can’t lack

I but he says he doesn’t have?

R As always

I How about your mother? Does he support your mum who is at home?

R No

I He doesn’t

R He doesn’t, to some extent they disagree and even go days without talking to each other such that he even doesn’t go home

I He doesn’t go home?

R He doesn’t

I Totally?

R The only time he goes home is if there is a burial at home which he is supposed to attend

I but now the family that he has, has he built for them a home?

R No, because my family is the first family and my mum always see that since he does not provide, there in no reason of another family coming to our compound

I Mmh

R So you see it brings something like conflict

I *(interjects)* conflict?

R between the two families and that brings challenges like the psychological ones, like for me I think the electrical engineering is the one which boosts me

I You have already finished?

R Yeah, I have finished grade 1

I Ow, it is in grades?

R Yeah, I have finished grade 2 and 3, I am now remaining with grade 1

I What is the difference, I thought you should start from grade one to grade 3

R Grade 3 deals with installations like house installations while grade 2 deals with machines, grade 1 deals with supply

I So how long is the course supposed to take

R One per year

I One?

R One grade per year

I Ooh so you have one left

R Yeah

I Where do you study?

R Kisumu poly

I Ooh Kisumu Polytechnic?

R Yeah

I Okay

R So that is what helps me, you might have someone call me to do wiring jobs

I Okay

R So I create the quotations and they give me money

I Okay, so that is what you use to support yourself and sister

R *(Interjects)* but I normally focus during weekends because weekdays I’m at work

I Okay, so these challenges, you have said they have affected you, have they affected you academically, professionally even in your socio-economic advancements?

R Yeah

I How?

R First of all they have affected me economically. I always feel like if my dad was able to provide for the family I could be using the money I get for my own needs only, and with these I could have been far, because if I see where my classmates are right now, I get depressed more and feel like the odd on out.

I How about educationally?

R Educationally also, right now I could have been done with the course or grade but I haven’t because sometimes this year I was expected to pay ten thousand shillings for registration but I couldn’t.

I for exams registration?

R Yeah for the exams

I You do the KNEC (Kenya National Examination Council) exams?

R Yeah, at the end of every session you do a KNEC exams so that you proceed to the next grade

I So if you don’t excel you repeat?

R Yeah

I Okay

R so, there was money which was required for exam registration and it was fifteen thousand so when I check my salary is only eight thousand and beside the eight thousand there are also some needs that I am supposed to cater for, so you see

I Yes

R So, I had to look for money, but I did not make it so as I was saying if my father could have supported I could have managed to do the exams

I Yeah

R Coz I could have saved for two months to have sixteen thousand

I So, you did not do the exams at that time?

R I did not, though it was in 2017 that I experienced that challenge but last year is when I started looking for money and I was able to pay and sit for the exams

I and since your father doesn’t support you, there is nothing much you can do since you are the one supporting your family too.

R Yes

I How about these other challenges? Like when you are sick?

R When I am sick I always feel like those are challenges that someone has to face because there is nothing you can do you just have to cope with it, like in my epileptic situation. I got help because before I started this job I used to do voluntary services at Lumumba. One of the Fridays as we were in a meeting I got an epileptic attack due to the stress I had. One of the clinicians who knew my situation was looking for a way of assisting me. So in the next meeting she addressed the team that there is a colleague who has challenges and the team should discuss on ways of supporting him. She did not specify during the meeting who was to be supported and requested them to see her in person if they were interested. I didn’t know that she was referring to me till when she called and informed me that she was referring to me during the team meeting. The team discussed and I was linked to the medical outpatient clinic in Jaramogi Oginga Odinga Hospital where I do attend my epileptic clinics.

I You mean KCH

R Yes here in KCH, so once I am prescribed a drug, they give me

I The Lumumba team gives you?

R Yes

I Those drugs are not here in KCH?

R They are there its only that they are charged so the Lumumba team buys for me.

I Okay.

R Yes

I That is good, and what support is available to help you deal with these challenges

R Mmh

I What support is available to help you deal with these challenges, you have talked of a clinician who helped you get drugs for epilepsy, how about financially, do you have support?

R Not really

I You had mentioned your sister, how is it with your sister?

R My sister only comes in when I am unable and acts as the last option

I So, what does she do?

R She works in a hotel

I Hotels in Mombasa?

R Yeah

I So once in a while she can support

R Yes

I What about when you are stressed, do you have someone to talk to or people to share your problems

R When I’m stressed, I normally don’t like sharing my problems with any other person unless I know you very well from A to Z. If I don’t know you well you can share my problems with somebody else and then it spreads and adds more stress

I That is true

R Yeah

I and just like a friend somewhere where you can confide issues or even your mum

R My friends no, but sometimes I call my mum, but you know I don’t like to call her frequently because if I tell her my challenges, she will be even be more stressed and that is what I am trying to avoid.

I Okay, so let’s now move to HIV care services, so would you please describe to me your experience the day you enrolled into HIV care?

R I was enrolled in to HIV care in 2006

I 2006?

R Yeah but I don’t know the specific dates, but it was in 2006

I Okay, so who accompanied you?

R My mum

I Oh your mum?

R Yeah

I and how did you make the decision to enroll

R by that time I did not know anything about registration, it was my mum who did that

I It was your mum

R *(interjects)* because by that time I was like 12 years

I Okay

R 12 years

I and you did not know about HIV care

R It was just that I started feeling sick so one night the sickness worsened to the extent that I was shivering so that is when I was brought here and was admitted in ward 2. I remember it very well since I spent two weeks here. So after the two weeks, I started taking the drugs without knowing the reason why I was taking them. The point when the questions started coming in is when I was already feeling better yet I was still taking the drugs.

I Okay

R So, I was asking myself why yet I was fine, because we used to come with her here

I You come with her to the clinic

R Yes. I am very sharp boy. So one day, I heard the doctor asking her if she had told me why I was taking the drugs, and she said no. The doctors asked me if I knew why I was taking the medication, and I told him that I was sick but got well so I did not know why I was still using the medication. At that time I was 13 years, so the doctor told my mum to go and tell me why I was taking the drugs, but still she did not tell me, she feared disclosing due to the challenges that we had, so we were coming for support groups and most of the topics which were being taught were HIV so I was wondering why HIV or I was a victim and I did not know, so at that point I concluded that maybe I was positive and I should just accept my situation if my mother did not tell me

I and did you ever ask her?

R Yeah, I asked her, there is a time we came to the clinic together and the doctor asked again if I knew why I was taking the drugs and I told him that I did not know, so he took us outside so that my mother could tell me. That is when she disclosed, even though it did not affect me that much because I already knew and had the clue.

I You knew because of the support group you were attending?

R Yeah, I was just waiting for a confirmation of the same.

I So, she did confirm?

R Yeah, she confirmed it and I had no option but to continue with my medication

I What went through your mind at the time she disclosed to you?

R Many things went through my mind like on that day it was about 11 or noon thereabout and from that time my moods totally changed like for a week I was just moody

I So what was going on in your mind?

R I was asking myself why me because I am the only one taking medication in our family

I Are you the first born or the las born?

R I am neither

I Okay. You are the second, you have an older and younger sister?

R Yeah

I Okay

R So I was asking myself how I was born and I still ask myself till today

I What about your father, is he on care?

R Yeah, my father is on care

I Okay, and how do you feel how you knew about your status or is there a better way you could have wanted to know about your status?

R There is no other better way that I could have learnt apart from how I learnt

I Okay

R because as according to me it is good to learn things by yourself so that it does not disturb you rather than having someone tell you.

I So, what has enabled you continue engaging in care at this facility?

R Mmh, pardon?

I What has enabled you continue engaging in care at this facility?

R In this facility or generally?

I or generally

R because when you find yourself in a situation, you just accept and move on because I always ask myself that if I give up in life, who will take care of my younger sister?

I and where do you take your drugs from?

R Here

I Here?

R Yeah

I What makes you continue coming here, is it the staff, the distance from your home, forget about the days that you work here, just you coming for care, what makes you come here specifically?

R Specifically, I think it is the relationship with the workers

I and how is the relationship?

R because I know when I go there I will get someone I know, and I will have courage

I So you are saying your relationship here with these people is good?

R Yeah

I Okay, and what about the quality of the services being offered?

R The quality is good

I the staff

R They staff I can’t say they are good or bad because I have not interacted with all of them so I can’t really know who is good or not

I So can you tell me an instance where you have experienced stigma

R Yeah, I have experienced stigma (laughing)

I When, where and how? *(laughing)*

R Like first in school

I High school or college?

R In primary school, when I was in class 6 I was infected with TB and you know with TB I had to come for check up every morning and at that time it was very tricky. Getting permission from the teacher was very tricky so I used to ask for permission and go back to school. One day I went to seek permission from some male teacher and he was our science teacher so there was a day he was teaching a specific topic on STIs and he was talking basically about HIV so it reached a point where he said those with HIV go to the hospital frequently, and referred to me like XXX goes to the hospital regularly.

I *(Interjects)* In front of students in class?

R Yeah

I and what was the reaction?

R People just looked at me but from then I never liked him

I (Interjects) and how were the students now treating you

R Not that good, I just had to bear with the situation and finish school

I and later on did you go back to him and tell him you did not like it

R I just felt that will just be foolish of me

I How did you feel about that

R I felt bad, but I just went and kept quite.

I Sorry, tell me anything you know about ARVs

R Antiretrovirals?

I Yes

R They are used to suppress the virus, they are used for immunity and are used by people infected by HIV

I Any other thing?

R I think you need to be specific

I No, just general

R Just the ones that I have given you

I Okay, now tell me about the time you missed taking your medication, what happened?

R The time I missed my medication?

I Mmh

R I remember only once when I left work late and when I got home I found my sister was sick

I Your younger sister?

R Yeah and it was a critical condition, so I had to bring her to the hospital for treatment and go back with her, so on bringing her to the hospital she was admitted for that night and no one was there to stay with her.I only remembered the following day that I did not take the drugs on that previous night, and I felt like ooh noo

I What did you do after realizing you missed?

R I went and took the one in the morning

I and do you have challenges in keeping your clinic appointments?

R In keeping my clinic appointments?

I Yeah

R No

I Okay, and have you ever missed your clinic?

R Yeah

I What happened when you missed?

R In had an exam in school and my mum was also in the village. She is the one who always come for them when I am busy and if she is around. Unfortunately that day she was in the village.

I So your mum also did not come for the drugs?

R She couldn’t manage

I So you missed your clinic visit for how long?

R 2 days

I for two days?

R Yeah

I So the next day you were able to make it?

R Yeah

I So what are your general comment on HIV care service that you are currently receiving?

R My general comments are that they are good, but I think there are some parts which need improvement

I Like which ones?

R Like when it comes to adolescent care, nowadays adolescent care in not that good as per my thoughts, because you find that they only have support groups, once in a month and in this other groups they engage with one another and they meet often. Before we used to be given transport but nowadays we are not given, another challenge we are only provided with soft drinks in the support group and some of us come hungry therefore cannot survive on it only, but before they could give us snacks

I Okay. How do you think your encounter with HIV care services can be made more satisfying?

R Can you elaborate more?

I How do you think your encounter with HIV care services can be made more satisfying?

R I think it can be more satisfying if this support group can be held like twice

I *(interjects)* a month

R Yeah instead

I *(interjects)* instead of once a month

R Yeah, like twice in a month can be favorable

I Support group twice a month what else?

R Transport thing

I *(interjects)* if they reimburse transport once they come

R Yes it can be favorable and can boost many adolescents turning up since it is a challenge

I So transport should be provided

R Yes and also for the young kids to be given porridge, maybe the parent is not well of and might stay the whole day here hungry

I So giving them transport will make the experience more satisfying?

R Yeah

I and even giving a snack or drink as they wait?

R Yeah, exactly

I How does being in school or college for young people like you affect their clinic appointments and even taking ARVs?

R because the thing I can say for those in schools especially in high school are experiencing a lot of challenges because taking drugs is hard and also taking the drugs is a problem since they don’t want to be seen because of stigma. The other one is if you are in a boarding school and you are given appointment of one month, coming to the hospital might be a challenge

I They are not given permission

R They are not, to some

I How about those who disclose to their teachers

R they can also experience stigma and that’s why some fear telling their teachers

I So those in school are not able to come because they have not disclosed and taking drugs is also a problem

R Yes

I Any other thing

R I think…

I It is just that?

R Yeah

I So being in school can affect how they take their drugs and how they attend their clinic?

R Yeah

I Okay, we are moving on well so what do you understand about sexual and reproductive health?

R STI

I STI, what else is part of sexual and reproductive health?

R It deals with sexual and reproductive issues

I Issues such as, as you have said STIs, family planning, things to do with condoms use, safe sex, prevention of disease transmission among others. How important is your access to sexual and reproductive health services?

R Individually?

I Yes, you how is it important to you?

R I can say that it is important because you can know how to go about it when they say you have STI, it has created knowledge on STI like I know the symptoms, so I can come for consultations

I Okay. Currently, where do you access information on sexual and reproductive health?

R At the health center

I any other place a part from the health center?

R No

I Only the facilities, how about the services?

R at the health facilities

I Where do you think is the ideal place to access this information

R Health facilities is the best place

I Why do you think so? And maybe not home or school?

R In a health facility you are sure of getting it, you know when it is taken to schools like the teachers to be providers, some teachers will not allow you to access them since they will say you are not eligible due to your age but maybe you know the reason why you want it.

I To what extent does the sexual and reproductive health services offered to young people like you meet their needs and expectations?

R It does meet, mostly to some girls who are in school like you will find that they engage themselves in sexual activities but once they get these services it will help them not to get pregnant

I Like for family planning?

R Yeah

I Okay, and what challenges do you as a person face when trying to access sexual and reproductive health services and information

R I don’t face challenges because if I need the information I will get them at the right time

I Do you think other youths face a challenge?

R Other youths?

I Yes

R Yeah, they can due to lack of information, and also due to lack of care

I and how does being in school for young people like you affects their access to sexual and reproductive health?

R That is very tricky, I think when you are in school teachers won’t allow you to get these services

I So they don’t get permission

R Yes

I What else?

R Lack of enough information

I Lack enough information?

R Yeah

I but maybe they are informed but they are in school like college

R If they are informed and in school, maybe just the lack of permission from the teachers

I Any other thing?

R Just like peer influence, maybe they have heard their friends talking badly about something

I Peer influence

R Yeah

I What else?

R I think only that, I don’t have any other

I how comfortable will you be, discussing your issues in regard to sexual and reproductive health concerns with a lay female health provider, someone of your mothers age?

R I don’t have a problem

I You will be comfortable?

R Yes, because I understand that an elderly person like my mum has that information about things I need

I What kind of issues will you be comfortable discussing with her?

R With?

I with that lay healthcare provider

R just things to do with sexual and reproductive health

I What specifically?

R (laughing) just sexual and reproductive health because other issues for me is tricky

I but now when you look at sexual and reproductive health, what specific issues will you be comfortable discussing?

R Maybe if I have a problem with my partner

I What else?

R Maybe if you see anything that warrants questioning about STI

I Where would you like these discussions to take place

R In a health facility

I Why health facility?

R If you get a nurse you are sure of the response

I How would you feel if you are assigned a lay healthcare provider to be your contact person and confidant and source of information on reproductive and sexual health when you come to the clinic? someone who can share your secrets and advice you on different things, how will you fill about it?

R There is no problem, as long as she abides by my rules

I Which rules are these?

R Confidentiality

I So you would want someone who is confidential

R Yeah

I what attributes would you like to see in them? What characteristics would you want to see in this person?

R a person who is friendly, understanding

I a person who is confidential, friendly an understanding?

R Yeah

I Okay, how would you feel receiving your ARVs together with your sexual and reproductive services at the same point

R There is no problem

I Okay, so what are the advantages of you receiving your ARVs and sexual and reproductive health together at the same point?

R It reduces a lot of movements

I Do you think there is a disadvantage of getting all these on the same day and point?

R No

I and what interventions would you propose to improve access to adolescent and young adults to sexual and reproductive health services

R First of all you provide these service in youth friendly center

I In a youth friendly center, what else?

R I think that is the main point

I any other thing you would want done?

R and then to the person who is to provide these services needs to be a youth

I Youthful?

R Yeah

I Why youthful

R because you get that approaching an elderly person with an issue might sometimes be hard and tricky and opening up is hard but to a youthful person is easy

I What else

R I think that’s all

I Thank you very much for your time, any question?

R Where are you taking this information as much as you are recording them?

I As I had told you, when you went through that consent, they are going to be transcribed, written then coded, then this information will be used to improve access to sexual and reproductive health among adolescents, so that is why we are interviewing adolescents, we are interviewing around 36 just to know what they want in order to improve the sexual and reproductive health, so it is coded like XXX said, even your name is not going to be used in the script. yeah

R Then after that?

I After that, if an intervention comes then it can be implemented based on the findings, yeah

R Its okay

I Okay.

Name of study: AYA

Type of Interview: IDI

Date of interview: 4-Jul-19

Location: Lumumba Sub-county Hospital

Name of interviewer: Harriet Fridah

Name of transcriber: Harriet Fridah

I Okay, so welcome to today’s discussion, my name is Harriet; we are going to start our discussion as I had earlier informed you. Just before we begin, I would like you to tell me about yourself.

R Myself?

I Yes, be a bit louder so that we are able to capture in the recorder well.

R *(noises from moving chair)* okay

I Mmh, yes

R Me I love myself

I Mmh

R Me I love girls, I love to play football, I love food

I Okay, that is good, maybe you could tell me about yourself in terms of age, what is your age?

R 16

I 16 years old. Level of education?

R Education

I Yes, you are in which grade? Or *(interrupted)* class?

R Eight

I Okay, so currently you are a student?

R Yeah

I Are you sexually active?

R Active?

I Have you had sex?

R Yes

I Okay, and have you impregnated someone?

R No

I Do you have both parents?

R I don’t have a father

I He died?

R Yes

I Okay, thanks. I want us to begin the interview, what challenges do you face as a young person?

R If you go home, your mother starts beating and asking you where you are coming from

I Just talk in a language you feel comfortable in, either English or Kiswahili, any is okay

R She asks you where you are coming from this late at eleven at night, I tell her I’m from fetching a book from a friend, then she asks what I’m doing at people’s homes late at night yet I am supposed to be reading. So I tell her I went for the book to assist me complete my homework so that I return it tomorrow. So, she warns me that if I stay out late again the next day she wont allow me back in, ill have to spend the night where I was. But I always tell her that I spend time with my friends so that I don’t get bored. She insists that even if it is a matter of meeting friends, why don’t I see them during the day say at eleven or noon, so that by five or seven in the evening I’m back home but she did not like that I was staying out till late, she warned that something bad might happen to me on my way back at night. I told her that I will maintain coming back at nine at night. She said that if I will maintain at nine is okay, she just didn’t want me coming back at eleven or midnight

I Mmh

R So I try coming back early nowadays

I So the challenge is the arguments from your mother?

R She only argues if you have done something wrong

I Okay

R But if you have done nothing wrong she does not have issues

I So apart from the arguments from your mother when you came back home late, what other challenges do you go through as a young person?

R interacting

I Mmmh

R Maybe if I fight in school, because I don’t like it when someone makes me annoyed, if they do I wont care who they are, I’ll beat them up and make sure they bleed. I don’t like being insulted, talking about my mother or about my family or even about myself or my mother, so that anger is so bad that it can make me kill someone.

I So, having an uncontrollable anger is also a challenge to you?

R Yeah

I Okay, any other?

R No

I Okay, only that. So, do you think this challenges have affected your education?

R No

I Kindly explain

R It has never affected my education

I *(interjects)* It has not affected it

R Yes

I Like now that you have said being angry, or being high tempered, has it affected your academics in any way?

R No

I Okay, how about…you said your mother quarrels you when you come back home late, has that affected you in any way?

R It has never affected me

I Mmh

R You know she is my mother so if she tells me anything, I’ll have to do it. If she tells me not to do bad things I won’t do them, if she tells me to do good things which will helps us, I do them

I Okay, and, so have you tried dealing with these challenges like now being high tempered

R I decided and even sometimes I ask myself why I’m so high tempered, I as myself why I’m like that, I try not to fight but I don’t know what happens, even the teachers complain about me

I How have you tried solving that problem or issue?

R My mum told me that if I feel like someone has wronged me, I should walk out of that place and sit somewhere else or not to talk to anyone until the temper goes down

I Okay, so that is what you always do?

R Yes

I Okay, what about the challenge of being in confrontation with the mother because of coming in late, how have you dealt with that challenge?

R Nowadays I come back home early

I Ooh you come back early

R Yes

I Any other thing?

R No

I So you think you have succeeded in dealing with these challenges?

R Yes

I Okay, to what extent?

R You know, the way I like reacting to things, so I want to be someone who does not have a lot of stories or conversations because my friends always ask why I like getting angry and I told them that there are some things I don’t like hearing and might make me angry so fast so nowadays I like joking with people

I Okay, and what support is available to help you deal with your challenges?

R My support is, I like being close to people so that it helps with my temper so that when I am there the only thing I can think of is how I’m going to budget for my lunch. Someone comes with their stories such that help me with fifty shillings to enable me buy three *chapatis* and beans stew, everyone minds their business. Others come with problems such as requesting for help or asking for bhang to smoke, but I can’t buy bhang, I ask them to ask for things like tea and bread but not bhang

I Okay, so, like you have said your challenge has been being high tempered?

R Yes

I I just wanted to find out if you have anyone who has supported you through that challenge

R Yes

I Who is that?

R My sister, she is the one who always tells me that I have high temper

I *(interjects)* Kindly hold on a bit...okay

R My sister always tells me that every time someone disturbs me I should tell them to look for someone else to disturb and not me, I like my things to go slowly so if you tell me to do different things quickly I won’t do that, if you tell me to bring water and mop, I won’t do it. So you have to let me finish my homework so that I can do the work later, I am the last born so they expect me to do everything, so I tell them that they know my reaction and behavior and my temper and you know I can do harm and be arrested and I don’t want to get arrested so I don’t like fighting but if you wrong me I’ll fight you

I Okay, so your sister is your support system?

R Yeah

I Okay, and apart from being high tempered being a challenge as a young person or as a youth, are there any other challenges that you go through?

R No

I You find that in some cases we have people who have relationship issues, there are those who have issues with the parents, you don’t have any other challenge?

R No

I Okay, good. So, I want us to move to HIV care services, and I just want you to describe to me your experience, the day you were enrolled in to HIV care, like how long did you take after knowing that you are HIV positive? where were you tested? How did you make that decision to be enrolled? What was going through your mind at that time?

R That day I was 5 years, I was told by my mum

I You were five years old?

R Yes, but I still didn’t know that I was positive

I Okay

R So I used to ask my mum why I usually go to the hospital all the time and she told me that I had malaria, when I was seven years, I asked why the malaria was not ending and she told me I had TB

I Okay

R So I asked the doctor when I was 10 years old why I came to the hospital every Friday and the doctor told me that I was positive, I told the doctor I was okay and was not positive but he told me that I had HIV and I said okay. I went and told my mum that the doctor had said that I was positive and my mum kept quiet, so when I turned fifteen years I knew my status and now it does not bother me. So nowadays I like putting my medication in the drawer so that when my friends come, they like taking my flash drive which contains movies, so that place is where I keep my drugs, so when I came home from school I found that they had taken my drugs and started asking which kind of drugs they were and who they belong to because sometimes we find XXX taking these drugs and he always tells us that they are for malaria but the drugs have been labeled CTD. When I came home they asked what they were and I told them they are my malaria drugs, and it’s the doctor who wrote the name not me, but they insisted that the tablets were too big to be for malaria, but I told them that the doctor told me that those ones are better of in treating the malaria. They insisted that they look like HIV drugs and even suggested that we all go for a test, but I told them I was okay, but they insisted so we went as friends.

I *(interrupting)* Mmh

R So we went for a test and I was found to be positive so one of my friends told the others not to use my stuff or share things with me. But one of my friends advised me to continue taking the drugs and that it will not affect our friendship because he knew I could not transmit to him. I told him that he was my true friend whom I would trust and to leave the rest to go, so we stayed back and talked a lot. So when I was in class eight, I started talking to girls and I had a girlfriend who also wanted us to go for testing together, but I told her that I did not want it but she insisted that if I loved her we should go for testing, so we went, when tested they started with her and she was negative while I was positive, so she said I had the disease and could infect her but I told her I cannot transmit to her if we used a condom while having sex, so she said okay, we will be using protection. Some friends told me to stop using the medication, but I told them that it was my status and I had accepted it and cannot stop using them.

I Okay. You have said that when you were five years old is when you asked your mother why you were taking the drugs

R *(Interjects)* Yes

I and she told you that you had malaria, and when you came to the clinic, the doctor told you that you are HIV positive

R *(Interjects)* Yes

I How did you feel at that time?

R I felt bad

I Okay, what was going on in your mind at that time?

R I had different thoughts, I felt like taking a knife and stabbing myself, I also felt like committing suicide through strangling myself, something else told me in my mind that I should not do that, that I will not die, I should just use my medication well and I chose that one. And as we can see I’m still alive.

I Which is good. Did you tell your mother that you discovered that you are HIV positive?

R Yes

I What did she say?

R Nothing

I Mmh, okay. How has it been?

R Good

I Okay, you are just taking your drugs as it is expected?

R Yes

I Okay, and you have told me that you went for testing with your friends and you turned out to be positive and there is one of them who did not want to share their items with you

R *(Interjects)* Yes

I How did you feel about that?

R Augh good

I Why good and someone doesn’t want to share their items with you?

R No problem, it is okay, if you don’t want to share with me I’ll show you the door, so I remained with my two friends who I share with and do almost everything together.

I Okay, that is good. So, has it been easy coming to the clinic and taking your drugs? Or how has it been for you?

R It has just been okay

I *(Interjects)* Yes

R It has been easy for me taking the drugs

I Okay, and what has helped in your adherence to clinic visits?

R Sometimes I finish my drugs and remain with maybe one dose, so I have to come back for a refill especially during exams so that I do get interrupted with my studies

I Okay, and what encourages you to come to Lumumba Hospital?

R The people in Lumumba really love me, I’m like one of them so I when my clinic day reaches I just tell my mum that my clinic day is today, and she gives me bus fare to come.

I So the workers in Lumumba have encouraged you to come to the clinic

R Yes

I They love you?

R Yes

I What else has motivated you to continue coming to Lumumba?

R I have friends here who know me, the doctors also know me

I Okay, any other thing?

R No

I Have you ever experienced stigma or is there anyone who has ever discriminated you because of your HIV status?

R No

I You have never experienced stigma?

R No

I There is no one who has prevented you from doing something because you are HIV positive?

R No

I Okay, so tell me anything you know about ARVs

R A?

I ARVs Antiretroviral drugs

R What I know is that it is good, I don’t like noise or differences with people

I What I’m asking is what do you know about these drugs? The ARVs.

R These drugs?

I Yes, what do you know about them?

R When you use the drugs it makes the virus in the body dormant

I Dormant?

R Yes

I Okay

R Such that when the virus starts to be active and you use the drugs, it makes them become dormant again and cannot disturb you

I You have known that the drugs make the virus inactive

R Yes

I What else do you know about these ARVs?

R Nothing

I You only know about that

R Yes

I Any other thing you have heard from people talking about in regard to these ARVs?

R There is no one else who I’ve heard talking about it

I Okay, and have you ever missed taking your ARVs?

R No

I Is there any point in life that you have missed taking your Antiretroviral drugs?

R No

I How about your clinic, have you ever missed attending to your clinic visits?

R No

I Okay, so do you have any challenges in keeping your clinic appointments?

R No

I You don’t have any challenge?

R No I don’t have

I Do you think other people could be having a challenge in keeping their appointments?

R No

I Like maybe your friends; do you think they might be having challenges in coming to the clinic?

R Unless you talk to them to understand

I but is there any of them who has told you that maybe they are experiencing certain things, so they are not able to come to the clinic

R No, none has told me

I and you have said you have not missed your appointments

R Yes

I So tell me about the last time…sorry, tell me your general feelings about the HIV care services you are currently receiving…How are the services you are currently receiving at the clinic?

R Good

I They are good

R Yes

I Aahh, what makes them good?

R In the past at the clinic there were some drugs where if you used, you could feel fatigue on the neck

I Fatigue on the neck

R Yes

I Continue

R You could feel dizzy but after joining this (not audible) I feel good nowadays

I Any other feeling about the services you get here?

R They are good

I They are good, what is good about them?

R We have doctors who love people, love children, if you come they welcome you very well

I Okay, so what do you think or how do you think HIV care services can me made more satisfying?

R Mmh

I How do you think your encounter with HIV services can be made more satisfying?

R What I know is that a times if you do not eat you will have a lot of virus in the body and that means you won’t live for long

I What I’m asking is as you come to the clinic, is there any other thing that can be done to help you continue coming or to encourage you to continue coming?

R Yes

I Like what?

R Since I like coming to the clinic, I believe there are some things that are important that I want to learn if I don’t know about them, so I when I get the information, I can pass It to someone else.

I So you want to be taught about other things

R Yes to be taught about other things so that I teach others

I So that you teach others

R Yes

I What kind of teachings would you want?

R How this stigma and other things like taking of drugs, and how it affects the body. I want to encourage others to continue using their drugs since the drugs are very important to the body. Even if you like a girl and she does not want you, just take your drugs since it is the drugs and not the girls which will help you in your life

I So those teachings are what you want so that you go back and teach the rest?

R Yes

I Okay, that is good. Any other thing?

R No

I Okay, do you think being in school or college for young people affects keeping their clinic appointments and taking ARVs?

R Yes

I How?

R You know, the teacher did not want me to come here, so when I usually come, the teacher starts talking bad about me and my status, but I always say, I know that I have the disease and I will stay with it and I will never die, so teachers really talk bad stuff about me but I always say let them talk, they will get tired and keep quiet, so nowadays this teacher is not there

I So you feel like the teachers can discourage someone from coming for their medication?

R They prevent sometimes but I always come to the clinic

I How did you take the situation where the teacher prevented you from coming to the clinic for your medication and also talking badly about you?

R Bad

I Mmh

R But I said to myself that I should let them talk till they stop on their own, so nowadays when they dismissed that teacher and brought in another one who knows my problems and cannot talk negatively about me. The teacher also knows how I am hot tempered, sometimes we sit together and have stories sometimes the teacher buys lunch and we eat together, that teacher does not have a problem

I Do you think that being in school can prevent someone from attending their clinic visits or taking their medication?

R Yes

I Okay

R We are three hundred of us who go to the clinic, so if it is a lady the teacher allows her to come

I Why do they agree for a girl to come and not a boy?

R I don’t know

I Mmh, okay so being in school affects in a way?

R Yes, so what I usually say is let the teachers choose the girls. I’ll decide on my own to go wherever I want, and no teacher will stop me.

I Okay, that is nice. So, I want us to discuss something on sexual and reproductive health. What do you understand about that?

R The way they dismissed that teacher now I feel okay, so I take the advice of this new teacher and go.

I That is good. Do you know the meaning of sexual and reproductive health?

R Yes

I What is it?

R I know, sometimes doing things and this… doing things by yourself

I Okay, so when we talk about sexual and reproductive health, we are talking about access to reproductive information, you have been taught about your reproductive system right?

R Yes

I Like the reproductive organs

R Yes

I and when we link it up to what we are discussing we want to look at things like safe sex, you’ve ever heard of safe sex?

R Yes

I Family planning use, STI and STD prevention, so when we talk about sexual and reproductive health we generally talk about that, diseases that affect our reproductive organs, diseases like the STIs and HIV, safe sex and when we talk about safe sex, we talk about things like condom use, is that okay?

R Yes

I And Even family planning is also part of the reproductive health because the family planning is to prevent people from getting pregnant. Is that clear?

R Yes

I They don’t prevent but they just, at that moment someone will not be able to become pregnant, is that okay?

R Yes

I So, how important is your access to sexual and reproductive health services?

R No

I It is not important?

R Yes

I Why?

R Because, you know at our home there are no people around so when they get you they can beat you up, if you are caught with girls the father will call the police to you so if you meet at the kondele fly-over it is regarded as a dangerous place

I So do you think it is important for you to get information or services about sexual and reproductive health?

R Yes

I Why do you think it is important to get that information?

R Because if you want other to understand, you tell them that if you are a man or a woman you should go (stammering) you should go…you should go for…for

I (Interjects) To be taught

R Yes

I Do you think it is important for you to get such information or service?

R Yes

I Mmh, so how important is it? Does it help you?

R It also helps me

I It helps you too?

R Yes

I So, currently *(participant’s phone rings),* currently, where do you access information on sexual and reproductive health? This information we have said about condoms, STI, where do you get it?

R Me

I Yes

R Where I hear it from?

I Yes

R I started hearing about it a long time ago

I Where?

R Just here in Lumumba

I at the clinic?

R Yes

I Where else have you heard the information on sexual and reproductive health ?

R I hear it from dances during roadshows

I During roadshows?

R Yes, you know there are so people who pass near our home with trucks which have condoms drawn on them, that one

I Okay, and where do you get the services? For example, if you want condom, where do you get them from?

R Just here

I at the clinic?

R Yeah

I What if you want family planning?

R I don’t want

I You don’t want?

R Yes

I and what if you want like maybe information, do you still get it at the clinic?

R Yeah, I get all the information here

I Even the condoms, you get them here

R Yes, we take them from here

I Okay, and where is the ideal place where you would like to get sexual and reproductive health services?

R For me, I think that is what I can say

I Where would you prefer getting those services

R Just here

I at the clinic

R Yeah

I Okay, and do you think the sexual and reproductive health services offered to the young people at the clinic like you, do they meet your needs and expectations?

R Yes

I Okay, it does meet your needs and expectations, so like for example when you said you come for the condom at the clinic, do they meet your needs?

R They know that we take them, so I am free to take them, everyone is free to take them

I So after taking them do they satisfy your needs the way you wanted it?

R Yes

I and do you think it also helps others?

R Of course

I Okay, and do you have any challenges when trying to access the sexual and reproductive health information and services?

R No

I You don’t have any challenge?

R Yes

I You have never experienced any challenge

R I have not

I So when you want any information for example like how to use a condom you will get it?

R I know how to use it

I but even if you come to the clinic you don’t experience any challenge when you want those services

R No

I Okay, and just uhm another question, do you think being in school for young people like you affects their access to sexual and reproductive health?

R Yes

I How does it affect?

R You know, when a science teacher comes and tells us something different like they say even if you are a girl, if you are told to do something you have to do it. So, in school we get different challenges, sometimes we are told that some doctors from Lumumba will come and such and such a person will be tested and give their bloods, so they say they sell our blood

I Okay, so you have just said that being in school will affect your access to sexual and reproductive health, say for example a young person like you or one in high school, do you think being in school can prevent you from coming to take a condom?

R It can’t prevent me

I What about the others, maybe like girls if they want family planning, can it prevent them?

R It cannot prevent them, you just come and talk, and they provide for you

I Okay, so how comfortable will you be, discussing your sexual and reproductive health concerns with a lay female health provider, someone of your mother’s age?

R That one too?

I Like how comfortable will you be, discussing your issues in regard to sexual and reproductive health concerns with a lay female health provider, someone of your mothers age? Can you discuss with them?

R I don’t talk to women

I You don’t talk to women but f you get someone working at the clinic who is almost the same age as your mother, are you able to discuss things that are troubling you with regards to sexual and reproductive health?

R Yes, I can tell her

I You can tell her?

R Yes

I Okay, what kind of things?

R I would teach her too and tell her my stuff

I Which of your stuff would you feel comfortable sharing?

R Like to tell her even if she is old she should take her medications

I but not necessarily on medication, things to do with sexual and reproductive health, can you discuss with her?

R Yes

I which kind of things?

R You know some people have problems, a times you hear they have a problem on their own, so they get scared

I Like for example, are you comfortable discussing with this person about your sexual life, will you be comfortable?

R No

I What would you prefer?

R You know, when you tell some people these things they would want to spread it out to other people, that is what I don’t like

I So which kind of a person would you prefer?

R Just the Doctor

I You’ll just prefer the doctor?

R Yes

I So you are comfortable discussing your issues with the doctor?

R Yes

I Okay, and if you have this issue of sexual and reproductive health for example you have a sign of an STI, like syphilis or gonorrhea, would you be comfortable in discussing these issues?

R With a doctor?

I Yes, it can be a doctor, so you have said you prefer a doctor, what about just a female healthcare provider?

R I don’t see a problem

I You can discuss with her?

R Yes

I Okay, where would you like such discussions to be carried out?

R For people to listen?

I Not for people to listen, just you and the lay healthcare provider

R Just the two of us?

I Yes

R It is supposed, and if they ask what the problem is I will tell him that the problem I have in my heart is this and this and they will advise me on what to do and what not to do.

I Okay, where would you want the talk or advice to take place?

R In there

I at the hospital, home or where?

R Just inside there

I at the clinic?

R Yes

I Okay, so initially you said you are comfortable discussing your issues with someone and how would you feel if you are assigned a lay healthcare provider to be your contact person and confidant and source of information on reproductive and sexual health

R Mmh

I How would you feel if you are assigned a lay healthcare provider to be your contact person and confidant and source of information on reproductive and sexual health when you come to the clinic, someone who can keep your secrets and advice you on different things, how will you fill about it?

R Good

I It will be good, and this person who will be assigned to you, what attributes would you like to see in them? What characteristics would you want to see in this person?

R The person?

I Yes

R The doctor told me not to fear saying anything

I (*Participants phone ringing*) so, you want someone who can encourage you

R Mmh

I Like for example, I’ll give you an example of myself, if I’m given someone at the clinic, I’d want someone who listens to me, what about you, what would you like from the person?

R A male doctor

I You would prefer a man?

R Yes

I Aha, what else? Remember this is a person you are going to share your secrets to

R Does not gossip

I What else?

R Does not spread rumors

I What else do you want from this person

R I want that if I tell him anything he should never tell anyone

I So you want someone who keeps your information private?

R Yes

I What else?

R Another one?

I Yes

R I like *(stammering)* I like reaction, I like solving issues at that point

I Okay, how would you feel receiving your ARVs together with your sexual and reproductive services at the same point

R What I would say is that I tell them

I No like when you come to the clinic, when you come for the ARVs at the same point you are advised on sexual and reproductive health and condoms, how would you feel?

R Good

I Okay, so what are the advantages of you receiving your ARVs and sexual and reproductive health together at the same point?

R What I can say is that even if you are given the drugs when it is almost expiring you need to come and take another one

I Okay, so do you see any help when you come to the clinic and you are given everything at once?

R Like what?

I Like you came for ARVs then you are also given condoms at that point

R Condoms can be taken without asking anyone

I Mmh, I was just giving an example like when you come for your ARVs and sexual and reproductive health at the same point, is there any advantage?

R There is importance of this

I Is there a disadvantage of getting all these on the same day?

R Yes

I Like which ones?

R Like when you are given, and you use them, you know some people love women so much

I Mmmmm Okay, thank you very much

R Welcome

I Thanks for participating in the interview. Do you have any questions?

R No

I Okay. Thank you.

Name of study: AYA

Type of Interview: IDI

PID: KLM 004

Date of interview: 4-Jul-19

Location: Lumumba Sub-county Hospital

Name of interviewer: Harriet Fridah

Name of transcriber: Harriet Fridah

I Okay so welcome to today’s session my name is Harriet and you are welcome to participate please feel free, whatever we are going to discuss is confidential. We will be discussing on sexual and reproductive health needs among adolescents and young adults okay? Let us begin by you telling me about yourself and be a little bit louder

R My name is XXXX.

I No you don’t have to tell me your name, you can tell me about your age, educational status, marital status if you are married, occupation if there is anything or activity you are engaged in.

R Okay, so I’m 20 years old, I am not married (laughing) I am single, still a student

I Okay

R I am in a college currently

I Are you involved in any activity, working sort of?

R No

I You are a student?

R Yeah, currently I am a student

I You are in college?

R Yeah, I am in college

I Okay, so do you have a baby?

R No

I Have you ever been pregnant?

R No

I Okay, are you sexually active?

R Yeah

I Do you have both parents?

R No, both dead

I Both died? sorry about that

R Okay

I They died when you were what age?

R My mum died when I was 12 and my dad passed last year.

I So whom do you live with?

R My sister

I okay, are you the last born?

R Yeah

I Okay, how many siblings are you?

R we are six

I Six?

R Five girls, one boy

I Aahh, okay. What are the challenges you are facing as a young person?

R Majorly, considering me and my condition, it’s like taking my drugs and my status has been a challenge. It happens a times you are not alone in the house, maybe some guests have visited or maybe like you see now I am in school, there are things I cant participate in school, they require me to participate at the exact time that I am supposed to be taking my drugs, because it will interfere. I will not also feel free in that at home there is this, you just feel odd because I am the only one infected in our family so a times there is some news on tv about HIV related cases, you just hear some funny statements talking about how it is bad and stuff, to them they don’t hold anything against me, they don’t even remember going through the same thing but deep down you get hurt because you know yourself and it is always on your mind, but you cant tell them or anything of that sort.

I Mmmm. Not really HIV related but any other challenge just generally as a young person that you are going through

R There are other stuff like family planning, okay on my side, when you are above 18 years you can access these services?

I Yeah

R If I am having sex?

I Yes

R but sometimes you can go to the family whatever setup, family planning centers then you are given the look like you are too young, you cannot indulge in such stuff, I’m not saying like young girls should go for such things, okay there is the good stuff and the bad stuff but I would prefer if someone is legally able to access them they should access them freely.

I So the challenges you are experiencing is that when you access family planning servicesyou feel like you are being judged?

R *(Interjects)*Yeah, you are being judged

I Okay

R You are being judged

I Okay, so what challenge or life challenge are you experiencing?

R Mmh, what can I say? Okay like myself I like keeping myself busy so a times you’d like to indulge in some activities maybe during the holiday but there are so many little things, maybe I am not the one with connections or staff but it is hard to get access to any activities that you can be involved in, whether it pays or not, so sometimes you just want to get busy or get the experience

I *(Interjects)* get the experience or get engaged in activities and get experience

R Yeah

I So its challenging because it’s hard to find

R *(Interjects)* hard to find

I What else, you have talked of you feel like you are being judged when you go to a family planning clinic, you need a place to engage yourself, but it is not forthcoming

R Yeah

I What else?

R Okay, if I think of anything along the way I will tell you

I Okay, so do you think these challenges have affected your education, socio economic advancements?

R Yeah, especially the one that connects to my status, it affects me most of the time. It took me long to find out about my status, I found out when I was 15 years, since childhood so it drained me, I lost a lot and it took me long to learn how I am so all this while I was just falling sick. When I was growing up I was really active but then it drained me especially the more I continued with classes, the more I continued failing, I could read and I knew I could pass but, I don’t know how to put it but it was so extreme in my body

I Okay, so when you say it affected your education, so it affected in that you were not able to study, or you were sick and not able to go to school or?

R Yeah

I That is one of the reasons?

R Yeah, like I remember I was in a boarding school and by the time I knew I felt so sick, actually it was my math teacher who called my parent, because I was good in math but whenever I did exams I was degrading. when my guardian came to pick me from school, that is when I found out that I was infected, so I couldn’t go back to that school, considering my status, it was a boarding school and I was not really free to take my medicine in a boarding school where other students were because it is challenging, it is not like you are in your own room or something

I Yeah, so maybe if I take you back a bit, at what point did you realize you are HIV positive or at what age?

R 15, I was in form two

I You were in form two?

R Yeah

I So when you were sick and brought for testing

R Yeah, I was taken to a certain hospital here that is when, after they did all tests and everything was okay. Actually I was diagnosed with TB at the moment so the doctor just proposed I should get tested. The funny thing my mum is the one who was infected, but my dad was not infected, so my mum knew her status, also she found out late because by the time she found out, it had already consumed her so we couldn’t be able to save her at that moment so she passed on. My family knew but since I was still a child and the youngest they did not inform me about the condition that my mum was in or even propose we go and get tested as a family. Back then I was in grade seven and maybe I could have even started medication early and prevented a lot. So...when I found out I couldn’t go back to school, actually it was early second term and I couldn’t go back to school at that moment, I was traumatized, I did not know what to do, I just felt it was the end of the world for me at that moment, I had to stay home the whole of that year and started the class afresh

I Okay

R *(Interjects)* Plus you know I was in a very good school, after that I didn’t go back to boarding, I went to a day, which is okay considering the standards, my performance could have been better if I were in the other school

I Okay, so it affected your academics, when you discovered you were HIV positive?

R Yes

I How about the challenge you talked of you want to be engaged in an activity but now it is not forthcoming, did it affect you in any way?

R I could say, somehow it has because when you are idle you get so many ideas coming in your mind, friends are there proposing so many things like parties and you indulge in things that you regret.

I When you talk of indulging in things that you regret, which are these things?

R Aahh, can I pass *(laughing)*

I *(Laughing)* Okay, that is fine, again another challenge you talked about is that you feel you are judged when you go for family planning, has that affected you?

R Yeah, it has

I Okay

R There is a time, actually it is early around twenty, actually I am turning 21 around October, so okay we are told, how can I put it, I wanted to access those family planning stuff so I went to this clinic… when I went there I told her my issue and she gave me the look of like seriously *(laughing)* okay you like physically I look really young, maybe 16 or something I told her I want services from this place and she said I was too young to be using those things. So the first time I only came to inquire, I was told, okay for me I’d rather take the precaution because I was seeing my friend right now is expecting and I have seen how it has affected her, so I was like I don’t want to find myself in that situation. I have just joined college and it will affect my studies, plus I’m not depending on my parents, I am depending on my guardian so it will be a double cost, you know your parent can understand and will still take you back and support you but when you are relying on someone else it’s too much. So, I was like let me go and get information so that I can avoid things like that, so this lady didn’t even want to explain to me whether the effects are bad, she just said you look young and she did not even know my age. People look young I might even be 25 and have my family, she did not even give attention she just said she is not attending to me, don’t engage in sexual activities, she just brushed me off .

I So you were not given the service

R No, she didn’t even tell me about the effects

I Even how it works?

R Nothing

I Okay, and did that affect you?

R Yeah, for starters, I had gone there, not even to start on the medication, I just wanted to inquire then go discuss maybe with a friend and see if I could start. I just wanted the information, is it okay for me? But she didn’t give me a chance; she just said I was too young.

I Wow, okay and have you tried to deal with these challenges?

R Yeah

I How?

R Okay, my…For family planning, when I come to the clinic I normally ask and the clinician often address me well and gives me the information that I need.

I Good. Any other way that you have tried to deal with it? Like challenges of being the only person who is HIV positive in your family, how have you tried dealing with such?

R You know I’m normally not social, I like keeping things to myself even when I have issues. Actually, funny thing , issues concerning my status, I have not been able to discuss with anyone.

I Not even your sisters?

R No

I What prevents you from talking to them

R Okay if there is one person that I kind of open up to is my doctor at the clinic. She is so open and friendly with me plus I know she will always keep my information confidential.

I Okay, so do you think you have succeeded in dealing with these challenges?

R Like?

I You talked of you wanted to engage yourself somewhere but not forthcoming, have you succeeded in dealing with that?

R that one I have not succeeded

I Okay

R Yeah

I and is there support for you to deal with the challenges?

R I have heard of support groups before but, I attended one late last year, but it was not related to that

I Not necessarily support groups, like is there support which is available like friend, sister, brother, aunt

R Oooh

I Okay, just even apart from the challenges you have, do you have people who you can fall back on like when you have issues? Not necessarily what we have talked of

R I guess all my sisters

I Your sister, apart from your sisters, any other person?

R No

I Okay, good, I think we are

R *(Interjects)* actually not all my sisters, just one

I One?

R *(Laughing)* yeah just one

I Why one? Why not the rest?

R The rest are kind of judgmental, they are not easy to talk to

I Okay. So, you have one person who you can at least open up to

R and she is the one I’m living with

I Okay, that is great. We are moving on well. I want us to talk about HIV care services. When we began you told me that you got to know your status when you were 15 years old, I just want to find out, at that time that you were brought in to the clinic and diagnosed with TB, were you started on antiretrovirals?

R It took me like a month to accept taking the medication, but the doctor proposed that I should be counseled. You know that situation; your mind is always all over, like, “how was I going to live?”

I Mmmmmm. Who brought you to the clinic at that time? your dad, sisters?

R My sister and her husband

I What about your dad

R my dad by then was staying at home

I So you were staying with your sister?

R Yeah

I Okay, so your sister is the one who brought you?

R Yes

I and now you were to be enrolled into HIV care

R (Interjects) Yes, I was

I so how did you feel at that time? When you had just discovered that you are HIV positive?

R Terrible, not even bad because I never pictured myself. Actually, the worst thing you know it is a different thing when you know you did a mistake like I acquired this from my carelessness not like transmission from parent, it was tough

I Tell me more. How did you go through that? Was there support?

R No, mostly I kept my problems to myself. I was not social so even disclosing was after some time

I I understand

R You know if your mum could have been there…. I was so free with my mum, maybe I would have opened up to her. With my sisters there is this gap like we are normally not that free and then I don’t open up to my friends because they can judge me. I could just stay in my room and cry or even write down something, actually once I’ve written down something and if there are questions that I have I just google and probably the next time I have a clinic visit I ask my doctor.

I Okay, and what really made it easy for you to enroll in care at that time?

R In terms of?

I Like when you were being enrolled, you just accepted

R It look me like a month to accept

I It took a month

R Even on that day I enrolled yes but I did not come to terms with the situation. I just like I had to do it but still I was not okay.

I Okay, and what has enabled you continue engaging in care here?

R Mostly is the reception you get from the clinicians here, like they are so friendly and make you know that they also understand your situation completely like any other questions that you may have they are always willing to help.

I Any other thing?

R You know when you get a very welcoming environment you feel free to ask anything

I So, the friendliness has enabled you?

R Yes. They show you like, they don’t put any boundaries like you are different from me or something of the sort.

I How about the distance from your place? Where do you stay?

R Nyalenda

I Okay, it is not far, you can still make it?

R Yeah, it is not far.

I Great. Tell me anything you know about antiretroviral drugs

R Anything?

I Yeah anything that you know about them

R All I know is that they are the ones that have made me reach where I am today because if I hadn’t started on that, I would have been forgotten already

I Okay

R and I must say, they help a great deal, like once someone has been diagnosed with HIV, the best option is to just start taking the medication immediately

I any other thing about antiretrovirals?

R Anything?

I Yes

R okay generally?

I Yeah, generally

R Mmh, I do know that, concerning how they protect the body or?

I Yes

R Okay, I know that they fight the viruses in the body, like for me currently my viruses are suppressed

I That is good

R Yeah, so plus different people take different types of drugs, like you are given according to the severity of your condition. I am grateful that I did not have to change drugs. There is a friend of mine who had to change from one drug to the other but with my case, the ones I started on are the ones I’m still using, yeah that is it, anything else?

I *(laughing)* okay so tell me about the time you missed taking your ARVs, what happened?

R I was going through so much I was having pressure and having issues with my sister. I was just going through a lot at that moment plus my studies, I had not recovered my grades, like I have said, I normally don’t talk and open up so easily so I had a lot of issues and couldn’t discuss with anyone. It even affected me, I could not concentrate well in class

I So you have said you also had issues with your sister, what really happened?

R Can I pass that *(laughing)*

I It is okay, you don’t really have to say it, so you missed your drugs at that time

R Yea. I felt like no one, like I didn’t even matter to anyone at that moment, because we make mistakes, I made a mistake plus, but the worse thing is my sister made a mistake but she could not accept the mistake so she reported to my dad. Let me just say what happened, so whenever I am sad I write it down but once I have written I don’t go back to check on it, it’s like where I express my anger. So, whenever I’m angry or messed up in the house or maybe there are days when someone can come in the house angry and releases all the anger on you, so I just write it down and feel okay. So there is this day I went out with my friend and forgot to keep my diary, so my sister walked into the room and found it on the bed and by curiosity I guess she opened it and read everything so when I came back I didn’t know what was happening *(laughing)* and honestly it’s not like I was holding anything against her, but to her, she saw that I was recording everything that she was doing to me so she was like I can’t stay with someone who is recording everything that I am doing to her, maybe you are planning on something in the future. So, she said, “Now you have to go home and stay with your dad.” I can’t stay with you anymore. Actually it happened I had resumed form two since it was the year before that I was diagnosed so it took me like a year again and it was second term. I was now in form three, actually it was on a Sunday and the next day on a Monday I was to resume classes. “Now I cant stay with you go home stay with your dad, since he is the only one you are okay with. She and the husband said a lot, actually they literally threw me out of the house

I Sorry

R It is funny, they were packing my stuff, so they gave me the exact fare to get me home, so I pictured, first she is my oldest sister so whatever she tells me, that’s it.

I What was your dad’s reaction?

R Obviously he took her side, I tried to explain to him. I was just writing, at that moment I could write a lot like poems and things so my dad took her side and said since I don’t want to study, just come home you start a family and I told him I can’t go home. I went to my aunts place, the sister to my mum and explained everything and she understood, so I stayed there for like two weeks, they didn’t know where I was and no one even bothered to ask me, so after two weeks is when my aunt called my dad and asked him how is your last born, first of all he lied, he said she is okay, “are you sure?” My aunt asked him. “Okay, she had issues with her sister and we have not heard from her for like two weeks maybe she got married,” actually they concluded that I got married. She even said some of my friends from school came home asking for me, so he said we assume she got married with a kid, so when my aunt told them the whole story they were surprised because they took me as a bad girl, actually I’m not supposed to say I’m perfect but at that moment I wasn’t.

I That is the time you missed taking your medication?

R Yeah, I felt like I was not needed

I Okay. Do you have challenges with keeping your clinic appointments

R Currently it is kind of tough because I’m studying far away from my clinic and it happens that the days that I’m supposed to report to clinic often it finds it’s when I’m having exams

I Okay, how do you handle that, like when you have exams?

R My sister, actually my same sister has been very supportive, she is the one who gets me the drugs and send me

I Okay, to school?

R Yeah

I Have you ever missed your appointments?

R Missing like?

I Clinic

R Okay, no I have attended all

I and generally what are your feelings on the services that you receive here

R They are good

I What makes them good?

R I have just said the way they deal with their clients, they are generally good, this is one place where you know people know who you are, the only place where you can be open and talk about your fears, your worries and get the responses

I Okay and how do you think your encounter with HIV services can me made more satisfying?

R Mmh, fine

I So you feel the services are okay?

R Not really

I Mmh

R There was an App I was referred to last time, like it could keep track of the time you are supposed to take your medicine and it helped me a lot. You know sometimes you get so busy that you forget that it is your time to take medicine but it could remind me of my visit and time to take my drugs

I So you’d want that App?

R Yeah

I to help you remind you?

R Yes

I What else?

R Mmh...

I Only that?

R No, plus, okay maybe make it, it should be more… like maybe have a specific date in place because you know we are not the only ones coming to the hospital plus a times, and another thing like the doctors who attend to us should be people who really understand our case

I So people who really understand your case

R Yeah

I You want an App like a reminder thing

R Yes plus another thing when coming in as I have said we are not the only ones coming in, it would be more private when getting the medics because a lot of people around, maybe that is my concern

I So you want like a private place to pick your drugs?

R Yeah

I You don’t want to go to a place where everyone picks theirs?

R Yeah

I Okay, so do you think being is school or college for young people like you affect their keeping of clinic appointments and taking ARVs?

R No, it shouldn’t affect you, as long as you know yourself and know the goals that you have set and you know these medicines help you. Once you stop taking them, it will literary affect you, plus I’m told you don’t have to come to specific hospitals if your drugs are over, you can go to a nearby clinic wherever you are to get the ones will last you until you go back to your clinic

I So being in school doesn’t?

R Yeah

I Okay, even other people, not necessarily you, even others are not affected?

R Okay, you can kind of be affected because it is a different thing like going to a near-by clinic like me I prefer coming here because I will be like going to a new clinic and you don’t know that procedure, the people there start judging you. It is a new experience like for the first time, especially if it is a long distance thing it can be a challenge

I What about taking drugs

R taking drugs?

I Mmh

R Okay I stay alone outside school, so that is the advantage that I have, like I am free to take my drugs but now considering the ones staying in hostels, it is tough, because hostel you don’t stay alone its like cubes of four people or six so maybe keeping track of time you will have to wait until they are asleep or keep them busy.

I Okay, we are moving on well. I want us to discuss on sexual and reproductive health services, what do you understand by the term sexual and reproductive health?

R sexual and reproductive health?

I Mmh

R It is generally, it’s about like being actively sexually, being sexually active and maybe accessing the medication, information, things like family planning

I Okay sexual and reproductive health as you have just said is accessing information on things to do with STIs, safe sex, condom use, STDs and STI prevention, things to deal with family planning or contraceptives.

How important is your access to sexual and reproductive health services?

R The importance?

I Mmh, how important is your access?

R It is very important because if I don’t get access to things like condom, you know the youth, we indulge in such things so to easily prevent infections you can use condoms and early pregnancies and it should be readily available

I and where do you currently access information on sexual and reproductive health services?

R My clinic

I Your clinic, so you only get information

R *(interjects)* no, not generally from the clinic, social media nowadays, internet, yeah

I How about the services?

R *(Interjects)* Even friends

I Even friends, okay. How about the service?

R So far like anytime, it has improved

I Where do you access the sexual and reproductive health services?

R Like, my case if I am interested I deal with my clinician

I Your clinic, okay. Where do you think is the ideal place or location you would like to access sexual and reproductive health services?

R I guess hospitals, clinics, a place where we have someone who is informed, not like we see, the family planning does not go well with everyone, like I heard you had to undergo some tests to know what suits you so that you are not given the wrong method, so I think a place where there is information, someone who is well aware about the products and stuff and can advice you

I To what extent are the sexual and reproductive health services offered to young people like you meet their needs and expectations?

R Okay, as per now I cannot say they are that…

I Do the services or the information with regards to sexual and reproductive health services meet your expectations?

R Yeah

I They do?

R They do

I So if you want condoms you get?

R Yeah

I If you want information on safe sex you also get?

R as long as you approach the right person you can get

I and as a person, what challenges do you face when trying to access sexual and reproductive health services?

R Challenges

I Yes

R Mostly being judged, that is the main thing

I You feel like you are being judged when you come to the clinic for sexual and reproductive health services?

R So you got an STD, you are too young why should you start on family planning things like that

I Okay. How does being in school or college for young people like you affect your access to sexual and reproductive health services?

R Okay, on my case, as long as you believe in yourself, you should not be afraid to get information, ask for anything because these hospitals are all over, so it depends with the person

I so being in school does not affect the access

R The access, no

I others feel that when they have exams it interferes with their access. Your thoughts on that?

R Now that depend on how you program yourself

I Okay. How comfortable will you be discussing your sexual and reproductive health concerns with a lay female health worker who is of your mother’s age or for you case your sister’s age?

R It’s tough

I *(Laughing)* It’s tough?

R Actually it took me time to even open up to my clinician. You monitor a person first, but it depends with their reaction, like you will find they are so open, I don’t know how they do it

I You wouldn’t feel comfortable

R Not all, most of them create tension but there are a few who you feel free approaching someone

I So what would you propose if you want to discuss your sexual and reproductive health with someone, which person would you propose?

R It could be a young person, elderly, it depends. There are elderly people who are approachable same as young people so it depends who do you, you have to examine the person, I am sure there is a way you can know who you can tell things but I’m sure you can’t lack one person

I and what issues will you be comfortable discussing with this person in regard to sexual and reproductive health services?

R Access to family planning, STI drugs

I What else?

R Majorly I think that is it

I and where would you like those discussions to take place?

R Information helps a lot, so you don’t want to get in to something that will affect you later, so it is good to ask first

I Good. Where would you like those discussions to take place?

R Hospital

I Why hospital?

R Its is kind of safe and private plus they know how to keep your information intact

I we are soon winding up. I just want your views on potential interventions when it comes to access to sexual and reproductive health services among adolescent and young adults. How would you feel if we assigned you a lay female health care worker who will be your confidant and source of information on sexual and reproductive health services?

R I will feel great

I You will feel great?

R Yeah like it would be a load offloaded from my shoulders because you know even if I have issues, there is this one person who I can go direct to

I (interjects) and get help

R Yeah and get help.

I so what attributes would you want in this person?

R Confidentiality, number one. He/she should not be judgmental, so confidential, not judgmental plus someone you who is free and friendly. One who creates that environment where you can talk to someone, like I’m talking to my agemate like you see how I talk to my best friends

I Okay, any other attributes?

R She should be informed; of course *(laughing)* you don’t want to go to someone who is not knowledgeable.

I *(laughing)* True

R maybe you have critical issues you want to deal with, because you want to maintain whatever, she just gives you information which she is not sure of.

I Mmmmm. What would you feel about receiving your ARVs and sexual and reproductive health services at the same place?

R I will be okay to help with the trouble of getting them from different place, plus there is no one who knows you well than your clinician so I guess at the point where they are giving you the drugs, they will know what is also best for you.

I Do you see any disadvantages with that?

R No

I Okay, and what would you propose as a person to improve on sexual and reproductive health services among adolescents and young adults?

R To improve on that?

I Yeah

R Maybe creating awareness

I to whom?

R To the youths in general because many youths are not sure, like personally when I went for the FP I wanted information because they did not know how it would affect me so they should create awareness so that the youths can know both the positive and negative effects, so that they can seek help

I Awareness, any other thing?

R Actually just that plus on how you can access them, create awareness on importance and maybe the accessibility

I Thankyou very much for your time, it was a nice discussion

R Welcome, indeed it was

I Any other question?

R No, I think that is it

I Thank you.

**AYA IDI: KLM 012**

**Site: KLM**

**Date: 11 Jul 2019**

**Interviewer: Harriet Fridah Adhiambo**

**Transcriber: Harriet Fridah Adhiambo**

**Version: English**

**Interviewee Details**

Age: 19 years

Sex: Female

Level of education: In University (1^st^ Year)

Occupation: Unemployed

Orphan hood status: Total orphan

Sexually Active: No

Marital Status: Single

Ever pregnant: No

I Welcome to this interview. My name is Harriet Fridah. We are going to discuss on sexual and reproductive health care access among adolescents and young adults as earlier informed during the consenting process. Kindly feel free to share and whatever we are going to discuss will be confidential. Kindly tell me about yourself

R I am (XXXX). I am 19 years old. I studied at Bishop…… Secondary school. Currently I am a student at University of Nairobi undertaking hospitality. I am single.

I Great. What are the challenges you face as a young person?

R Unemployment

I Mmmm, unemployment. Tell me more…

R Lack of money

I What else?

R Only those.

I Okay. When you talk of unemployment and you are currently a student, are you looking for employment?

R Yes, so that I can get money to help my family.

I Okay. How many children are you?

R We are two,

I you have a brother or sister?

R Brother

I In campus, secondary?

R (Interjects) In primary school.

I Whom does he stay with?

R My guardian

I Here in town?

R Yes.

I Any other challenge you face as a young person apart for unemployment, lack of money to take care of your family?

R I don’t have nay other challenges.

I Okay. Do you think these challenges have affected your education and socio economic advancement?

R No

I They haven’t affected you in any way?

R No, because my guardian provides.

I Ooo, your guardian provides. Okay.

R Yes

I So for example if you need money your guardian provides.

R Yes she does. So they don’t affect me in any way.

I Okay. Have you tried to deal with these challenges? Like unemployment? Lack of money?

R I offered catering services to get some money.

I Okay. Is it something you did once or you always do?

R I assisted my aunt. She is the one who got the tender to offer catering services.

I She got tender to offer the catering services and paid you for assisting her?

R Yes, she paid me.

I Any other way you deal with the challenges?

R There is an organization called (KEYOP). They take young girls, educate them and give them some money (6,000/=) at the end of the month for up keep. I was among those who were taken by the organization.

I That’s great. Tell me more about the organization

R They take young girls and educate them.

I So, they are educating you? Where?

R Yes, at University of Nairobi.

I Okay. Which course are you undertaking?

R Hospitality

I Okay. So you are paid on a monthly basis?

R Yes, but it takes long before they pay us. You know it is a government project

I How do they identify those that need to be supported? Does one apply?

R Yes, you apply first. If you are successful then you are taken.

I Okay. You have told me that you have challenges with getting employment and also you don’t have enough money but you at least try to get some jobs like catering and there is an organization that supports you. Do you think you have succeeded in dealing with these challenges?

R Yes,

I To what extent have you succeeded in dealing with these challenges?

R Not much

I Mmmm, when you talk of not much, could you explain further

R As in, the money I get is not enough

I Okay. What support is available to help you deal with these challenges?

R Support?

I Yes

R Support from my guardian

I Mmmmm

R They give me money, like lunch money…

I Okay. Any other support available apart from your aunt?

R My other aunts

I Okay. How do they support you?

R Advice and sometimes they give my guardian money to support us.

I Okay. Apart from your relatives, do you have any other support?

R The dream girls

I Mmmm, tell me more about the dream girls?

R It’s also an organization that educates young girls. They send you 2,000/= per month for upkeep, provide you sanitary towels and sometimes clothes.

I That’s great! I want us to discuss on HIV care services.

Could you please describe to me your experience the day you were enrolled to HIV care.

R I was very young then and I did not know that I was HIV positive.

I When was that?

R 2008

I How old were you at that time?

R I was 7 years old.

I Who accompanied you to the clinic the day you were being enrolled to HIV care?

R My aunt used to bring to the clinic

I Did she ever tell you why you were coming to the clinic regularly?

R No

I At what point did you discover that you were HIV positive?

R In 2014.

I How old were you then?

R 12 years

I Okay. Tell me how you got to learn of it

R Through Sunburst. They told us that we were all HIV positive during one of the support group meetings.

I Mmmmmm,

R We were being taught on the importance of taking ARVs and that is the point where they mentioned that we were all HIV positive.

I That is when you knew you were HIV positive?

R Yes

I Did you inform your guardian that you found out that you are HIV positive from the clinic?

R No, I didn’t tell them.

I Why?

R I ignored that because they are the ones who used to bring me to the clinic when I was young. So, definitely they knew that I was HIV positive.

I has your guardian ever opened up and discussed with you about your status?

R No.

I The day you were being taught by the sunburst team you discovered that you are HIV positive. How did you feel about that?

R I just took it normal

I Mmmm, you took it normal? What do you mean by taking it normal?

R I have been taking those drugs since I was young and you know people with HIV live long if they take drugs as expected. So I just took it as part and parcel of my life.

I Mmmmm, what was going on in your mind at that time?

R Honestly, I felt bad that my aunt kept the truth away from me

I You felt bad…… Did you ever tell her?

R No

I You kept it to yourself?

R Yes. I was very angry but could not tell my aunt. So, I asked my mother.

I Mmmm

R My mother died last year

I Sorry about that. You told me you have been living with your aunt/guardian since you were 7 years old. Where was your mother before she died?

R She was working in Nairobi. My brother and I were living with my aunt here in Kisumu.

I Okay. You told me you asked your mother why you were HIV positive. What did she tell you?

R She explained to me everything

I Mmmmm, tell me more?

R She explained that I was born HIV positive and that she is also HIV positive. At least I felt relieved when she explained that to me…..

I How about your younger brother?

R No, he is not HIV positive.

I Okay. What about your father?

R I am not aware

I Okay. What has enabled you to continue engaging in care at this facility?

R At least I get the drugs that kill the viruses

I Mmmmm apart form the drugs, what enables you to continue engaging in care here. Is it the staff? Is it that it is close to where you stay? Quality of the services offered?

R The staff

I Mmmmm

R Lets say when you have an emergency; they listen to you and assist as needed

I What else?

R The staff do not share my information

I Ahaa, they don’t disclose your status

R Yes.

I Any other thing?

R No, only those.

I Okay. Have you ever been discriminated against because of your HIV status?

R Where?

I Anywhere, not necessarily at the clinic but anywhere

R No.

I You have never experienced stigma…..

R No.

I Do you think there are people who have been discriminated against because of their HIV status?

R Mmmm, I heard of a story

I Tell me about that story

R There was a girl who was sick and when she went to the hospital for treatment, she was tested and found to be HIV positive. She didn’t believe that indeed she was HIV positive and attempted to commit suicide. People did not want to share anything with her because they thought she would infect them. She was traumatized by these events. Her sister came in and talked to her and explained that their mother was HIV positive that’s why she also turned out to be positive. She finally accepted and now she is an advocate for HIV in one of the organizations.

I Mmmm, that unfortunate. What do you think about that?

R It is bad

I Mmmm, okay. Tell me anything you know about ARVs

R ARVs?

I Yes

R They help kill the virus

I Mmmm. What else?

R They kill the viruses in our body.

I Yes, any other thing about ARVs?

R I only know that.

I How about taking the ARVs? Tell me anything you know

R If you take them, you will live long since they lower the virus levels in the body

I Mmmm, what else?

R If you take the drugs, nobody will know that you are HIV positive.

I Mmmm… There are people who say that they have side effects. Have you heard or experienced any side effects?

R No.

I Okay. Tell me about the time you missed taking your ARVS, what happened?

R I missed but nothing happened. When I came to the hospital, they told me that was bad and I should not at any time miss taking drugs.

I You missed for how long?

R One day

I Is it a one-time event or you always miss?

R It was just once.

I Okay. What made you miss taking your drugs?

R Time

I Mmmm

R I normally take my drugs at noon and the rest in the morning. So 10 minutes elapsed from the time I am supposed to take and I decided not to take till the next day.

I Mmmm, so you reported this at the clinic?

R Yes, and they told me not to repeat that.

I Okay. Have you ever missed again form that time?

R No

I You take your drugs at what 12.00pm?

R No, I take them at 8.00am and 8.00pm

I Okay. Could you please share with me the challenges you face with keeping your clinic appointments?

R Sometimes you have exams and you my return date is also due

I Mmmm, what do you do in that situation?

R You either come to the clinic or postpone till the next day

I Ahaa, so when you have exams it makes it challenging to attend to your clinic appointment. Any other challenge? Like transport to the clinic?

R No.

I Apart from having exams, what else makes it challenging to keep your clinic appointment?

R You could only have lunch break to come to the clinic, unfortunately when you come the queues are so long

I mmmm, long queues. Tell me about the last time you missed your clinic appointment

R I have never missed my clinic appointment

I Mmmm, even when you have exams?

R No

I Okay. What are your general feelings about the HIV care services you are currently receiving?

R The services are good.

I Mmmm, what makes them good?

R The drugs we are being given really help.

I Mmmmm

R We are educated on the dangers of not taking ARVS and also they used to give us trans port when come to the clinic

I You are being given transport money to the clinic?

R Nowadays they don’t

I Mmmmm, okay. Have you asked why you are no longer being given transport?

R No

I You have said that the services being offered are good, the drugs given are helpful, you are educated on the dangers of not taking ARVs and you used to be given transport to come to the clinic. Anything you need to add regarding your feelings towards the HIV services offered?

R *(Silence)* None

I How do you think your encounter with HIV care services can be made more satisfying?

R The queues

I What about the queues?

R They are usually long especially during adolescent days (Thursdays), you will also find adults who have come. It should be for adolescents only

I Okay, so you want the adolescent days to remain only for the adolescents and adults should not come

R Yes.

I Okay. How else would your encounter with HIV services be made more satisfying?

R The other services are okay.

I How does being in school/college for young people like you affect their keeping clinic appointments and taking ARVs?

R Maybe they are feeling bad about their HIV status and feel isolated form the rest. So it becomes difficult to keep their appointments

I Mmmm

R At times one could have exams and also the clinic appointment is due; it will therefore interfere with your clinic appointment attendance.

I Mmmmm, what else

R Transport. One may lack transport to come to the clinic

I Mmmm okay. How about taking ARVs while in school/college?

R Being in school does affect taking ARVs because they fear that other students will see them taking the drugs and start telling everyone.

I Mmmm, fear…..

What else?

R I think fear is the main thing.

I Okay. Do you fear?

R At times. I rarely take my drugs in school though….

I Mmmm. You take them at home?

R Yes, at home.

I Great. We now move on to sexual and reproductive health.

What do you understand by the term sexual and reproductive health?

R How to take care of our bodies, how to prevent STIs and STDs.

I You are right. Just a clarification, what do you mean by taking care of our bodies?

R Using condoms while having sex to prevent you from getting or spreading infections.

I Okay. What else?

R Maybe you are pregnant and you wan to abort the baby, you can seek advice since its dangerous.

I Mmmmm, great. You have an idea of what sexual and reproductive health encompasses. As you have mentioned, it involves access to sexual and reproductive health information, safe sex, prevention of STD/STIs, condom use, family planning/contraceptives.

How important is your access to sexual and reproductive health services and information?

R Its important because when I want to have sex, I use the condoms

I Mmmm.

R Also helps one to know if they have the STDs/STIs

I Good. What else?

R Family planning

I Tell me more about family planning

R When you don’t want to become pregnant then you can use family planning

I Okay. Where do you currently access information on sexual and reproductive health?

R From the health facility

I Mmmm, where else?

R My aunt

I Mmmmm

R School

I You are taught on sexual and reproductive health in school?

R Yes and also the dream girls

I Okay. How about the services? Where do you currently access services on sexual and reproductive health?

R At Lumumba health center and Russia hospital (Jaramogi Oginga Teaching and Referal Hospital)

I Where is the ideal place/location you would like to access sexual reproductive health services?

R Lumumba Hospital

I Mmmm, why Lumumba?

R They offers good services

I Okay. That means you would like to access those services from a health facility

R Yes.

I How about at home?

R You know I live with my guardians so I may not be comfortable receiving the sexual and reproductive health services from home.

I Okay. To what extent are the sexual and reproductive health services offered to young people like you meet their needs and expectations.

R When you have protected sex, you protect yourself from acquiring STDs/STIs

I Mmm, so does that meet their expectations/needs?

R Yes.

I Mmmm, what else?

R They also get chance to educate their peers on benefits and risks abased on the information they have received.

I Okay. What challenges do you face in trying to access sexual reproductive health information and services?

R I don’t experience any challenges.

I Okay. When you want information or services you get without any difficulties?

R Yes.

I How does being in school/college affect young people’s access to sexual reproductive health information and services?

R They misuse it

I How?

R Some people are just ignorant. They know they are HIV positive and still engage in unprotected sexual intercourses

I Okay. Just being in school/college does it affect a young person’s access to sexual reproductive health services?

R No, in school you can also be given those things like condoms. Most boarding schools have school nurses, they can go the nurse and she will provide them with the information or service.

I Okay, so you think being n school/college does not affect a young persons access to sexual and reproductive health services?

R No.

I How comfortable will you be discussing your sexual and reproductive health concerns with a lay female healthcare provider, someone of your mother’s/guardian’s age?

R (Silence)I will not feel comfortable

I Mmmm, why?

R If she asks me other questions then I will not respond

I Mmmm, like which ones?

R Like, “Have you had sexual intercourse”

I Mmmmm, okay. So, what would be your preference?

R A female

I Okay. A female who is older or younger?

R Younger

I So, you will be comfortable with a younger person

R Yes

I Okay. What issues will you be comfortable discussing with this person in relation to sexual and reproductive health?

R How to access family planning, condoms and how to use PreP and PEP

I Ahaa. Okay. Where would you want those discussions to take place?

R At the facility

I Okay. Why at the facility?

R At least at the facility they know you and understand you better.

I How would you feel if we assigned you a lay female healthcare provider to be your confidant and source of information on sexual and reproductive health?

R I will be okay

I Okay. I remember earlier on you mentioned that you would prefer a younger person

R Yes.

I what characteristics/attributes would you want in this person. This is someone who will be your confidant and source of information

R Understanding

I Understanding. Mmmmm

R Non judgmental

I Mmmmm

R Respectful

I Understanding, non judgemental, respectful. What else?

R One who keeps your secrets

I Confidential?

R Yes

I How would you feel about receiving your ARVs together with sexual and reproductive health services at the same point?

R (Silence)

I An example is when you have come for your ARVs and also wanted condoms or family planning then instead of going to another room for the family planning, you are given all these at the same place where you receive ARVs.

R I would not be comfortable

I Mmmmm, why?

R I just don t like it that way

I You would prefer to take your ARVs then go to another room form the other services?

R Yes,

I Okay. Do you think there are advantages when you receive these services together?

R (Silence)

I You know there are people who would prefer to get services at the same room so that they don’t have to come back another time.

R Yes, but

I (Interrupts) you would prefer to receive them separately?

R Yes.

I Okay. So you feel there are disadvantages of receiving these services together at the same point?

R Yes. Maybe the provider will tell your friends

I Mmmm. Okay. Any other disadvantage?

R None, only that one…

I What do you think can be done to improve access to sexual reproductive health services and information among adolescents and young adults?

R Educating them, giving them drinks when they come to then clinic and offering transport.

I Great. What else can be done to improve their access to sexual and reproductive health services?

R Encouraging the adolescents and young adults to access the services and also having a separate room for those services

I Good idea. Any other thing you think would be good for adolescents and young adults?

R Only those.

I Okay. Thank you very much for your time. We have come to the end of our discussion. Do you have any questions?

R No

I Great. Thanks.

**AYA IDI: KLM 012**

**Site: KKM**

**Date: 18 Jul 2019**

**Interviewer: Harriet Fridah Adhiambo**

**Transcriber: Harriet Fridah Adhiambo**

**Version: English**

**Interviewee Details**

Age: 19 years

Sex: Male

Level of education: Completed secondary

Occupation: Unemployed

Orphan hood status: Partial Orphan

Sexually Active: Yes

Marital Status: Single

Impregnated woman: No

I Welcome to this interview session. My name is Harriet Fridah Adhiambo. I would like us to begin by you telling us about yourself. (Your age, marital status, educational level, occupation…..)

R My name is T….. I finished my high school at Chianda boys last year. I am 19 years old and currently I am traveling a lot because I’m sorting out my education situation. I am yet to receive my Identity card, which I applied recently. I don’t like serious people very much (Laughs) but I love those who are lively.

I Okay. Currently what are you doing?

R I am just helping at home with house chores.

I Okay. Are you married?

R Not yet.

I Ever impregnated someone?

R No.

I Do you have both parents?

R No. My mother passed on while I was 3 years old. I live with my guardian here in Kisumu.

I Okay. How about your dad?

R My dad is there but he doesn’t communicate. I live with my guardian.

I Okay. I want us to discuss about the challenges you face as a young person

R The challenges I face with my drugs?

I We can talk on the general challenges, not challenges related to HIV care and treatment.

R Sometimes some people stigmatize me because of my physical appearance. While in primary school they used to say that I looked like an old person and that was and is still a big problem to me. My life is also not as good because there are a lot of family issues concerning my education that really fills my mind. So, I am not a happy guy always but I try. You know when you have a lot of thoughts you can die, so I try to avoid them a lot and stay positive. The main problem is just family issues here and there concerning my education, where I should learn, what I should do and I find it so hard even whom to believe. One person says one thing and when you ask someone else they say a different thing. So it’s hard to believe who could be saying the truth, who is right or wrong or even who is leading you to the right path. It’s a bit challenging.

I You have talked of stigma and family issues as challenges. How about high school, did you experience stigma?

R I did but it was not that much.

I Okay. How was it?

R People just wondering about my physical appearance, like I look old but it didn’t bother me at all. So it wasn’t much.

I Okay. You have also talked of family issues concerning your education. Could you please tell me more?

R My foster mother/guardian wants me to take my education here in Kisumu but my real grandmother…

I (Interjects)The mother to your father or mother?

R The mother to my mum. She wants me to take my education in Mombasa and also my aunt who has always been there for me and is my favorite also wants me to take my education in Mombasa.

It became a very big issue, as there was misunderstanding between the two groups but my foster mum gave in and told me that if I decided to go to Mombasa I will also have a different life from what I have had while here in Kisumu. I will meet new people and begin a new life there. You know here in Kisumu there is a lot of hype and a lot of things going on at least in Mombasa I will be able to focus. That is where I was born and adapting is very easy. The environment there is conducive and I can comfortably read, associate with people and I think that is good for my education.

I Have these challenges affected you educationally, socioeconomically or even in other ways?

R Yes, they have affected me. Before I finished my high school, I had this thought of what I will do when I finish high school. By then I was in form 3 and this really disturbed me. What next after I finish high school? My family kept on saying that I finish first then they will see what I will do. I decided to get buried in my studies so that I could excel.

I finished high school and could help around with house chores and even at times when my grandmother wants to see me I travel to her place. Even as we speak, I am from the village where my mother’s sister’s home is. I am not around through out, I am on and off.

I Okay. How have you tried to deal with these challenges?

R I try to be calm, avoid thinking a lot and keep my self busy with people I enjoy being around so that those thoughts do not cross my mind. If they come, I pray over it and try to avoid.

I To what extent have you succeeded in dealing with these challenges?

R I have tried. Sometimes when it’s too much, I share with my female friend. I share with her and she advises on what to do. I then confidently overcome them. Another time when something comes up, I also share with her. She gives me good advises and I am able to cope.

I Great. So you feel you have been able to deal with the challenges

R Not much, but I am trying

I What support is available to help deal with the challenges apart from your friend?

R I am this person who doesn’t trust easily. If I tell you something or I decide to be open with you, then I trust that you will not spill the beans. It’s hard to trust my family because they will not keep quiet. When I share with one of them, they find it hard to keep it to themselves, so they end up telling others and when it reaches my foster mum, it becomes another story. It is therefore very hard to share with them. I either keep it to myself or share with my friend.

I Great. I want us now to discuss on HIV care services. Could you please describe to me your experience the day you were enrolled to HIV care.

R I was enrolled to HIV care when I was 3 years old. At that time I was very sick and nearly dying. So my parents/guardian’s took me to hospital. By then my mum had not died but she was very sick. I had infections all over my body and was looking terrible. My foster parents ensured I took medicine daily and was fed very well. They are the ones who told me about this because by then I was very young.

I It was your relatives who took you to the hospital because your mum was not feeling well at that time

R Yes, it was my relatives

I Your mum died when you were how old?

R 3 years.

I Sorry about that. I am sure at 3 years you could not really tell what was happening

R No, I couldn’t

I At what age did you know that you were HIV positive?

R When I was in class five-six there I was disclosed to.

I By whom?

R My guardian who stays in Kisumu and my cousin who is also in Isiolo. We used to stay together with her at my grandmothers place before I moved to live with my aunt in Kisumu. She used to encourage me that I am not alone and that my other three cousins were also like me (also HIV positive and taking ARVs). She was always there for me. I used to cry a lot and wonder why me, it was not easy.

I The moment you realized that you are HIV positive, what went through your mind?

R Like I am not normal, I cannot live a normal life with people. I felt embarrassed, and at a younger age knowing that, I really got traumatized.

I How many siblings are you in your nuclear family?

R My father left when my mum died. The last time I saw him, he had another wife and 2 kids. My foster mum here has two daughters.

I Your mum only gave birth to you?

R Yes. I am her only son

I Okay. What has enabled you to continue engaging in care at this facility?

R At first I was in Mbagathi. They were very strict on health matters and when you miss they could call your parent to find out why you are not keeping your appointments and taking drugs as expected. We came here when my mum (foster parent) was transferred to come and work and I had just finished primary school waiting to join High School. I transferred my clinic from Mbagathi to KCH. That is how I ended up at KCH (Kisumu County Hospital)

I Okay. What has enabled you to continue to engage in care at KCH? Is it the distance to the facility? The staff? quality of services?

R What I can say is that the staffs are welcoming, friendly and the distance to the hospital from home is not far as I usually trek. The first time when I came I used to take a long time because of the queues. Children and parents were mixed

I Ooh, it was a mixed clinic?

R Yes. So you had to come early enough so that you are treated but now at least they have a section for children only. So, anytime you come, you will be treated and you will not take more than 2 hours. It also depends on the day because there are days also children come in numbers especially during the holidays.

I Could you please tell me an instance where you have been discriminated because of your HIV status?

R About my HIV status, I don’t usually disclose to everyone. I like to keep it low an only a few of my friends know it. The others only assume or think but they do not have evidence.

I Have you been stigmatized?

R Stigmatization, no. Just apart form the mockery about my physical appearance.

I That is great. Could you please tell me anything you know about ARVs?

R The ARVS?

I Yes.

R I knew about them back in the days when I was young. These drugs I was told that they really helped me when I was very sick. You can stay up to 20 years or more if you take them properly and you will not die. They have really helped but as you know, it can sometimes be a challenge because taking drugs daily is not an easy task. You have to close your mind and heart and say that this is my daily life and if I do not take them then I die. Its not easy waking up every morning to take those drugs and also in the evening, it is not easy but a matter of commitment.

I Any other thing you know about ARVs?

R Just that. You have to take them daily and you will live long.

I okay. Tell me about the last time you missed taking your ARVS. What happened?

R At times I engage in activities that take away my mind till I forget. Also, I can take a nap and forget to set an alarm or remind someone to wake me up. I therefore end up oversleeping and not taking the ARVS .

I Okay. Could please share an instance when you missed?

R Currently I am in upcountry. There is this day I was sent to the shops late in the evening. On my way back, I met my friends and we started catching up. I went back to the house at 9.00pm only to realize I did not take my drugs, which was due at 7.00pm. My grandmother was crossed with me for not taking the drugs on time but it was too late. I had to wait till the next day.

I Okay. Are there challenges you face with keeping your clinic visit appointments?

R As I told you, I just finished my school recently. You find that at times my clinic appointment was scheduled during school days and I was not able to get in touch with my mother to pick the drugs on my behalf because she worked in Eldoret, quiet some distance from Kisumu. This was challenging because I could miss my drugs even for 4 days before I could get refill.

I Does that mean you did not have any other person to send?

R No, I didn’t. Even when I was in Nairobi it was challenging. I had to wait till we close for the holidays then come for enough drugs to push me through the term. At some point I lost the yellow clinic card and it became very difficult to access drugs form another clinic but I finally made it home and got refilled at Mbagathi. Again when you keep on asking for permission from school it raises a lot of questions which I was trying to avoid.

I Okay. So, the challenge was that you are not able to get someone to send to pick up your drugs.

R Yes.

I Mmmm, When is the last time you missed your clinic visit appointment?

R I missed my appointment around April because I had traveled to Mombasa to visit my aunt. However, I went to Shimoni Health Center for refill and reported to my clinic when I came back.

I What are you general feelings about the services you currently receive here?

R The services are really good. The staffs are friendly and very supportive.

I How does being in school for young people like you affect their keeping of clinic appointment

R Sometimes you do not want people to suspect you have that condition. You therefore have to come up with something to cover up for that. So you miss your clinic deliberately because you do not want a lot of questions. Its like the there are people who are just on your case or wanting to know what happens in your life.

I Okay. Apart from the fear of being questioned, what else can affect young people from keeping their clinic appointment?

R Friends. When you have friends it becomes very difficult to get out of their site because most often you spend time together. It’s even harder when you are supposed to visit the clinic because they will be interested in your whereabouts. It is therefore safe to keep it low

I Okay. So, you think being in school/college will affect clinic attendance among young people

R Yes. There are people whose eyes are on you such that you can’t excuse yourself to go out and take your drugs. They will keep questioning, even your friends. So it becomes difficult while in school or college to attend your clinic as scheduled especially in boarding schools. Also when you keep asking for permission and you haven’t disclosed your status to the teachers because of one reason or the other, it raises eyebrows.

I Okay. I want us to move on to discuss sexual and reproductive health. What do you understand by the term sexual and reproductive health?

R Sexual and reproductive health entails body growth, both physical and emotional, that every individual has to pass through to become a mature woman or man. It also entails how conception takes place, prevention of unexpected pregnancies and prevention and treatment of sexually transmitted diseases.

I Great! You have the right definition. Just to add on what you have said, sexual and reproductive health also entails safe sex, access to sexual and reproductive health information, STD/STI prevention. How important is your access to sexual and reproductive health information and services?

R It really helps because I get to know that there are various diseases, how I can prevent myself and partner from getting these diseases, how to prevent unwanted pregnancies and if I am not able to abstain from sex then how I can do it.

I Okay. Currently, where do you access information on sexual and reproductive health?

R My aunts and foster mum. They are the ones who advise me on safe sex and those things.

I How about sexual and reproductive health services, where do you currently access them?

R Condoms are everywhere nowadays. I get them from the hospital or when I am upcountry I get them from the community library.

I Okay. Where is the ideal place you would love to access information and services in regards to sexual and reproductive health?

R It can’t be really a bother where you would want to access them. Sometimes things happen when you least expect. So, I usually have condoms and travel with them just incase

I Where would you prefer to access those services?

R I would prefer to get them from the hospital because that is the same place I get my ARVS.

I Okay. Do the sexual and reproductive health services offered to young people like you meet their expectations and needs?

R On a scale of 1-10 I can say 5. I say 5 because not all youths/adolescents get to meet their expectations with the information and services offered. Like some don’t like using condoms however much they are offered, they prefer to have sexual intercourse without condoms because they term it as “sweet”. Others will also not use the family planning methods offered.

I Okay. So you would give it 5 to mean half does meet their expectations and the other half doesn’t.

R Yeah

I What challenges do you as a person face in trying to access sexual reproductive health information and services?

R On information, I usually rely on my guardians because they want me to grow up to become a descent man but sometimes they tell me that I am young and should not engage in sex that when they were my age they did not. That I should wait till I am maybe 25 years old. However, I feel that was their time but now its my time and a totally different generation. I don’t have a challenge with accessing the services though.

I How does being in school/college for young people like you affect their access to sexual reproductive health services and information?

R It doesn’t affect that much. If you are a person who for example does not want to infect the others then you would take the necessary measures so that no one should have the slightest idea of what is going on in your life. Its about you making a choice on whether you want o access the services or not, so school/college doesn’t affect.

I So, you are saying school does not affect but it all depends on you?

R Yeah, school doesn’t affect much it is you decide on the best course of action. If you decide to infect people then its your choice, if you decide not to then you know where to access the services and plan yourself on how to go about it.

I Okay. For example a young girl is in school or college and they want family planning. The fact that she is in school, does it affect her access to the FP services?

R You know girls are different in a way

I (Interjects) I was just using them as an example

R In general

I Yes.

R I don’t think it affects. No

I Okay. How comfortable will you be discussing your sexual and reproductive health concerns with a lay female healthcare provider, someone of your mothers age?

R I am always free discussing my issues with people who are trust worthy.

I So you will be confortable?

R Yes

I Okay. What issues will you be comfortable discussing with this person?

R Many issues

I In relation to sexual and reproductive health?

R Issues concerning my sex life. Normally I prefer to have sex with those who are slightly older than me … but I can discuss with her everything even when I have issues with my girlfriend.

I Okay. Apart from issues concerning your sex life what other issues will you be comfortable discussing with her?

R If I have any questions in regards to sexual and reproductive health , I can always discuss with her.

I Okay.Where would you want such discussions to take place?

R Anywhere private. It also has to be conducive. There are places that are private but not conducive because some people are around, “walls have ears”

I Anywhere private like home? Hospital?

R Yes, could be hospital or home as long as it is private.

I If we assigned you a lay female healthcare provider to be your confidant and source of information on sexual and reproductive health whom you earlier mentioned you will be comfortable with, what attributes would want in this person?

R I want someone who is lively, someone who loves jokes and not a serious person because I fear them. I want someone who is as open and free with me

I Any other attributes?

R No, those are the main ones.

I Okay. How would you feel if you received your sexual and reproductive health services together with your ARVs at the same point? An example is when you come for your ARVs and you are also issued with condoms at the same place or someone comes for ARVs and also issued with family planning method of their choice at the same point

R I feel its okay. It would be good but also will depend on the place in terms of privacy but generally I feel that is okay.

I What are the advantages of receiving your ARVs together with sexual and reproductive health services together?

R The advantage is that I will keep on reminding myself that I need to protect self and others. You know having a family at a tender age is not easy so it is safe when you access these services together.

I Okay. Any other advantage?

R Mmmmm, only that.

I Okay. How about the disadvantages of receiving your ARVs and sexual and reproductive health services together?

R The disadvantage comes when I take a lot of the sexual and reproductive health services like for example condoms and I don’t use them regularly. I just like company, someone I can talk to and not necessarily have sex with them. My parents will also wonder what I am doing with them and start looking at me from a different angle. That’s the main disadvantage

I Okay. Thank you very much. Do you have any questions?

R No, I am okay.

I Great. Thank you for your time.

R Welcome

Study: AYA

In-depth interview

Location: Lumumba

Name of interviewer: Harriet Fridah

Name of transcriber: Harriet Fridah

PID: KLM 014

I Let’s begin by you telling us about yourself? (age, education, marital status, current occupation).

R I am seventeen years. Am still in High school. My name is S………….

I What are the challenges that you face as a young person?

R I have been a given the responsibility of a Deputy President here at Kisumu County Hospital. Equally in school, our deputy teacher knows about my HIV status. He keeps for me my drugs and has allowed me to share with other students who are HIV positive my experiences and share with them what I learn at the clinic. Those who have challenges with taking medication often come to me so that we can share our experiences and learn from each other like the operation triple zero.

I Great. That is kind of you. How do you think these challenges affect your education?

R You see like now when we are together, people will start wondering why we are always together. Through this then one is forced to lie because you can’t tell everyone about your situation. Then sometimes you speak to someone then they don’t do what you have told them/shared with them.

I So, you find it difficult when you talk to people but they do not follow what you are telling them?

R Yes

I Any other challenge?

R None

I Mmmmm. How have you tried to deal with these challenges? For instance, you said that when you take time at the clinic people question what you were doing. How do you handle that?

R Yes. Where I study most of the people are affected with asthma. So, most of the time they normally fall down. I also pretend that I fall down so in most cases when I take medication, they know that I have asthma.

I To what extent have you tried dealing with these challenges?

R I also share a cube with one of the girls in my dormitory. Whenever she could see me take my drugs, she could ask me why other asthmatic people were not taking their drugs on a daily basis. I told her that my condition is complicated even though theirs is stronger than mine. She even used to insist that I open my mouth so that she could confirm the medicine I am taking but I had to tell her that and she should stop bothering me with her questions. Since then, she never asked me those questions till she finished high school.

I That was creative of you. Thank you for sharing your challenges and how you have been able to deal with them.

R Yeah

I Great. We will now move to HIV care care and services. Could you please describe to me your experience the day you enrolled in HIV care?

R I was raped when I was about 4 years old. Mmm I think I was 6 years old when that happened. I was taken to the hospital but he doctors said that I was okay as there were no signd of rape according to them. They did not even do any test on me and neither was I given medication. I started becoming sick when I was in standard 2. I could barely finish a week in school. My mother became worried and took me to Gur Nanak hospital this is where I was examined and tested. I tested positive for HIV and was immediately referred to District Hospital where I was initiated on ARVs.

I How did you feel when you learnt of your HIV status

R At that time I was a young girl and I didn’t know what it meant by being HIV positive. I was naïve and everything was normal to me.

I was very weak at that time and most of my friends deserted me. The moment I was put on anti-retroviral drugs, my health status improved and I gained my body back and now even my friends started associating with me. I remember when I was transferred to a new school I never had any friend because I had rashes all over my body and I was this thin and weak girl. When I did my first exams in that school, I emerged top from then I got friends and even people I could now talk to.

I How did you take it when you knew about your HIV status?

R I was very young by then and I took it normally. I was really supported with my parents and teachers because they knew my status. I could not do school chores like cleaning the corridors. I was prioritized over every other person. It somehow made lazy but I tried my best to cope with others.

I Okay. So, you made the decision of taking care of yourself?

R Yes.

I You mentioned that your parents are supportive. Do they always accompany you to the clinic?

R Yes, they do.

I What made you to start thinking positively about your HIV status?

R I remember there was a time I was sickly. My mother was crying holding my hands. In her mind, she thought I was sleeping but I could see her well. She could for go her work just to take care of me. This motivated me and I purposed in my heart to take my drugs so that I can be strong and healthy to achieve my dreams and support her in future.

I Great. That was thoughtful of you. What has enabled you to continue engaging in care at this facility?

R I was not attending any support group meeting till last year when my mother allowed me to attend. The first time I attended a support group meeting, I met so many people whom I was left wondering if these people were in a similar situation like me. They were happy, socializing, and sharing knowledge and this is where I learnt information that I never knew. This made me to become free with the doctors and everybody at the facility. I could explain any problem I had.

I Nice, what is the proximity to the facility? Is it accessible or quiet distant?

R It is accessible. When I started care here we were staying far from the facility but we recently moved nearer.

I How are the services at the facility?

R The services that I get here are very good. I get information from both the doctors and at the support groups. Recently I was told by the doctor to choose a convenient day to attend clinic which was a very good step. This is better compared to when the doctor decides for you the day you should attend the clinic.

I Great. How friendly are the facility staff?

R The staff are also very friendly.

I Can you tell me any instance where you ever experienced stigma or been discriminated against because of your HIV status? For example, someone not letting you do something or not wanting to be around you, someone not wanting to share your items?

R No I haven’t had any.

I Okay. Tell me anything you know about antiretroviral drugs (ARVs).

R I know that they help to fight the virus so that they may be low. If they are active, they will affect my immunity’s ability to fight diseases.

I Mmmmm. Thank you. Tell me about the time you missed taking your ARVs. What happened? 10.46

R This happened recently. I normally take my drugs at nine o’clock in the night. A certain teacher came to teach before we retired to bed. He taught us till 10.00pm. When it reached nine I couldn’t concentrate because my time for taking medication had reached and the teacher was not leaving either. She left at 10.00pm I just rushed to the dorm to remove my clothes from the hanging line. I just remembered that I did not take my drugs the next morning. I went and informed the school nurse who told me not to forget again. I had a slight headache that day..

I Kindly share with me the challenges you face keeping your clinic visit appointments?

R I have no challenges.

I Tell me about the last time you missed your clinic appointment

R That was a long time ago I cannot even remember.

I You can’t recall what happened?

R No, I cannot.

I Okay.

What are your general feelings about HIV care services you currently receive?

R Initially we used to share the clinic with adults which was a bit boring and time consuming. We could even stay there from morning to evening. Currently we have our own adolescent center. It’s very good because you just come and pick drugs and go. There are no ques

I Great. How do you think your encounter with HIV care services can be made more satisfying?

R It is just okay. I don’t think there is any other thing that I would need. I am comfortable.

I How does being in school/college for young people like you affect keeping their clinic appointments and taking their ARVs?

R You find that those that are in boarding schools they have to travel each and every time to come for their appointment. His affects them in a way because you are forced to ask for permission and you end up missing some lessons. I would prefer that when you are going to school you take enough drugs to take you through until school closes.

I Great. We want to move on form HIV services to our next area of focus on sexual and reproductive health. What do you understand by the term ‘sexual and reproductive health’? You can think of this in terms of access to sexual and reproductive health information, safe sex, contraceptive use, STI’s prevention.

R This is the keeping of the private part in a healthy or safe way. My teacher tells us that sex is not a must, you can wait till the right time and age. For the contraceptives am not sure but I think they are these implants that are planted so that when you have sex you don’t get pregnant. STI’s in full is sexually transmitted infection that are gotten only through sex. in order to prevent STI one has to use condoms or just abstain.

I How important is your access to sexual and reproductive health services?

R Like in our school we normally have meetings and talks and medics are normally invited and they talk to us on sex issues. Even our teachers normally discuss this. So, it is very important to me.

I Where do you currently access information on sexual and reproductive health?

R Okay am sponsored by DREAMS there we get every kind of information. We get information on entrepreneurship, sexual education among others.

I Where do you currently access services on sexual and reproductive health?

R I haven’t received any because I do not use them.

I Where is the ideal place/location where you would like to access sexual and reproductive health services

R Here at the facility because I am much free with the staff.

I To what extent are the sexual and reproductive health services offered to young people like you meet their needs and expectations?

R Most people in high school they are affected by these issues of boyfriend girlfriend relationship, so those that are from poor background tend to engage in sex to get money to sustain them in school.

I Does the sexual and reproductive health services offered meet their expectations?

R Yes

I What challenges do you as a person face in trying to access sexual and reproductive health Information and services?

R Okay I don’t face any because I do not use them

I Okay. How does being in school/colleges for young people like you affect their access to sexual and reproductive health services

R The nurses at the health facilities and schools are the ones who are supposed to give these services but you find that some of them are harsh. At times when you go there and you are diagnosed with a disease the nurse can gossip about it with the teachers. This can in turn lower one’s self esteem. This affects students access to these sexual and reproductive health services.

I How comfortable will you be discussing your sexual and reproductive health concerns with a lay female healthcare provider of your mother’s age?

R I believe that these older people have got more experience than us but not all of them. You will just look at that person and see whether you can share.

I What issues would you be comfortable discussing a lay female healthcare provider of your mother’s age?

R Maybe about sex and relationships if you want to engage. So you go and consult on how to handle such situations.

I Where and why would you want those discussions on sexual and reproductive health to take place?

R In schools, hospital and even at home should be encouraged. At times you may want to do a wrong thing but once you remember what you were taught then you start behaving accordingly.

I How would you feel if we assigned you a lay healthcare provider to be your confidant and source of information for your sexual and reproductive health needs?

R It will be okay but it will depend on the type of person .it should be somebody who is understanding and is ever serious.

I What would be your ideal attributes/characteristics of such lay healthcare provider to act as your source of information on sexual and reproductive health?

R He/she should be someone who keeps things to herself even if its parent who comes to look for information.

I You mean confidential?

R Ooh yes

I Any other attributes?

R None

I How would you feel about receiving your ARVs together with your sexual and reproductive services?

R It would just be okay.

I What would be the advantages and disadvantages of receiving your ARVs and sexual and reproductive health services together at the same point?

R For somebody like me the advantage will be that I will get both the ARVs and information about reproductive health.

I Any other advantage or disadvantage?

R None.

I Thank you very much for your time. We have come to the end of our interview

R Welcome.

Name of study: AYA

Type of Interview: Adolescents IDI-001

Date of interview: 14-May-2019

Start time: 1:30Pm

Stop time: 2:00Pm

Location: Kisumu County Hospital

Name of interviewer: Irene Okumu

Name of transcriber: Irene Okumu

**I**: Hi, my name is Irene Okumu, I work for FACES and currently I am conducting interviews, IDIs for adolescents and young adults on sexual and reproductive health on what they would like to be incorporated in their care and with is?

**R**: XXXX

**I:** Sorry

**R**: XXXX

**I:** So, we will ask you a few questions since we have gone through the consenting process. I would ask you a few questions and whatever you feel uncomfortable with you do not have to answer and we will make this short, if you have any questions you will let me know, if there is something that you do not understand you also let me know.

**R**: Okay

**I**: Thank you

**R**: Welcome

**I:**So I will have different categories of questions, I will have challenges that you have experienced, we will also have the stuff that you have gone through, your HIV care services, sexual and reproductive health{ respondent is coughing} and lastly views on potential interventions that you would like to be incorporated in what we are already doing as FACES. So first of all, tell me something a little bit about yourself, your age, your education, your marital status, occupation if you have any.

**R:** Thank you. I am called XXXX, a student at Tom Mboya, …second I take my medication… is called what.. Support group centre

**I:** Okay, so are you married?

**R**: No

**I:** You are not married?

**I:** What do you do apart from school?

**R**: Nothing

**I**: You do not do anything?

**R**: Yeah

**I**: Okay, so I would also want to know if you have challenges that you face as a young person

**R:** Yeah there is

**I**: For example?

**R**: Mind set of the public

**I**: You find that a challenge?

**R**: Yeah

**I:** Okay and how do you think these challenges affect your education and your professional achievements. Maybe we would say how does it affect your education first of all

**R:** First if you hear a little bit, some will abandon you as friends.

**I**: Okay

**R**: Yeah, so they will look at you as positive

**I:** What is your professional achievement, how does that give you challenges?

**R**: Loneliness

**I**: It gives you loneliness

**R:** And giving me stress making me uncomfortable with other people

**I**: With other people? And what about your socio economic advancement in terms of finances, your social life

**R:** About in our family?

**I**: No, just in general. Just you as an individual maybe in your family, maybe at school, with your peers, with your friends

**R**: No

**I:** It does not?

**R:** Yeah

**I:** Alright, and have you ever tried to deal with these issues, these challenges?

**R**: Yeah

**I**: How? At least one example

**R**: When it reaches time for taking my drugs, I’ll go in a private room where I stay

**I**: That’s in school, and what about at home?

**R**: At home my mum knows about my status.

**I**: Okay. So *….* *(Interjection)……*

**R**: I am just comfortable

**I:** Ooh you are comfortable with your mum?

**R**: Yeah

**I:** Alright. That is good and to what extend have succeeded in dealing with these challenges that you have told me about?

R: *….. (Silence) ….* Nothing much. I just assume them and just give them time and I do my own part

**I**: You do your what?

**R:** My own part

**I:** You do your own part?

**R**: Yes

**I**: Okay and have you found any support? What support is available to help you deal with these challenges?

**R**: First, here we have a group called Sunburst

**I**: Aha

**R**: Yeah, they always give us advice

**I:** Alright

**R:** How to care and take your medication at public place with people that don’t know your status

**I**: Okay

**R**: Yeah

**I:** And any other way apart from the Sunburst?

**R**: No

**I**: You do not have any other you only have Sunburst?

**R:** And some counselors here

**I**: Ooh you have some counselors here?

**R:** Yeah

**I**: Okay, so the counselors how do they help you?

**R**: Giving us advice on how to take drugs

**I**: Okay

**R**: Yeah

**I:** Alright and on your HIV care services, kindly describe to me your experience the day you enrolled in HIV care?

**R:** Yeah, it was not easy

**I**: Okay

**R**: Yeah

**I**: Okay, do you remember when this was? When was it?

**R:** 2003 and I was in class one

**I:** Ooh, you were in class one?

R: Yeah,

**I:** Okay, so how long was it after knowing that you are HIV positive?

**R**: From Seven years …… no five

**I**: Five years? Okay and when you knew about this where was this? How did you make the decision to enroll or someone else enrolled you, I mean how did you enrol into HIV care?

**R:** I was enrolled up with my mum first, she was taking drugs {the mother took him for enrollment}

**I**: Okay

**R**: Not knowing the reason why

**I**: You were just taking but you had no idea

**R:** So it reached 2003 when my mum and the second counselor told me about it though it was not easy

**I:** Okay, it was not easy?

**R:** Yeah

**I:** Ooh, mhm... so when you say it was not easy, I wanted to ask if there is anything that made it easy for you to enroll, I mean you ended up enrolling right?

**R**: Yeah

**I**: So but you were already taking medication?

**R:** Yeah

**I**: So I cannot talk about the enrollment part because you were already in medication anyway

**R:** Yeah, not knowing

**I:** Not knowing, okay. What has enabled you to continue engaging in care at this facility?

**R**: Support from both my parents

**I:** Mhm..

**R:** Yeah at the clinic

**I:** At the clinic?

**R:** Yeah

**I**: What about in terms of distance, how far do you live from this facility?

**R**: It is not far away, just from... About 40 kilometers from Awasi.

**I:** Ooh, you come from Awasi?

**R**: Yeah

**I:** Okay, so you have to use transportation to come and all that?

**R**: Yeah

I: Ooh so when you come from school, you go back to Awasi?

**R**: No. right now I am here in Kisumu.

**I**: Ooh you are living in Kisumu?

**R**: Yeah but from my primary I was coming from there.

**I**: Ooh so a long time you used to come from there but now you are in college, from college you board? Are you a boarder?

**R:** No

**I**: You are a day scholar?

**R:** Yes

**I:** So where do you live? Where do you come from when you come for your medication?

**R**: Right now?

**I**: Yeah

**R:** In Kisumu near school

**I**: Ooh, you live in near school?

**R**: Yeah

**I**: Okay. That is good and what about the quality of services that you receive here?

**R:** Good.

**I**: Good? What do you mean when you say good? Just a little bit on that

**R**: They are caring

**I**: They are caring?

**R**: Yeah

**I**: Who are caring?

**R**: The counselors and the doctors here

**I:** And the doctors here, mhm.. that’s good to know and also if possible, can you tell me any instance where you ever experienced stigma or you were discriminated against because of your HIV status.

**R:** *…. (Silence) ….* Nothing

**I**: Let us say for example, you want to do something and someone does not let you do it because you are HIV positive. Have you experienced something like that? Or you have not experienced something like that?

**R**: Yeah

**I:** Okay, and if you could tell me anything you know about ARVS, what do you know about them?

**R**: They are drugs that help us to prevent the chances of HIV to manipulate in your body *(The participant meant to replicate in your body)*

**I**: Okay,

**R**: Yeah

**I:** Nice and what about the time that you ever missed taking your ARVS? What happened?

**R:** The time?

**I**: Yeah, have you ever missed your ARVS?

**R**: Yeah,

**I**: Okay, what happened? What made you miss?

**R**: In high School, we went for school sport

I: Okay,

**R:** Yeah and we delayed and my drugs were at home, it forced us to sleep at the school and I did not carry my drugs

**I:** You came back late from the sports day and you spent the night *……(Interjection)……*

**R:** With the boarders.

**I**: Okay with the boarders.

I: Also kindly if you could share with me challenges that you face keeping your clinic visit appointments if there is any.

**R**: No

**I**: You have not missed your appointments?

**R:** Yes.

**I**: Okay, you always come for your appointment?

**R**: Yeah, the thing they are doing good, they keep on reminding us when it is two days to, they contact us

**I**: Contact you using what?

**R**: Telephone

**I**: They call or SMS?

**R:** SMS

**I:** Ooh so they send individualized SMS like “Hi XXX you need to come for your… (interjection)…..

**R:** Yes, for your clinic tomorrow

**I**: For your clinic tomorrow?

**R:** Yes,

**I**: So now that you came today it means you received a message like yesterday or two days ago?

**R**: Yeah.

**I:** Okay, okay and tell me about the last time that you ever missed your clinic appointment if there is any.

(**Nodding to mean he has never missed his clinic appointment)**

You never missed your clinic appointment. {Coughing}. What are your general feelings about HIV care services that you currently receive?

**R**: Here?

**I**: Yes here

**R**: It is good

**I:** Okay

**R**: Yeah

**I:** What do you mean if you say it is good {Giggles}

**R:** The kind of treatment like counseling

**I** : So the counseling and the treatment that you get here

**R**: Yeah,

**I**: Okay, How do you think your encounter with HIV care and services can be made more satisfying?

**R:** Sorry

**I**: Right now you are receiving HIV care right, how can you make it better

**R:** Make it better?

**I**: Yeah, how can you make it more satisfying to you as an individual?

**R**: Okay, I think there was a time, we had a group, I think it was in 2017

**I:** Okay

**R**: We had a camp for the youths taking drugs here and they took us near Nyabondo. It was good, we really enjoyed from that. So here, we have not seen such a thing. I don’t know what happened

**I:** Okay,

**I:** So you would like something like that to be incorporated within the care?

**R**: Yeah

**I**: Okay, Like how frequently if we were able to?

**R**: A year

**I**: Like in a year about once**?**

**R**: Yeah

**I**: Okay

**R**: During the holidays

**I**: During holidays?

**R:** Yeah

**I:** Okay, I get it. So how does being in school or being in college for young people like you affect keeping their clinic appointments at taking the ARVS?

**R**: Sometimes you may be given a date, when you are doing your exams**.** So you find it a bit harder for the doctor charge to change the date

**I:** Ooh the date after hospital?

**R:** Yeah

**I**: Okay, because now exam dates are really strict

**R:** Yeah.

**I:** Okay,

**R:** So it makes it hard

**I:** Alright, any other thing apart from exams**?**

**R:** Nothing much

**I**: Nothing much, okay on sexual and reproductive health which is the major deal here, what do you understand by the term sexual and reproductive health, you as an individual**.**

**R**: Yeah

**I:** What do you understand?

**R**: By sexual reproductive**..**

**I**: By sexual reproductive health that term, sexual and reproductive health, what do you understand?

**R**: I think it is just when you cannot have sex

**I:** And what about in terms of access to reproductive health information or safe sex, contraceptive use, STIS and prevention if we talk in that context, what would be your understanding**?**

**R:** Reproductive,

**I**: Okay, so we do have the term sexual and reproductive health, right? So in sexual and reproductive health, we have reproductive information, we have got safe sex, we have contraceptive use. We have STIS and prevention. So in that context if I ask you about sexual and reproductive health, what do you understand, what comes to your mind?

**R**: They are matters related to sex**,**

**I**: Ooh, they are matters related to sex?

R: Yeah

**I**: Okay, any other thing apart from in relation to sex, any other thing that comes to your mind

**R:** Diseases

**I**: Diseases like what?

**R**: STIS

**I**: Like STIs

**R:** And HIV,

**I**: And HIV?

**R:** Yeah

**I:** Okay, and how important is your access to sexual and reproductive health services?

**R:** How?

**I:** How important is your access to sexual and reproductive health services?

**R**: {Silence}

**I:** I can reframe the question?

**R:** Yeah

**I**: Okay, so you said that in your head when you think about sexual and reproductive health services, you think about the STIS like HIV, you think about sex and so when I ask about the importance when I ask about how important is your access to sexual reproductive health I would like to know is you as an individual, when you access sexual reproductive health, what importance is that in your life? What does it do for you in your life? How does it help you?

**R**: Okay, it comes to knowing ones status

**I:** Okay, anything else in terms of let us say contraceptive, what would come into your mind?

**R**: (Inaudible)

**I:** Okay, do you know anything about contraceptive?

**R**: No

**I:** Contraceptive are ways of family planning which is like use of condoms like for women is different so for men I would say like use of male condoms, we have got other permanent ones like vasectomy so in terms of that what comes to your mind? How does that help you?

**R**: In terms of family planning

**I:** Yes like in contraceptive so it just reproductive health in general but you can use like safe sex, how is that important to your life or contraceptive the use of male condoms, how is that important to your life?

**R**: I think it is important because it helps to prevent ways of contracting HIV.

**I**: Okay, any other thing that it would prevent?

**R**: ( Silence)…..Unwanted pregnancies

**I**: Aha, any other thing that you might think apart from the two?

**R**: (Silence) contracting the STIS

**I**: STIS, so I would like to ask where you currently access the information on sexual and reproductive health, where do you get that information from?

**R**: Here

**I**: Here?

**R**: Yeah

**I**: Okay like when you say here like FACES is big like which particular part, where do you get that from? Like which side of FACES when you come the facility.

**R**: In the group where we normally discuss

**I**: Okay, there is a Sunburst group?

**R**: Yeah,

**I**: Okay, what would be an ideal place or an ideal location where you would like to access sexual and reproductive health services? A place you feel will suit you well to receive your sexual and reproductive from?

**R**: Just here at the clinic

**I:** Just here at the clinic

**R**: Yeah

**I**: Okay, and to what extent are the sexual and reproductive health services offered to young people like you meet the needs and the expectations like your needs and expectations, whatever is provided here does it meet to your needs? Does it meet your expectations?

**(Participant nodding in agreement)**

**I:** It does?

**R**: Yeah

**I:** If I could hear a little bit about that, how does it meet your needs or how does it meet your expectations?

**R**: First when we were being asked about the challenges *….(Noise in the background)…* confidence to express

**I:** Alright,

**R**: Yeah,

**I:** That is nice. And what challenges do you as a person face in trying to access sexual and reproductive health information and services. Have you faced any challenge as an individual?

**R**: No

**I:** You have not faced any?

**R**: Mhm

**I:** Okay. So since you are in school, I would like to know how this affects your access to sexual and reproductive health services.

**R:** At the school?

**I:** No, since you are in school right?

**R:** Yes

**I**: Does it affect your access to sexual and reproductive health services in anyway?

**R:** No

**I:** No it does not?

**R:** Yeah

I: Okay and how comfortable will you be discussing your sexual and reproductive health concerns with a lay female health care provider of your mother’s age? Do you understand that?

R: Yeah

**I**: How comfortable will you be?

R: *… (Chuckles)…*

**I**: Okay, like right now, your mum, not really your mum let us say at the facilicity or at home, there would be a health care worker who is not a nurse, who is a clinician right? Who is not a doctor but let us say like a counselor or a mentor mother kind of a person that you would be discussing with your sexual and reproductive health but they are in the same age as your mum though they are not your mum. Would you be comfortable discussing with such kind of a person?

**R**: No *…..(Chuckles)…..*

**I**: No, why?

**R**: I would feel a little bit shy

**I**: You would feel shy?

**R**: Yeah

**I**: Okay, but who would you prefer to discuss with?

**R:** Somebody from the facility

**I**: Ooh, somebody from the facility?

**R**: Yeah

**I:** So even if they are a female person the age your mum but they have to be from the facility?

**R**: Yeah

**I**: Okay, you just do not want someone from outside?

**R**: Yeah

**I:** Okay, even if everything you are going to discuss is going to be confidential?

**R**: Yeah

**I**: Okay, so what issues would you be comfortable discussing with this lay female health care provider of your mother’s age? Let us say if they are from the facility, what you would discuss with them?

**R:** …. (Chuckles)... Anything

**I**: Anything?

**R**: Yeah

**I:** Okay

**R**: I would be free

**I:** You would be free?

**R**: Yeah

**I**: Maybe give me one or two examples of these anything that you are talking about *….(Giggles)….*

**R**: Okay, matters related to sex

**I:** Okay

**R**: Like HIIV

**I:** You would be free to talk about that?

**R:** Yes

**I**: Okay, any other thing apart from matters relating to sex and HIV, is there any other thing that you would like to discuss?

**R**: No

**I**: No?

**R**: No

**I**: Okay, where and why would you want those discussions on sexual and reproductive health to take place?

**R**: At the facility

**I:** At the facility?

**R**: Yeah

**I**: Okay, why the facility?

**R**: It is a bit confidential

**I:** It is a bit confidential?

**R**: Yeah

**I:** Okay, so on your views on potential interventions like I told you before that we would like to incorporate interventions that would help adolescents in seeking sexual and reproductive health, how would you feel if we assigned you a lay health care provider to be your confidant and a source of information for your sexual and reproductive health?

**R**: I would be happy

**I**: You would be happy?

**R**: Yeah

**I**: *….(Giggles)…* Ooh, Okay. What would make you happy?

**R**: For that post?

**I**: Mhm

**R**: Yeah, I think I can *….( Inaudible segment)…*

**I**: Sorry?

**R**: I can do it

**I**: You can ….. Okay. Let me rephrase this question, so right now, you said you can speak to a lay health care provider, a female lay health care provider from the facility, let us talk *….(car hooting in the back gound)….* about that health care provider and we assign you one who is like your confidant, someone you can talk to when you want, someone you can confide in when you have a problem

**R**: Yeah

**I**: And this is the person who will give you information on sexual and reproductive health, you understand that?

**R:** Yeah

**I**: So how would you feel, if we decided that XXX, we have decided to assign you maybe Mary Anne *( Pseudonym)* to be your confidant, to be your source of information in case you have a problem, you go to her directly in case you need information you go to her directly, how would you feel about that?

**R**: Great

**I**: You would feel great?

**R:** Yeah

**I**: Okay and what would be your ideal characteristics of such a lay health care-provider to act as your source of information on sexual and reproductive health, what would you want, how do you want this person to be, what type of characteristics do you want to see in such a person?

**R**: First, she must be confidential whatever we discuss should not be shared

**I**: Okay,

**R**: Should not *….(inaudible)….*

**I**: Should not?

**R**: Carry it **(participant means to discuss with other people)**

**I**: Should not carry it?

**R**: Yeah

**I:** Okay, any other thing? Any other characteristic, just tell me all the characteristics you would like to see in that person?

**R:** Friendly

**I:** Mhm

**R:** Yeah, that is all

**I:** Just confidential and friendly?

**R:** Yeah

**I:** You do not want any other thing?

**R**: Yeah

**I**: Okay and what would you feel about receiving your ARVS together with your sexual and reproductive health services? Let us say like in this room where we are in, this is the sexual and reproductive health room right? So let us say the clinician was in here attending to you and we have the sexual and reproductive care services right here, let us say after you have take your medication all the care that you would need for sexual and reproductive health get it in the same room instead of being referred to another room, how would you feel about that? Would you want it to be put together or you want it to be separate?

**R:** *….(Whispering)….* Separate

**I:** You want it to be in a separate room?

**R**: Yes

**I:** Why is that? Any reason for having it separate and not in one room?

**R:** *….(Chuckles)….* just separate

**I:** Just separate, okay so what would be the advantage or disadvantage of receiving your ARVS and your sexual reproductive serves together at the same point?

**R**: Disadvantage?

**I:** Yes or advantage like yourself you have said you do not want, you want it separate?

**R**: Yeah

**I**: So to you it is like a disadvantage?

**R:** Yeah

**I:** So why is that?

**R:** Sometimes he or she may not be that person who is confidential so they may take the information and tell other people outside

**I:** Alright

**R**: Yeah

**I:** And what about if they are people who are confidential? Could that change your mind or you would still want them separate?

**R**: You may not know the mind of someone

**I**: You will not know their mind?

**R**: Yes

**I**: So, you still feel you want them separate; you do not want it together?

**R**: Yeah

**I**: Okay, thank you very much XXX for your time, we have come to the end of our interview and I would like to thank you for answering all of my questions and I hope whatever we have discussed here we will be able to make informed decisions with the information that we have. Thank you very much.

Our interview has ended. It is now 2:00 p.m and the date today is 14^th^ of May 2019. We are in KCH the sexual and reproductive health room.

I Good Afternoon

R Good afternoon

I My name is Irene Okumu and this is AYA study. The date today is 11^th^ July 2019 and the time now is 1445hrs, in this interview we will be discussing challenges that adolescent goes through during HIV care services, sexual and reproductive health and possible interventions that adolescent would like to be incorporated into the healthcare services. In case you do not understand anything, you can kindly ask it, I will repeat, rephrase or put it in a way that you can understand it better. If you do not feel like answering any question, you are not cohered to answer, you can just let it pass, alright?

R Yeah

I Okay, welcome to the interview.

R Thank you

I So first I would like to ask you to tell me about yourself, what your age is, your education, marital status and current occupation if you have any

R Okay, my name is xxx, I’m 22 years old

I What else?

R Uhm

I What is your marital status?

R For now I’m single

I Right

R Yeah

I Do you have a boyfriend?

R Yeah, I have a boyfriend

I Okay

R Yeah

I Do you have any kind of work that you are doing now?

R No, for now I don’t have any job

I *(Interjects)* You don’t have any job

R Yeah

I Do you go to college?

R No. I am just doing computer lessons

I You are just doing computer studies

R Yeah

I Alright, thank you. So as an adolescent or a young youth, what are some of the challenges that you face as a young person?

R Challenges in life or?

I General

R Mmh okay, some of the challenges I face are from the people who know that I’m taking drugs, sometimes they feel so bad, most of the times if I ask my friends their take on HIV they are the kind of people who brings stigmatization so I don’t enclose to them most of the time, most of the time I just keep quiet and they don’t know my status, some, and also some of the challenges that I face is that my aunt doesn’t support me

I Whom do you live with?

R My aunt and my uncle

I Okay and what about your parents?

R My parents died when I was a little girl

I How old were you then?

R I think three years

I Okay

R Yeah

I So you have been living with them all that while?

R Yes

I Okay

R But we have so many challenges with them

I Okay, and how do you think these challenges affect your education?

R My education?

I Yeah

R I think because my uncle does not have a stable job, during my schooling, I used to be sent back home most of the time because of fee arrears, so most of the time I was out of school until I had to transfer to as school at my grandmother’s place where I completed school from there.

I So that was a day school or boarding school?

R Day school

I Okay, so who was paying for you while you were at your grandmother’s?

R My grandmother

I Ooh your grandmother was paying for you the fee?

R Yes

I Okay, and have you tried to deal with these challenges in any way

R Yeah, sometimes I cry, sometimes I talk to somebody who understands me, like my best friend who is in Nairobi right now

I Okay, a girl or a boy?

R A girl

I Okay, so she understands you well?

R Yeah

I Is she also HIV positive

R Yeah

I Okay, do you take medication at the same place?

R No, she takes at Lumumba

I At Lumumba hospital?

R Yeah

I How did you meet?

R At a camp

I Okay, that is nice

R Yes

I So, for how long have you been friends?

R For long as I can remember

I For long as you can remember?

R Yeah

I So, anytime you feel like you have a lot of issues you talk to her

R Yeah

I And you share with her

R Yeah

I How do you feel after that?

R After talking to her I feel relieved and fine

I Okay, that is a good way to deal with things at times

R *(Interjects) yeah*

I Just talking to let it out, okay, to what extent have you succeeded in dealing with these challenges?

R I can remember there was this time I refused to take my medicine and my aunt was forced to call one of the leaders to come and, what is it called?

I Counselling

R Yeah counselling

I Okay

R Yeah

I So they came and talked to you?

R Yeah, they came and talked to me and they also talked to my aunt and I started to see reasons why I should continue taking the drugs

I Okay, why did you want to leaver them at that time?

R I was just tired of them

I You were tired of the medication?

R Yeah

I Uhm, is it because you take them everyday or you just got tired of them?

R The reason was that I was the only one who took medication in that house

I I mean, but your aunt knew the reason why you took the medication?

R Yeah

I Okay, but after you were counselled did you pick up well?

R Yeah, I picked up well and used them

I Okay and since then you have just been taking your medication?

R Yeah

I Okay

R Right now I don’t have any problem with my medication

I You don’t?

R Yes

I Nice, that is good, so what support is available to help you deal with these challenges?

R I think meetings and when you meet people with the same status as you can help

I Okay, so when you say the people like you, where do you find these people?

R At the facility, yeah, at the facility or in the groups

I You do have groups?

R Yeah

I Ow, you have support groups?

R Yeah

I Wow, tell me something about that

R *(Laughing)* Support groups motivates us, they teach us a lot of stuff that we don’t know in real life like in real life most people do not know about PrEP

I True

R Yeah and PEP

I So that is discussed in those groups?

R Yeah

I Do you guys meet physically or there are other ways that you meet?

R We have a Whatsapp group then we meet either once or twice in a month

I Okay, you meet physically?

R Yes

I Ow, that is nice, so I would also want to ask you questions about HIV care services here in the facility, and I would like you to describe to me your experience the day that you were enrolled into HIV care if you could remember

R Mmh, when I came here I did not know the reasons why I was taking those drugs, but they treated me well, they welcomed me, they tried all they could to support me in any way, yeah and somehow they made taking the drug to be fun *(laughing)*

I Okay, and when was this, do you remember?

R No, I can’t remember

I You can’t remember?

R Yeah

I but do you remember who brought you in for medication?

R Yes, my aunt

I Your aunt is the one who brought you?

R Yes, that happened after I was found with the disease, we visited several hospitals because they did not know what was happening to me, so we walked from different hospitals until one of my aunts suggested that I should just go to the VCT and be tested and that is how I bought

I So where were you tested?

R It was not here, it was, that hospital near Kibuye

I Russia? (JOOTRH)

R No

I Marie stopes?

R No, it is no longer there

I Which one was it?

R I don’t even know but we were given transfer to come here

I Tuungane?

R Yeah Tuungane

I Okay, okay, alright, what has enabled you to continue engaging in care at this facility?

R Their services are just wow

I Alright

R and they treat people well

I They treat people well

R Yeah

I What about in terms of distance, how far was it?

R In distance it is not far

I Is it like a walking distance or how much do you use to come here?

R Around fifty shillings

I Around fifty shillings?

R Yeah

I and can you tell me of any instance where you ever experienced stigma or you were ever discriminated against because of your HIV status

R I can remember severally when I was in form two third term, we had issues with my aunt then I ran away from home and then I left all my medicine there so somehow she noticed that I was staying with another man, but I was not staying with any man but they knew the guy, so they came to his place and beat him up then my aunt, I’m not sure if it was intentionally or not, you know such issue, everyone came to know about it because she shouted that I even take the ARVs, and forgotten the drugs at home, so where I stay now, people don’t talk to me because they know I’m HIV positive so yeah, that stigma is still there

I Okay, so you leave in a plot kind of a place?

R Yeah

I So no one talks to you in that compound?

R Yeah, no one

I Okay, so the person who shouted was your aunt?

R Yes

I and she was saying that

R *(Interjects)* that is what I heard because I was not there

I Oh

R Yeah

I So you were not in that house when your aunt went and attacked?

R *(Interjects)* Yeah, I was not there

I Sorry for that experience, so...

R *(Interjects)* and also in school, yeah in school the day school, some of my friends came to know that I was taking drugs so rumors started spreading in the whole school, so if someone wanted to talk to me they feared and even said I’d infect them so I told them I could not infect them even if they helped me with things like razor blades but they never helped

I So, how did they come to know about your status, did you ever tell them?

R No, I never told them but you know sometimes when I did not show up in school they were curious so they wanted to know why on specific dates I was not in school then the principal of that school also was careless, because at times when I sought permission even when he was in front of the students he’d say its okay, you can go to the hospital, so people used to ask me what I kept on going to do at the hospital

I Alright, so on ARVs, tell me something you know about ARVs

R ARVs, I know that they make my virus to sleep and they help me not to get sick, I am also protected

I Okay, any other

R That is it

I That is it?

R Yeah

I You don’t know any other thing about it?

R No

I Alright, and have you ever missed taking your medication?

R Yeah

I What happened that you ended up missing your medication?

R Mmh, I was, I went to a party and that time I was taking my medication at 6

I 6 am or pm?

R 6pm in the evening

I Okay

R So, I forgot to carry it and that place was far, so I had to miss

I Okay, any other time that you missed?

R Mmh, when I was with my boyfriend

I What made you miss?

R He was there

I Okay

R and also, I had not told him yet

I Alright, and were you able to disclose later?

R Yeah

I So, now you can take it in front of him comfortably?

R Yeah

I Okay, nice also you can share with me the challenge you face in keeping your clinic visits or appointments

R The challenge?

I Yeah

R I don’t have any challenges in that

I Do you miss your clinic visits?

R Yeah

I Okay, what makes you miss this clinic visits?

R When sometimes I have the medication, I just assume that the time has not yet reached for me to go back or sometimes when I travel

I but now, how do you deal with that now? Even if you still have medication?

R Right now I give information that I won’t be available on time and they give me advice on where I can take them from

I That is nice, also tell me about the last time you missed your clinic appointment, what happened

R Lat time?

I Yeah, the last time you missed your appointments, if you could still remember

R It was just recently and when I came back I was asked where I got the drugs and I told them that I got them from a clinic in Nairobi but I don’t remember the name so by the time I came back the arguments were like why I didn’t tell them earlier that I would not come, something like that

I Okay, so this place that you take your medication, what are your general comments about HIV care services you receive?

R General?

I General feelings, how do you feel about the services being offered?

R General feelings, Mmh I think they are the best people, they try to help

I Okay

R and I love this place

I You love the place?

R Yeah

I Why is that?

R Because of the support groups and the camps, and also the friends that I have made

I You have made a lot of friends?

R Yeah

I That is good, how do you feel your encounter with HIV care services can be made more satisfying

R Maybe if they don’t put it in an open place, because in an open place people might know that that place is for HIV people, so they know

I Okay, within your age group, say after 24 years you will be treated as a full adult

R Yeah

I So, before that time that you are transferred to the main clinic, how would you want us to make it more satisfying just before we move you but just within here

R Just to bring more camps

I More camps?

R Yes

I What else?

R Activities

I Like what kind of activities?

R Let say picnics and these activities that people do at the camps like skipping the rope, and such things

I Okay, wow, and how often would you like these activities organized?

R Once in a month

I So, in a year you want like 12 activities *(laughing)*

R Or let’s say, Mmh after three months

I After three months, right? I think that would make sense

R Yeah, after three months

I You told me that you are not like in full college, but you are doing computer studies, right?

R Yeah

I How long do you stay in school while doing that?

R Two months

I Two months?

R Yeah

I So, during the day, what time do you get in class?

R Around 11 to 1pm

I So, how does being in that college for you or young people like you affect keeping your clinic appointments or taking your ARVs?

R I t doesn’t affect

I Okay

R Because I take my medication during the night and the clinic I come in the morning

I Okay, so, in sexual and reproductive health I would ask you a few questions and I would like to know what you understand by sexual and reproductive health?

R Sexual?

I and reproductive health yes

R Sexual and reproductive? For my own knowledge it is about sex

I Okay

R Yeah, that is all I can say about it

I That is all you can say about it

R Yeah

I Okay, and what about access to reproductive information?

R About family planning or?

I Yes just like that

R Family planning, personally I don’t really like family planning

I Okay, why is that?

R Because of rumors maybe

I For example which rumor?

R Mmh, okay if you are given a family planning method then when you remove it you might not become pregnant

I Okay

R And them most people say they over bleed

I And what about safe sex?

R Safe sex? You just use condom

I Condom, okay and you are okay with that

R Yeah

I Why is that?

R I always feel like if a person uses a condom, they despise you or they think you are dirty, something like that

I Okay, what about in terms of STIs and preventions of STIs

R STIs?

I Yes

R What about them?

I Like, so STI is also part of sexual and reproductive health, do you know anything about STIs?

R Yeah

I Okay, what do you know about STIs?

R That once somebody is infected and you have sex without condom you can also get infected

I Alright, so that matter that you have just told me about safe sex using a condom that you feel that it is not good because someone feels that you are dirty, when you compare the two, what do you think about that?

R *(Laughing)* I think my own view was wrong

I You get the idea

R Yeah

I So safe sex, you realize that contraceptive use by use of condoms, that is also a form of contraceptive, do you know that?

R Yes

I It can prevent you from getting diseases and STIs and it can prevent pregnancies and infections and such like stuff

R Yeah

I Now you get the idea, right?

R Yeah, I can now get the idea

I So, to you as an individual, how important is your access to sexual and reproductive health services?

R How important?

I Mmh

R Pardon

I Okay, have you ever used sexual and reproductive health services?

R Yes

I Okay, what have you used before?

R Just condom, but personally I’ve not used one

I Like any contraceptive?

R Yeah

I and what about in terms of information?

R Information I have heard severally

I Okay

R Like the one for injectable, the one they put here

I That is an implant

R But I don’t know their names because I have never used them

I That is okay

R And then the pills

I Then the pills?

R Yeah

I Okay, and in terms of access to information, how do you get this information?

R Mmh, my aunt used the pills but she over-bleeds and she uses implant, you said implant, right?

I Yeah, the one for the arm

R I hard it is okay, but it depends with how your body will take it

I It goes with hormonal imbalance

R Yeah, if the body does not accept it, it can be bad for you

I Yeah, okay and do you have any other way that you receive this information apart from just knowing that my aunt is using pills and depo and all that?

R Yeah, they gave us some teaching on that subject

I Okay, where did you get the teaching from

R Just here in this clinic

I Okay and where do you currently access your sexual and reproductive health services?

R Currently?

I Yeah

R Just within the clinic

I So, what kind of services do you come for?

R Condom

I Condom services?

R Yeah

I Okay, that is fine, so when you come to the clinic they just give you or you have a specific place where you get them

R We have specific place like here you have to come to the nurse

I okay, that is nice, so, let us say that for this section of reproductive health services, where is a good place where you would like to access these services?

R Mmh, of sexual and reproductive health?

I Yeah

R At a clinic

I At a clinic?

R Yeah

I Any other place?

R I can say at KCH

I KCH the whole hospital?

R Yeah

I Wow, okay, so, to what extent are the sexual and reproductive health services offered to young people like you, meet the needs and expectations?

R Mmh, to meet the needs how?

I Like when you receive these services, do they help you in any way?

R Yeah, they have

I to what extent?

R To the extent that it is fare and convenient to me

I Okay, and have you had challenges as a person trying to access sexual and reproductive health services and information?

R No, I don’t think

I You don’t think so?

R Yeah

I Alright

R Because they are always open

I Who are always open?

R All the support staff

I Okay, that is nice, so you told me that you were doing some college and we asked this before, but we would also like to know for you as a young person or you as young people, how does being in school affect the access to sexual and reproductive health services

R It does not affect because it is only two hours that I am in school

I Okay, and do you think it would affect if you could be in a school where you stayed from say morning to evening?

R Yeah

I Okay

R Because that time you would like to have time, or let’s say if you are in a boarding school, you will not have time

I Okay, and how comfortable would you be discussing your reproductive health concerns with a lay female healthcare provider of your mother’s age, but now yours I’d put of your aunt’s age or someone who is your peer or younger than your aunt maybe

R Who?

I Okay, let’s say right now we decided that okay XXX we are going to give you a lay healthcare worker to be your confidant, say someone your aunt’s age or someone who is a little bit older than you but younger than your aunt, how comfortable will you be discussing sexual and reproductive health with

R If the person is nice I will be comfortable or but if not, I won’t be comfortable

I You won’t be comfortable?

R Yeah

I Okay and where would you be comfortable discussing these things with?

R let say somebody who is two years older than me or three years older than me

I Okay, so where would you be comfortable discussing? In terms of location, place

R Mmh not in an open place, in a closed place like this one

I Like this one Mmh *(laughing)*

R Yeah

I Okay, so where and why do you want those discussions on sexual and reproduction health, so probably you’d start with where

R Where, I would want to be?

I Yes

R Somewhere private and the person I trust

I and why would you want that

R because sexual reproduction health, some of the things you ask are secrets and need a place like this and most people don’t know about it

I Okay, so, we have been going on well and we are almost ending our interview, I would like to get your views on potential interventions, when I say interventions is like things you would like to be done to be able to access your care well, both ARV and sexual and reproductive health. So first of all, how do you feel if we assigned you a lay healthcare worker to be your confidant and source of information for your sexual and reproductive health?

R My healthcare provider?

I Yes, someone who is not a nurse or clinician, they do not have like a full medical background but have knowledge on the same that we would want this person to be your source of information, to be your confidant

R and then, if they are the same status as me it will be okay

I So, for you one of the things is that you want someone who is HIV positive, so how would you feel if we decide to assign to you someone like that?

R I will just be comfortable with it because you have said he/she will have knowledge on this sector

I Yes, just not a nurse or clinician but have knowledge

R *(interrupts)* but they have knowledge of these things

I Yes

R I will just be fine with it

I Okay, so, when we decide to assign you this person, what would be the ideal characteristics of this person

R Polite, like you *(laughing)*

I *(laughing)* Okay

R and funny, they should not have a serious face all the time

I Alright, you want them to smile

R Yeah, they have to smile so that you don’t get bored

I Any other attribute or characteristic that you would want them to have?

R Mmh…polite

I You have said polite

R Respect my view

I Alright

R They don’t laugh when I say something that I’m not sure of

I Okay

R And then the person should be ready to shar anything that I will ask

I Okay like to give you answers and responses to your questions?

R Yeah answers

I Okay, that is good. How would you feel if you received both your ARVs and sexual and reproductive health services? Like a one stop shop kind of thing

R Mmh they are just fine because they are somehow related

I That is true

R So, it will be just good for me

I That is you are okay if they are offered at one place like that?

R Yeah

I Okay, that brings me to the last question, what will be the advantages of receiving both the ARVs and sexual and reproductive health services together at the same point?

R Because we don’t have to walk from one place to another, it will be like killing two birds with one stone

I One big stone?

R Yes

I as we know in life, everything that has an advantage has its disadvantage, so what are the disadvantages

R Of taking?

I Of king the ARVs and sexual and reproductive health services at one place

R I can only think of one, maybe because somebody may take you as a loose person say if you have come to take condoms or you have come for contraceptive injections they will say you were not responsible

I Okay

R So that is a disadvantage

I from your perspective do you think that is true?

R Yeah, I think it is true

I It is true that when you come for sexual and reproductive health services you are a loose person, or you are not responsible?

R Maybe if you just find someone who is not learned

I Okay, is there anything else you would want to tell me before we close the interview?

R Mmh, yeah

I Go ahead

R About sexual reproductive health, which family planning is good for a lady who has never given birth?

I So, that is for family planning?

R Yeah

I That one is usually depends with a persons hormones, people are different so what one person can use is not necessarily what the other uses, usually what is good when you come to the clinic you talk to a clinician who is in charge or the nurse in charge and they will talk and advice you on which method to use, we have some which are hormonal and some are not, we have pills, depo, there is another one

R There is one when injected with one does not have her periods

I Yes, but it depends as I have said, it is just the imbalance in hormones, so what usually happens s that the nurses will advice you so after that they will tell you on which one to use

R But it takes a while to get pregnant

I It is true, but normal body function resumes but you have to be checked frequently, and the other one is a condom, you get my point, right?

R Yeah

I or it just depends with the hormones so when you come to this particular clinic and ask them those questions one by one they will give you the answers you need, personally I do not have a full medical background but the little information that I’m giving you is not full information okay

R Okay

I Thank you very much for your time

R Thank you

I *(interrupting)* sorry you were saying something, kindly go on

R Thank you

I It is okay, so thank you very much for your time and thank you for answering all my questions, it was nice having you and we have now come to the end of our interview and the time now is 1525hrs. Thank you very much

Name of study: AYA

Type of Interview: IDI 021

Date of interview: 16^TH^ JULY 2019

Start time: 1240hrs

Stop time: 1323hrs

Name of interviewer: Irene Okumu

Name of transcriber: Nelson Ouma

I Welcome to today’s interview, the date today is 16th July 2019 and the time now is 1240hrs and my name is Irene Okumu, this is AYA study and we would now like to start our interview, be free, in case you don’t understand anything, let me know. In case you have a question, you are free to ask, and I will answer you, welcome.

R Okay

I We will be speaking about challenges that adolescent experience, we will also talk about HIV care services, sexual and reproductive health and on possible views on potential interventions that you would like to be included in your care. So first of all tell me something about yourself

R Okay, so I am happy to be here, today I am happy I was called for this interview and I have been waiting for something like this to explain about myself to other adolescents, so I am happy to be here

I So what is your age? How old are you?

R 22 I am turning 23 in October

I and what is your level of education?

R form four

I You have finished form four

R Yeah

I Have you gone to college?

R Not yet

I Not yet, what are you currently doing?

R Just some business, like selling clothes

I Okay. And your marital status, are you married?

R Not really but girlfriend

I You have a girlfriend?

R Yes

I That is still fine

R Okay

I Do you live with your girlfriend

R I do live with her

I And you have not married her?

R *(Laughing)* I have married her for one week

I So you marry her for one week then she goes back?

R No

I Okay, so also as an adolescent, what are some of the challenges that you face as a young person

R First I realized that I was taking drugs in 2006, that is where I started having a lot of questions in mind, yeah so as a young person I was feeling uneasy especially in gatherings so when it was time to take drugs it was so hard, even in school

I Were you in boarding or day school?

R I started in boarding school but because of that I moved to a day school

I Your parents are the ones who transferred you or what happened?

R Yeah, I was born with it so I realized that when my mother died in 2003, that is when I realized that I was taking these drugs

I And your dad

R My dad is there but my mum passed away

I Your mum passed away?

R Yeah

I So you lived with your dad all that while when your mum died?

R Yeah

I Okay, and so these challenges that you have, how did these challenges affect your education?

R They didn’t affect my education that much because at that time I had started accepting what was going on with me, so I didn’t that much a problem of having education

I Okay

R Yeah

I And you told me that you sell clothes

R Yeah

I How does that affect you?

R It just affect me with time

I Okay, what about time?

R Sometimes, for me I take drugs at 9pm and that time is the same time that i am in my business

I So 9 pm that is at night?

R Yes at night

I So you work at night?

R Yeah, I start selling at 5 pm

I Ow, you start selling at 5

R Yes

I Where do you sell?

R Just here near Kisumu boys

I Ow, by the wall

R Yeah

I So what do you sell there?

R Just ladies’ clothes and babies

I That is nice, okay and how have you tried to deal with these challenges that you are telling me, like taking your medication and all that

R Just to first you have to accept that you are taking it, for me I don’t care, I just carry with a bottle of water and taking a drug so anyone who sees me taking it does not take it seriously

I So currently you take your drug at what time?

R At 9 pm

I Just at 9 pm still

R 9 pm and 9 am

I Oh so you still use the two dose one?

R Yes, I started it once, but nowadays I take once a day

I The one for once

R Yes

I You take it at what time? Or you have maintained at 9pm

R Yes

I Now I get it, so to what extent have you managed to deal with these challenges?

R Come again

I The challenges that you have, what extent have you managed to deal with them?

R I am still dealing with them, I have not exhausted dealing with them

I You are still struggling with them

R Yes I am still struggling with them

I Okay, no problem, so what support is available to help you deal with these challenges?

R Sometimes just, when I want to take my drugs I feel like it is time but I have my girlfriend who sometimes is there for me, she is the one who keeps me going in using these drugs

I So she knows about your status?

R She knows

I Is she HIV positive or negative?

R She is negative

I But she really supports you with your medication and everything

R Yes

I Okay, so we can say that she is your support system?

R Yes

I Which other support system do you have?

R My dad

I Your dad?

R Yes

I How does he support you?

R He started supporting me when I was still young and up to now he is still concerned about me

I Does he stay in Kisumu too

R Yes

I And do you stay alone or you stay with your parent?

R I have my own house

I You rented a house?

R Yes

I So the job that you do is sufficient to cater for your needs?

R Since I do it alone, it really helps me

I Okay, so on HIV care services, I want to know if you can describe for me your experience on your enrollment date if you could remember

R Experience?

I Yes, how was it?

R The experience on that day was that I was being taught about HIV, how to manage it, that was the experience I had from the facility

I How old were you at that time?

R I was in class two at that time

I Your mum had already died?

R My mum died as I was about to join class 1

I When you were joining class one?

R Yes

I So its your dad who brought you here?

R Yes

I Okay, so what was running in your mind at that time?

R At that time I did not know what was going on or what I was being counselled on

I True, you were still young

R Yes, I was still young

I Yeah

R So when I realized the purpose of taking the drugs, I was already in class six or seven thereabout

I Who told you?

R I used to take my medication but asked a lot of questions, I used to wonder why I used to take the medication

I And then it was daily

R Yeah, so at times I would get angry and ask my dad why I was the only one among my sibling who was taking the medication

I You are the last born?

R No I’m the first born among the boys

I And do you have sisters older than you, how many kids are you?

R In our house we are 3

I Three?

R Yeah

I And you are the last born

R There is a girl then I have a younger brother

I So the last born is a boy

R Yes

I Does he take medication too?

R In the whole family, I am the only one who uses medication

I Okay

R So when I realized at that time, I started asking a lot of questions like I was born with, so I asked my dad, between my mum and himself, who had the disease? So he was also affected and he had to tell me that mum is the one who had it, so I was born with it, it really disturb me when I thought and asked myself why me only, I had even sometime back stopped using the medication because I was even tired of it, I had some stupid friends because when you sit with them, that is the only story they want to tell you.

I Just a story from nowhere?

R Yeah just a story from nowhere like in that group there is a person who dated a girl who was infected so that becomes a whole subject of discussion and I was never happy about it

I Okay, and what made it easy for you to get into care?

R The facility made things to be easy for me

I How was that?

R Just teachings, the teachings were easy and before that I did not care about anything, I regarded that the life was mine

I You decided to live your life?

R Yeah so up to now I don’t see it as a big deal

I In terms of distance, where do you stay?

R Now

I Yes

R I stay here in Nyalenda

I So you use a *tuk tuk* to come to the clinic?

R Yes

I What about sometime back

R I used to come from home direct

I Where is home

R It’s like some six kilometers to town

I Towards Mambo or Rabuor

R Rabuor

I That is your rural home?

R Yes

I Your dad stays there?

R Yes, he stays there

I Okay, what about the services provided in the clinic, do you think they were good? Or you think some things need to be changes?

R They were good and helpful

I What about the staff at the clinic, how did they assist you?

R The staff treated me well, I did not see anything bad

I Okay, that is good, do you know about Stigma

R Stigma?

I Yeah

R Maybe if you remind me

I What about discrimination?

R Yes

I Like say is there a time when someone has refused to help you or even be close to you because of your status being positive, like someone looks down upon you and would not want to hang out with you or things of those sort?

R That was when I was in school

I In school?

R That is the reason which made me change school

I Which school were you in?

R I was in XXX

I Near your home

R Yes

I Did they have a day school program too?

R At that time it was both but nowadays it’s fully a boarding school

I What happened that made you leave?

R We had a school tournament, so I had a friend who we disagreed with and he was the one who knew my secret

I You told him or how did he know?

R He just knew since he was my friend and would access my locker box, so on that day we had a disagreement and he shouted all over things about me and sat in groups talking about me, so I did not feel good if someone came to me and asked about my situation, so I did not feel okay, so I had to tell my dad that I was not comfortable there and that he should transfer me to a day school to enable me learn or I drop off, I gave him those two options

I So where did you transfer to?

R He took me to some day school

I It was no longer XXX

R Yeah

I What about the other school, there was no discrimination?

R There was no discrimination because I did everything from home

I So they did not have that opportunity?

R Yeah

I Have you experienced cases where people don’t want to sit with you or share things with you because you were HIV positive?

R Those challenges were just in that former school, after that I did not see it.

I Did you ever tried telling the teachers

R I had to tell my dad to talk to the school principal and explain to him my status because I was afraid of telling him myself, so I had to ask my dad to tell him

I Did he tell him?

R So I started telling him about it even before people found out about my condition, I wanted him to inform the principal early enough so that he can even keep my medication in his office and I take it from there, so I started telling him about it earlier but he was just reluctant, so the only teacher who knew about it was the sports teacher, he’s the one who knew

I Did he ask you about it

R He did not take it serious because even if asked I used to said that I did not have the disease, so if he could have asked, my response would have been the same

I The same?

R Yeah

I What if the principal would have asked?

R I would only tell him the truth once my father told him

I Okay, can you tell me something about the ARVs that you are currently using

R Okay, like right now when I started using the single dose, I always felt dizzy but right now I’m used to it and they are not bitter, there are some drugs I started using at first when I started using the drugs, they were so big and you had to mix them with water before you drink

I So you used to crush them to powder form

R Yes and I also had a liquid medication

I That is when you were still young

R So that is when I realized that I’d stop using the drugs

I What about when you stopped using the syrup?

R I was given smaller tablets which looked like the one for headache

I Okay, and how were those ones?

R They were okay

I Do you have an idea of what ARVs do in the body?

R Like

I Like how do they help you when you use them?

R For me they help me since the ARVs have helped me, I heard in a certain song that if you use the drug you will live healthy so that really

I (Interjects) the song for those twins?

R Yeah, so that is when I started using my medication well and started going to the gym, so it helps me exercise well

I Okay, so you have said there are times when you even wanted to stop using your medication, did it reach a point when you stopped using your medication?

R That time, I was so mad at my dad, I did not take my medication, you know when you want to know why you are the only one using the medication, you have a lot of unanswered questions, so I had that challenge

I So you missed taking it or you were angry but still taking your medication?

R I was angry but at the same time I used to take my medication

I So you can’t say there is a time when you missed taking your medication?

R There was, I used to miss when I was in school

I So what made you miss?

R A times classes, sometimes I did not miss completely, I just missed with time

I So you take them but take later?

R Yes I took later

I Okay, I understand, so for you, you used to come for your medication in person, is there a time you faced any challenges in taking your drugs

R When I came on my own?

I Yes

R The challenge I saw was now that they have a youth center I see it is very easy than going to the clinic and you find older people the ages of your parents, maybe the others were neighbors, so it was a bit hard

I When you were on the adult’s section, was there a time when you missed a visit

R Yeah, I missed some appointments when I went to Nairobi

I Did you call them to notify them that you were going?

R Yes I called them, I even sent my dad

I To pick the medication for you

R Yes

I He sent it to where you were?

R Yeah, he sent it to me

I So you have mentioned that you missed an appointment, but your dad came and took the medication for you, is there an instance where both of you failed to come to the clinic?

R He can miss with one day

I He can miss with one day?

R Yeah

I Aahh, okay so like right now that you told me you take your medication from this side

R Yeah

I What feelings do you have about that?

R I always feel good, sometimes I even take time to have stories with the people around

I What do you think we can add to make the HIV care services to be better?

R I have never thought about that, but it will come

I It will come, if you remember just tell me okay?

R Okay

I You had told me that sometimes you missed taking drugs because of classes when you were in school right?

R Yes

I You used to come on your own to take your medication when you were in school?

R Yes I used to come on my own

I How were you managing in leaving the school?

R Maybe I feel like I have a headache and sometimes my father would call the school directly and I was released

I Was it hard or how was it?

R Sometimes it was hard because they came to get used to me leaving the school all the time

I You just leave

R I just leave, so mostly my dad had to call for me to be given permission

I Okay, when you used to leave for medication, can you tell me what else made you miss taking your medication while in school?

R When we had a lot of people in the dorms, that usually made me miss taking my drugs and the day will pass

I Even the evening dose

R I am talking of the evening dose, in the morning people are in class and other places so the one at night when we have a lot of people in the dorm it passes without me taking it

I What time did you use to sleep?

R At 10 pm

I 10 pm, so what time did your evening preps end

R at around 8 pm

I You leave your classes at that time?

R Yes we leave our class

I You leave for sleep?

R No we don’t go to sleep, we go to the school hall

I At night?

R Yes at night

I What do you go there to do?

R On that night they call all say form ones and they are taught one subject say like Biology collectively

I Okay, so its like night remedial classes?

R Yes

I So on sexual and reproductive health, have you ever heard of anything on sexual and reproductive health?

R Yes I have

I What have you heard kindly share with me

R *(Laughing)* Mmh

I What have you heard about it?

R Just ask the questions and I will answer you according to how you want me to answer

I the way I wat you to answer?

R Yeah

I Okay, where do you get information on sexual and reproductive health?

R Where I get it from?

I Yes, where you get it from

R I have heard of that information even here

I Even here?

R Yes

I What did you here exactly?

R Not that they were making stories

I They were teaching you or?

R Yes we were being taught

I What were you being taught about? I am, curious

R *(Laughing)* Aahh

I Can you remember what you were taught?

R I can’t remember well, you know at that time you attend the sessions, but you are not serious about it

I but just a small thing about what you were taught? Can you remember anything?

R Why a small thing, if I knew I would have told you everything

I So you don’t have even a small bit of information

R I don’t have

I okay, at that time when you were being taught, was anything to deal with family planning mentioned?

R Yeah they taught something on family planning

I And do you, yourself know anything about family planning?

R I know

I Where did you learn it from?

R I have known it as I grow

I As you grow?

R Yes

I You heard people talk about it or where do you get the information?

R I just found myself knowing the information

I Okay, what do you know about it?

R What I know, let me tell you, when you have like your cousins and they have reached adolescent stage, you hear their mother has taken them for family planning

I Those are the female?

R Yes

I Okay

R So I heard that it makes people become fat, such like things?

I Apart from making them fat, what else?

R It prevents unwanted pregnancies

I What about STIs, do you know anything about STIs?

R That name is familiar but

I *(Interjects)* So STIs are the sexually transmitted diseases or infections

R Okay

I You know those ones?

R Yes

I What do you know about them?

R Is it a must I answer even those questions?

I No, if you don’t want to answer that you just tell me and we will skip that question

R Then lets jump those questions

I Okay, so you have told me that you came to learn about sexual and reproductive health on your own?

R Yes

I And a bit of information from the hospital

R Yes

I In terms of access, can you get such services?

R Which services?

I Reproductive health services. Where can you get them from?

R I don’t know how I can tell you

I Okay, are they services that you have personally receive

R Personally?

I Yes

R Yeah

I Where did you receive the services from

R Just here

I Here in the hospital?

R Yes

I Okay and you said you got the information from hearing what people were talking about like your cousin being taken for family planning

R Yeah

I Where else would you get the information apart from the one about your cousins

R I can get it from the hospital and from the charts

I The charts mounted on the walls?

R Yes, the ones on the walls

I Okay, that is good, so where do you think is the ideal place where one can get the sexual and reproductive health services?

R For me, I am used to the facility

I What if there were no facilities?

R If there were no facilities, it would be hard

I It would be hard?

R Yeah

I So you prefer it to be in the facility?

R Yes

I Okay, so do these services help you or any other adolescents

R It can help me and even other adolescents

I I will ask you some questions and if you are not comfortable to answer it will still be okay

R Okay

I In what way do you think it helps them?

R It helps, you know people grow

I True

R You know you may have a girlfriend and such stuff

I Okay, and do you think the services meet your expectations? Or do you think there are areas which need improvement

R No

I and are there challenges that you as a person face when trying to get these services or information?

R Challenges?

I Yes

R I don’t see

I You have not come across any

R I have not

I All has been well?

R Yes

I Don’t worry we are proceeding on well and soon finishing up, so you said that when you were in school you had a problem in using your drugs and problems in leaving school to go get your medication, what about on sexual and reproductive health, were you experiencing challenges too on that area?

R To get?

I Those services while you were in school

R In school, some people used to come and teach us

I They came and taught you?

R Yes

I How many times in a term would they come in a term?

R Not even in a term, maybe when I was there I have just seen them for a year or so

I Okay, what about when you went to a day school, did they used to come?

R The day school was a mixed school, so I used to see them come

I You saw them?

R Yeah

I Can you recall where they came from?

R Where they came from facility, homes or?

I No like you remember when they come they introduce themselves like I am Irene from KEMRI or I am from FACES, where did they come from if you have an idea

R Some came from some hospital in rabuor

I In Rabuor?

R Yes

I That is FACES

R Yeah, there is another one too

I In Rabuor?

R Yeah

I Okay, right now would you be comfortable discussing issues to do with sexual and reproduction issues and be given a health care worker who is not a nurse or clinician but is old enough to be your mother or father?

R In fact I am always free with the elderly people

I You are free with them?

R Yes

I So if you were to be assigned someone you will be free having a female or male

R I prefer a woman

I You prefer a woman?

R Yeah

I Ow! If given the mother or woman, what kind of things would you discuss?

R Private things

I It is private?

R Yes, there are some things I would ask her about this disease, and if I have a girlfriend, for you to have sex with a negative person and you also want a kid

I True

R So what do you do if you want to get a baby in such a scenario

I Okay, what else would you have liked to ask?

R I can ask about PreP

I You have heard about PreP?

R Yes

I What did you hear about it

R I have only heard about it from people

I From people?

R In the past I used to hear about it but I did not know its use

I But later

R But now that I have a girlfriend, I fully understand it

I Okay, so for the lay healthcare worker, where would you feel comfortable discussing your issues?

R Just outside or somewhere like this

I In a room enclosed such as this one?

R Yes

I Why would you chose such a place

R You are sure that you are safe on the things you want to say

I Okay, we are almost winding up, let us discuss some of the interventions from the program which can help you and other adolescents, so lets say we want to give you this woman to help you with any information including matters sexual and reproductive health, how would you feel about that?

R It would be so great

I It would be great?

R Yes

I Why?

R I can’t say why but

I Can you have a reason?

R I think it is good because everyday there are always new things coming up

I Okay, that is good, I would also like you to describe the character of this lay health care worker that we will assign you

R Should be first understanding, not easily annoyed, be soft

I You don’t want a serious face?

R I don’t and she should be of less words

I So she should have an approachable look, talks less and what was the other? The characters that you would want from the person

R She should not be strict

I Talk less

R Yes, talk less and there is a word I cant explain, she should not be uneasy person who does not give me time

I She should be settled?

R Yes

I What other character would you like?

R Just someone who I can engage with other stories, not necessarily about these issues

I Someone you would engage in general?

R Yes

I Okay, so right now if you come for your ARV services and then we have the same services in the same room, how would you feel if both services were offered in the same room?

R To be in one place

I Yes

R I don’t see it being hard because both help me so it will be good if both are together

I What advantages would it have if placed together like that?

R If they are put together?

I Yes, what advantages would that have?

R If they are put and discussed in one room?

I Yes such that when you are picking your medication, you also receive say something like a condom and such issues and even you can seek matters to do with sexual and reproductive health, what advantage would that have?

R The advantage I would see there is that we have talked about everything and I understood all of it so it will be easy if it is thing to do with PreP I know how to deal with it or if it is to deal with condoms I will also know

I So would you prefer them put together or separately?

R I would prefer them being together it will be good

I That is true, what do you think would be the disadvantage of putting them together?

R There are some things you may not want to discuss like condoms, you may not want to discuss

I Okay. Do you have any questions you would like to ask before we close?

R About this?

I Yes

R I can just ask like do you work for the facility?

I Yes I work with KEMRI FACES

R So KEMRI is the one which has brought this up?

I Yes but we deal with adolescent officers

R So now that we have finished, is there chances where would meet again and talk about these issues.

I Chances are there, but you will find people dealing with adolescent in the facility to help you, you have people in the facility who help you right?

R Yes

I So if you have any questions they will assist you and they are there to assist you

R Okay

I Or you wanted me to be coming

R Yes, I have never been interviewed by someone like you

I Why?

R You are flexible

I That is good, in this life you have to be flexible

R You can work anywhere with those smiles

I Okay, lets close this discussion

R Okay

I Thank you very much for your time, we have now come to the end of our interview and the time now is 1323hrs. Thank you.

Name of study: AYA

Type of Interview: IDI 023

Date of interview: 16^TH^ JULY 2019

Start time: 1350hrs

Stop time: 1443hrs

Name of interviewer: Irene Okumu

Name of transcriber: Nelson Ouma

I My name is Irene Okumu, and this is the adolescent study, welcome to the interview and the date today is 16^th^ July 2019 and our interview will be conducted in both English and Swahili, the time now is 1350hrs, first of all I would like you to tell me a little bit about yourself, your age, education level, marital status and your occupation if you have any now

R My name is XXX, I am a 21-year-old Kenyan citizen, I just did my form four exams the other year

I When? Last year or?

R Sure

I Okay

R And at the moment I am a driver

I What is your level of education?

R Uhm

I After the form four, did you do any other thing?

R I just did driving the other day

I You did driving?

R Yeah

I So your current occupation is a driver?

R Yes

I So where do you drive? Or whom do you drive for?

R On calls

I Oh, you drive on calls?

R When Irene wants a driver, a long-distance driver, so she gets me

I Oh, I get it, that is nice

R Yeah

I So what are some of the challenges you face as a young person?

R Basically is a job issues, since I did my form four and now that I have a driving license I hoped that coming to KCH I might get a job

I Okay

R As an intern driver

I Okay

R Or even a, just as an intern

I Mmh okay

R So maybe that is my major issue

I So you want to pursue a career in driving or something else?

R I may take it as a mechanical driver

I Okay, I get you, alright any other challenge apart from job?

R Family issues

I What are some examples?

R Mmh the age gap between me and my elder sisters, you find that they take me as a kid and say I have nothing to tell them

I Yeah true

R I have my feelings and I have my stories to share

I What is the age gap?

R Uhm

I Okay, first of all how many children are you?

R We are six of us

I You are six?

R And it happens that I am the last born

I You are the last born, so what is the gap between you and the fifth born?

R Sorry…for year bracket

I Okay

R But for the ones its eight to fifteen years

I Yeah, because they are way older

R Yeah

I Okay, so how do you think these challenges affect your education or professional achievements

R I am always this type of person who takes it positively, despite the challenges, I take it as a step because out of the challenges is where I learn from

I Okay, that is nice. So, have you tried to deal with the challenges that you face?

R Like I told you about the family matters, I try being with them, like ask if we can go for lunch together, so the less that I have I use it to spend with them so that they also know that as much as I’m still a kid to them

I *(Interrupting)* yeah, you are trying to bridge that gap

R Yeah, I still need them in my life

I Yeah true

R you are my sisters and you are my brothers, I need you

I Okay

R Yeah

I So, do they support you in any way with your challenges or you are the one who has to reach out?

R I can say they are trying their level best

I Okay

R Because, for them to pay for my school fees until my school completion

I They are the ones who paid your fee?

R Sure

I Okay, that is nice

R Then being taken to driving school

I They also did that?

R Yes

I All of them together or specific ones?

R Mmh

I Or you don’t know, you just know that they paid to you

R I just know generally that they paid

I They paid for you?

R Yes

I So they pass through your dad?

R Funny enough I come from an orphan family, I don’t have both parents

I Ow! You are a total orphan?

R Sure

I Okay

R But a grown total orphan

I Okay

R Yeah

I So you just grew up with your sisters

R Sure

I And how many brothers do you have?

R There are two brothers, and four sisters

I Okay, so all along ever since you were young you just grew up with your sisters and your brothers?

R Sure

I Okay

R Not really but until 2016, we are from a large family

I Okay

R And the woman who we were left under to take care of us by bad luck left us in 2016

I What do you mean by left you, she died or just left?

R She died

I Ow she died

R Yes

I And your birth mother

R When we were still young

I When you were still young?

R Yes

I So these brothers and sisters that you have, they are all your biological siblings, or some are step siblings?

R These ones are real

I They are real ones

R Sure

I So what support is available to help you with these challenges that you face?

R kindly come up again

I the challenges that you have what kind of support do you have to help you overcome them

R the ones that is available is family itself, secondly my profession as a driver, a times if I actualize my role as a driver, it makes me feel appreciated, like if I am called to do a job, I feel like okay someone cares

I *(Interrupting)* someone knows you

R yeah

I someone cares about me

R yes

I nice, so on HIV care services, would you describe to me that day you enrolled in HIV care

R It was a challenge

I it was a challenge, what happened?

R okay, I can say that it happened on a road accident

I you had a road accident

R yes

I okay

R back in 2005, I was travelling to Eldoret and was, have you been to Eldoret?

I yes

R Which estates do you know in Eldoret?

I Uhm, I know, just a minute how do I put it, my brother used to live there

R where?

I those sides of polytecnic

R langas?

I langas, that is where my brother lived then there is this other one after langas, anyway there is langas, then barracks, the army barracks

R I used to stay in Huruma

I Okay

R so in huruma, I was rushing to take my assignments and there is where I was involved in an accident, it was, people tell me that it was the worst accident in the estate

I were you in a *matatu*?

R I was running then and hit by a moving vehicle and everyone knew that I was dead, so I was taken to the hospital where I stayed for three days, so I don’t know if it was during the rescue or at the hospital is where I received contaminated blood

I they performed a blood transfusion?

R yes

I do you have an idea of how many points you received?

R like around six

I around six points?

R yes

I okay, so after recovering, by then you were studying in Eldoret?

R yeah, primary school

I you were in primary

R yes

I so you were in primary by then?

R yes

I which class?

R I was young, class four

I around class four

R yes

I you were staying with your sisters?

R yeah

I okay

R my older sisters

I okay

R yeah

I so you were taken to the hospital mtrh

R yes, I was taken to the referral hospital

I and treated and given blood transfusion

R yes

I how did you realize that you are HIV positive?

R later after around four months, I started feeling tired and exhausted, you know apart from the normal exhaustion, these were maybe because of the accident but this exhaustion was not on the joints, a times I didn’t have appetite, sometimes I didn’t feel like going for long calls so from there I started feeling that I wasn’t that good

I there was something wrong, you could feel it?

R yeah

I so then what happened

R it was so hard for my sisters to tell me

I so they took you back to the hospital?

R so for me I was like let me take myself

I you were still in class four?

R it was on a third term towards class five and the church that we used to go had some nurses working at the uasin gishu district

I Ow UG

R yeah, so you know that instance where you talk just to free yourself

I Mmh

R so I told them, since I was involved in the accident, yes, I still feel the joint pains

I *(Interrupting)* but you still feel bad

R yeah, some sort of uneasiness, that feeling where you want to get out of that accident so that is when I was tested and they told me that do this, we will gave to involve your sisters, so after that, after a month I was given some medication and was told they would at least reduce the pain that I was feeling, so apart from using those drugs, there are other medication that I was given to take, so for the second batch I was told it would suppress the pain that I was feeling

I but did they tell you about your status?

R no, they didn’t tell me like immediately, it took steps for them to tell me, they just used to tell me, do you know the reason why we have given you this other medication, your blood is terminated and the only way we know is maybe when you got a blood transfusion, the nurse knows us well so she had to be free with me

I true, so she was the one who told you

R yes

I so on the day that you were being enrolled in to care, what was going through your mind, as a young boy, what was really going through your head?

R I was asking myself a lot of questions like why God had left me an orphan and to worsen it now I was on drugs, I was he trying to reveal in me? So in class five my performance went down so this nurse stepped in and talked to the school so that they understand my situation

I Okay

R	so towards the end of the year I started catching up in school

I	you started improving again?

R	yes

I	Okay

R	but that year, I always feel like what happened those years back just happened the other day

I	it is still fresh

R	Yeah

I	so when you were being enrolled into care, if I get you correctly you said that you were not told what the drugs were for, you were just told that it will reduce the pain?

R	yes

I	so when did you come to know that they are ARVs?

R	when I was given some activities to perform and I was at Ampath

I	Ampath is at referral

R	so its called Ampath and I was placed in a support group, so that is where

I	you ask yourself a couple of questions

R	and then when you hear what people talk about and ask yourself if its you they are studying or what, so after finishing the support group, I asked one of the support staff, how the thing is conducted because I feel you are talking about me so she told me I was in a safe place and that they were here to help me and children leaving with the virus

I	Mmh

R	out of curiosity I asked her how they knew I had the virus and they told me through link ups

I	so by then you didn’t know, or you already knew?

R	okay to my mind it was for the pain but when I was in Ampath I started understanding

I	*(Interjecting)* you started knowing

R	yes

I	so you asked them how they knew about it because in your head, you were not thinking about anything of that sort

R	even if my sisters knew, they just knew but they couldn’t tell anyone, so I knew maybe it was one of the ways they wanted to help me as their sibling

I	okay, what about engaging in care, what has enabled you continue engaging in care at this facility, because seems you transferred in, so when did you transfer in?

R	2011

I	you transferred in 2011?

R	yeah

I	so it has been a while

R	yeah

I	okay, so what makes it easy for you to come to this facility? Where do you live? What is the distance from home to the facility

R	first with the staff I must admit they are friendly, they handle you like you are part of them, they handle you as their guest that means that they handle you with respect, then the distance between kaloleni and here is a walking distance

I	okay

R	so when I’m called its just okay because I am around

I	when you came for enrollment, how comes you were not enrolled in Lumumba and yet Lumumba is closer than here?

R	by then, I stayed with my other sisters who stayed in, you know Oil Libya

I	the one back here?

R	yes, there is an estate behind there near the tourist market

I	at railways?

R	yeah, so we used to stay there

I	it’s called ardhwings

R	yes

I	ardhwings estate

R	so that distance was closer to here

I	yeah because if you take a *tuk tuk* you just highlight there

R	no one could notice that I came here too

I	yeah true, so since KCH is big if you get in to the hospital, no one knows where exactly you are going

R	yeah

I	true, I get it. Okay, what about the quality of services that you received?

R	they try to give their best

I	their best

R	yeah because be it on a one to one counselling, be it on support group, be it on drugs, they are doing their level best

I	and what about the friendliness of the staff at the clinic?

R	it is good

I	it is okay

R	very okay

I	Okay

R	very okay

I	alright, that is good to know, would you tell me of an instance where you ever experienced stigma or you were discriminated against because of your HIV status?

R	when I was in school

I	you were in a boarding school or was it a day school?

R	after knowing my situation I did not go to a boarding school so most of the times, I was even called to Nakuru High but since I knew about my issues, I did not go

I	Okay

R	so I was admitted in UG high but now

I	is UG High a boarding school?

R	there is a part where it is a boarding school and part of day

I	day schoolers

R	yes, so I was a day student, so most of the time I was with my friends who I was with back at home, so if it happens by mistake that at least referral guys come to school they would find that the first person they look for is xxx

I	you?

R	yes and then the vehicles were labeled Ampath, so my friends were always like, boss, how comes when these people come here they only look for you? And I told them that I was a champion, but later someone twisted it to say that I am a champion for HIV so I have to work with them if you are positive, but the fact has it, majority of the people there are positive, I saw that they were just hitting me with the reality, I can say it was not easy, more so you leave primary school and when you get to a high school you find that the stigma is indeed real

I	Its so real

R	yes and then there is always some form of freedom when you are I high school, you find that you are not treated the same way you were being treated in primary school

I	it is different

R	and then there is how they behave, it hit me such that I was even thinking of changing schools

I	what did they used to do, apart from saying that you were a HIV champion, what did they do so that it made you request for a transfer?

R	I used to be a scout and most of the time I used to be engaged with people in different activities such as camps, games within the county and you know it is required that when you go for such events you camp over there and people have to organize themselves, so you know when you remove your things without people noticing, so people ask if I will ever get well

I	okay

R	but if someone asks that and you don’t associate with them

I	so this is at the camp?

R	yes

I	so these are the students from other schools or from your school?

R	from my school

I	okay

R	even at home they wanted to monitor you

I	how you operate, so they were your friends but used to come at home just to check on whether you were going to use your medication or not

R	yes

I	okay, so tell me anything you know about ARVs, what do you know about ARVs?

R	one thing about ARVs, I wish they would make one which would be compatible with your system

I	the one you have is not compatible?

R	there is a time back in 2015 when it used to disturb me

I	okay

R	but I exchanged it with another one and now I am well

I	so the one you have now is the one pill a day one or you still take twice?

R	I still do

I	twice?

R	yeah twice

I	so what else do you know about ARVs apart from those side effects, what else?

R	it keeps me healthy, right now I am sure Irene that if I was not in this project you would have not known that I have HIV

I	yeah, you can’t tell, it is difficult, no one can know unless they say it

R	and then it’s all about acceptance, it is hard

I	I know, but you just have to try, but at least you are trying, it’s just a matter of trying. What about the time you missed taking your ARVs, is there a time when you missed taking your drugs?

R	It happened that I was invited for a certain job and I was in kakamega when they called so was confused if I should come back to Kisumu or I just go then come back, but my math did not add up well, so when I went I didn’t take my medication in the morning, and even in the evening, so I didn’t take three doses then if I am committed, I do a lot of drinks so I forgot that I was supposed to take food and fruits, do you know that I spent one week in hospital after that

I	Sorry, okay, so that was the only time you missed your medication?

R	yes

I	okay and kindly share with me challenges you face on keeping your clinic visits or appointments

R	I always find it convenient for me

I	okay

R	then in a weeks’ time I am always reminded of my visits then before two days I am reminded again through a phone call

I	so who sends messages to you

R	the counsellors

I	the counsellors here?

R	yeah

I	are they the same ones who call or someone else calls?

R	there is someone who always calls out

I	but from the facility?

R	sure

I	okay, tell about the last time you missed your clinic appointment, have you ever missed your clinic appointment?

R	for two days

I	what happened?

R	I wasn’t around, okay they reminded me up to the last minute then it happened that at that last minute is when I was not there

I	Okay

R	by the way, most of my family members do business and most of the time I like giving them company, some go as far as Nairobi and Kampala so to show that I am still part of them, I give them company

I	yeah true, so you go with them? When they are going to get their stuff, you go together?

R	yes

I	so that is the time you missed?

R	yes

I	so what are your general feelings about HIV care services that you currently receive?

R	I don’t know if it is because I have made a rapport with the facility staff but I find it, I can refer someone at KCH

I	you can refer someone, so it’s a good thing that will make you refer someone

R	yes

I	so what is it that they do that will make you refer someone?

R	their friendliness, their genuineness, they are ready to receive whatever anyone says

I	okay, that is nice, and how do you think your encounter with HIV care services can be made more satisfying

R	by reaching out to the schools and youths in churches and maybe enriching youths in these social places

I	so you want the facility to reach out to youths in those separate places that you have mentioned

R	yes

I	okay, so how will that make your services more satisfying?

R	because I know there are many youths in church who suffer from what I did go through and even in the schools, there are those who keep to themselves

I	I get you, I get your point now

R	it will be more satisfying

I	Okay, I get you, alright, so how does being in school for young peo[le like you affect their clinic appointments and taking their ARVs? Before you told me that you would go for camps and when you are in those camps someone would see you take your medication and ask if you will ever recover, so what other challenge did you ever have?

R	maybe opening to the teachers, because there is somewhere I read the other day that not all that laugh with you are your friends

I	that is true

R	even in school, not all teachers will love you, so I used to imagine for those who did not like me like four of them against me, imagine other students in my situation who passes through them and cannot open up to them

I	okay, so it was difficult for you to find a teacher who you would open up to?

R	Exactly

I	so did anyone know about your HIV status in school?

R	there is the senior teacher who knew, so I don’t know if he told other teachers, when one is punished say they send me to bask in the sun

I	when you are basking in the sun, what are the other people doing as a punishment?

R	someone is given a punishment like cleaning a hall, slashing

I	so you were given lighter punishment compared to them?

R	yes

I	okay, and what about in terms of keeping clinic appointments, being in school did it give you a challenge in keeping your appointments?

R	it wasn’t that much harder because we had a health club which was also an Ampath health club so you can imagine

I	yeah that was

R	that was much easier

I	yes

R	I can even say that it was not that

I	*(interjects)* difficult for you

R	yes because it was under red cross so we decided to merge with Ampath

I	so Ampath used to come into school for health clubs?

R	yes

I	and on sexual and reproductive health, what do you understand about sexual and reproductive health?

R	let me start with sexual health, these are the organs that separate a man from a woman

I	Mmh

R	then reproductive health is more of the sexuality

I	what about in terms of access to reproductive information? Do you get sexual and reproductive health information?

R	yes

I	where do you get them from? Or what do you understand by that information?

R	I think being taken at various stages of sexuality like transition from childhood to adolescent, then adolescent to adulthood, these are things that may happen or occur during this period

I	Okay

R	and I expect these at this and this time

I	alright, and in terms of safe sex?

R	that is VCT, that is where the use of condoms come in and at least knowing one’s status

I	yeah, true

R	before intercourse

I	so do you practice safe sex yourself?

R	sure

I	okay, so what about contraceptive use? apart from condoms, what about other contraceptive use?

R	in my age I just use condoms, the rest maybe later

I	the rest later, and your girlfriend?

R	thank God at least she admits

I	okay, what about STIs and prevention of STIs, what do you know about that?

R	wow!

I	*(laughing)* yes

R	sorry for this

I	it’s okay

R	last year there was someone who got STI and I forgot the troubles, he was the diss of the year

I	what happened?

R	Awasi is not far from muhoroni and chemelil, so most of the time we were taken to see how the sugar production works

I	from school?

R	yeah, so you know in our age we like experiencing sex, so I don’t know he went and had sex with an elderly girl and that is how he developed the STI he was feeling the pain and we could even see it

I	from his facial expression?

R	yeah, he was hurting

I	so do you know of ways of prevention?

R	one of the ways I know is safe sex, then if you know you have been transmitted, you go and seek medication immediately

I	okay, to you as an individual, how important is your access to sexual and reproductive health services?

R	I do practice them cautiously because I wouldn’t want someone’s daughter to start crying because of me, so I’m conscious with my girlfriend since I am open with her and the way I have accepted her is the same way she has accepted me, so that we live long

I	Does she know your status? Have you disclosed to her?

R	she does

I	so is she HIV positive or negative herself?

R	she is negative

I	she is negative?

R	yes

I	okay, so where do you currently access information on sexual and reproductive health?

R	sorry

I	where do you currently receive information on sexual and reproductive health?

R	through the support groups

I	where else apart from support groups?

R	maybe in church activities and social places like if they call youths, say we have been invited at the polytechnic or the museum, like the other day we had an adolescent open day

I	on Saturday?

R	yes, so we learn about things to do with STIs

I	that is good, so for you, where do you currently access your sexual and reproductive services, not information but now the services?

R	At KCH

I	KCH?

R	yes

I	Where else apart from KCH?

R	I once tried it out with LVCT, but I found it that most of their staff know me on a personal level, like they know my family

I	okay

R	so maybe in their mentality they thought I was loose but when it comes to your health it is not being loose, it is about you having the information and the actualization part of it

I	okay, alright so for you what is an ideal or the best place for you where you would like to access sexual and reproductive health services

R	if I was to be given that opportunity, referral

I	referral

R	I think I would say the Ampath part of it had a way of conducting their activities that you wouldn’t even feel stigmatized or discriminated in the community

I	so Ampath is still like a hospital, so I will take that as a hospital

R	sure

I	okay, so apart from hospital, where else would you like to access such services?

R	maybe in churches

I	in churches

R	I want somewhere where the pastor will open up with everyone like he should be courageous enough to tell me to do this and this and not the other things, it will be better for your own health.

I	nice, and to what extent are the sexual and reproductive health services offered to young people like you meet their needs and expectations?

R	I think they are doing their level best and they can use their communication to enrich the young adults

I	alright, so do you feel like it is meeting the needs and expectations of the young adults?

R	yes

I	okay, so what challenges do you as an individual face when trying to access sexual and reproductive health services?

R	just how I told you before, in LVCT someone might think one is loose

I	okay, so that is one thing you experienced?

R	yes

I	any other experience?

R	I was being asked that at my age why I wanted to know things to do with sexuality, and I used to tell them that I am growing, and it is something that I experience on a daily basis

I	okay, you told me you were in school, so being in school, or in college, but for your case now that you are in high school, how has that affected your access to sexual and reproductive health services, you told me that when you were in Uasin Gishu Ampath used to come and you moved from Uasin Gishu to another school right?

R	yes

I	when you moved to that school, did you also get access to those services

R	yeah, there is another health center in Awasi, it is a clinic under the catholic church, so the school gave us that chance for them to visit us and on Sundays we go visit them and have some conversation together

I	so how comfortable will you be discussing sexual and reproductive concerns with a lay female health care provider of your mothers age?

R	I will be as comfortable as giving out information and acquiring from her and then that age bracket is good because I will be able to talk to her just the same way a child talk to the parents so that they don’t see any barriers

I	and what if they were your peers, let say someone a little bit older than you?

R	maybe going to lunch together, then I open up to them

I	but you will be willing to share information with that person

R	sure

I	okay, alright so what issues will you be comfortable discussing with the lay female provider or the peer we are talking about?

R	how to access safe medication, and how to deal with acceptance, sometimes it is good to accept yourself, you find most of the time, like I was in Eldoret, a day happened there were three boys just of my age, they did not accept themselves and out of that you think someone has gone to have fun but they end up committing suicide just because of lack of acceptance

I	so these were your friends or people you know?

R	sure

I	so they committed suicide because they were HIV positive or other issues?

R	They were positive and they to some point they felt like they were disowned

I	I’m so sorry about that, so this health care worker, where would you like those discussions on sexual and reproductive health to take place?

R	maybe on a social place, somewhere like in church apart from hospital, if they come in the neighborhood

I	neighborhood?

R	yeah

I	okay, why would you chose such a place or those places that you have chosen?

R	like in church, as much as we are going there to study the word of God, He is God of Love

I	Yeah

R	and apart from love, you shall direct the love to me and as much as it is bad, you have to quote a bible verse

I	yeah true, I get you

R	if you quote a bible verse and tell me my mistake, I will be like okay

I	it is in the bible, I get it, so we are almost finishing our interview, so I would also like to get from you potential interventions that you would like to be incorporated in the services that you receive, how would you feel if we assign you a lay worker to be your confidant and your source of information for your sexual and reproductive health?

R	wow! It will be a privilege

I	why is that?

R	because can say the mistake which happens and once you accept and embrace your mistakes, the people around you will help you out

I	true

R	with your issues, I will be comfortable

I	alright, so this health care worker that we will assign to you, what characteristics would you want to see in them?

R	genuine

I	genuine

R	then if it is bad, it is bad, as it is be genuine to be himself or herself

I	any other character that you’d like to see in them, you are the one choosing the attribute such that if we decide that XXX we want to give you or assign to you someone, then we remember XXX said he wants a genuine person, what else?

R	someone who has confidence on themselves then someone who can connect with the youth

I	Okay

R	I know we are in a ahealth centre, but they should have brains

I	brains? *(Laughing)*

R	yeah

I	I get you, so what would you feel about receiving your ARVs together with your sexual and reproductive health

R	I will prefer than in my medications someone else to handle it completely and for sexuality to have a different person

I	so you don’t want them to be put together?

R	yes

I	okay, so it will still take me to the last question, what will be the disadvantages of receiving the ARVs and sexual and reproductive health services together, now that you have said that you’d want them separately

R	one, I think it will be too monotonous

I	alright

R	to them it would be like I told you about it

I	so you don’t want them going about telling you that you did not do this and this or the thing that I told you

R	yes

I	that is in taking medication

R	yes, then when it comes to sexuality

I	and however much you don’t want this put together, at the same point, do you think there will be other advantages for the other adolescents if not for you?

R	sure because to some point they will have some sort of confidence and privacy, you may handle me medically then Annabel handles my sexuality, so I will be much comfortable sharing with Annabel on sexual reproduction than being open to you, you will be strictly based on my medicine

I	what about if we have say Annabel to handle both, or if not Annabel any other clinician to handle both

R	I think I suggest two different people is that, you will be capable of checking on my medication in case my health goes down and advise me appropriately, that confidant

I	I get you, is there anything you want to tell me before we wind up this interview?

R	Just access adolescents living with HIV in churches, in schools and in the estates, youths really keep to themselves outside here

I	Thank you very much for the information that you have given us, we will try and use this to help the adolescents to strengthen their sexual and reproductive health services and their HIV care, we have now come to the end of our interview and the time now is 1443hrs, thank you

R	Thank you so much for your time

I	Thank you

Name of study: AYA STUDY

Type of Interview: IDI 013

Date of interview:

Start time: 1031hrs

Stop time: 1110hrs

Name of interviewer: Irene Okumu

Name of transcriber: Irene Okumu

I	Okay, welcome, my name is Irene Okumu and this is AYA study, today we are here to conduct an interview on challenges and different ways in which we can be able to help adolescents in order to access sexual and reproductive health and on possible interventions that can be incorporated within the health systems that can be able to assist the adolescents, and the time now is 1031hrs and this is our participant from Lumumba hospital. So, we will just go right away to our questions in case you do not understand anything you let me know I can rephrase it for you or say it in a way you can understand more, if you do have questions feel free to ask and I will be able to answer you

R	Okay

I	Alright, welcome, so our first set of questions will be challenges that you as an adolescent experience, but before we start on the challenges, I will first of all ask you to tell me about yourself, how old you are, your educational background, your marital status, your current occupation if you have a job that you are doing

R	I am called XXX I currently finished my form four last year, and I’m 19 years old, I am single.

I	Okay

R	Yeah and I currently don’t have a job

I	Okay. You are staying at home?

R	Yeah

I	Alright, so as an adolescent, what are some of the challenges that you face

R	There are various challenges

I	You know sometimes at this age you cannot explain everything to your parent

I	True

R	So, they might not understand so you keep them to yourself and they hurt

I	Alright, so these challenges that you face as a person, how do they affect your education?

R	Sometimes, let’s say you have various friends and you are planning to go somewhere, you know you need money when you want to go somewhere, so you want to borrow money from your parents but you don’t know how to explain it

I	Where you are going?

R	Yeah

I	So this place that you are going, where is this?

R	Maybe you are going to hang out somewhere

I	Okay, so it becomes a little bit difficult to ask for the money?

R	Yeah

I	Okay, but is there a way you deal with these challenges?

R	Yeah, sometimes I deal with them

I	in what way?

R	I call my sister and explain out

I	Oh, your sister is older or younger than you?

R	She is older than me

I	Okay, she is older than you

R	Yeah

I	So when you explain do you get this cash that you want to use?

R	She always send the money

I	Wow

R	Yeah

I	Okay, so, to what extent have you succeeded in dealing with these challenges that you face?

R	Not every time you want you will get

I	Alright

R	You know you can’t borrow money from someone everyday

I	True, so, apart from these financial challenges that you are citing, do you have other challenges?

R	Yeah, there are various challenges

I	Okay, like which one?

R	You know sometimes like in the clinic when you come to pick your drugs, maybe you need to be somewhere by 10am and maybe you came at 6am, and the doctors delay until you get out at 2pm and you had to sit for an exam, sometimes I find myself just going and coming back later

I	So, what you are saying you used to come from Chulaimbo to get your medication then go back to school?

R	Yeah

I	Was this a boarding school

R	Yeah

I	Okay, so

R	*(interrupts)* you know there, you can’t explain to all the teachers what you are going through

I	Yeah true, okay so, what support is available to help you deal with these challenges?

R	I used to tell my sister to go and pick drugs for me and bring them to school

I	Alright, so she did that all the time or sometimes?

R	All the time

R	After I had informed her

I	Okay, of the challenges that you were going through

R	Yeah

I	Okay, and what about the other challenges, how have you gotten support

R	The challenges…I can’t say much

I	You can’t say much? Let’s say for example you had said there are some things that could hurt you but you can’t discuss with your parents

R	You know you just can’t share; you share with friends; you know when you are with friends you share whatever you are feeling and they will understand you

I	So all you do you just share with your friends?

R	Yeah

I	But you don’t tell any other person

R	Yeah

I	Alright, so on HIV care services, I’d like us to describe to me your experience on the day you enrolled in to HIV care. Do you remember?

R	Yes

I	Okay, tell me something about that

R	I enrolled in HIV care when I was in class four by that time I did not know what these drugs were for, I came to learn about them when we came from some support groups and we were being taught so I realized that this drug was for this particular disease and I just adapted with it, I just took my drugs well

I	When was this? Do you remember?

R	When I was in class eight, 2013

I	That is when you realized what the medication was for?

R	Yeah

I	and when you were enrolled, do you remember when this was?

R	I was a small boy

I	And where was this?

R	It was in Lumumba hospital

I	And the decision for you to enroll in HIV care, were you part of the decision or how did that come about?

R	I was not part of it, you know I was very small, and my parents didn’t tell me what was happening I just agreed to what the doctors said

I	And do you remember what happened on the day you were taken to Lumumba?

R	I can’t remember

I	Okay, that is okay, and on that day that you were being enrolled you were a small boy, but I believe there are some things that were going through your mind, what was going through your mind?

R	I was asking myself that this drug that I am supposed to be taking every morning and every night, so I was wondering if I go to class five I thought I was not going to take it every day

I	Okay

R	So I started throwing away the drugs

I	Why were you throwing them?

R	Because I did not know why I was taking those drugs

I	Okay, and who accompanied you when you went to the clinic

R	It was my mum

I	Just your mum?

R	Yeah

I	And where was your dad at that time?

R	He was working in Uganda

I	Okay, but was it easy for you to get enrolled on that day?

R	It was not easy

I	Why is that?

R	Because we were being enrolled for the first time and there were various procedures that we had to pass through

I	And you came from school or you were at home?

R	I was in day school by then

I	Oh, you were in day school by then? So, were you able to go to school on that day?

R	I was not, in fact that day I was sick

I	So when you were taken to Lumumba you were taken to seek medication for other sickness or you were just taken to enrollment?

R	By that time I did not know, you know you are a small boy

I	All you know is that you were taken to the hospital

R	Yeah that we went for treatment

I	Its okay, so what encouraged you to continue engaging in care at the facility?

R	Yes, when we were going for the support groups, you find children like you there who open their hearts and told us what happened to them and why they keep on taking the drugs then I said to myself I cannot stop taking this drug. I had to keep on moving

I	So that made it easier for you?

R	Yeah

I	What about in terms of distance from where you live?

R	By then we did not live far from the hospital, we were living in Milimani

I	Okay, that is not far. What about the quality of services at the facility

R	Sometimes you can go there, and the doctors are fast sometimes you can go there and become bored because you came first, and you see someone who has just come being served

I	An adolescent or someone older?

R	An adolescent

I	So its like they jump line or?

R	Yeah and they also waste a lot of time

I	The adolescent or theclinician?

R	The clinicians

I	What do you mean by they waste a lot of time?

R	You know sometimes you can sit there at the waiting bay and see a pile of files and they are not even at the desk

I	Oh, I get you, and the staff in Lumumba, are they friendly people?

R	I can say that all of them are friendly

I	Apart from the wasting time part of it you can say they are friendly

R	Yeah

I	Okay, that is nice, so, could you tell me of an instance where you faced stigma, or you were discriminated against because of your HIV status

R	I have never experienced stigma since where I was learning was a boarding school and no one in that school knew my status even my close friend

I	They didn’t know?

R	Yeah

I	Was there any other student who was in medication that you may know of?

R	Yeah, I knew of a certain boy, but he did not know that I knew

I	Ooh, how did you know?

R	I was just seeing him, we were sleeping in the same cube and I was seeing the way he was, he always used to take his drugs in the morning and in the evening

I	What time did you take yours

R	I was taking mine at night and in the morning

I	You could see him taking his, but he could not see you taking yours?

R	*(laughing)* yeah

I	How? How many were you in that cube?

R	We were six

I	Okay, that is nice, you say you were able to see that one (laughing)

R	*(laughing)* yeah

I	So, it means no one has ever known about your status, is there anyone who could have known your status?

R	In school there was only one person, the bursar

I	How did he know about that?

R	My mother disclosed to him

I	Okay, how did you feel about that?

R	It was just good because he was a good teacher, he was those teachers who don’t make stories with their fellow teachers, very quiet

I	Alright, so was he of help to you?

R	Yes, I always told him when my drugs were over, when to call my mum to get me some drugs and he did that

I	Okay, that is good. So, tell me anything you know about ARVs

R	Okay ARVs are drugs that when you take them you reduce the amount of virus in your blood and you have low viral load and high number of CD4 cells

I	Good, and what else do you know about ARVs?

R	(Laughing) Mmh

I	You have told me to lower your viral load

R	Yes

I	Your viral load goes lower, your CD4 goes higher, another thing that you know about them?

R	You stay even healthy

I	Okay

R	You don’t get any opportunistic diseases

I	Nice, alright Okay, tell me about the time you missed taking your ARVs

R	In school there was a time, you know when you are in school, and you are out of pocket money and you are taking drugs, you can get very tired, hence I just missed taking the drugs

I	Okay, and what about the food in school?

R	Sometimes it is good, sometimes it is bad

I	So you don’t want to take food in school?

R	So you don’t eat, you know you can’t eat, and you take the drugs

I	Yeah, true, okay so, when you miss your medication in school, apart from not having your pocket money, what else could make you miss your medication

R	Sometimes let’s say you have gone for the music festival and it is not around Kisumu and you know at that time you are being hurried to get in the bus and it is 5am you can’t remember everything, so you just get in without the drugs

I	Okay

R	and you go and stay even a week out of school

I	Okay, and if possible, can you please share with me the challenges you face keeping your clinic visits or appointments

R	Challenges, you know there was some time that I was doing some computer studies, that time say you have an appointment at the clinic and you also have to be in class by that time, so you don’t know whether to go to the class or for the drug

I	Alright, so any other challenge apart from the one where your school time table clashes with your clinic visit

R	There is no other challenge

I	If you remember anything just let me know

R	Yeah

I	Okay, tell me about the last time you missed your clinic visit

R	The last time I missed was say in April

I	April this year?

R	Yes

I	What happened?

R	I was not around Kisumu, I was in Nairobi, but I went to the nearest clinic and got the drugs

I	So, you only missed your clinic and not your medication

R	Yeah

I	That is good, were you able to contact your clinic to let them know you were not coming?

R	Actually they called me before the appointment date

I	Oh, so you told them in advance that you will not be around

R	Yes

I	So, what are your general feeling about the HIV services that you currently receive?

R	Actually at Lumumba they give us the best care

I	Okay

I	What do you mean by the best care?

R	because they understand everyone

I	Alright, so when you say understand, in what way? What would make you feel or say that they understand?

R	You know when you know with support groups, the way they talk will make you know that they understand what we go through

I	Okay, so that brings the feeling that they do understand what you are going through?

R	Yes

I	How do you think your encounter with HIV services can be made more satisfying?

R	At the facility?

I	Yes at the facility or even outside

R	Yes, actually there was a time when FACES used to organize camps in various place, you know those things you can find an adolescent who does not know why they are taking the drugs

I	True

R	and you go there and find someone who knows why they are using the drug and they share their minds and this ones start to change and takes the drugs well

I	Maybe if I can take you back a bit, there is one time you said that for you what made you start using the medication again when you had stopped was that you saw some of the adolescents taking their medicine, was this a support group or camp?

R	It was a support group, then we went to a major camp in 2013

I	So when you went to that major camp, what was discussed during that major camp

R	They showed videos of other adolescents suffering from the same disease and you know when you are watching a movie, you get to feel what the actor is feeling

I	Yeah true, like we cry when we watch movies

R	Yeah

I	True, okay so you think those camps really help a lot

R	Yeah, the camps really help

I	That is good, and how does being in school or college, I know you told me before but maybe something else may come up, foe young people like you affect their clinic appointments and taking there ARVs?

R	Yes, in school, let say you are in boarding school and you have some friends who do not know or live your side, and if you are going to bath they are with you, if you want to eat they are with you so when you want to keep a few people and they don’t know your status, so you just have to miss your drugs because you cant go with them

I	Yeah true

I	Like these friends, are you in the same class or different class?

R	We are in the same class, and the same dormitory and they are your friends

I	*(Laughing)* Yeah

R	So you play the same game

I	So everywhere you?

R	Yes, we are together.

I	Okay, I get that, I am trying to picture how that one works, that is nice, then on sexual and reproductive health services, I would like to know what you understand by the term sexual and reproductive health

R	Mmh, sexual and reproductive health is if someone is sexually active

I	Mmh

R	Yes, I don’t understand much

I	Maybe if I break it a little down for you, so, access to reproductive information, where do you get this information from

R	Yes at the facility we were always taught everything, how to use protection, something like that

I	Alright, what about safe sex

R	We are also taught those ones

I	What are you taught?

R	We are taught that before having sex you have to use protection let’s say like the condoms, how to put them on, how to remove them safely, and how to dispose it off

I	Okay, and in contraceptive use, have you been taught anything or what information do you have on that?

R	Mmh, contraceptive yeah there are various ways of family planning

I	for example?

R	We are taught the one for let say for injecting in the arm, I don’t know the name (laughing)

I	Okay, it is okay, another one?

R	We also have the female condoms

I	Any other one

R	I can’t think of any other

I	If you remember you just let me know

R	Yeah

I	Okay, and what about in terms of STIs and prevention of STIs

R	We were taught about STIs, but I can’t remember of the preventions

I	of the preventions?

R	Yes

I	Okay which STIs have you been taught about?

R	We were taught of syphilis, gonorrhea, only those ones

I	Okay, if you remember any other you can tell me, and you said that you do not remember how they are prevented

R	Yes

I	So how important is your access to sexual and reproductive health services?

R	You know when you are taught about the sexual and reproductive health services, you get a way of preventing the STIs, you cannot easily contact them

I	Good, so you have gone back to that question where you were saying you don’t know how it is prevented, so how do we prevent STIs *(laughing)*

R	*(laughing)* How to prevent STIs?

I	Yes

R	By getting good sexual education

I	Okay, any other because I can now see the points are coming

R	Yeah, they are coming

I	So any other way?

R	and being neat, changing your clothing every day

I	Any other way?

R	I can’t think of any

I	You can’t think… don’t worry if you remember another one you will say

R	Yeah

I	Okay so where do you currently access information on sexual and reproductive health?

R	I access them from the facility, let’s say when we come for the support groups, there are various activities and we are taught different things

I	So what various activities are there?

R	Games, painting, drawing, playing and teaching other adolescents

I	When you say teaching other adolescents, what kind of things do you learn

R	the sexual and reproductive health and how to take your drugs, you are also reminded the names of the various drugs that you take

I	Okay, so like you remember the names of the medications off head

R	I remember the ones I used to take but I changed my drugs last month

I	Why was that?

R	because I had LDL viral load and people were changing their drugs to the one that is taken once

I	So you changed to that one?

R	Yeah

I	How do you feel about that one?

R	It is good

I	So you only take it once a day

R	Once a day

I	it Is easier, so what time do you take it?

R	I take it at 9

I	at 9 am or pm

R	pm at night

I	That is good, you see the advantages of viral suppression and adherence?

R	Yes

I	You get that, okay so what is the ideal place or location where you would like to get or access sexual and reproductive health services?

R	I just find here in the facilities as the best place

I	Why is that?

R	You know at the facility, the clinicians as I had told you, they know you better because I started going there when I was small

I	Alright. So, let’s say in a situation where we don’t have a facility, what other options would you have?

R	Let’s say I can go to the nearest clinic or youth center, yes

I	and if we don’t have a youth center?

R	just look for an older person

I	So a male or a female?

R	any

I	Just provided that they are elderly?

R	Yeah

I	Okay, that is nice, then to what extent are the sexual and reproductive health services offered to young people like you meet their needs and expectations?

R	You know when someone is growing, let’s say they are sexually active and want to try out anything

I	True

R	so, when you are taught before you can avoid it

I	Okay, so you feel that sexual and reproductive health information should be given before?

R	Yes

I	to adolescent about what age?

R	Say at 15 years

I	Okay, but do you feel that the sexual and reproductive health services that you receive there meets the needs and expectations?

R	Yeah, they meet our needs

I	in what way?

R	You know, sometimes they teach you something and you don’t know what this thing is, but you get to know and encounter it when the day goes by

I	Okay, for example what

R	We are taught, like before we used to see these condoms as jokes and they told us and started teaching us when I was in class eight, and when I was growing, I came to know what they were teaching us about

I	in terms of what you were being taught

R	Yes

I	So, what challenge in you as a person do you face when it comes to access of sexual and reproductive health information and services?

R	So now there is no challenge because when they want to teach they call us a week later

I	*(Interjects)* a week earlier

R	Yes, a week earlier, that there is a meeting on a certain day

I	So when you come for the meeting it is purely for the sexual and reproductive health issues?

R	Yes also how to use our drugs, and how to keep them safely

I	Okay, so do you remember anything like when was the last time you had the meeting?

R	this year I haven’t gone to any meeting

I	but you have been called?

R	Yes, I was not around

I	but if you remember the last one that you ever attended, what were you told on sexual and reproductive health?

R	sexual and reproductive health, we were taught on how to be neat at all times so that you can avoid infections

I	True, what else?

R	I cannot remember

I	It has been a while?

R	Yeah

I	It is okay, but if you remember you let me know

R	Yeah

I	Alright, so how does being in school, I know you have told me that you are done with high school and you were doing computer, but use that experience when you were in school at that time

R	Okay

I	How did be in school as a young person like you affect access to sexual and reproductive health?

R	In school I could not get access because in school you know you can’t go out anytime you want to, you just stay in school, you can’t tell the teacher that you want to go to the clinic, he or she will ask you many questions so you just have to stay in school

I	Alright, and was there a time that your school ever brought in information on sexual and reproductive health services?

R	Yes, they used to hire certain people to come and teach us

I	People from where?

R	There is a certain hospital Marie stopes

I	Oh Marie stopes, so they used to come to school, what were they telling you?

R	They just talk about how to use this protection, how to maintain yourself, how to avoid

I	Okay, and how often did they come?

R	They used to come once a month

I	So if you have like three months in a term

R	*(Interjects)* They come three times

I	Okay

R	*(Interjects)* sometimes they miss, they don’t come

I	Since you were in school for four years, you were in that school for four years?

R	Yes

I	So throughout your time do you remember how many times they came?

R	No I can’t remember

I	They started coming from form one or sometimes later?

R	No they started coming later

I	Okay, that is when you were already in that school?

R	Yeah, they started coming in form three

I	Okay, you as a person, how comfortable will you be discussing sexual and reproductive health issues with a lay female healthcare provider of your mother’s age or your peer? Someone a little older than you.

R	I will just be comfortable because you know they understand what people pass through, you know he or she is an elder and they know what they have passed through at various stages

I	Okay, so if you had a preference, would you prefer someone older or a peer?

R	a peer

I	You’d prefer a peer

R	Yeah

I	Why is that?

R	You know with a peer, he or she will talk to you like you are in a certain level

I	Okay, so if you were to chose between someone of your mother’s age or a peer you’d chose a peer?

R	Yes

I	Alright, so what issues will you be comfortable discussing with that person?

R	I will just say sexual and reproductive health

I	What in particular?

R	Let’s say the prevention methods, the drugs to take, let’s say someone has sexually interacted with someone who is HIV positive, there is a certain drug, let’s say the PrEP and something like that

I	What do you know about PrEP?

R	PrEP is the drug that let’s say that a girl with HIV and I’m a boy without HIV we meet and the one without the disease does not want to contract the disease, he or she takes the medication after encounter.

I	So where and when would you want those discussions on sexual and reproductive health to take place?

R	Let’s say in an open ground, outside under a tree

I	in what sort of place like in town we have trees, in the village we have trees

R	In town

I	Why would you prefer an open place?

R	So that you can let out your heart and discuss everything

I	Okay, so you feel more comfortable when you are in an open place than enclosed place

R	I am comfortable anywhere

I	but you still prefer under a tree?

R	*(laughing)*Yes

I	Alright, thank you for the information that you are giving us and we are almost winding up our interview

R	Yes

I	but before we do so I would want you to give me views on potential interventions that you’d like for us to incorporate in the healthcare system that will help you as an adolescent, so how would you feel if we assigned you a lay healthcare provider to be your confident and source of information for your sexual and reproductive health

R	It will be good, let say he/she is filling me with the information

I	Why would that be good?

R	At the facility because I can reach there very fast

I	Okay, so where would you have the lay healthcare worker assigned to you?

R	I don’t understand

I	Let as say you have been assigned a healthcare provider to be giving you information and you told me that would be a good thing, so I’m asking why would you think it would be a good thing?

R	You know if he/she is assigned when it gets to a certain date they will feel like it is time to give me information

I	Alright, and what would be your ideal attributes or characteristics of such lay healthcare worker?

R	Let’s say a peer

I	So you would like a peer?

R	Yes

I	Okay, about what age

R	something like 25 years

I	Okay, what other characteristics would you like?

R	Friendly, he can also be…I don’t know *(laughing)*

I	Okay let us, maybe we can have a recap, let’s say we have assigned you someone right?

R	Yes

I	Maybe male or female, and they are talking to you about sexual and reproductive health services, so picture that person. What would you like to see in that person?

R	Let’s say 25 years, friendly and also understanding, only those ones

I	Okay, if you remember something just tell me before we close so that I can capture it

R	Okay

I	and what would be your, what would you feel about receiving both your ARVs and sexual and reproductive health together?

R	Together like let’s say when I come for…

I	*(interrupts)* yes

R	I’m also given information

I	Like when you get in a room, the same room where they attend to you they also give you services on sexual and reproductive health

R	It would be good but let’s say you should make it that when someone is waiting at the waiting bay, he/she is given the sexual and reproductive health education

I	at the waiting bay?

R	Yeah so that you can engage your brain, so that you don’t see that you are wasting a lot of time

I	Okay, so you prefer to be given the information first before you get into the room?

R	Yeah

I	So when you get in to the room and the room is a one stop shop how would you feel about that?

R	It is also good

I	What makes it good?

R	because you know you have to think about both of them and weigh them

I	When you say think about them, think in what way or think about what?

R	You know when you are taught something, you think about the good and the bad side and now you think of the good side, yeah

I	So what would be a good side for you?

R	You practice the education

I	Okay, so as we come to the end I would like to ask what are the advantages and disadvantages of receiving your ARVs and sexual and reproductive health services at the same place or point?

R	It will be good because when put together, you will have to schedule a fixed date and come for the health education and ARVs because if they are separate you will have to come on separate dates

I	So that is an advantage

R	Yeah

I	Any other advantage?

R	Only that

I	You can’t think of another one, alright, and what about disadvantages

R	Actually I cannot think of disadvantages, yet it is not yet tried out

I	Okay, so let us say that we want to do that experiment

R	but I thought that you know when you put it at the clinician is telling you both of them and you are waiting at the waiting bay just seated let’s say you should take someone and teach the people at the waiting bay as the clinician is waiting to attend to them

I	Okay

R	So that you can engage the brains of the people at the waiting bay

I	I’m getting it, correct me if I’m wrong, you are saying that while you are at the waiting bay someone is there giving talks on sexual and reproductive health?

R	Yeah

I	So when you get into the clinician room, you get the ARVs

R	Yeah

I	Okay, so in sexual and reproductive health, there are services that you need let’s say now they are not information but now when you get into the room now your ARVs and services

R	Let’s just say at the waiting bay is just information and then when you get to the clinician is the services

I	Yeah, now I get it, so that one is an advantage. Okay, what about a disadvantage

R	I can’t think of any

I	What you can see is only the advantages, alright, thank you very much XXX for your time, it has been an interactive session

R	Yes

I	and the information that you have given to us, we will be able to use it to strengthen sexual and reproductive health services for adolescent and young youths. Thank you so much for your time. We have now come to the end of our interview and it is now 1110hrs, thank you.

Name of study:	AYA STUDY

Type of Interview:	IDI

Date of interview:	14-July-2019

Start time:	2:30 pm

Stop time:	3:05 pm

Location:	KCH Adolescent Centre

Name of interviewer:	Irene Okumu

Name of transcriber:	Irene Okumu

I	Welcome for the interview today; my name is Irene Okumu and this is AYA study dealing with adolescents and the challenges that adolescents experience and therefore I would like to ask you a few questions. In case you do not understand anything kindly let me know so that I can be able to repeat or to explain it well. Feel free and if you have a question just ask and I will answer. The time now is 2:30 pm and we begin our interview.

I would like to ask you challenges that you experience as an adolescent but first all could you tell me something about yourself in relation to age, education, your marital status and also if you have a job that you are doing.

R	I don't have any job and I am still single; I am a student only that.

I	You are in school and how old are you now?

R	I am 19 years

I	You are 19, and where do you go to school?

R	RIAT (Ramogi Institute of Advanced Technology)

I	Alright, so you as an adolescent which challenge do you experience as a young person?

R	There is none.

I	You don't have any challenge?

R	No

I	And what about any problems that you may be experiencing?

R	There is none

I	None, so you are fine in all aspects?

R	Yes, am fine

I	And what about challenges in relation to drug adherence or keeping clinic appointments?

R	Yes, sometimes when I meet someone that I know, I go into hiding until they leave that is when I can come in and be attended to.

I	That is also a challenge

R	{Laughs}

I	Another challenge?

R	Only that one

I	That's the only one that you have

R	Yes.

I	And what about in relation to drug adherence?

R	Yes. When I was in secondary school, I stopped taking my pills. I would take them and throw them into the toilet, that way.

I	Why did you decide to do that?

R	Because I had a friend who found me taking medication, she started asking me XXX, what are the pills for? I went to the bathroom and when I came back, she had opened up my bag and they had removed the medication. That is what made me stop my medication. Then Meg and the team advised me, that is when I started adhering well.

I	Alright, how do these challenges affect you in terms of your education?

R	For now it does not bother me, I am doing well

I	You are now doing well, it seems like you are doing well, what about in terms of income?

R	None

I	All these that you have encountered, how have you been able to deal with these challenges?

R	At times when I don't have money, I borrow from Safaricom and carry on with a small business on Saturday within the college compound because we don't go to class. I sell chips and that is what helps me.

I	What else do you sell?

R	Other sorts or friend potatoes

I	And your college is it a hostel set up or you go back home?

R	No, I don't go back home.

I	Do you live within the college premises?

R	Yes

I	And are you allowed to transact business within the college premises?

R	I live outside the school premises, if you live outside you are allowed to do business.

I	Therefore there are ways that you do business?

R	Yes

I	With this business, what challenges have you experienced as an adolescent living with the HIV virus?

R	It is okay with me because no one is aware that I live with the HIV virus.

I	And what about the fact that you are still a young adult?

R	That is what bothers me most. I see how beautiful girls dress up and leave while I am attending to my business. I usually console myself that God is aware and let me keep looking for money. That is mainly because my parents do not have the ability to support me at all times and I cannot ask from them always the reason I started the idea

I	Who again?

R	My parents

I	Are they the ones who suggested?

R	No, they lack the ability to support me so I just have to work.

I	But do you get harassed?

R	No

I	Are your parents alive?

R	Yes

I	Okay. I would also like to ask you that of the challenges that you have experienced, to what extent have you been able to deal with them?

R	In no way

I	Have you tried to deal with your challenges in any particular way?

R	Not yet

I	And let's say this, you told me that when you see someone you recognize at the clinic you hide. How have you been dealing with that challenge?

R	For now, I am not afraid anymore. I just come, pick my medication and leave. I just go back to school.

I	And what about if someone sees you taking the medication, how do you deal with that challenge?

R	The other day, there is a lady who saw me XXX and she asked me what I had and she saw them in my bag. She asked me if I also take these things and I asked her what her problem was. I also told her that this was my life, she just laughed and left.

I	Is she your friend or you go to the same college?

R	No, she is a friend from home

I	The place where live by the college?

R	Yes

I	Is there any support that you have received to help you deal with these challenges that you experience?

R	I shared with Meg {Adolescent peer lead} and they assisted me.

I	In what was did they assist you?

R	Sometimes they send me some little money

I	The money that they send to you, is it their own money or where do they get the money from?

R	That, I have no idea

I	Okay, you have not asked but you know that when you ask for support, they assist you.

R	Yes, and they assist me

I	They all assist you or a specific individual?

R	Mostly Meg is the one who assists me

I	Okay, Meg. And what about your medication, I would like to ask if you could kindly tell me how you felt on the day you were enrolled into HIV care.

R	I started care when I was very young, I didn't know about anything. When I got to class six, that is when I inquired from my mother what the medication was treating my mother told me while crying and that is when I knew.

I	And at the time when who had taken you to the clinic?

R	My mother

I	So when your mother brought you, is that when you found out or how did you find out that you had the HIV virus?

R	I was not aware of anything because I was still very young. I knew when I was in class six that is when I knew that I had the HIV virus.

I	Where were you on that day when she was disclosing to you these facts?

R	We were in the house

I	You were in the house

R	On that day, she called me at 7:00 pm to take my medication and I told her that I was tired of taking the medication, what is this medication for? That is when she told me.

I	After knowing that you were HIV positive how long

R	{Interjects} I had a lot of stress and I used to cry in class just that

I	And who followed up on you?

R	My mother did

I	What made it easy for you to

R	{Interjects} To come to the clinic alone?

I	No, first of to take your medication

R	My mother told me that if you don't take the pills, you will die and that is your life that is when I started taking my pills.

I	At the time when you were taking your medication, and picking your medication from this facility, what made it easy for you to continue engaging in care at this facility?

R	The healthcare providers are so good and they give you advise

I	What else do they give?

R	Meg and the team are confidential and when you confide in them, they don't talk about it and they also love people, just that.

I	And in terms of the services that you receive at this facility?

R	It is good

I	In what way is it good?

R	It is just good {Laughs}

I	Kindly explain a little bit on the part that you say is good

R	It's because they love children and when we come, we are well taken care of. Sometimes we are called to go and attend camps and that is the reason why we love this place.

I	And what about in terms of distance to the clinic, how far is it?

R	Nothing

I	Is it far or near?

R	It is close to this place, because public transport is only Ksh. 50 to this place.

I	And during the time when you close for holidays, do you go back home or you stay where you are?

R	Yes, I go back home.

I	Where is your home?

R	In Nyawita

I	Your home is in Nyawita and it seems Nyawita is closer than where you live by the school

R	Yes, Nyawita is closer than college because you can even walk to this place

I	Oh, you can walk. And what about the health care providers, are they friendly?

R	They are very friendly

I	To what extent are they friendly?

R	A lot of it {Laughs}

I	A lot, kindly tell me a little bit more

R	They are loving, just that

I	You see them to be loving and that is fine

R	Yes

I	And could you tell me of an example where you have been discriminated against because of your HIV status?

R	Yes, when i go to the camp i get selected to teach people and sometimes I teach them how to dance and I have been told that when we close for holiday I should come and teach people dancing because am in the “sakata” dance group

I	And what about if there is a way in which people have discriminated against you or made you feel stigmatized because of your HIV status?

R	I am no longer afraid and I am free. I tell them that it's okay.

I	What about before?

R	I was afraid before and I would cry if someone discriminated against me

I	And what about if someone tells you anything now?

R	I don't keep it in my mind

I	Does it bother you?

R	Not at all

I	And what about deep down in your heart?

R	Nothing at all

I	Have you experienced where people don't want to be around you because of your HIV status?

R	Yes, that happened a lot especially those we were with in high school

I	Okay, where did you attend your high school?

R	Ahero Girls

I	Ooh were you in boarding?

R	Yes

I	Did the teachers support you in any way?

R	Yes, the teachers supported me. When these girls started insulting me, the teacher now was storing my medication for me so I would go and pick my medication but throw it away and I would pretend as if I had swallowed the pill then I would throw it away

I	Later, were you able to overcome the challenge?

R	Yes

I	I would also like to ask you now that you have been on HIV care, what do you know about these drugs?

R	These drugs help us so that the virus can sleep so that we can have zero virus.

I	Okay, any other thing?

R	That's all that I know

I	You only know that it makes the virus sleep?

R	Yes, very much

I	And you don't know of any other thing?

R	No

I	Okay, and about the time that you missed to take your medication, what happened that made you miss your medication?

R	I was sick and I stopped taking the medication. I was hospitalized here, Meg and the team started counselling me and that is when I started taking my medication well.

I	And what made you stop taking your medication?

R	Because people were talking

I	What were they saying?

R	That she is on care, she has been promiscuous and has been infected and that is what really hurt me. But now I don't care, whatever they say about me I still take my medication because I tell myself that it is my life and I also tell them that they have not been tested and that they don't know their HIV status.

I	And, is there a time that you have missed your clinic appointment?

R	Yes

I	What happened that you missed your appointment?

R	I was told to come for medication by my mother but then after people had told me that, I was afraid and I would not get to the facility. I would get to town and just walk around then go back to the house.

I	When you go back to the house, did she ask you?

R	Yes, she asked but I told her that the facility was closed. Then she sends me to go back the following day and I would tell her that they are on off duty. {Laughs}

I	For how long did you keep up with this?

R	For two weeks {laughs}

I	And at that time, did you have any medication?

R	I did not have any medication

I	So how did you manage to come back on care?

R	Mega and the team traced me at home

I	What did they tell you when they found you?

R	They asked me my reason for not coming to the clinic so I explained and they counselled me and told me that those are things that happen and that I should go and take my medication so I came back with them and took my medication.

I	Alright. At the time when you come to the clinic, what are your feelings on the services that we offer in the clinic?

R	They are good

I	Good in what way?

R	They are just good {Laughs}

I	You know degree of goodness can be because of something specific that you receive, or san action that someone does, what brings the goodness?

R	The way they work well, if you come, they are fast and they don't keep you waiting

I	Any other apart from being fast and good, what else do they do that makes you have a good feeling?

R	They have pieces of advice that they give us on how to adhere to your medication, they laugh with you and play with you. Only those.

I	Do you think your HIV care services can be made more satisfying?

R	That one I don't know

I	Okay, let's say you come to the clinic, is there anything that you feel we should do as healthcare providers to make you have satisfying services?

R	Advise, if they can give us advise on how to adhere to medication, they sit you down and talk to you not talking while in a hurry.

I	Any other thing that you would like?

R	Only that

I	That's the only one. is there anything that you feel we are not doing well so that we can omit?

R	Nothing

I	We just leave it the way it is

R	Yes {Laughs}

I	There before, you told me that you were in a boarding school, and now you are in college, how does that affect keeping your clinic appointment?

R	{Mumbles}

I	When you are in college and you are scheduled to visit the clinic, is there something that prevents you from attending your clinic appointment on the scheduled date?

R	Nothing

I	And your peers, do you see them facing challenges that may prevent them from keeping their clinic appointment?

R	Others are afraid

I	What are they exactly afraid of?

R	They are afraid that people may laugh at them

I	Just laughter?

R	Yes

I	Nothing else?

R	That people will talk {Laughs}

I	What will they be talking about?

R	That they are going to pick their drugs

I	Just talking about medication or there is something else that they talk about?

R	That's all they talk about.

I	I would also like to ask you, as I had earlier on told you that I whatever we are researching on is on sexual and reproductive health of adolescents like you. What do you understand by the term sexual and reproductive health?

R	{Silent}

I	You as an individual, let us say that on how you receive information on reproductive health, safe sex, contraception or on STIs. What is it that you understand?

R	The one for reproductive health.

I	Tell me a bit on what you understand on reproductive health?

R	Kindly let us pass that question {Giggles}

I	Do you find it difficult to answer?

R	Yes

I	What about if I explain in a way that you can understand more?

R	Yes, repeat the question.

I	So we have reproductive health, right?

R	Yes

I	What does it mean?

R	Child birth

I	And in reproductive health, there are other things that happen right?

R	Let us say someone can have a method of family planning, do you understand anything on family planning?

R	I know that family planning prevents one from getting pregnant

I	Now, do you know methods of family planning?

R	No

I	And have you heard someone else talking about family planning?

R	Yes

I	What did you hear them talk about on family planning?

R	That family planning is bad and that it prevents you from having children and that it destroys the ovaries.

I	And when you come to the clinic do you get information on family planning?

R	No, not yet

I	Of what importance is your access to sexual and reproductive health services?

Let us say that you as an individual, on matters relating to sexual and reproductive health, what is it that you take to be important?

R	There is nothing

I	You feel nothing is important?

R	Yes

I	Why do you feel that way?

R	Just like that {Laughs}

I	Okay, and where do get information on sexual and reproductive health?

R	From my parents

I	What do they tell you?

R	They tell me that I should not give birth at this time that I should look back at how the house is and that I should study first.

I	What do they mean when they tell you to look at how the house is?

R	They mean how they suffer.

I	Does your father go to work?

R	No, he just sits

I	He sits, and your mother?

R	She sells chips by the road side and sometimes she does not sell.

I	And how do they survive?

R	Just the chips business.

I	And your father, is he sick or not?

R	He lost his job when how went for his grandmother's funeral then he got fired.

I	So sorry. And where do you currently receive information on sexual and reproductive health? All the information, you said you get from your parents, is there any other place you get them from?

R	No there isn't.

I	So only from your parents

R	Yes

I	And what about at the clinic?

R	That one we have not been told anything

I	Okay, you have not been told anything

I	{Noise in the background} And where do you feel comfortable receiving sexual and reproductive health services?

R	At the hospital

I	Why the hospital?

R	Because they have the knowledge and they know everything so they should be the ones to tell us everything

I	It's them to tell you everything

R	Yes

I	Apart from the hospital, where else would want to get these services?

R	Our grandmothers who are old can also tell us

I	So you have said at the clinic and from elderly people, any other?

R	Only those ones

I	Alright. You as a student or an adolescent who is still growing, what challenges do you face in trying to access sexual and reproductive health services or even the information?

R	I find information even on the road when people are talking, like me people are talking so much because I have added weight and people are saying that I am pregnant, so I hear from other people and from my mother so my mother would call to ask me that she has heard people saying this and that. Someone saw you and thy are saying that you are pregnant so I tell her that I don't know and they should know because they are the ones talking.

I	How does being a student in college affect your access to sexual and reproductive health?

R	I am not yet prepared for that

I	You are not prepared. And what about when you are in school and there are services that you would want to access but you are in school, what challenges do you face?

R	I will bring him here so that he can be tested first then I tell him m status because I cannot infect him because maybe he is not infected.

I	What about in terms of questions relating to sexual and reproductive health, when you are in college what challenges do you face when trying to access information that can help you?

R	There is none

I	By the way, are you educated on these matters in college?

R	No

I	You are not taught and what about your friends, do you get to discuss about these matters?

R	No, I don't have time with them because when I leave class I go and attend to my duties then I sit back in the house.

I	Alright. Would you feel comfortable discussing your sexual and reproductive health concerns with a lay female health care worker who is of your mother’s age or of a closer to your age?

R	An older female

I	What would be your reason for preferring an older female?

R	Because she is the one that I can confide in. She would understand more than a father. A father will not understand anything.

I	What about if you are assigned a different woman not your mother whom you can talk to about your concerns?

R	I can

I	And what if you are assigned someone else who is not your age but a little older than you?

R	Not someone a little older than me let them be much older.

I	Why the difference?

R	Because I see them as my agemates so there is nothing that they can tell me

I	Where and why would you like these matters relating to sexual and reproductive health to be discussed?

R	In the house, sometimes at the hospital only those.

I	When you say in the house, whose house do you mean?

R	The elderly woman's house

I	What about in the hospital?

R	In an office

I	In an office. Alright. Do you have a preferred time that you would like tfor these discussions to take place?

R	Yes

I	About what time?

R	Let it be at about 2:00 pm that is when I can be able to discuss with her.

I	So the one in the house, would you prefer 2:00pm?

R	Yes

I	What about the one at the hospital?

R	I can call and let them know that I am on my way so that we can have a discussion

I	Now these people, people despite the fact that you are to meet them face to face it seems you would be willing to even talk to them on the phone?

R	Yes

I	Which is comfortable for you, talking on the phone or talking face to face?

R	Talking face to face

I	{Noise in the background} Face to face. I would also like to seek your opinion on the services that you receive and what we can add as a research or what you feel should be done away with from the clinic. What would be your feeling if we assigned you a lay healthcare worker to be your confidant and your source of information for your sexual and reproductive health?

R	{Silent}

I	This older woman that we talked about, let us say that we have decided XXX we want to assign you XXX to be the one to give you these information, to be your confidant when you have concerns, how would you feel if we assigned you such a woman?

R	I would feel good.

I	What would make you feel good about it?

R	Because I will not be hiding things that are hurting me, if I have an issue, I can call her and tell her that that on a certain day I will be free and that I can go so that we talk. Yes, just like that.

I	Any other thing that you would like to add onto that?

R	That's the only thing.

I	And, for such a lay health care provider, what characteristics would you like her to have? What type of behavior would you like her to have that will make you comfortable and be able to share with her easily?

R	Let her not be one who comes back and tells the hospital staff on what you have discussed with her. That is not a good person, one that keeps secrets.

I	What about if she does not know healthcare providers from the clinic?

R	That would be fine.

I	Therefore you want a person who is confidential?

R	Yes

I	What else would you like her to have apart from being confidential?

R	Only that.

I	You want her to be confidential only?

R	Yes

I	And any other character where if you discuss with somebody and you would say I don't like someone who speaks in a high tone, I would like a more polite person. What characteristics would you like for this person to have?

R	Be polite and does not say just anything. Be extremely polite.

I	So only the two?

R 	Yes

I	And how would you feel about receiving your ARVs together with your sexual and reproductive health services?

R	When you come?

I	When you come to the clinic, let's say you enter a room like this one where we are at this time and I am the only attending to you as a healthcare provider. I have your ARVs in here and I said that we also want to provide sexual and reproductive health services in here. How would you feel if it was put together?

R	That is not a good idea.

I	Why is it not a good idea?

R	Because everything is in there plus the medication that you will be taking are also there. It's not a good idea, I just feel that it is not right.

I	You feel that it is not right?

R	Yes

I	How would you prefer for it to be arranged?

R	To be put separately

I	When you say different, what do you mean?

R	Let medication have its own place and other services to be allocated their own rooms.

I	You have said that you do not like the services in one room, if I may ask let us say that we have decided to combine the services what benefits would that have?

R	None.

I	No benefits

R	No

I	Even a little

R	There is none

I	Even if you think a little bit?

R	There isn't

I	What disadvantages do you see?

R	Nothing

I	Okay, you said that when it is put together it's not good, you feel that it is not right. Now that you feel it should not be put together, why do you feel that it should not be put together? That thing that is in your mind that makes you say no, this is not right. What is it that makes you feel that way?

R	Nothing

I	It's just a thought?

R	Yes

I	We are almost winding up and thank you for the responses that you have given. All that we discuss will be used to strengthen health services. Thank you so much for your time and the time now is 3:05 pm.

Name of study: AYA STUDY

Type of Interview: IDI 017

Date of interview:

Start time: 1520hrs

Stop time: 1602hrs

Location: UNIM

Name of interviewer: Irene Okumu

Name of transcriber: Irene Okumu

I	Okay, thank you very much for accepting to be part of the interview, my name is Irene Okumu and this is AYA study, I would like to ask you a few questions concerning adolescents and young adults on the challenges that they face on accessing sexual and reproductive health services and in case you do not understand anything during the session, kindly let me know and if you have any questions in between, kindly just stop me and ask

R	Okay

I	And I will be able to answer your questions

R	Okay

I	The time now is 1520hrs and we begin our interview, so first of all I would like to know something a little bit about yourself

R	Okay, my name is XXX, I am a form four leaver yet to join college

I	Okay

R	I’m a daughter, sister, I don’t have much to say.

I	You don’t have much to say, how old are you?

R	I’m 19 years

I	What about your marital status?

R	Mmh, not married

I	Alright, do you have any small job you are doing now that you are out of school?

R	No, but small courses, sign language, music

I	Oh, you like music?

R	Yeah

I	That is nice, so as a young person what are the challenges you face?

R	Challenges in terms of?

I	In general

R	*(Interrupting)* oh in general, uhm my challenges are mainly, since I finished form four, I didn’t expect the world to be this different like I was just not prepared for this

I	You want to go back to school?

R	Okay, somehow, I want to be with the people I was with in the school, the people like, the outside world is cruel, that is what I can say

I	Exactly what has it done to you?

R	You can’t trust anyone, you are on your own apart from your parents, you can’t really trust anyone who really wants to benefit on their own

I	Okay, so these challenges that you face, how do they affect your education, or how do they affect your education?

R	Okay fine there is peer pressure, the main challenge that affect my education is that many people are like education nowadays you don’t go far with it, like there are no jobs, so they are like let us do these other things that can help us make money quicker and education can wait, that is what people say.

I	What are these other things that can help make money faster rather than being in school

R	The things that my friends say are maybe like music, when it comes to music you sing, but where they want me to go and sing, I just can’t go like in the club at night and I can’t do that

I	What type of music do you do?

R	I do all sort of music, I do not have a specific genre, I just sing all types of songs

I	And where is your ideal place to do your music?

R	Maybe in events like weddings, birthdays but not just clubs, at night, no

I	Okay, so have you tried dealing with these challenges?

R	Okay fine, I tried to deal with it but it was impossible so what I did was just to drop singing, not drop but postpone until I am financially stable so that I do music with my money because there are many challenges when you get to the studios, if you have your friends like someone is ready to help you but at the end this person wants something in return and you are not able to deliver so

I	What do they usually ask for in return?

R	Some of them want sex, some of them want you to be in a relationship with them and maybe you are not ready

I	Does that cut across all the studios?

R	Not specifically studios but, some one tells you that you have a nice voice maybe when you sing and put on your status, someone says you have a nice voice so with time they say I’m helping you with music, what are you giving me in return? They don’t have to say it directly, but you can have

I	*(Interjects)* read between the lines

R	These people, they are really nice by the way. They give good first impressions but afterwards they change so you just have to do something else

I	Okay but you still have the heart to pursue your music later

R	Yeah, I sing but I don’t ask for help from anyone, I just want to finish my university, start working then get back to doing music

I	Okay, so all these challenges that you go through, to what extent have you succeeded in dealing with them?

R	Uhm, most of them, I’ve never really found the best solution, okay I don’t run away from them but maybe I seek help from my friends who are medics, some from my parents but not all

I	Okay so you have friends who have medical background?

R	Yeah, many friends

I	Okay, what about other challenges that are not medically related?

R	You know I don’t trust people easily so some I keep to myself, some I just let go, some I talk to people, I don’t really talk to people about my problem most of the times so

I	*(Interjects)* So how do you feel when you bottle up all these problems? When you have these bad feeling and you don’t have someone to share them with, how do you feel about that?

R	Okay, the feeling is a bad one, it is not easy, sometimes you feel like giving up but based on the things that I want to do, you just press on but it is not easy

I	but you are able to find friends that have medical background, how do you fell after that?

R	I feel relieved and I feel much comfort in them, so it is kind of easy, but I don’t share with them everything

I	You don’t share everything

R	Just specific things

I	You just choose what to share?

R	Yeah

I	Okay, on all these that you go through, what support is available to help you deal with these challenges?

R	Support, as I had said, I have some friends who are much older than me, they are the only ones, and they are specific, they are not my relatives, they are just my friends, they are the only ones who help me

I	Okay, they help you in what way?

R	They tell me what to do, they advise me then I make the decision on my own

I	Okay, and do they have medical background?

R	Yeah, my friends are doctors

I	They are all doctors?

R	Yeah

I	That is nice, alright, on your HIV care services, could you describe to me your experience the day you enrolled in to HIV care

R	It was not easy, I was young, and I had a lot of questions

I	Do you remember how old you were?

R	I was nine years

I	Okay were you in grade school by then?

R	Yeah

I	How long was it after knowing your status that you were HIV positive?

R	It was like I don’t know, I don’t know what happened, you know before I used to take the drug, but I didn’t know why I was taking them, so I was very active

I	Like an active child?

R	Yeah and I used to ask a lot of questions, I think that is why my mum told me much earlier, though I didn’t understand

I	*(Interrupts)* but you got the information

R	Yeah

I	Okay, and when you were brought to the facility, do you remember where that was?

R	Uhm, I only remember Lumumba

I	Lumumba hospital?

R	Yeah

I	So there is where you still take your medication?

R	You mean the test or?

I	Okay, when you were enrolled into HIV care, you were definitely taken in to a facility, so which facility were you taken to?

R	Lumumba

I	Do you still take your care from there?

R	Yeah

I	Okay, and at that time when you were being enrolled, do you remember what was going through your heads at that time?

R	I had a lot of questions because I was the only child out of four and again the third born so I was like why me and such kind of stuff, so those were the questions

I	So you had two brothers or two sisters

R	Two sisters ahead of me and one brother

I	And what are their status?

R	They are negative

I	All of them?

R	Yeah

I	Even the younger one?

R	Yeah

I	Okay so you were asking yourself why?

R	Yeah

I	Okay, and is there anything in particular that made it easy for you to enroll on that day that you were brought in to the facility?

R	Support group because I came to learn that we are many children, I knew HIV was for adults and cursed people, I did not have the education by then, information was not that good

I	Alright, and as an individual, what has enabled you to continue engaging in care at the facility

R	At least I learnt that it is only a condition, it doesn’t hinder one from doing anything, yeah

I	Alright, how far do you live from Lumumba?

R	It’s not that far

I	Where do you live?

R	Russia quarters

I	Is that why you have medics as friends?

R	No

I	Is it not related at all?

R	No

I	Alright, what about quality of services at the facility

R	The care givers are just awesome and understanding, but maybe to me

I	Yes to you as an individual

R	Yeah, they are good, they have helped me a lot of times

I	What about the staff at the facility, what is their attitude towards you as an individual?

R	That one I don’t know how to answer, because I dint focus on people, so I don’t even know what they think

I	Okay, but would you term them as friendly or unfriendly staff?

R	To me they are friendly

I	Alright, if you can tell me a little bit of any instance where you experienced stigma or discriminated against because of your HIV status

R	Maybe many times I battle with it but the one that I was shocked with was when I was in high school and I was in boarding, by then I knew my condition so when I was in high school I really fought for leadership, and I was a leader in many things so I had to tell everyone to get out of the door but at the back of my mind I want to take my drugs, so I was also the leader of the clubs like I was the chairperson of the drama club and now we were having an invent in Kisumu so I instructed some girls to like, do some duties and one of the girls was like you are disturbing us and yet you have left your medicines go and take them, I was shocked, that was the only time

I	How did they come to know about that?

R	I don’t know because I never told anyone, I think the person was checking through my bags and found them, but I think I was strong, I didn’t show them my feelings, I was just strong

I	How did you feel about it?

R	It was bad but… *(Becomes emotional)*

I	It’s alright, just let it go

R	That is just life

I	And is there also an instant where people have refused sharing with you their stuff of denying you from sitting for an exam because of your HIV status?

R	No because I don’t tell people

I	Apart from your parents, who else know about your status?

R	My boyfriend only

I	What is his status?

R	Negative

I	Okay, so tell me anything you know about ARVs

R	All I know is that ARVs, I call them Hope by the way, they help me boost my immunity and make my viral load to be low

I	Any other thing?

R	They prevent me from opportunistic diseases.

I	Okay, why do you call it hope?

R	That is the name I gave it because I am carrying my life in my hands, anytime I stop using it I think that will be the beginning of my failure

I	Alright, so that is why you call it hope?

R	Yeah that is my name for it

I	Okay, I would like to know if there is anytime you missed taking your ARVs

R	Yeah

I	What happened that made you miss taking your ARVs?

R	I was tired, it is not easy, I was just like, I was tired because taking these drugs everyday is not easy, but at least at this point that I have reached, at least I don’t take them twice a day, just once

I	Were you switched to the new one?

R	Yes, and I think that is a very good one

I	Do you remember how long this was when you stopped taking your medication?

R	When I was in form two, you know in that form there is a lot of pressure, you want to do this and this, it was just a burden for me, at first I missed for one week and I was just fine, so I said let me try another week and I was fine so when I came back to the hospital the viral load was high

I	So despite being fine the viral load was high, how did you go about that

R	I was talked to by my care givers and they told me that, I didn’t know that the viral load was high because I was just healthy, so they just talked to me, my parents, I saw there, like how they acted on it, they were not pleased, I didn’t want to disappoint them so I just took them

I	So would you kindly share the challenges you faced keeping your clinic visits or appointments?

R	Sometimes you fear going to take the drugs because you don’t know what people are thinking, those are again some of the reasons why I stopped my career of music, I don’t know like in the industry there is a lot of people and competition, so I was like what if people know about this

I	So in terms of coming to the clinic, we do have the appointment dates, do you miss your appointment dates

R	No, I have never missed

I	And your medication

R	Maybe if I forgot but I don’t think so

I	What are your general comments on the HIV care services that you currently receive?

R	They are just awesome

I	Awesome in what way?

R	I am just contented with the care I receive because it is something for free I don’t pay for it

I	Apart from not paying for it what other feeling do you have?

R	My care givers are friendly

I	So let me ask you, you have talked about your care givers for the second time, so apart from your parents, who else are your care givers

R	The doctors

I	Oh, the healthcare providers

R	Yeah

I	Alright, and how do you think your encounter with HIV services can be made more satisfying?

R	I don’t know, maybe what they can add, maybe they can bring a telephone line where if you have a question, you just ask and you are answered

I	Like a phone?

R	Yeah, phone number and another thing they educate the negative people, they educate them about HIV

I	Okay, what in specific would you want them to be told?

R	That it is not a crime to be positive and they should not fear HIV

I	So you told me that you were in boarding school

R	Yeah

I	So when there, for young people like you, how does this affect keeping your clinic appointments and taking your ARVs?

R	Okay, for me I don’t know but for others maybe the time that you are told to come back maybe you have exams, maybe your school is a strict one, but for me, my days used to come during the holidays or half terms

I	So there is no day you wanted to come for medication, but you were not able because you were in school?

R	Now that time when my viral load was high, my mum came.

I	So when your viral load was high, this was found out when you had left school or when you went back at home?

R	I was tested over the holidays and by the time the results were out, I was already in school

I	So your mum followed you to school?

R	Yeah

I	What was the experience?

R	I was shocked. She didn’t tell me I knew it when I reached home

I	So when she came, did she just come for the normal visit or what was it?

R	It was a weekday and a school day like she didn’t expect it because she was also told it was a serious case

I	So she was called by the facility?

R	Yeah and she had to leave work and come and the day, what made me start using the drugs was like she was with me in the vehicle but she is still doing her office work and I was like this is stress and I don’t want to stress anyone, so those are the things that made me come back to my drugs

I	So on sexual and reproductive health, what do you understand by sexual and reproductive health?

R	As for me I don’t know, these is all about sex, family planning, sexual education, sexual diseases

I	What is your understanding

R	Sex education, sexually transmitted diseases, family planning

I	Alright, what about access to reproductive information? What do you know about that?

R	Pardon

I	What do you understand in terms of understanding sexual and reproductive health information?

R	All I know is that, the education is done in the hospitals, I don’t know if it is free

I	Have you personally accessed the information?

R	Like I said from my friends

I	Just from friends, but within the facility you have not accessed any information?

R	No

I	Any other place apart from the hospital and doctor friends?

R	Online, YouTube

I	Wow, what is there on YouTube?

R	You see how it is pathetic, the videos, how to control them

I	STIs?

R	Yeah, talk shows

I	Talk shows like which ones

R	Like those white doctors, show on how to control them, they show you the videos and it is not a good picture

I	Alright, so do you know anything on safe sex?

R	The one with protection part? Yeah

I	Tell me something about that

R	Safe sex is whereby you and your partner you know your health status and knowing in that you consulted a medical person, yeah

I	Alright, and on contraceptive use, what do you understand about that?

R	I have never heard on anything to do with contraceptives because I don’t like them

I	Why don’t you like them?

R	The stories

I	Okay, what do you hear about them?

R	They make people barren, they make people fat

I	*(Laughing)* you don’t want to be fat?

R	No, but that barren part, by the way I have a friend who used to use them in high school and she used to overflow during her periods and it was pathetic

I	So those are some of the stories you hear about contraceptives?

R	Yes

I	But everything that has disadvantage has an advantage to it, what have you heard about contraceptive that you think is good?

R	It makes you not to have unwanted pregnancies.

I	And what about STIs and prevention?

R	What I have heard about them?

I	Yeah, or what you understand

R	All I know is that they are spread at a high rate and very fast

I	Alright

R	Another thing I know is that treating them is not easy, yeah that is all I know

I	To you as an individual, how important is it to you to receive sexual and reproductive health services

R	Yeah it is important because it makes me have limits of what to do and what not to do

I	Any other thing?

R	No

I	Those only?

R	Yeah

I	You have very few words *(laughing),* alright so where do you currently access information on sexual and reproductive health apart from YouTube friends and online?

R	*(Laughs)* Maybe like events

I	Any example of an event please?

R	There is an event I attended at Agha Khan hall and it was about a lot of things, but it was all about sex talk

I	Someone organized this and people were invited or how did you end up there?

R	Yeah, people were invited, and you know events, youths can’t miss and I saw there were very many organizations, it was not one

I	Do you remember the organizations

R	I think KEMRI, something to do with CDF. I remember posters, DREAMS

I	So for you, information is obtained from medical friends, YouTube, online, events, any other?

R	Posters but I assume the posters most of the time

I	You assume the posters

R	Yeah

I	Where are these posters placed

R	On the streets

I	The ones placed on roads?

R	Yeah, radio

I	Alright, that is nice, so for you as an individual where is the ideal place where you would want to access sexual and reproductive health services?

R	Event

I	At the events?

R	Yeah

I	Why is that?

R	Because as youths we like things that brings us together and then at the event you don’t know anyone’s status so it brings people together

I	So any alternative apart from events where else would you like it to happen?

R	As I had said there should be a line where you call, ask questions and you are answered

I	So the ideal place is like a hotline for adolescents to call and somewhere they could meet too?

R	Yes

I	So, to what extent are the sexual and reproductive health services offered to young people like you meet their needs and expectations?

R	It is so much helpful because at least it gives them limit, you see I had friends who like when we finished class eight they dropped out and when you come on holidays you get to hear that so and so has a child so you are like this is bad and the next thing you hear someone has changed and when you inquire more you are told that uhm, the rumor goes that they gave her family planning to help her at least it helps

I	So apart from contraceptives, what about these other services like access to information

R	I don’t know much but at least people should be informed and they have the exposure that they lacked

I	So, you as an individual, do you feel like it meets the needs of the adolescents?

R	Yeah

I	You told me that it brings limitations, so what other thing does it do?

R	At least it helps them, when they are limited to somethings, they plan their future well, it helps the sexually active people

I	How does it help them?

R	At least they don’t get pregnant. By the way does it hinder them from getting STIs?

I	No, but it depends with the contraceptive that you use, so if you like use the male or female condom, I would say yes it will, but if you do not use condoms then definitely not

R	Most of my friends use, the one they put here (pointing her upper arm)

I	That one is called depo, so when that one is done, mostly done with married people so for young people it is really advised to use condoms and things like that

R	You see, that is another problem because the friends think it even prevents from getting the diseases, so it is only pregnancy?

I	Yes, so because you know how HIV is transmitted, so that one does not prevent HIV at all or even STI, so what challenges do you as a person face when trying to access sexual and reproductive health services and information?

R	Uhm…

I	Let us start from, do you access the services

R	By the way yes but the challenges I face are maybe back in the health centers when you are used to one clinician or doctor and then you come back another time and you find the clinician is gone so what you think is that all your secrets are going somewhere else so you have to start, that is the only thing that gets me worried

I	Okay, what do you deal with that

R	Okay what I did or rather what I do, I still talk to them because they are still good but I still communicate to the ones that were there

I	How do you get their contacts?

R	They give me

I	Alright, earlier on we were talking about young people being in school and the challenges that they are facing like when taking their ARVs, keeping clinic appointments, so I would like to know on how being in school or college affect access to sexual and reproductive health services?

R	When you are in school you have to go back home most of the time reason being you have to sort some stuff about your health and you are like you are in a school and let’s say a girl school for that matter, so they will start raising eyebrows like why do you go to the hospital most of the times or rather why do you go home most of the times like an example is that some schools they don’t allow you to keep the drugs, you have to go to the nurse and keep the drugs there and you are given in bits so going there every time they will just know that you are going their for medical purpose so the nurses too are human being, you can’t trust them

I	Okay, I think this is a little bit off but still within, since we started the discussion, you have really talked much about trust issues, I’m just wondering if you have major trust issues with people

R	Yes but those ones are confidential

I	Okay but they are things you have been able to manage?

R	Solving them?

I	Yeah

R	Yeah I have managed

I	Okay, so how comfortable would you be discussing your sexual and reproductive health issues with a lay female healthcare provider of your mothers age or someone who is your peer

R	I would be comfortable but male

I	Male, so someone say your mothers age but male or your fathers age

R	Yeah

I	Okay, what brings that difference?

R	Most of my friends are male, they understand better. Girls, I’m a girl so I know myself

I	Okay

R	And again not my peers, most of my friends are not even my peers, they are much older than me even by 20 years

I	Wow so you don’t hang around people your age?

R	I do but maybe for fancy stuff, having fun but I don’t share my problems with them

I	Okay, so what issues would you be comfortable discussing with these healthcare male providers of your fathers age

R	Sexual issues, about life, I’d seek help yeah, things that I can’t share with my parents I share with them

I	So where would you like these discussions to take place?

R	Events, through the phone or we just talk face to face

I	Do you have like an ideal place where you’d want to meet for such talks

R	Maye in an open place but we are just two.

I	Alright, why would you choose such a place

R	For example a hotel we are just talking, just as I do with my friends, we are just in a hotel having drinks and talking

I	So you are having drinks and sharing at the same time

R	*(Interjects)* or if it is too sensitive and can make me cry, personally we can go to the hospital and spare time for me

I	Alright, so you have been able to access them easily and be able to talk to them

R	Yeah

I	Okay, so we are almost winding up and I would like to hear your views on interventions that you as a person would like to be incorporated in the system to be able to help young adolescents access sexual and reproductive health services easily, so I would like to ask, how would you as an individual feel when we assign you a lay healthcare provider to be your confidant and source of information on sexual and reproductive health

R	Like you give me one person

I	Yeah, we assign one person to you

R	One person that I don’t know or?

I	You can either know or don’t know the person, which one would you prefer?

R	Maybe if I knew the person

I	So you want to choose the person assigned to you?

R	Yeah because I really have bad issues with trust

I	Okay so you’d prefer that if there will be anyone assigned to you, you be part of the choice making

R	Yeah, someone that I am used to, maybe my leader in the hospital or the peer leader, maybe one of the clinicians

I	What if they are a lay healthcare provider, someone who works at the facility but do not have a medical background

R	I think those ones are the best

I	Okay, so

R	*(Interjects)* provided they are older

I	And this one that you told me that you’d prefer male

R	Yeah male or if it is female, old

I	Older than your mother?

R	The one who has experience in life

I	Alright, such a person, would you prefer if they were your mum’s age or older?

R	Even older than my mum

I	So if we do decide that XXX we want to assign you such and such a person, what would be your ideal characteristics of such a person?

R	Secretive, humble and not judgmental

I	Any other thing?

R	Social and interactive

I	Alright thank you. What would you feel about receiving your ARVs together with your sexual and reproductive health services together?

R	It is an added advantage

I	In what way?

R	At least I can go with two problems at the same time and gives me an easy time and it may ease my trust because it is the same person I’m talking to

I	Okay, so of these services what will be the advantage of receiving ARVs together with your sexual and reproductive health services together?

R	I think that is the best one actually

I	What would make it the best?

R	I don’t have a problem accessing them because I will be accessing them at the same place

I	Okay, what would be the disadvantage?

R	Maybe if you keep telling your issues to one person, I don’t know, okay fine even that person is human, what if that person disappoints me, you know those are sensitive issues so I will feel bad the problem comes in when that person decides to share your issue with others

I	Alright as we come to the end of our interview, is there anything else you want to add on what we have discussed?

R	I don’t know much about this organization, so what I want to know is that maybe you provide this HIV education to the negative people, you see some people take it wrongly, I told you I don’t trust people easily but maybe you ask like if I were HIV positive what would you do or like, yeah like a friend who is HIV positive, what would you do and they will be like I can’t do this, the answers are really bad and scary, if not for my strength then, maybe to others because I’m concerned about others, me I have learnt how to deal with my issues but maybe they educate them that they should not despise the person, they should care because it is not easy

I	In terms of healthcare services and anything that you would like us to add on care?

R	Like I have said I am just contented

I	and in terms of activities?

R	They should organize more events that bring us together, yeah we have them but they are not enough

I	What events do you have?

R	The support groups, sometimes we have them in different places but they should be more

I	You wish they could be more?

R	Yeah because you feel good when you are people who you understand each other

I	How frequent would you want them?

R	It’s always once, so maybe twice during the holidays

I	Thank you very much for your time and thank you very much for answering all my questions we have now come to the end of the interview and the time now is 1602hrs thank you

Name of study: AYA STUDY

Type of Interview: IDI 031

Date of interview: 15-Aug-2019

Start time: 12:00 pm

Stop time: 12:45 pm

Location: UNIM

Name of interviewer: Irene Okumu

Name of transcriber: Irene Okumu

I	 Welcome to today’s interview. The date today is 15-Aug-2019 and the time now is 12:00 noon. Everything we discuss here will be confidential. First of all, I would like to ask you the challenges that you face as an adolescent and tell me a little bit about yourself.

R	I am XXX from Kisian and am a form four at Sinyolo Girls. For me, the challenges that I do face as an adolescent, I thank God for giving me a curvy body {Laughs} and I find it difficult walking around because everyone is looking at you and if you refuse to talk to someone, they insult you.

I	What age are you?

R	I am 19 years old

I	Is there something that you do back at home even part time to get some money?

R	No, I don't.

I	So, you've said that the curvy body that you have is your trouble, is there any other challenge that you have?

R	I am a silent girl and I do take everybody as my friend so most of the people take that as an advantage.

I	And in terms of your social life, do you have any challenges?

R	No, I am a friend to everybody

I	These challenges that you face a s a young person, how do you think they affect your education?

R	They affect my education and mostly the boy/girl relationship. I am in a relationship and most of the time I am a mature and there are some things that I would like to discuss with my boyfriend as mature people because he is also a mature person

I	(Interjects) How old is he?

R	He's 24 years

I	I assume he's not in high school

R	He's not in high school. So, like now am on holiday and am planning to go back to school, maybe he wants us to meet and when I refuse, he gets angry (Laughs) so when I go back to school, to be frank I will just imagine the way he is feeling.

I	What does he do?

R	For now he is working as a primary school teacher somewhere around Kisumu

I	These challenges that you have, how have you tried to deal with these challenges?

R	We have a guidance and counselling teacher in school so I normally approach her and speak out what is affecting me and she helps me with what to do.

I	When you discuss with her, do you feel relieved?

R	I feel relieved because she is a very mature woman and she knows how to deal with girls.

I	Any other way that you have dealt with these challenges apart from your guidance and counselling teacher?

R	My mum. My mum is my first role model and my mentor too.

I	Why is that, I would like to hear more about that

R	She knows how to advise me like her daughter, she brings me closer.

I	How many children are you?

R	We are seven but am the last born

I	How many girls all together?

R	Four girls and three boys

I	You at least have a way of dealing with these challenges, you have your guidance and counselling teacher and you also have your mother. What about your siblings, do they also help you work on these challenges?

R	No, I don't like sharing my problems with my siblings (Laughs) I trust my mum.

I	Your relationship problems, can you discuss with your mum?

R	Yeah

I	Okay. That's nice, and to what extent have you succeeded in dealing with these challenges?

R	Come up again

I	Okay, the challenges that you have, you've deal with them through the guidance and counselling teacher and the other way is your mother. Have you succeeded in dealing with the them?

R	Yes.

I	To what extent?

R	Probably my performance in school was very poor but when I came and spoke out to both of them, at least now my performance is now good.

I	And is there any other support that is available to help you deal with challenges apart from the two that you've told me?

R	My friends

I	In school or back at home?

R	In school, I have a very best friend and we share our problems like girls, we talk and advise each other.

I	Are you in the same class?

R	Yes, she is my classmate

I	And have you disclosed to her your status?

R	Yes, she knows everything about me.

I	And she has not talked about it to anyone?

R	No

I	That's good. It seems that you have three different ways of dealing with your issues, that’s interesting. So, on your HIV care services, for how long have you been on care?

R	Since I was in class four. Let me say at birth but for me I never knew about it until I reached class four when a nurse by the name Mitto, she was working here (To mean Lumumba Subcounty Hospital) but she retired. She came to my mother because that time I was sickling, every day in hospital and hospital was my home. She told my mum that my health was bad and that she should look out for other ways to deal with it. We then came to Lumumba and I was tested and I got into medication without knowing the reason why I was taking the medicine. When I reached class four, I started complaining to my mother and wanted to know why every time i was the only one in the family taking drugs while the rest dd not take. She told me that I had Asthma so i took medication thinking that I had Asthma. When I reached class eight, I was somebody who could be told something so my mother disclosed everything to me and she told me that I was HIV positive and that I had to take it that way and move on with my life.

I	How did you feel about that? What was going through your mind at that time?

R	I asked her I got the infection and she told me that I was born with it and so there was nothing that I could do since I was born with it. I just had prayed and asked God to help me through my life.

I	How has it been?

R	It is not easy by the way. It is not easy but it was easy when I was at home with my parents as a day scholar in Primary but now in secondary it is hell. When I joined form one, my drugs were kept by the matron and since that matron was an old lady, so when the time comes for me to take the drugs and the teacher is still in class so you can't leave. So, one day, she found me on the pavement and shouted "you, do you expect me to follow you so that you can take your drugs?" I just ignored her and so that my friend told me that the matron was calling me and she wanted to know the reason why i was assuming the matron. By that time, I had not told her anything about my status so I just told her XXX just leave me alone, I will deal with the matron later. I did not like the way she talked to me so I went to the deputy teacher and told her. That brought an enmity between me and the matron. In form one third term, I was not feeling well and I was wondering how this woman was going to talk to me. So, I l stopped taking my drugs and in did not take my rugs in form one third term

I	The whole term?

R	Yes. I did not take the drugs and when I came back home, my mum noticed that I was weak and she wanted to know why. Since I cannot hide anything from her, I just told her the truth. When I joined form two, we went with her to school, she raised the issue and it was dealt with. In school, you cannot miss to find mad girls, so gossiping in school. I was being talked about and they said XXX is on medication and things like that. I just sat down and said that if this God is the one who has brought me this far until I have joined high school of which I never knew that I would join, let me just take them positively and leave them to talk. So, in form two second term, I decided to tell my friend the truth. I told her and she went and warned those girls (Laughs)

I	She started fighting for you

R	By the way she is a tough girl. She told them that it was not good to talk that way about anybody if you don't know the truth and you only hear rumours and you are talking about it. By the way, in our school life is not easy because the diet with the drugs, no shopping so persevering.

I	How did you feel when your friend now took up the fight for you?

R	I felt wow and I knew that I had someone who can support me and fight for me.

I	I think that is really interesting, you could not fight back but someone else came and was fighting for you.

R	And fought for me. She asked me "XXX, why are you crying let's go to these girls and warn them, so for me, I said no maybe I can go to them then they start again and she told me, now let me go and face them.

I	Did they ever bother you again after that?

R	No

I	I would also like to ask what has enabled you continue engaging in care at this facility.

R	At this facility?

I	Yes. Is this where you have been receiving care all along?

R	Yes, Lumumba is my best

I	Why have you been able to stay here, what has made it easy for you?

R	They are friendly people and they know how to talk to people like us and bring us closer. At least you know that when you go to Lumumba, they are just friendly.

I	What about in terms of distance from here to home?

R	Since it's my health, I just have to take it though it is far.

I	What about the quality of services that you find at the facility?

R	They are good.

I	For example what

R	(Laughs) Let's say the activities that are normally carried out here

I	Like which ones?

R	The OTZ (Operation Triple Zero), the OTZ activities even though most of the members have left but it's still okay.

I	Left to where?

R	They have transferred

I	To other facilities?

R	Yeah

I	Okay, would you know the reason?

R	I don't know.

I	You've told me about the experience that you had in school about where the other girls were talking about you, could you also tell me of any instance where you experienced stigma or you were discriminated against because of your HIV status?

R	I have not because I am an indoor girl so it is very rare for me to associate with people.

I	That is at home, what about at school?

R	At school, it was only that one and probably the teachers because my class teacher knew so she is normally a woman who likes discriminating according to performance so I don't know what came over her. She went and told other teachers in the staffroom.

I	Oh, how did she know, who told her?

R	At that time when I was sick in school, the matron she is friends with the matron so asking for permission you have to pass through the matron and the class teacher. The matron told her that XXX is supposed to go and pick her medication and she was wondering what medication so the matron asked her "You've never known that you have a HIV positive girl in your class?"

I	So, she went and told the other teachers?

R	Yeah but the maths teacher came and told me what was going on in the staffroom. She told me to take it positively since gossiping is one of the characteristics of women in the society.

I	Apart from the Maths teacher, did any other teacher show you a bad side now that they realized you were HIV positive?

R	No.

I	And what about the students, did they do stuff like I can't sit near you because you're HIV positive, I can't share this with you?

R	No

I	You already told me that you are on ARVS, I would like for you to tell me anything that you know about ARVS.

R	ARVS are very strong drugs and if you don't have the heart, you cannot make it.

I	Why?

R	Mostly after taking them, you need rest and you have to eat well so it also depends with the parent and the family that you're coming from. Even if it's a poor family, you just have to struggle and you have to make sure you eat a balanced diet.... (Inaudible segment)

I	You've told me about the time you stopped taking your medication when you were in school because of what the matron had done. So, tell me about the time you had stopped taking your ARVS apart from that one about the matron, is there any other time that you ever missed?

R	Yes.

I	Okay, what happened?

R	In form four this year. We were having an exam at exactly 6:30

I	PM?

R	Yes. So, it was a maths paper and excusing myself was not that easy so I just let it go for that day.

I	Was that the only day you missed or you missed other days also?

R	Only that one.

I	You as an adolescent, could you kindly share with me the challenges you face keeping your clinic appointments?

R	How? Explain further.

I	Okay, you find that one is given a TCA of let's say after every 3 months you come back for your clinic appointment. Is there something that you feel is a challenge for you to keep those clinic appointments?

R	Yes. Most of the time I don't normally come to the clinic when am in school. It's my mum who normally comes.

I	She comes and gets the medication for you?

R	Yes. I only come when am supposed to be tested for blood (To mean drawing blood for Viral load test)

I	At home during the holiday, is there anything that would make it a challenge to come?

R	No.

I	Also I would like to ask you, that last time that you missed your clinic appointment you said that your mum is the one who came for you. So every time that you miss your clinic, is she the only one who comes for you?

R	Yes, she's the only one who comes for me.

I	And this one is all because you're in school.

R	Yes

I	Is there any other reason that you missed your appointment and she came to get medication for you?

R	No. I normally come.

I	And what are your general feelings about the HIV care services that you currently receive?

R	Let me say, when I came to Lumumba, I was like a person who was about to die

I	That's when you were in class four?

R	Yes. I was thin (Laughs) but coming to Lumumba, after all the guidance that I received by the way I was guided when I was in class eight on how to take care of myself now and now live to accept myself.

I	Any other feeling?

R	No

I	How do you think your encounter with HIV services can be made more satisfying? Is there something you want us to improve on?

R	Let me say, as for me am a student and students mostly in our school we don't carry food stuff so you might find a parent is maybe giving you Ksh. 500 and he/she expects that you call after one month and with all the hunger in school, if you sit and think that at 7:00pm am supposed to go for my drugs and I have only eaten vegetables (Laughs) How will I be able to stay up to 11:00pm in class?

I	And what about at the facility, is there something that you would like for us to improve on to make it better?

R	For me, Lumumba is just okay.

I	You've told me that when you're in school, you don’t come for your medication, it's your mum who comes and also taking your medication in school, it's a little more difficult than when you are at home. So how does being in school for young people like you affect keeping their clinic appointments and taking their ARVS?

R	Let me say, I am supposed to come back to the clinic like tomorrow and that same day I have an exam, even if not a exam because an exam is not an issue. Some of the students like me and my fellows seeing you every day going at home, they will be wondering why you go home all the time and what your problem could be.

Another thing is when it reaches time for you to take your drugs, each and every time you're out of class going to the matron, girls will always remain to be girls and they will always remain to be gossipers (Laughs) immediately you leave class they will say "She has just left, she's now going to take her medicine" so things like that.

I	So, you feel that is really a big challenge

R	Yeah

I	On sexual and reproductive health services, you indicated that you are sexually active from the form that you filled, I would like to ask you what your understanding of the term sexual and reproductive health is?

R	(Silence)........Eh eh

I	Okay, let me make it a little easier for you. On sexual and reproductive health, we have got different activities. We have got reproductive information that someone can get, we have got safe sex, use of contraceptives and we also have STI prevention. Maybe I would first ask you, do you know anything about reproductive health information?

R	Let me try, there are many diseases and we also have how to prevent pregnancy and things like that. As for me, I personally (Laughs) am not under any contraceptive and am not feeling like being under any of them.

I	What would be your reason for that?

R	(Laughs) my time has not yet reached for me to be involved in such things.

I	Okay, and when is the right time?

R	(Laughs) maybe after form four.

I	And what about safe sex, what do you know about safe sex?

R	Safe sex is maybe when you want to engage in sex and you use condoms.

I	What about STIS, do you know anything about STIS and their prevention?

R	STIS and prevention, you can also use condoms to prevent it.

I	This information, how important is your access to sexual and reproductive health services, do you get these services you as a person?

R	Yes, in school.

I	What is offered in school?

R	The guidance and counselling. We have it every Thursday and it's compulsory for everyone.

I	One on one or in groups?

R	General, the whole school.

I	Who runs that?

R	Our guidance and counselling teacher

I	Okay, does she teach any other subject?

R	Yes, she teaches French.

I	Therefore, these sexual and reproductive health services that you get, how important is it to you?

R	It has really helped me a lot. There was a time, I told you that I have a boyfriend. There are temptations mostly when you are together of which those temptations are not very easy to prevent but there was a time that we were tempted and we engaged in sex and we used condoms of which we were advised in school that

I	(Interjects) Oh okay, so this one you had already been taught in school so you had an idea.

R	Yeah

I	Alright, does he know your status?

R	Yes, he does.

I	What about him, do you know his status?

R	Yes

I	Is he also HIV positive or he’s HIV negative?

R	No, he is negative.

I	You also told me that you access information on sexual and reproductive health in school, where else do you access this information?

R	My mother. My mum is one person who will always tell you, she will always call a spade a spade (Laughs) and anytime we are with her and she feels like advising me, she does that. She will tell me XXX I know you have a boyfriend so what you need to do is this, this and that since you also know your status and it is not good if you infect the other person.

I	Where does your mother work?

R	My mum is a business lady

I	These services that you normally get, apart from the information, is there any other service that you get on sexual and reproductive health?

R	Yes. My boyfriend is another person. We talk and he tells me that "You, what happened last time was not good but we are supposed to accept and it will not happen again, we have to do this, this and that"

I	That's interesting. Okay, for you, what would be a good location where you'd like to access your sexual and reproductive health services?

R	My safe place, at home.

I	Why at home?

R	At my place, my mother's place.

I	Why would you choose home instead of other places?

R	I am an indoor girl and I don't normally find it easy to get out unless I have been sent.

I	And to what extent are the sexual and reproductive health services offered to young people like you meet their needs and expectations? Now, this one is not just you, even the other adolescents, do you think it meets their needs?

R	To some, no.

I	And to others?

R	Yes.

I	Okay, you tell me the yes part of the response and then you tell me the no. So how does it meet their needs and to what extent?

R	To some girls, let's say you are being advised, you take it the way it is and you follow the instructions the way you're being given and if you follow all the instructions that you're being given, you will find it smooth for you to be in a society and also to be in a relationship with someone because you know if this comes you know the actions to take. Also, my no part of the answer, this issue of pregnancy, for me pregnancy is something that somebody wants (Laughs) yes, there is no accident.

I	(Laugh) But even married women say it's an accident

R	(Laughs) No, no it's not an accident.

I	Okay, why is that?

R	Let me say, mostly in high school somebody learns from form one up to form three then she gets pregnant and since she joined form one, she has been advised throughout the term and also our principal also normally says "Choices have consequences" so if you have it in your mind that when you go home you have to do this and you don't plan to use anything (To mean unprotected sex) so how can you say that someone is pregnant and that it is an accident? There is no accident.

(Both laugh)

I	So, it's something that people do intentionally?

R	Yes

I	You as an individual, what challenges do you face in trying to access sexual and reproductive health information or services?

R	(Participant's phone vibrating) Let me say, if somebody wants to advise me then a crowd of people just look at me, I feel it's a no.

I	Any other challenge?

R	Being advised then you tell me "Let me just find that you've done the same" I don't like being told that.

I	They should just advise you and let it go with no extra comments?

R	Yes. Not that everywhere you meet me you have to make a comment warning me not to do what you advised me on, I don't like that.

I	When they keep commenting, does it now make you want to go and do what you were asked not to do?

R	(Laughs) Yes!

I	Why is that?

R	(Laughs) Ai, if you’ve advised me once, it is fine and I've followed all the instructions, so why keep on commenting like that all the time?

I	Any other challenge that you face?

R	Only that.

I	Okay. Our interview is coming along well. So, you told me that you are in boarding school, so how does being in school for young people like you affect their access to sexual and reproductive health services?

R	Somebody like me, I don't like guidance and counselling.

I	Oh, why is that?

R	I don't like reason being: you're being advised, you open school and you hear the principal calling out a list of pregnant girls so you just wonder after all the talks that we have been having with the teacher now this girl, for me after seeing the list of those pregnant girls I just see there is no need of going for guidance and counselling. It's better I go and hide in the library and read. There is no need.

I	Why because it's not you who is pregnant, it's them?

R	It is embarrassing. How can a teacher just waste her one-hour lesson coming to talk to you girls then you disappoint her, it's not good.

I	So that's one of the challenges. But what about in terms of getting services. Let's say someone wants services on family planning or information, is that a challenge being in school?

R	We've never had such a talk in school. They normally just advise us to be careful with the men and in fact our principal usually says "Don't just open your legs" (Laughs)

I	(Laughs) Just plainly like that?

R	Yes. Don't open your legs.

I	And when people come back the following term?

R	Their legs were open so she normally feels embarrassed.

I	For you, how comfortable will you be discussing your sexual and reproductive health concerns with a lay female healthcare provider of your mother's age?

R	I will feel okay.

I	Why is that?

R	Because she will be advising me and making me know more on how to take care of my life and how to protect myself.

I	Any other reason?

R	Because she will be older than me, she knows everything and she has lived long, she will be giving me some knowledge to know all about sexual reproductive health.

I	And what issues would you be comfortable discussing with this lay healthcare provider?

R	About my relationship status, I will be very free with that and discuss a lot with her.

I	Any other thing that you can discuss with her?

R	My health status.

I	So we have relationship and health. Any other thing? Sorry if am digging too much.

R	(Laughs) Only those

I	Alright. And when discussing with her, where would you like to have these discussions?

R	In a private place, just the two of us.

I	At home, at school, in town?

R	At home.

I	Your home, her home or it doesn't matter?

R	It doesn't matter.

I	Why would you choose that? I mean home.

R	It's comfortable.

I	(Laughs) Because of the chairs?

R	(Laughs) Yes because of the chairs

(Both laugh)

I	It can't be the chairs only (Laughs) What other comfort does it come with?

R	At least we can have a private room just the two of us discussing.

I	Okay, we are almost coming to the end but before we close, I would like to have your views on potential interventions that you would like incorporated  and I would first of all ask how you would feel if we assigned you a lay health care worker to be your confidant and source of information for your sexual and reproductive health?

R	Come up again.

I	So, in the other session, we talked about your feelings and if you would be comfortable having someone in your life who is lay healthcare provider of your mother's age right?

R	Yes

I	I am now asking as an intervention if I decide that now, I want to assign to you Mary (Pseudonym) to be your confidant and someone you can discuss with your sexual and reproductive health issues. How would you feel about that?

R	It will be just okay provided that anything we discuss will be between me and her

I	And what would be your ideal characteristics of such a lay healthcare provider. What characteristics would you want her to have?

R	She must be friendly and understanding.

I	Okay, those are two

R	Caring and at least she has to accept me the way I am with everything that I will tell her about me.

I	Alright, and how do you feel about receiving your ARVS together with your sexual and reproductive health services? In the same room kind of thing.

R	It's okay.

I	Explain to me why it would be okay.

R	It would be okay because it will save me a lot of time.

I	Apart from time, what else?

R	I'll be having time to talk to her about what is affecting me.

I	Another one, let's get about five

(Both laugh)

R	I'll be free with her.

I	And what would be the advantage and disadvantage of receiving your ARVS, sexual and reproductive health services together at one point? So, you've told me that you'd prefer for it to be in one place, so what are the advantages of having it in one place?

R	It is just okay because in studying sexual reproduction, we also have some of the diseases that you get when you engage in sex and one of them people believe that it's HIV/AIDS so we might talk about the cause of HIV/AIDS and also the cause of STIS that we have. IT's fine to be put together.

I	And what about in terms of just accessing let's say family planning services within the same room where you get your ARVS, do you think that will be a good idea?

R	Yes, because even in our OTZ studies we are also being told about the use of contraceptives and I think it’s just okay.

I	What do you think would be a disadvantage of putting the services together like that?

R	For somebody like me, now you want to show me on how to insert those things in your body (touching her upper arm)

I	Ooh, the implants?

R	Yes, the implants. Since am not using them there is no need

I	Any other thing that you feel may be a disadvantage?

R	Only that.

I	Alright, so thank you very much for the information that you've given us and we have come to the end of our interview, it’s now 12:45 pm. Thank you

**Study ID:	033**

**Site:	Lumumba**

**Time:	0946Hrs to 1030Hrs**

**Date:	05 Sep 2019**

**Interviewer:	Belinda Odhiambo**

**Transcriber:	Irene Defrates**

**Gender:	Female**

I: This is Aya participant 03 Qualitative Interview conducted at Lumumba clinic on 5^th^ September 2019 and the time is 9:46 a.m.; by Belinda Odhiambo. Okay, I would like to begin by asking a bit about yourself. Yeah. Can you tell me about yourself?

R: Okay, myself as my name you know, I’m Rose Atieno (not her real name) from…I stay in Kisumu. I am a graduate from Maseno University. I tested HIV positive in December 2013, since then I’ve been on drugs;

I: 2013?

R: Yes, but I started taking them in 2014.

I: Alright, thank you for that. Are you currently doing any work?

R: No only that I’m starting to be… I have a contract to be a peer leader at Lumumba.

I: Peer what?

R: Peer lead, adolescent peer lead.

I: Are you married?

R: No.

I: Can you tell me the challenges you are generally going through now as any other person?

R: Related to my status?

I: Just anything, general challenges.

R: Okay, generally the most challenge is about getting a job; it is very hard to get employed in Kenya nowadays. Another challenge is when it comes to relationships at this age of ours, it’s very hard to be in a relationship due to me being HIV positive; you’ll find one ready to be in a relationship with you but the moment they realize you are HIV positive they will no longer be interested, they’ll think that it is a death sentence.

I: So you have faced these before?

R: Yeah, many times.

I: How do you feel about that?

R: Okay, for now I haven’t suffered from any stigma so it’s okay to me. The first experience was hard but nowadays I’m okay with it.

I: Can you tell how the challenges you talked about employment and relationships; can you tell me how these affect your educational or professional achievements?

R: For the relationship it doesn’t affect my professional achievement, for employment I think I have started somewhere since I have a contract now; it is a start because I graduated last year and can say that I’m starting to achieve something in my career.

I: is there anything that you did…let’s say after disclosing your status with your potential partners you might be in a relationship with and after them knowing about your status they to shut you out, or didn’t want to be a relationship with you, can you tell me how did you dealt with that?

R: Okay, how I dealt with that, most people when they hear about it they do not to talk and so I decide to keep quiet also. Others also do try being just normal friends and not boyfriend, they do support me in my treatment and in any other way; I have many friends by the way who know about my status, some of them begun as friends while other wanted a relationship but decided to just friends. Also I’ve been in a relationship with someone who is negative, he first accepted my status and we dated for about one year when he started to change; he began having many girlfriends and didn’t want to go for HIV tests to know whether he is okay or infected even though we always used protection. So we broke up. After that I also got in a relationship with another negative guy, we broke up because of his mother as she knew I was HIV positive and told me that I couldn’t be with her child… her son. And that is how we ended the relationship.

I: How does that make you feel as a person?

R: Okay, when the mother decided that I felt bad because the mother is also on care, so I felt bad why was she saying that I should not date his son and yet the son had already accepted me the way I am.

I: Okay. Right now if someone doesn’t want to date you because of your status. Do you feel you are ok with that?

R: Yes, I’m okay with that and for now I have decided to date someone like me, who would understand me.

I: Have you ever talked to anyone about this?

R: About my status?

I: Not really but about the relationships you want to have but are not working out due to your status.

R: Yeah, I have many people…

I: What did they say; did they support you in any way?

R: Yeah, they always support me and give me advice; they always tell that I’m still young and still have a long way to go and will find the right man who will accept me the way I am.

I: Who specifically told you that?

R: I have a friend called Nick he is always supportive, I have another friend called Maurine, she is also supportive when it comes to relationship issues.

I: Okay, thank you. We will now talk about HIV care services. You told that you tested positive in 2013 can you tell your experience the day you enrolled in HIV care?

R: The day I enrolled or the day I was tested?

I: The day you now enrolled in HIV care.

R: Ok the day I enrolled, by then I had I accepted my status, it was in January 2014. I accepted because my mother told me to; my mother isn’t that literate so when I tested positive I told her… by then I was still a virgin so I don’t understand how I got infected. My mother told me to go for the medication and will be okay with time as my younger sister who is the last born was born under PMTCT so she was given Nevirapine and some other drugs. After sometime she was discontinued from the medication as she tested negative, so she thought the same thing that happened to my younger sister would happen to me. That gave me the morale to come for the drugs knowing that I would turn negative at some point, I later learnt that the situation is permanent so I accepted it though I reached a point when it became hard for me to disclose my status that is when I joined campus. It is new environment; change of things and having roommates, I was lucky I stayed with one in my first year whom I had to tell my status as there was no way I would take my drugs there without her knowing what they were for. I told her my status and she was very supportive and would remind me of the time to take my drugs. In the second semester of the same year I stayed with another girl; I didn’t tell her my status for the four months I stayed with her, the reason being that she used to talk bad of people with HIV and I was afraid of telling her. I therefore used to take the drugs in hiding. After that I decided to be open and add no stress upon me, almost everybody around me knows my status now.

I: So far can you tell me your feelings about disclosure, what could you say helped you overcome stigma?

R: What helped was… like… I used to follow HIV activists in Facebook… the way they are open and can take their medications openly in a vehicle or party, so I said to myself that if these people could be so open and they are so free why could I not be like them, I therefore decided not to hide as it only adds a burden to you always thinking about what people say or think about you; so I decided not to care they about what they think as it is my life.

I: You told me that you tested positive in December and started HIV care in January. What could it be that made you wait for a month?

R: I was still in shock by then and I didn’t accept; I was saying to myself “why me” when all that time I had been a good girl with no boyfriend why test positive at this time? Initially I was taken to the hospital by our pastor as I had some tiny wounds in my feet, when the doctor saw he referred me to the VCT where I tested positive, I told the counsellor there not tell the pastor who took me there and I haven’t any idea if he now knows. I haven’t ever discussed with him my status.

I: So he took you and left you there?

R: No we even went back with him but he did not enter in the testing room where I told the counsellor not to tell him.

I: Did he not ask you? Like what was happening or?

R: Yeah. He did not ask; he only asked if I was going to JOOTRH which I think the doctor told him. So that’s why I didn’t get enrolled immediately also I was coming from Nyahera and they wanted to refer to a nearby hospital. I later talked to them to allow take the drugs from JOOTRH, it also reached a point when I went for another test because I felt that maybe there was a mistake and when I went back the results was the same; the line from the test result was light as I was already on medication and they wanted to test it again but I told them that I was already on medication at Russia.

I: What was it that actually made you decide to enroll in HIV care?

R: What made me decide is what my mom told me though I later realized that it is good to be on care because before I was enrolled I used to feel some headache, an unexplainable type which I can only say was bad, when I started using the drugs it disappeared and so I saw that the drugs could help.

I: What has enabled you continue engaging in care at the facility that you normally go to?

R: For now I know it’s good to be on care because when you are on care it reduces the amount of virus in the body and when your VL is suppressed you can stay for long and do your things normally with no disturbing opportunistic diseases in your body. That is why I can't miss taking my drugs and the clinic appointments.

I: Why is it that you only wanted to go to this facility you are in now?

R: I just don’t want… I’m okay with the clinic.

I: What is okay there, what do they do that you feel is ok; what is right?

R: The procedure is just okay, how they handle clients.

I: How do they handle you?

R: Okay. I can say that they are friendly, keep time and always encourage us by talking a lot about HIV; they have boosters have every six months where you get to know what to do to suppress your VL, how you should live, about sexual reproductive talk and many other talks such that you know how to handle your partner, they also help in cervical screening and many other things. So they help a lot.

I: Have you ever experienced stigma or been discriminated against such that some people don’t want to be around to you?

R: I have not experienced stigma, not after I got to know my status; before getting tested I did experience stigma though I wasn’t aware I was HIV positive. I was tiny and had the tiny wounds on me that community already knew I was HIV positive. There is this time in school when one of friend broke up with her boyfriend and so she thought I was after the guy which wasn’t true and so the guy came told me that I was the one made them misunderstand each other, he told me that he couldn’t date me because I was HIV positive. The boy stayed with the pastor who took me the hospital and was our family friend otherwise the girl thought I wanted to her boy’s girl. It is then that they told me I was HIV positive and did not belong in any relationship or should not be in a relationship. I ignored them because I hadn’t known my status yet. Another scenario was during a symposium in our school; a guy from a neighboring school gave me his number when I had some people telling him not come to as I was HIV positive. I used ask myself what they saw in me that had them take me to be HIV positive but all the same I ignored it too because I still dint know my status. In another instance, I went for the test, the guys came to our local center and during the test asked of my age which I told them was 17 years having not tell me my status and went ahead to ask if parents were around, they weren’t and I was told to go for another test later with her which I suppose is the time it really came out that I was HIV positive though they didn’t tell me. There were many people in the test and each shared their negative status when I on the other hand was not told of mine. I began to get stressed as to why everybody got their results when I wasn’t told mine. That is the time I experienced stigma but after test I don’t remember experiencing any stigma

I: Thanks for sharing that, can you tell me anything you know about ARVs?

R: What I know about the drugs firstly is that they help in viral load suppression, another thing there are various types of ARVs; that is the first and the second line ARVs, so when enrolled for the first time you given the first line ARVs and you are advised to take the drugs well so that you aren’t switched to another line.

I: Was there any time you missed taking your ARVs?

R: Mmmh, I think there was. It was once when I went somewhere and forgot to carry my drugs.

I: Okay. Can you share with me any challenges that you have experienced in making it for your clinic appointments?

R: I have never missed my clinic appointments; I do send my sister when I’m not around. The only when it comes to the appointment was during the time I was in school as you would find that I had an exam the same time I was to go for the appointment, any way as I had said they are supportive at the hospital and after telling them you had exams they would attend to you first and let you go for the exam.

I: So there hasn’t been any time you missed your appointment due to exams?

R: No, I have never missed; if I’m not around I do send my sister to get them for me.

I: What are your general feeling about HIV care services you currently receive?

R: I think currently the HIV care is good because it helps a lot; ARVs doesn’t have many side effects now as compared to before. Also the burdens of ARVs… okay sometimes for me I use only… I take my drugs once a day so I use the TLE which is taken once a day… for me I feel it’s okay, there is no burden but I feel for some people that they have a burden in taking their drugs, like for children, you will find that they are given many drugs especially those in second line; you’ll find a child taking four drugs so I always feel that there is burden for them in taking drugs.

I: Ok. What do you think could be done to make your HIV care services encounter to be more satisfying?

R: Well, I think it’s already in the process because nowadays there is the service after every six months and also the community groups where in a group you could send only one of you to go get the medication though I’m currently not in that group as I had issues with my viral load and discontinued of the six month interval visit, I’m currently working on it.

I: Do you work on the monthly or three months?

R: I was given a TCA for two months.

I: How do you feel about that?

R: I feel bad about it; when I checked my VL I was stressed because I don’t know the problem, it used to be time and I changed it, I change everything but still the VL is up and is stressing me but I’m working on it.

I: Okay. Moving to the sexual and reproductive health, what do you understand by the time sexual and reproductive health?

R: What I understand… I think its changes that occur like in adolescents where you start experiencing some changes in your body and become sexually active. Then.. that is how I understand it (both laugh).

I: Tell me about access to reproductive health information.

R: Like? Repeat.

I: access to reproductive information like anywhere or somewhere you can get information about reproductive health

R: In the hospital before the clinic services begins we always get taught where they mostly handle the reproductive health issues, also when I was in campus we had a unit concerning reproductive health.

I: What about safe sex?

R: On safe sex I’ve only heard about it in the hospital where we get taught, I’ve also heard it in support groups.

I: What of place you’ve been taught of contraceptive use, STIs and prevention measures?

R: Those I learnt from campus and at the facility.

I: How important is your access to sexual and reproductive health services?

R: It is important as you get to know what you need in life; like now being HIV positive I get to know the importance of having safe sex as we don’t want to have new infections and you’ll have to protect your partner if he is negative, if you don’t know your partners status then it would be important to practice safe sex to avoid reinfection; it important because it helps you.

I: You you have access to sexual reproductive health information at school and at the facility. What is the ideal place for you to receive sexual and reproductive health services?

R: I think anywhere would be good for; a facility, support group, or workshop.

I: Why do you think anywhere works for you?

R: Information is good from anywhere you get it; it could be school where they could start a workshop as they are the most affected in terms of sexual reproductive health. So anywhere you provide the information, it will help.

I: To what extent under sexual and reproductive health services offered to young people like you meet their needs and expectations?

R: To me I feel that the education I get on sexual and reproductive health is really helpful, but talking about on my experience with other young girls and men I think it doesn’t help them that much, maybe they don’t get much information; you’ll find these days that premarital pregnancies, early pregnancies are so much and I’ll say that there is still a lot to do when it comes to reproductive health talk.

I: So you feel that because of this early pregnancies there are needs that are not met?

R: Yeah.

I: Any other reason you have for them feeling this way?

R: Also, people are so ignorant and if not the early pregnancies you will find that a girl is Form One and is already on family planning forgetting that there are other sexually transmitted diseases like HIV and STIs. So when there is a proper education on sexual and reproductive health it could help as it will touch on the general topic.

I: So you think that some in the early stages should not actually be on family planning?

R: I think it’s good but it shouldn’t be encouraged that much as the frequency of having will increase; they will see that they can't get pregnant forgetting she could contract other infections.

I: What do you individually face in accessing the sexual and reproductive health services?

R: The only challenge is the needed information doesn’t reach to you when needed; you need the information urgently at one time and fail to acquire it. For me I’m lucky as I can always get the information at the facility. So what of others who are at home, they don’t go to the facility.

I: So as individual you are not facing any challenge in accessing the services?

R: Yes.

I: Okay. How does being in school for young people like affect the access to sexual and reproductive health services?

R: When in school especially the boarding school the schedule is always so tight that there isn’t time for sexual and reproductive health except being taught biology. In campus I didn’t attend any workshop; I only had a unit dealing with sexual and reproductive health. I therefore think you should organize more workshops in schools to deal with the services. In colleges they could be but we haven’t accessed the information; it could be organized but not everyone get the information that there is a workshop.

I: How comfortable could you be when discussing your sexual and reproductive health concerns with a female health care provider around your mother’s age?

R: I think I could be very comfortable sharing because I always share many things with my mother.

I: What if the health care provider is your peer?

R: My peer would be much better (both laugh).

I: So you prefer peer to an older person?

R: Yeah, you know there are certain things you can't share to an older person; you can't tell an older person everything about you especially the sexual issues. Some of your peer would be better as you would have the same experience and would be much open.

I: So can you tell me exactly what issues around these sexual and reproductive health services that you would be very comfortable discussing with someone your mothers age?

R: What I can talk to them about… maybe it’s relationship issues because any time I have…like the previous relationship I was in I was free to talk to my mother and get some advice on how to go about it. Another thing like… okay like sexual education you know touches on many things not only on sex, you could have a problem on your body and at such I would easily talk to someone older.

I: What of your peer?

R: I would be free to also talk to my peer on relationships; when it comes to peer there are somethings you can't share freely. Like I had this experience with a certain lady, the person was suffering from STI though I couldn’t tell if it was syphilis or other, I knew it was an STI yet I couldn’t approach her as she wasn’t that open and went to an older person, being that she was going to trust the older mother, this she did to keep the issue confidential; for someone like me we would go about spreading it. But for older people they can be trusted.

I: Okay. Having said that sexual and reproductive health services should be given anywhere it then follows that it doesn’t matter where, or could please talk more on it?

R: About that, anywhere means that any place we access… like mostly adolescents and… like let me say young adults under 30 years I think. Anywhere where including work places, hospitals, schools, and universities would help as you would have reached out to almost everyone and they would have that information.

I: Okay. How would you feel we assigned you a lay health care provider to be your confidant and source of information for you sexual and reproductive health?

R: I don’t understand.

I: So we just want to give you a person whom you can go to if at all you have any questions or any concerns about sexual and reproductive health, that is the person you will go to, if you want to talk to anyone about your issues with that, that is your point person. How would you feel?

R: I would feel good because… I think most people want that but they can't afford it because if you go to the hospital to see gynecologist you’ll have to pay a lot but if assigned one you will good and have more information; should you assign me one therefore I would be happy and share more with the person.

I: Can you tell the ideal characteristics of how you would want this person to be?

R: Okay, the first thing is that the person should be friendly another is that the person should be able to keep confidential issues also the person should be professional; you will find one working in an office and outside he/she goes about speaking about your issues. The person should not behave this way. Finally, the person should always be there for you.

I: What would you about receiving ARVs together with the sexual and reproductive health services?

R: In the facility?

I: Mmmh.

R: That could also be good as you would be having two activities at the same time therefore spending less time.

I: What could be the disadvantages of having the two services at the same time?

R: The disadvantage is that it could take a lot of time as when taking ARVs there procedures you have to follow and it could take you one or two hours to get served after which if you have to go wait again for the sexual and reproductive health services it would inconvenience you if you were in a hurry to go somewhere; like the I went to the hospital for cervical screening I left there at 12 p.m. when I went at 7 p.m. I really wasted a lot of time.

I: So it has both advantages and disadvantages.

R: Yes, what they should do is to have prior plans; they could see when one is coming for the ARVs and book her for the reproductive health talk, they should therefore work out quickly having in mind that the person is going for another service.

I: Do you have any questions as far as our discussion today is concerned?

R: Another question I have is like: what topics do you always teach/or prefer teaching in the sexual and reproductive health package?

I: Do you have an idea of the topics you would want to get addressed?

R: What I think is just…for me I think it is safe sex. Then another thing is that when it comes to reproductive health you could talk about cervical cancer screening and STIs. You could talk about STIs. That is it.

I: That is ok. Thank you. We have come to the end of the interview and we thank you for your participation and the time now is 10:30 a.m.

**Study ID:	035**

**Site:	Lumumba**

**Time:	1205Hrs to 1250Hrs**

**Date:	05 Sep 2019**

**Interviewer:	Belinda Odhiambo**

**Transcriber:	Irene Defrates**

**Gender:	Female**

I: This is AYA study qualitative interview conducted at Lumumba clinic on 5^th^ September 2019. The time is 12:05 p.m.; by Belinda Odhiambo. Let’s begin by you telling us about yourself.

R: I’m Lorita (not her real name, clears voice), I live in Kibos and I’m 20 years old. I’m uuhm a Form Four leaver… I’m jobless.

I: Are you working?

R: Not really, I’m jobless.

I: Sorry, I meant are you married?

R: No, I’m single.

I: Alright; can you tell me the challenges that you face as a young person in general?

R: General?

I: Uh huh.

R: Love…

I: Love; challenges in love?

R: Parental love.

I: Parental Love?

R: Yeah.

I: Tell me about that.

R: You know in this condition that I’m in, that love you could get from a parent or those around you is not there; second, intimidation…Mmmmh.

I: What do you mean by intimidation?

R: Like if there is a chance that I can do something the first thing somebody will talk about is your school and status and tell that you can't do that particular thing; so it is such.

I: So they think you can't do it because of your status?

R: Yeah.

I: Ok. When had you experienced such a thing?

R: It was one month ago.

I: One month ago. Can you tell me what happened?

R: There were chances; people were going to join NYS to do courses so… the people who were around by that time we were few Form Four leavers, so they asked if I could and I said yes. So someone from elsewhere just said “do you know your condition cannot make you to go there?” So she wanted to know the person whom I was living with and I said it’s all fine and then lets cancel it and give the chance to another person whom they felt like…

I: Has this ever happened to you again or it was just that once?

R: No, it was that once.

I: Who do you stay with right now?

R: I stay with my brother.

I: Okay. Any other challenges faced as a young person?

R: Buying of those… I don’t know how I can categorize them; buying things like pads and panties, at times it’s very challenging. I at times do these adult jobs to get them and at time I fail to get, so I have to use the little I have to move on.

I: Your brother doesn’t support you in buying these stuff?

R: A times he will but other times he won't because he doesn’t have a stable job.

I: Okay. So how do you cope?

R: With life?

I: Yes, with these challenges you are experiencing.

R: Okay, right away I used to work in a certain small hotel but abruptly the job stopped; there was some reconstruction where the road was being expanded making the job to stop. I’ve now been at home waiting for another job even the construction works.

I: What about coping without parental love?

R: Sometimes when I come here I can be encouraged on what to do and how should I cope with it; such advices have been pushing.

I: Who do advise and encourage you?

R: The doctors who are here.

I: Okay. So do you think the challenges you mentioned affect your educational achievement during the time you were going to school?

R: It really did… yeah.

I: How?

R: By that time, I didn’t have anything as begging was hard on my side. So like when I joined Form One and in Form Two I was at the top but all over sudden things changed, life became hard; nothing I can do. So sometimes I could just go without those things and could beg from my fellow students, some could give while others would want something in return which I also had nothing to give them back.

I: Okay. Other than the support you received from the doctors that encouraged you, has anyone else helped you with these challenges?

R: Yeah, like friends in the same condition as me, they do encourage me.

I: When you talk of the same condition as you, what do you mean by being in the same condition?

R: Being positive.

I: Speaking about HIV care services can you describe to me your experience the day you enrolled in HIV care?

R: I can't remember well but that I can remember is that I was very young when I came here. So one day… I was being told as a story; I came when I was small and was very sick and was admitted to JOOTRH, from there I enrolled (not clear 8:52-53) in that place. So me I never knew that I was positive until the day the doctors disclosed, this because the person who brought me here was not my relative and it’s at class five when I was told that I was sick, so it pained a lot because all along they used to tell me that… at first I was diagnosed with TB and was told after taking the drugs I would be well and so I took the drugs and finished them for a certain period to I asked them why I was taking the medication but was met with silence. I never knew why until the day that I came here and was asked the same question, “Why do you think you are taking these drugs?” I told them that I didn’t know and they told me that I was sick, I was in class five then.

I: Is it the same class five that you were diagnosed with TB?

R: That was when I was in the lower classes.

I: That is when you had TB. Just continue. Ok. So you knew your status well when you were in class five?

R: Yes, it really pained me because the persons I thought could tell me these thing early were not able to tell me.

I: Why would someone else (not clear 10:42)

R: By that time it were just my close friends then later my relatives; my parents died long time ago, when I was very young. So majority of our family members were still slow, by that time I think HIV was not that much known so they told me late. They came and took me to JOOTRH. That is when I knew my status.

I: So you were enrolled in HIV care without understanding what was going on?

R: Yeah, I never understood.

I: What has enabled you to continue engaging in care at this facility?

R: Okay, when I come I meet many people, besides that I feel that I’m not the only person living with that disease.

I: Other than that, Is there any other thing that does make you continue coming for the care?

R: Yes, there these advisors, these have really pushed far especially here in Lumumba; I really thank the doctors because if they weren’t there I wouldn’t have made it this far; they keep on advising me about taking my drug things, will be fine, everything will be okay and I will live normally. So I took the advices they gave and that’s why I’m where I am today.

I: How do you find the quality of services at the facility?

R: They are really good; very good.

I: Do you stay close by?

R: Not really.

I: But you still come all the way?

R: Yeah.

I: Okay. Can you tell me anything you know about ARVs?

R: What I know about ARVs, one is that when you take them carelessly they can harm you… when you take them without food they can weaken your body. Thirdly, when you don’t take them at the right time the viral load will shoot up.

I: Do you have any other knowledge on them?

R: They reduce the chances of viruses in the body.

I: They reduce chances or virus?

R: Like… The virus do not come active; when I’m positive and don’t take ARVs or take and skip or even leave them when I feel well will make the viral load shoot up and by the time I start taking the first or second level medication they will have to… there is nothing that these people can do so if it is the second nothing they could do; nothing at all…I do… I feel there is nothing they can do if it’s the second stage.

I: Tell me about the time you missed taking your ARVs.

R: I missed… when we were in music I remember… I was the one… I remember that we were going to Homabay and since I was coming from far I was forced to sleep in school, so we were converged in one class the bus taking us was very very early because we practiced up to late hours and the time passed so I never took.

I: For how many days did you not take?

R: It was only that day.

I: So you couldn’t because there were other people in the room with you?

R: Yes.

I: How did that made you feel?

R: I was very disturbed even though we were (not clear, talking in low tone16:34) because at times I was being that if you don’t take your ARVs there is nothing that you can do, so take the drugs well.

I: Can you share with me the challenges you have been facing keeping your clinic appointments?

R: I can just say that fare is…(silence)

I: What about fare?

R: At times we lack that I can't even be able to come so I… at times I do miss and following day I could start walking as early as six as I come here, go and just trekking.

I: Does this happen severally or once in a while?

R: Severally.

I: What do you do when you miss coming and don’t have any medication left?

R: Mostly I used to check on my drugs and check on the date so if you are not financially stable I’ll just know that tomorrow by this time I’ll be out and start walking coming here.

I: Was there any time that you were out of drugs, didn’t have the fare and didn’t walk to the facility also?

R: I’ve never done that; mostly they used to give me excess drugs so if I miss coming today I’d explain to them why. Mostly I used to make sure that I’m here even though other time I’d be late but at least I’m here.

I: When was the last time you missed coming?

R: Of late I’ve not missed.

I: When exactly did you miss? This month. Last month, early last year.

R: It was last year.

I: Tell me about it.

R: At times you ask people who live around you. Some may tell you that at around this time I’ll give you or send you the money, can wait up to when the time passes and you come here at around 1 p.m. when its late and given drugs to last you two weeks. On coming back again you may still lack the money, so it was very very challenging and so I knew… from that I remove my (not clear 20:08) so I’m like living… like this is my life so I could know when I am… but if I do something and get awarded I could keep that money to the day I’d be coming here at the hospital.

I: What are your general feelings about HIV care services you currently receive?

R: To me they are always good. I think some… there is a point I said to myself that if only this people; referring to Lumumba, they could build houses here where people with challenges like me could stay such that we don’t suffer that much. Also the advices they do give can push you somewhere.

I: Okay. What do you think can be done so that you can be satisfied from the HIV care you have been receiving?

R: Generally, you mean?

I: Yes. What can be done so that the services you receive can be better, or its find with you.

R: Okay, what they can do is that when we come early - at times I do come here early and live late, so what they can do is adjust on the time; when a patient come at this time he or she is supposed to leave at this so that another patient can be served and allow us to leave early.

I: Is it just the issue of time?

R: Yeah, only that.

I: Okay. For someone who has been in school recently, how does being in school affect young people like you from keeping their appointments and taking their ARVs?

R: You know… I’ll speak on my case. I used not to come on the exact time, like when I was released in half term I could come because I knew my school very well, I knew my friends who were close to me so I never wanted to suffer stigma when I was in high school. I tried very much for no one to know about my status but since… they came to realize when I was in Form Four and that was very challenging because at times the date would be set… Form Four is always working working working, exams here and there. So I missed some of those exams just to come here and take my drugs because there was nobody to come and get them for me, it was only me.

I: Were there times during exams that you were not able to come for your medications?

R: Yes, I remember there was a time I wasn’t able to until they sent someone from the office to come. The following day after they came we were so busy and things were changing so like… I was afraid like when I miss these exams and don’t go there, bringing a parent was very hectic, I was… it was just hard as no one was able to volunteer. So I was weighing because when I come late then it would be another case as I would have to explain where I went to, so it was very hard.

I: What about taking you’re ARVs in school?

R: I was in high school, day school.

I: So you took them only at night?

R: Yes.

I: Did you have any challenges with that?

R: Taking drugs at home?

I: Yes.

R: The person whom I was living with; I don’t know if I can say was courageous because at times we would leave things well set so that is the time I realized I was being stigmatized because I found a group of women and they exposed my status… she exposed my status and at time I could find the drugs left everywhere, wherever you came they would say… so even the children knew and would ask me why I was taking the drugs. At times I would just get quiet because I have no answer to give them… to go there then they will…

I: How were you related to the person who exposed your status?

R: I was staying with my bother but he was not… he used to work far and was never around, so the wife.

I: How did you feel about that?

R: It was very painful. It took like two months for me… for me, for that trauma to melt away from my heart.

I: Whom did she tell?

R: There are several women also living in that plot so that’s the issue.

I: Okay. Moving on sexual and reproductive health, what do you understand by the term sexual and reproductive health?

R: To my understanding I think it means that if someone is sexually active or the other way maybe related to that… diseases, that’s according to my understanding.

I: Which is fine. Have you ever accessed any reproductive information?

R: Not really.

I: What about safe sex?

R: Never

I: You have not been talked to about when someone wants to have sex, they need to do this so that they don’t contract diseases?

R: No, were only taught that in high school and primary school.

I: What about contraceptives?

R: Those we learnt in secondary school.

I: STIs and prevention?

R: Yeah.

I: Where did you learn about them?

R: Primary school.

I: So all this information you have received, you have access to them in school. How important is it to access this information on sexual and reproductive health?

R: It makes you be aware.

I: What do you become aware of?

R: You be aware of the diseases and how best you can prevent it.

I: Since you left school have you ever accessed this information on sexual and reproductive services?

R: No.

I: What about services have you ever accessed any services on sexual and reproductive health?

R: Services like?

I: Contraceptives use.

R: No.

I: You have not.

R: No.

I: Okay. What is your ideal place for acquiring these services and information on sexual and reproductive health?

R: The hospital.

I: Hospital. Why?

R: Because these people are more knowledgeable, they know the impacts and the benefits.

I: To what extent are the sexual and reproductive health services offered to young people like you meet their needs and expectations?

R: Where?

I: You told me that you access this in school right?

R: Yes.

I: Did you feel that your expectations were met concerning these topics?

R: No.

I: Why do you say so?

R: They were not able to answer some of the questions so that’s why we need specialized personnel who can elaborate everything.

I: Other than unanswered questions what other reasons do you feel your needs were not met?

R: Only that.

I: What challenges do you as a person face in trying to access this information on sexual and reproductive health services?

R: There is no challenge.

I: Can you access it right now when you want to?

R: Yes, I can.

I: From where?

R: Lumumba.

I: Okay, but you haven’t been accessing it?

R: Yes.

I: Any reasons why?

R: I just feel like time is not yet there.

I: Can you tell me more about that?

R: Let me talk about contraceptives. I’m not sexually active and so I don’t know the case of using these contraceptives. So the right time… when I think this is the right time I could just come and talk about it and get the help.

I: How does being in school for young people like you affect your access to sexual and reproductive health services?

R: It affects them in their education in that… for example… let me say, when I start using contraceptives at high school yet I want to attend those lessons, I won't because I know, I won’t concentrate not mac you contraceptive like condoms and family planning have their impacts on their own, so you can't keep them together with education; like you want to do this and that.

I: So anyone can’t balance between?

R: Yeah it is impossible.

I: Ok. How comfortable will you be discussing your sexual and reproductive health concerns with a lay female health care provider of your mother‘s age?

R: I’d be very open.

I: What would make you open to talk to an older woman?

R: We are in the same gender and there is nothing I can hide because maybe she has gone or has seen more than me. That is why I can be open and tell her everything so that if she has other advices or encouragement she could share it with me.

I: What about discussing with a female health care provider who is your peer?

R: It would also be fun.

I: What’s fun about it?

R: Maybe she has undergone certain problems that has made her use contraceptive or other things which I have not used, so she will advise me on what to do and what not to and their repercussions. That’s why it’s fun.

I: Can you point out the issues that you would comfortably discuss with this woman of your mother’s age and also a female healthcare provider?

R: First it would be the challenges affecting us, second it would be how to curb those problems or challenges we are facing.

I: Which challenges to the specific?

R: Let’s say like early pregnancies; what drives a woman to be pregnant and besides that abortion so maybe we can discuss deeper on the topic.

I: What issues can you discuss with your peer?

R: We could discuss the same topics and even the related diseases.

I: Where would you want to access this information on sexual and reproductive health?

R: The hospital.

I: Why the hospital?

R: I think it’s safer and able to handle confidentiality.

I: How would you feel if we assigned you a lay heath care provider to be your confidant and source of healthcare information for you sexual and reproductive needs?

R: I have no problem.

I: Why so?

R: I would give them the best service; those people who require it and some of the things they should expect when we are giving them those services.

I: I am not sure if I get you. What I am trying to ask is that if we give you a healthcare provider who you can talk to, who you can go and seek services from or if you have any concerns you just go and talk to her.

R: If the person is secretive or cannot expose the things we discuss or may be the things she may be assisting me with, then I’d be okay. If not I’d rather go to someone who I feel like cannot expose anything cannot expose anything.

I: Other than that what other quality would you want this person to possess?

R: Friendly, encouraging.

I: Okay. What would you feel about receiving ARVs together with your sexual and reproductive health services? When you come for ARVs then you also receive sexual reproductive health services if you want?

R: It would be very fine because coming for the service concurrently will save time and fare of coming back another time for individual services should they be separated.

I: Any disadvantage of receiving the services concurrently?

R: Maybe let’s say like the person who was giving me those services never knew that I was sick… something of that sort or maybe he or she is… sees or feel like that you are sick. Maybe expose, maybe they never knew the person well. Yeah

I: Saying the person didn’t know, is this someone who knows you personally or just a random person?

R: No, like… like there we are used to those doctors and so maybe if a new face appears to give me those services and I don’t know him… I don’t know anything about you or maybe you used to see me somewhere and never knew I was sick then I come here and you give the services and then from there you start.

I: What if the people offering these services are just the same people you’ve been seeing but now they want to incorporate these services with your ARVs appointment dates?

R: It is very fine.

I: Thank you so much for your time for talking to us today, do you have any questions up to now?

R: I have a question; is this end of the interview?

I: Yes.

R: OK.

I: That is the question (both laugh). Okay. We have come to the end of the interview and time is 12:50 p.m.
