# Supplementary material for: Preferences for accessing sexual and reproductive health services among adolescents and young adults living with HIV/AIDs in Western Kenya: A qualitative study
Source: PLoS One. 2022 Nov 16;17(11):e0277467. doi: 10.1371/journal.pone.0277467 (PMC9668131; doi:10.1371/journal.pone.0277467)
Supplement: Attachment — Submitted filename: Response to Reviewers.docx [file pone.0277467.s004.docx]

**Letter of Response to Editors and Reviewers**

PONE-D-21-29137

Manuscript Title: Preferences for accessing sexual and reproductive health services among adolescents and young adults living with HIV/AIDs in Western Kenya: A qualitative study.

Dear PLOS ONE editors,

We would like to thank the editors and reviewers for their important comments on this manuscript. We have edited the manuscript in response to the comments and provided both a marked-up and a revised copy of the manuscript as instructed. The authors hope that the editors will be satisfied with the changes we have made to the manuscript. We feel the comments and revisions to the manuscript have made it stronger.

Below please find each comment with our response indented and in bold print:

1. Please ensure that your manuscript meets PLOS ONE's style requirements, including those for file naming. The PLOS ONE style templates can be found at
   <https://journals.plos.org/plosone/s/file?id=wjVg/PLOSOne_formatting_sample_main_body.pdf> <https://journals.plos.org/plosone/s/file?id=ba62/PLOSOne_formatting_sample_title_authors_affiliations.pdf>

**We have revised the manuscript to meet PLOS ONE’s style requirements including file naming.**

2. We note that you included minors (age<18) in your study. Please provide additional details regarding minor’s consent. In the ethics statement in the Methods and online submission information, please ensure that you have specified whether you obtained consent from parents or guardians. If the need for consent was waived by the ethics committee, please include this information.

**We have provided additional detail regarding minor’s consent. Assent was obtained for adolescents below 18 years, and their parents/caregivers provided written consent (Line 189-190).**

3. Please update your submission to use the PLOS LaTeX template. The template and more information on our requirements for LaTeX submissions can be found at <http://journals.plos.org/plosone/s/latex>.

**We have updated our submission based on the PLOS LaTeX template.**

4. Thank you for stating the following financial disclosure: "This study was an internally funded at Kenya Medical Research Institute.
MON is the author who received the award. Sponsors and funders did not play a role in the study design, data collection and analysis, decision to publish, or preparation for the manuscript."

We note that one or more of the authors is affiliated with the funding organization, indicating the funder may have had some role in the design, data collection, analysis or preparation of your manuscript for publication; in other words, the funder played an indirect role through the participation of the co-authors. If the funding organization did not play a role in the study design, data collection and analysis, decision to publish, or preparation of the manuscript and only provided financial support in the form of authors' salaries and/or research materials, please do the following:

a. Review your statements relating to the author contributions and ensure you have specifically and accurately indicated the role(s) that these authors had in your study. These amendments should be made in the online form.

**Below are the author/co-author roles. These changes have been updated in the online submission form.**

1. **HAF was a Research Assistant in the project. Her roles included conducting participant interviews, transcription, coding and analysis.**
2. **ZK and MON-Principal Investigators**

**ZK and MON provided oversight of research development and implementation. ZK was directly involved in leading and supervising the qualitative activities including focus group and individual interview guide development, conducting interviews, coding/analysis and results dissemination.**

**MON was involved in the management of local regulatory supervision and administration of the study.**

**All authors reviewed and approved the manuscript.**

b. Confirm in your cover letter that you agree with the following statement, and we will change the online submission form on your behalf:
   “The funder provided support in the form of salaries for authors [insert relevant initials] but did not have any additional role in the study design, data collection and analysis, decision to publish, or preparation of the manuscript. The specific roles of these authors are articulated in the ‘author contributions’ section.

**We agree with the statement, and we have updated the cover letter to reflect this.**

5. In your Data Availability statement, you have not specified where the minimal data set underlying the results described in your manuscript can be found. PLOS defines a study's minimal data set as the underlying data used to reach the conclusions drawn in the manuscript and any additional data required to replicate the reported study findings in their entirety. All PLOS journals require that the minimal data set be made fully available. For more information about our data policy, please see http://journals.plos.org/plosone/s/data-availability.

Upon re-submitting your revised manuscript, please upload your study’s minimal underlying data set as either Supporting Information files or to a stable, public repository and include the relevant URLs, DOIs, or accession numbers within your revised cover letter. For a list of acceptable repositories, please see http://journals.plos.org/plosone/s/data-availability#loc-recommended-repositories. Any potentially identifying patient information must be fully anonymized.

**We initially uploaded transcripts and would want to request if they could be withdrawn so that we upload the in-depth and focus group discussion guides as suggested by Reviewer #1.**

Important: If there are ethical or legal restrictions to sharing your data publicly, please explain these restrictions in detail. Please see our guidelines for more information on what we consider unacceptable restrictions to publicly sharing data: http://journals.plos.org/plosone/s/data-availability#loc-unacceptable-data-access-restrictions. Note that it is not acceptable for the authors to be the sole named individuals responsible for ensuring data access.

We will update your Data Availability statement to reflect the information you provide in your cover letter. **Thank you**

6. We note that you have stated that you will provide repository information for your data at acceptance. Should your manuscript be accepted for publication, we will hold it until you provide the relevant accession numbers or DOIs necessary to access your data. If you wish to make changes to your Data Availability statement, please describe these changes in your cover letter and we will update your Data Availability statement to reflect the information you provide.

**We have uploaded the files containing in-depth interviews and focus group discussion transcripts. Should our manuscript be accepted for publication, we allow the journal to use these files as repositories.**

7. Please include captions for your Supporting Information files at the end of your manuscript, and update any in-text citations to match accordingly. Please see our Supporting Information guidelines for more information: <http://journals.plos.org/plosone/s/supporting-information>

**Reviewers’ Comments**

Reviewer #1: Apologies to focus on the negatives, there was clearly good and necessary research in this document, but my comments are below. My biggest concerns are CONFIDENTIALITY BREACHES OF PEOPLE LIVING WITH HIV IN THE SUPPLEMENT document.

Ethics:
Two major points of concern:

1. The information in the supplementary information is inappropriate. I only read a tiny bit of it and already had a young person’s town, school name, their age, school year, employment, sex, and their intentions to go to university. IT IS THEREFORE POTENTIALLY ENTIRELY IDENTIFIABLE to anyone who knows these young people. This error makes me question data governance in this project. In my opinion, local actions need to be taken to investigate this confidentiality breach. The interview transcripts should never in my opinion be published in their current form. A suitable alternative would be to append the interview and focus group discussion topic guides.

I think that the interview and focus group topic guides should be appended rather than the interviews themselves in the form that they are in.

**We apologize. This was an oversight on our end. We mistakenly uploaded our raw files instead of separate transcripts that did not contain participant identifying information. Based on the reviewer’s advice we are withdrawing the transcripts and instead submitting the interview guides.**

2. Why were healthcare workers and caregivers asked if they were sexually active? This does not seem an appropriate question as the study was NOT about their sexual behavior. Was the ethics team aware that this would be asked of these groups of people? In my country this would be inappropriate data to obtain in this context, as it does not relate to the research question. Again, I question whether training on data governance needs to be strengthened in the reporting team.

**We apologize if this was not clear. The question on sexually active was meant for adolescents and not healthcare workers**

General feedback:
The use of the word "facility" throughout this piece had me confused and needs some clarity.
I would want the interview and FG topic guides as appendices (and not the transcripts, for reasons above)

**In Kenya, hospitals are commonly referred to as health “facility.” We have replaced the word “facility” with HIV clinic or hospital based on the context.**

Abstract:
“AYAs generally preferred receiving SRH services from the health facilities offered and served by trained healthcare providers.” – having read the whole paper, I am not sure I have seen evidence of the “trained healthcare providers” bit of this statement? I think maybe the abstract should just say that AYAs generally preferred SRH services to be co-situated with clinical facilities? Your data seems to support the idea that healthcare providers feel that training is important.

**Thank you for this comment. We have revised the abstract and the discussion to reflect preference of SRH services to be co-situated with clinical facilities (line 19).**

Introduction:

A Key issue: I think for me the article would be a bit easier to understand if a bit more of the context was given about the presence of PEPFAR and its projects and centers for AYA living with HIV, their relationship to state-provided healthcare, and therefore the policy need for research of this type.

**We gave some context on PEPFAR in the study setting section since the hospitals in which we conducted the study are supported by PEPFAR-funded HIV care programs. We have revised the section to include additional roles and the relationship with state-provided healthcare (line 139-140)**

“Like many countries in SSA, Kenya still grapples with challenges in improving the utilization of adolescent sexual reproductive health services that urgently need focus” – Is focus a big issue for healthcare providers? I think this needs to be made clearer.

**We have clarified in the introduction section that the SRH services need urgent focus by stakeholders engaged in adolescent sexual and reproductive health (Line 87-89).**

The other clear difference between AYAs living with and living without HIV is that the latter group is already in regular contact with healthcare professionals for their HIV. In many countries these professionals would feel confident in advising the AYA about SRH needs, certainly in giving out condoms, or signposting them to SRH-specialist services. The challenges here are that some services may have known the AYA since they were a child, and then the child feels that they cannot discuss their sexuality with them, and that the HIV team will have an interest in discussing disclosure with the AYA. A fresh or unknown staff member from an SRH team can be helpful in these situations. I didn’t hear much about this angle from the introduction and would like to know how things are in Kenya and why the patients’ own HIV teams aren’t able to cover their SRH needs. Or are they? Are they meant to? It would be really useful background information to have, as then the other questions and statements would make more sense.

**From a review of the literature, SRH staff members are hesitant to discuss with AYA living with HIV/AIDs matters on sex and contraceptives due to cultural norms. The same applies to some healthcare workers in Kenya, they believe AYA are not supposed to engage in sex and or use contraception before they are married hence AYA feel they are judged with these healthcare providers when they access SRH services. Indeed an unknown staff member would be helpful in breaking this barrier particularly among AYA who have been in care from. We have revised paragraph 3 of the introduction to reflect some of these ideas (Line 80-85).**

Methods:
Unclear how patients were consented, especially regarding the language they were consented in, language of consent form, and were the participants clear that participation didn’t affect the care they were receiving, as PLWHIV? I note some people had only “some primary” education – were measures put in place to support those who were illiterate?

**Participants were consented in their preferred language i.e. either English, Swahili, or Dholuo and assured that their participation in the study would not interfere with the care they were receiving at the HIV clinics. We have updated line (192-195) in the manuscript.**

**Despite just having some primary education, participants were able to comprehend the questions being asked in the language of their choice and responded well to the questions. The research assistants clarified questions in cases where participants seemed not to have a clear understanding of what was asked. We did not have cases of illiterate participants’ who needed extra support.**

Data security of recordings etc. is not mentioned.

**We indicated in the manuscript (Line 232-234) that the audio files from the interviews were downloaded onto a password-protected study computer and the same backed up on external drives and kept off-site with restricted access.**

Unclear in methods section why broad code groups were given in this section– would usually be in results section unless the codes were a priori codes, furthermore, it is not clear if these codes were a priori codes or not and this needs to be clarified.

**We used a combination of inductive and deductive methods to derive the codes and the codebook which means some of the codes were a priori. We have updated this information in line 241-242 in the manuscript and omitted the broad code groups earlier mentioned.**

Did the interviewer sex match the participants? what training had the research assistants had? Abstract mentions they were trained and experienced, but this isn’t in the text, which is a bit unusual as typically there shouldn’t be anything in the abstract that isn’t inside the main article. Training is especially important as discussing sex with very young people.

**Thank you for this comment. The research assistants were trained in qualitative methods and handling adolescents and had accumulated more than 3 years’ experience working with adolescents and young adults at the SRH clinics. We have revised line 202-204 in the manuscript to reflect this.**

Also, how old were the interviewers, given your research’s findings that young people prefer to talk to younger staff?

**The interviewers were 5-10 years older compared to the participants but were well trained and experienced in handling adolescents including, establishing rapport and making adolescents comfortable to talk.**

Were the FGDs held with boys and girls together – if so, it would be interesting to know what drove this decision?

**We conducted two FGDs with male adolescents, two with female adolescents, and one FGD held with boys and girls together to get the dynamics of the discussion while together. The FGDs with health workers and caregivers had a combination of both male and female participants. Our experienced moderators managed the discussions to minimize dominance within individual participants as well as between gender (i.e. males or females)**

Where were interviews held? What efforts were made to keep the conversations from being heard?

**The interviews were held in private and secluded rooms away from patient and clinical staff traffic. The participants were assured of their privacy and informed that they will pause discussions if there was to be any intrusion (191-195).**

Were parents informed of the interviews? how were issues such as school attendance navigated for these interviews, to encourage participation and minimize impact on life?

**Parents/ caregivers of participants below 18 years provided consent for their children’s participation in the study and were involved in scheduling of interviews of their children. The study was mostly conducted during school breaks and some during their clinic appointment dates. Scheduling with other participants was based on their availability. Hence, participation in the interviews did not have any interference with the participant’s schedule/activities.**

Were there any pilots performed? If so, should be mentioned.

**We did not perform pilot studies**

What proportion of those approached agreed to participate, if known? If the number of people invited is unknown, this should be stated, and why it is unknown.

**Among the 33 AYA who were approached to participate in the in-depth interviews, 30 participated. Two were not interested whereas one did not show up for the interview. However, all participants mobilized for FGDs participated (Line 174-177).**

I am not clear whether the AYAs and the carers were known to each other or not, i.e. were the carers picked because they cared for the specific AYA being interviewed?

**They were not necessarily parents of AYAs recruited in the study, but they were known to each other.**

How were carers and healthcare workers approached to be in the study, I couldn’t see this?

**A list generated from the adolescent reception module contained contacts of all AYA’s parents/caregivers. The staff used the list to approach parents who accompanied the AYA’s during clinic visit and called those who did not attend clinic. The clinic in charges approached the healthcare workers in each department and informed them about the study. Those interested were referred to the Research Assistants and the FGDs were scheduled. (Line 167-172)**

How was the number 30 chosen? I am not clear at what point saturation was found – was it after all 30 had been interviewed or before then? The wording isn’t quite clear. It reads more like 30 was chosen as it was expected that by this point saturation would have been reached, which is not quite the same thing as reaching thematic saturation.

**Initially we were approved to conduct up to 40 in-depth interviews and 16 FGDs. However, we reached theoretical saturation after 30 IDIs and 8 FGDs.**

Were all 3 FGDs asking the same questions, or was each group tackling different questions? Also, how many people were in each FGD?

**All the three FGDs asked similar questions.**

**Results**

What groups were the healthcare workers from? E.g. what proportion was nursing, doctors, etc.

**The healthcare workers included were nurses (18.75%), clinical officers (18.75%), Pharmacy technologists (12.5%), Laboratory technicians (12.5%), Community health Assistants (18.75%), and Peer Educators (18.75%). Line 278-280 updated**

What proportion of the patients were living with HIV?

**All adolescents who participated in the IDI and FGD were HIV positive and enrolled in care across the two hospitals. However, we did not collect information on the HIV status of the caregivers and healthcare workers.**

And what proportion of the patients participated in group discussions? Did all the AYAs have IDIs and FGDs?

**The two groups were mutually exclusive. Those who participated in the IDIs were not invited to participate in FGDs and vice versa.**

At least one male in the staff group seems to have (perhaps understandably as presumably he was indicating that he is a father) said that he was previously pregnant/expectant – this raises questions about whether the question was relayed to all participants in the same way? It is probably worth unpicking from your data and commenting to explain how this happened (or changing the way the question is described in the table).

**Thank you for this comment. The question asked was, “How do people react if a young woman becomes pregnant/a young man becomes a father?” The question was relayed to the participants the same way. The said participant meant he was a father.**

I would have liked a general paragraph in the results to outline how the FGDs had been – was there discord? How much did people disclose? Did boys or girls, older or younger people dominate discussions in the FGD for AYAs? Were staff in general agreement or were there differences eg. based on rank or location of their work? Was there a lot of difference in the views raised at the 3 groups? Were the caregivers able to give many opinions? What were the main concerns within the 3 groups of people?

**We did not observe any noticeable differences between the views of boys and girls, caregivers and healthcare providers-subgroup analysis. We have included this statement on line 281-282.**

Why are the IDIs so underrepresented in your quotes? It seems a shame that you did ?30 IDIs and they only get 2 quotes.

**We have included additional quotes from the IDIs in the results section. See line 300 - 315, 321-325, 450-453, and 480-486.**

Preferences sections:

Were the locations discussed (schools, hotels, pharmacies etc) prompted from a list/interview or FG prompt or spontaneously mentioned by the participants in the different groups?

**The responses were mostly spontaneous based on the question “where do you prefer to receive SRH services and why?”**

“If you access those other things, maybe there are not good places you can get from them. But here, they will tell you the good ones and the bad ones (IDI #2 at KCH)” I think this quote needs some extra words in square brackets to help it make sense as at the moment I don’t understand it, even within the context given in the text

**We have included additional quotes from the participant and an explanation in brackets to give context. (Line 350-355)**

“I feel that it would be beneficial because I have seen community health volunteers who talk to children, and they open up. I think the issue of age makes them have confidence in you (FGD on 23 August 2019” – this quote needs to say which of the 3 FGD groups this quote came from-specify if adolescent female, male, healthcare provider or carer

**The quote came from the FGD with caregivers. We have updated this information (Line 341).**

“Others were inclined to have a trained professional go to schools on specific days to provide SRH services and offer psychosocial advice. However, other participants had a contrary opinion arguing that if teachers were to be assigned to provide the services, they would be judgmental and deny AYAs services with the reasoning that they are young and they should not be accessing such services.” - I do not find these two statements contradictory unless one assumes that the trained professional going to the schools is a teacher (and I don’t know why they would necessarily be?). there is also quite a lot of duplication of this paragraph in the following quotation so it could be trimmed.

**We have revised the paragraph based on the suggestion to read as below:**

**Others were inclined to have a trained professional go to schools on specific days to provide SRH services and offer psychosocial advice. Others argued that if teachers were to be assigned to provide the services, they would be judgmental and deny AYAs services because they were young.**

“elderly” doesn’t really feel like the correct word, I think, where you have used it. Perhaps just “older” would be better?

**Thank you for the suggestion. We have replaced the word “elderly” with “older” in all sentences we have used it**.

Throughout the section on preferences of counsellors I wasn’t getting a very good idea of which groups of people were thinking what in terms of the preferences. This needs a bit more clarity (even if they were all in agreement, for example).

**We have specified the specific group in terms of preferences and stated that the preferences cut across the three groups (AYA, healthcare providers, and caregivers) during IDIs and FGDs. We also included additional quotes to illustrate.**

“receiving services at the facility” – this could be clearer eg. by stating “within a hospital building” (as to me, facility could mean anything including a school for example). I think this terminology is in the abstract as well and would just be clearer if it used more precise language.

**We have replaced the word facility with hospital and HIV clinic as appropriate.**

Key point: Did the IDIs concur up with the FGDs on your key findings (ie. preferred gender and age of care provider for girls and boys, and preference for SRH services co-situated at a venue like a clinic or hospital)? Did the different FGDs generally concur with this or were some more in agreement with your conclusion and others less so? I think this needs a bit more fleshing out as it is interesting if there are trends (rather than just the odd quote).

**The IDIs concurred with the FGDs on key findings. There were no noticeable differences.**

**Discussion:**

I don’t feel from reading your results that there was a strong preference for hospital or clinic co-situated SRH facilities, among the AYAs; rather that there was a large mix of views and maybe the predominant view was one of reference for co-situated SRH services (the word you use is “many”). I feel the discussion and abstract implies there was a fairly clear preference for healthcare co-situated SRH services. Therefore I suggest that the results section needs to justify the conclusion that there was an overall preference (eg. by stating that a majority of participants said it (please also see above note about showing more clearly if it was in FGD or in IDIs that this conclusion was most evident; AYAs or staff/caregivers)), or the discussion/abstract need to decrease the weight currently placed on preference for SRH being co-situated with healthcare (eg by saying the most commonly voiced preference by AYAs in ___(FGD/IDIs) was x”).

**Thank you for this comment. We have revised the discussion section to capture the most common voiced preference by the AYA’s and included additional quotes for illustration.**

“AYAs generally preferred receiving SRH services from the health facilities offered and served by trained healthcare providers.” – having read the whole paper, I am not sure I have seen evidence of the “trained healthcare providers” bit of this statement? I think maybe the abstract should just say that AYAs generally preferred SRH services to be co-situated with clinical facilities? Your data seems to support the idea that healthcare providers feel that training is important.

**Thank you for this comment. Based on the findings, we have revised the abstract and manuscript to say that AYAs generally preferred receiving SRH services co-situated with clinical facilities (Line 432-433).**

“One of our main findings was the need to design SRH interventions that ensure all services the AYAs require are “under one roof."”. This may be true, but you don’t evidence it much if it is a main finding. For example, there are no quotes from AYAs illustrating this (the quote that mentions parents not knowing what services are being sought does not illustrate the desire for a “one stop shop” with multiple services for the AYA, merely the fact that a health facility masks the SRH activity by also offering non-SRH activity that the person does not intend to use).

**We have included quotes from IDIs and FGDs to illustrate that the participants’ preferred receiving all services under one roof at the hospitals.**

**The quote that mentions parents not knowing what services are being sought illustrates that since the AYA cannot share with the parent/carer regarding the need to access SRH services, going to a hospital with services under one roof allows them to access SRH services in the pretense that they went for treatment of other health conditions.**

The sentences about the youth centers are interesting, but I don’t really see how they link in with the “under one roof” idea very clearly – are they co-sited with HIV clinical teams? This needs greater clarity.

**The youth centers are co-situated within the HIV clinic. These are specifically meant to serve AYA up to the age of 24 years before they transition to adult HIV clinic. Having youth centers with all services under one roof reduces the potential loss of participants as they move from one clinical room to another, and stigma associated with receiving certain services at designated clinical rooms.**

I’m not sure what a “stigmatizing outburst” is – needs greater clarity/rephrasing.

**We have replaced “stigmatizing outburst” with stigma (Line 589).**

“These findings highlight the importance of having a ‘safe space’ environment for the provision of SRH services within our health facilities and interventions that will improve access to the existing SRH service provided to adolescents living with HIV/AIDs at the adolescent centers.” Who does “our” refer to? And where would the safe spaces be? Within PEPFAR or within the health centers?

**“Our” referred to the HIV clinic. We have omitted the word. We meant a conducive environment (safe spaces) within the HIV clinics at the hospitals.**

It would be interesting to hear why you think that girls preferred younger, and men older, healthcare professionals – is there any theory you can link with?

**From our review of literature most theories we have come across focus on gender preferences, but we have not come across one that specifically points out to why girls would prefer younger and men older. However, we think that girls preferred younger healthcare professionals because they are approachable, the female AYA’s are free to share with them and have recent experience transitioning from adolescent to adulthood. In most cultures from different ethnic communities in Kenya, males are mentored on social roles and community cultures by older men therefore, there is likelihood that the male AYA would consider having an older man for their SRH needs.**

Typos:
AYA, SRH, SSA, IDI, FGD – define when first mentioned, not later in the document

**Defined and updated in the manuscript.**

“20 million girls aged (15-19 years)” should be “girls (aged 15-19)”

**We have updated the manuscript to reflect this change.**

“if they were aged 14-24, living with HIV and on (in) care at the two facilities” [should read IN care]
 **We have updated this in the manuscript**

I can see that orphan/total orphan is a recognized term, but it is a bit confusing – suggest it might help readability to change to “one/both parents deceased”

**Thank you for this suggestion, we have replaced orphan/total orphan with one/both parents deceased.**

“This ensured that participants were free to discuss sensitive issues” – suggest you might mean “felt comfortable”?

**Yes, meant felt comfortable. The revised sentence reads this, “This ensured that the participants felt comfortable discussing sensitive issues”**

Not sure if a typo – what are “health families” p.15 on my document?

**We apologize, this was a typo. We meant health facility. We have revised to read hospital.**

“This study explored the perspectives of adolescents, caregivers, and health care providers on improved access to sexual and reproductive health services of AYAs living within western Kenya.” Currently this reads as though the services are already improved and people are being asked about them – I’m not sure this is what you mean though.

**We have replaced the word “improved” with “improving”**

“horrible staff attitude” – suggest you mean “disrespectful staff attitudes” or something similar. “Horrible” is not an appropriate word for a journal, unless a quotation.

**Thank you for this suggestion. We have replaced “horrible staff attitude” with “disrespectful staff attitudes”**

“In as much as AYAs in our study had a strong preference for receiving their SRH services at health facilities, some preferred other outlets like retail pharmacies” – this doesn’t make sense to me linguistically and I think you mean “Although most of the AYAs in our study had a strong preference for receiving their SRH services at health facilities, some preferred other outlets like retail pharmacies”. Thank you for this suggestion. This was an error. We have revised the sentence to read as below:

**“Although most of the AYAs in our study had a strong preference for receiving their SRH services at health facilities, some preferred other outlets like retail pharmacies”**

- **Reviewer #2:**

Well written and excellent manuscript of an important issue around AYA. The introduction however needs to be revised on various aspects- grammar, references and literature.

**We have revised the introduction section of the manuscript as advised.**

The factors outlined as contributing to inadequate access to SRH do not only apply to ALHIV but to all adolescents in general. The distinction between these two groups and the differences needs to be outlines as well as the possible reasons. More literature is needed in the background regarding preferences of healthcare providers and caregiver. More results could be included as well. Also in the discussion comparison with other studies could be made stronger.

**Thank you for the suggestions. We have revised the background to include more literature (line 76-85), added more quotes to illustrate our findings in the results section and modifications in the discussion.**
