# Supplementary material for: Preferences for accessing sexual and reproductive health services among adolescents and young adults living with HIV/AIDs in Western Kenya: A qualitative study
Source: PLoS One. 2022 Nov 16;17(11):e0277467. doi: 10.1371/journal.pone.0277467 (PMC9668131; doi:10.1371/journal.pone.0277467)
Supplement: Attachment — Submitted filename: Response to Reviewers 2022-10-25.docx [file pone.0277467.s005.docx]

**Letter of Response to Editors and Reviewers**

25^th^ October 2022

PONE-D-21-29137

Manuscript Title: Preferences for accessing sexual and reproductive health services among adolescents and young adults living with HIV/AIDs in Western Kenya: A qualitative study.

Dear PLOS ONE editors,

We want to thank the editors and reviewers for their important comments on this manuscript. We have edited the manuscript in response to the comments and provided both a marked-up and a revised copy of the manuscript as instructed. The authors hope that the editors will be satisfied with the changes we have made to the manuscript. We feel the comments and revisions to the manuscript have made it stronger.

Below please find each comment with our response indented and in bold print:

1. Table 1 shows a total of 9 female healthcare workers and also says that ten healthcare workers had ever been pregnant/expecting - perhaps if the question was "whether you or a partner had ever been pregnant" (is this what you mean by the question "ever been pregnant/expecting"?) was what was interpreted of this question by participants? It's not the biggest point, but your paper will appear more reliable if it doesn't seem to be saying that men were saying they had been pregnant (I'm assuming no transgendered individuals here).

**The question was asked across both genders. Whether females had ever been pregnant/expectant or if they were male if they had ever made someone pregnant. However, we have amended the table by omitting the proportion of caregivers and health workers who were pregnant/expectant since it doesn't add much value** (**Table 1 revised).**

2. As I suggested, please include age characteristics of interviewers in the article text (apologies if I missed it).

**Apologies for the oversight. We responded in the rebuttal letter but failed to include it in the manuscript. We have included it on line 12 and line 145.**

3. Please make explicit, as you said to me in response to my comments, that the FGDs and the IDIs concurred with each other around your overall conclusions and that you did not see any significant differences between the AYAs/caregivers/healthcare workers on where SRH services were best provided. This is an important finding.

**Thank you for this comment. We have included the statement in our overall conclusion. Line 439-449.**

4. line 250 - the quote following this sentence about AYAs' expressed preferences seems to have been from an adult, so it doesn't illustrate the expressed preference, only another person's assertion about their preferences (minor point).

**We have replaced the quote with two quotes from AYA IDIs (Lines 261-267)**

5. Darting back to the methods section, I think that what you told me about how you conducted interviews during school break times to accommodate the AYAs' other needs and to aid their ability to participate in something I would suggest including in the text. This demonstrates good practice for other research groups (I couldn't see it - apologies if it is there).

**Thank you for this comment. We have included this statement on lines 124-127**

6. I would like to see your hypotheses for why adolescent boys like getting SRH care from older men and adolescent girls from younger women explored in the document - you have fed back some ideas to me but not put them as hypotheses in the discussion as far as I can see (apologies if I missed it). Cite evidence where you can, but ultimately this is a potentially interesting avenue for future research, which you should point out. Your standpoint within a similar cultural milieu of the AYAs means that your interpretation is far better than, say, mine would be, and it is interesting and useful!

**We have included the hypothesis in lines 419-424**

7. Darting to the interview and FGD guides - were all questions used for all interviews and all FDGs, or did you only say focus on one or two areas for each FDG, say? it seems like a lot of questions to get through in quite a short time. Please outline whether all question areas were covered in all interviews and FGDs, or whether the FGDs and interviews tended to pick up on just a few areas. To my mind, it doesn't matter which is true, but I think it needs to be stated.

**We had guides for each of the two data collection methods, i.e. FGD and IDI. Updated on Lines 152-153**

Thank you again, these are my only thoughts now, and they are quite minor, great job!

**Thank you so much for the comments; they were very insightful.**
